# Histone deacetylase 4 reverses cellular senescence via DDIT4 in dermal fibroblasts

**DOI:** 10.18632/aging.204118

**Published:** 2022-06-09

**Authors:** Yuri Lee, Min Ji Song, Ji Hwan Park, Mi Hee Shin, Min-Kyoung Kim, Daehee Hwang, Dong Hun Lee, Jin Ho Chung

**Affiliations:** 1 Department of Dermatology, Seoul National University College of Medicine, Seoul, Republic of Korea; 2 Department of Biomedical Sciences, Seoul National University Graduate School, Seoul, Republic of Korea; 3 Institute of Human-Environment Interface Biology, Medical Research Center, Seoul National University, Seoul, Republic of Korea; 4 Department of New Biology, DGIST, Daegu, Republic of Korea; 5 Department of Biological Sciences, Seoul National University, Seoul, Republic of Korea; 6 Institute on Aging, Seoul National University, Seoul, Republic of Korea

**Keywords:** cellular senescence, DNA damage-inducible transcript 4, histone deacetylase 4, oxidative stress, ultraviolet light

## Abstract

Histone deacetylases (HDACs) remove acetyl groups from lysine chains on histones and other proteins and play a crucial role in epigenetic regulation and aging. Previously, we demonstrated that HDAC4 is consistently downregulated in aged and ultraviolet (UV)-irradiated human skin *in vivo*. Cellular senescence is a permanent cell cycle arrest induced by various stressors. To elucidate the potential role of HDAC4 in the regulation of cellular senescence and skin aging, we established oxidative stress- and UV-induced cellular senescence models using primary human dermal fibroblasts (HDFs). RNA sequencing after overexpression or knockdown of HDAC4 in primary HDFs identified candidate molecular targets of HDAC4. Integrative analyses of our current and public mRNA expression profiles identified DNA damage-inducible transcript 4 (DDIT4) as a critical senescence-associated factor regulated by HDAC4. Indeed, DDIT4 and HDAC4 expressions were downregulated during oxidative stress- and UV-induced senescence. HDAC4 overexpression rescued the senescence-induced decrease in DDIT4 and senescence phenotype, which were prevented by DDIT4 knockdown. In addition, DDIT4 overexpression reversed changes in senescence-associated secretory phenotypes and aging-related genes, suggesting that DDIT4 mediates the reversal of cellular senescence via HDAC4. Collectively, our results identify DDIT4 as a promising target regulated by HDAC4 associated with cellular senescence and epigenetic skin aging.

## INTRODUCTION

Cellular senescence is a permanent cell cycle arrest induced by endogenous and exogenous stresses, including DNA damage, organelle stress, and telomere dysfunction [1, 2]. Senescent cells activate senescence-associated secretory phenotype (SASP) and amplify the impact of proliferative arrest. Cellular senescence contributes to aging and is associated with age-related diseases and impaired tissue regeneration [1]. Senescence-associated β-galactosidase (SA-β-gal), which is present only in senescent cells but not quiescent or pre-senescent cells, is widely used to detect senescent cells. Increased p21 and p16 levels have also been used as markers of senescence [3]. While p21 is important for establishing senescence, p16 is involved in maintaining the senescence phenotype [3]. Another characteristic feature of senescent cells is their abnormally enlarged and flattened morphology. SASP components can also be used to evaluate senescence [1, 3].

Many studies have attempted to control aging through epigenetic regulation [4–8]. Epigenetic changes can regulate gene expression without DNA sequence changes and are thought to influence cellular senescence, aging, and age-related diseases [7, 9]. As aging occurs, various epigenetic alterations, such as aberrant histone modifications, alterations in DNA methylation patterns, and genomic instability, take place. These aberrant epigenetic processes can promote inflammation, cancer, and other diseases [7, 9, 10].

Histone acetylation is a major epigenetic modification, and changes in histone acetylation patterns are associated with aging [11–14]. Histone acetylation is mediated by histone acetyltransferases (HATs), whereas histone deacetylation is by histone deacetylases (HDACs). In mammals, 11 HDACs (HDAC1–HDAC11) and seven SIRTs (SIRT1–SIRT7) have been identified as histone deacetylases [15, 16]. Our previous studies showed that among these HDACs and SIRTs, HDAC4 was significantly decreased in UV-irradiated human dermal fibroblasts, aged human skin *in vivo*, and UV-irradiated human skin *in vivo* [17, 18]. HDAC4 is a key member of Class II HDACs and is involved in several cellular functions, such as cell growth, survival, and proliferation [19]. In human fetal lung fibroblasts, overexpression of HDAC4 delayed cellular senescence by stabilizing SIRT1 [20]. Di Giorgio et al. showed that HDAC4 is reduced during senescence, and it monitors the acetylation levels of histone H3 on lysine 27 (H3K27ac) at specific enhancers that supervise the senescent transcriptome [21, 22]. Despite these findings, the multifaceted regulatory roles of HDAC4 in skin aging remain largely elusive.

Here, we elucidate the candidate targets and potential mechanisms of HDAC4 associated with skin aging by an integrated analysis of RNA sequencing data generated from HDAC4-modulated dermal fibroblasts with the previous mRNA expression profiles relevant to cellular senescence. Our results demonstrate that DNA damage-inducible transcript 4 (DDIT4) is significantly regulated by HDAC4, and that HDAC4 could delay senescence by regulating DDIT4 expression.

## RESULTS

### HDAC4 expression is reduced in H_2_O_2_- and UV-induced senescent fibroblasts

**Figure 1:**
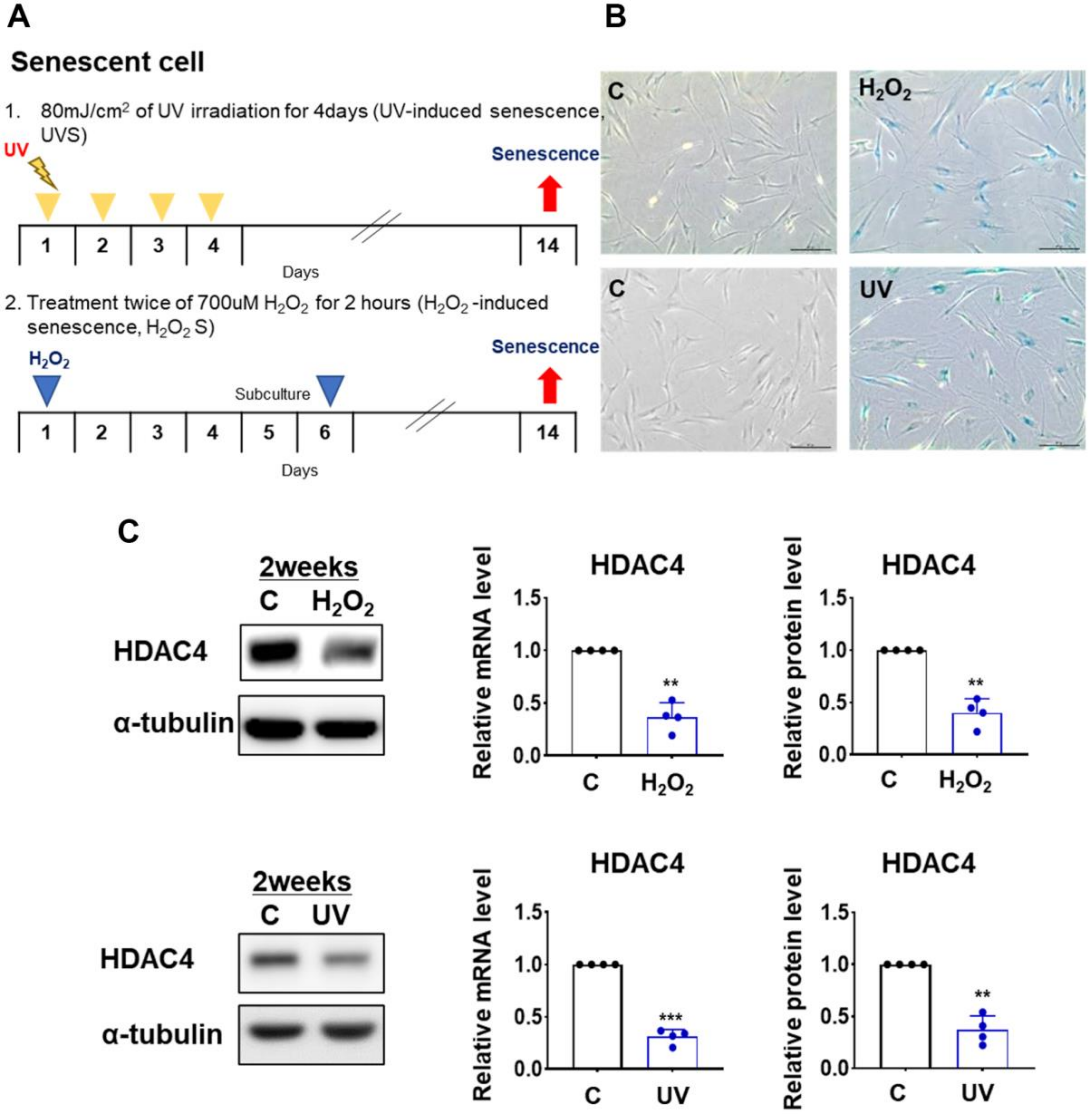
**HDAC4 is decreased in H_2_O_2_- and UV-induced cellular senescence. ** (**A**) A scheme of H_2_O_2_- and UV-induced cellular senescence using human dermal fibroblasts (**B**) Senescence-associated beta-galactosidase assay was performed to detect senescent cells for control (left) and senescence-induced (right) cells (n = 4 each). Original magnification: x100. (**C**) HDAC4 expression analyzed by quantitative RT-PCR or western blotting. Alpha-tubulin was used as loading control. Data represent the mean ± SE (n = 4, ** P <0.01, *** P <0.001). C: control.

To understand the role of HDAC4 in the senescence process, we first examined changes in HDAC4 expression in two different cellular senescence models: H_2_O_2_-induced senescence (H_2_O_2_S) and UV-induced senescence (UVS) (Figure 1A). The induction of H_2_O_2_S and UVS models was confirmed using SA-β-gal staining (Figure 1B, upper and lower panels, respectively). The expression of HDAC4 mRNA and protein was significantly decreased in both H_2_O_2_S and UVS models (Figure 1C). This implies that reduced HDAC4 expression is a consistent finding under senescence conditions.

### Integrative transcriptome analysis identifies DDIT4 as an HDAC4 target in senescence

We performed RNA sequencing analysis of primary HDFs transfected with HDAC4 siRNA or HDAC4 overexpression vector to identify HDAC4 target candidate genes. By comparing transcriptomes from HDAC4-overexpressed and-knockdown fibroblasts, we identified 339 Differentially expressed genes (DEGs) (208 induced and 131 suppressed by HDAC4) as potential targets of HDAC4 (Supplementary Figure 1A and Supplementary Table 1; see Materials and Methods for details). The genes induced by HDAC4 (208 genes) were mainly involved in the processes related to cell cycle (cell division, DNA replication, and DNA repair) or epigenetic regulation (gene silencing and histone exchange; Supplementary Figure 1B). However, the 131 genes suppressed by HDAC4 were mostly associated with processes related to protein secretion (extracellular vesicle, cytokine production, and interleukin-1 secretion) or lysosomal lipid metabolism (endocytosis, lipid metabolic process, and lysosome; Supplementary Figure 1C).

**Figure 2:**
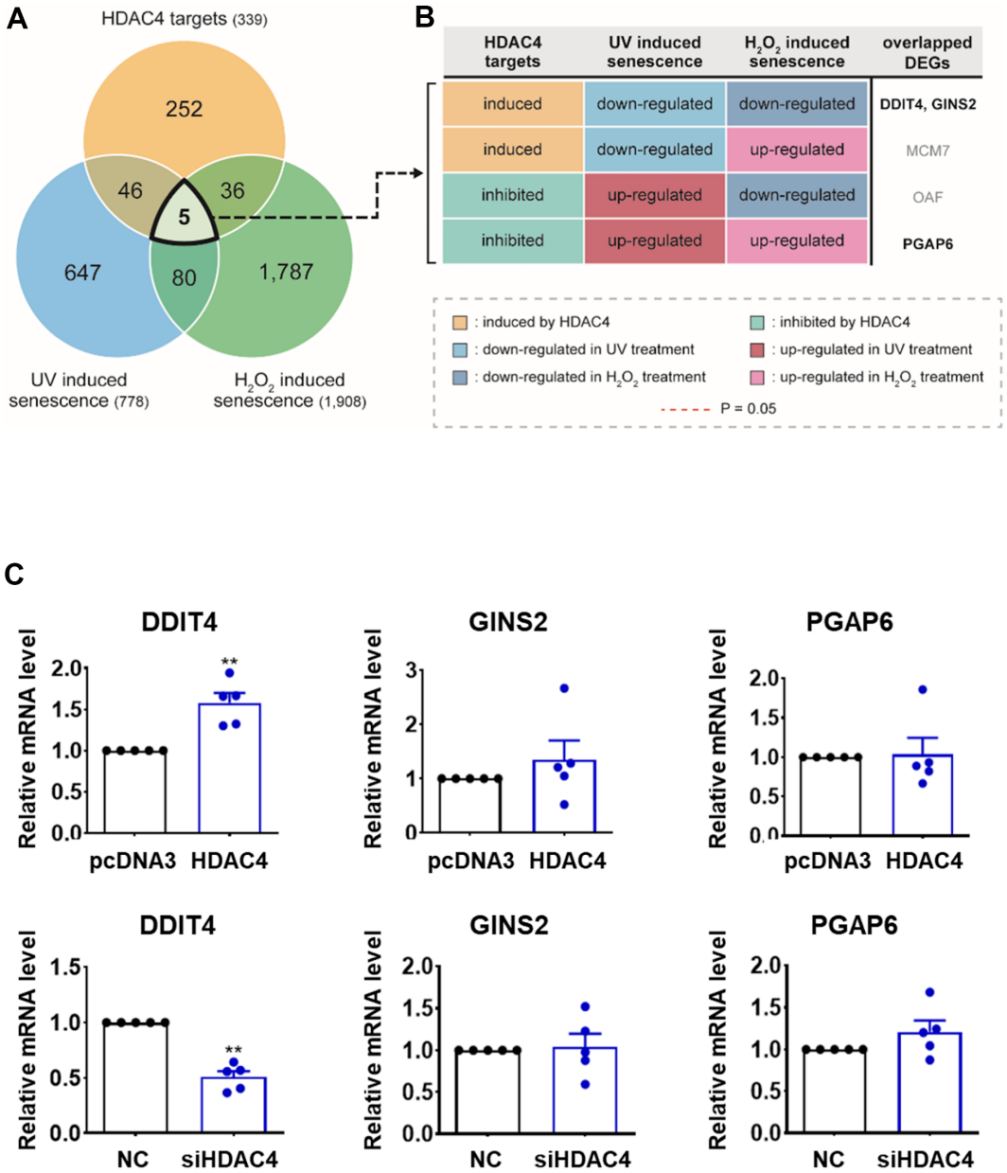
**Integrative transcriptome analysis identifies DDIT4 as a candidate target. ** (**A**) Venn diagram showing the relationships among DEGs identified from the three comparisons: HDAC4 overexpression versus knockdown (HDAC4 targets); UV-induced senescent versus control HDFs (UV-induced senescence); and H_2_O_2_-induced senescent versus control HDFs (H_2_O_2_-induced senescence). Numbers in parentheses, numbers of DEGs. (**B**) Differential expression patterns of the five overlapping DEGs in the three comparisons (see legend). (**C**) The mRNA levels of DDIT4, GINS2, and PGAP6 analyzed by RT-PCR (n=5). Data represent the mean ± SE (n = 5, * P < 0.05, ** P <0.01, *** P <0.001). NC: scrambled control.

To sort out more reliable targets of HDAC4 involved in the regulation of skin aging, we compared our DEGs with those identified from the two previously reported transcriptome datasets, which were generated from UV-[23] and H_2_O_2_-induced senescent HDFs [24]. Among the 778 DEGs from UV-induced senescence [23] and 1,908 DEGs from H_2_O_2_-induced senescence [24] (Figure 2A), 51 and 41 genes were shared with our DEGs, respectively. Only five genes (DDIT4, GINS2, MCM7, OAF, and PGAP6) overlapped among the DEGs from previous senescence models and our HDAC4-modulated model. Among them, given the reduced HDAC4 level in senescent cells, DDIT4 and GINS2 induced by HDAC4 were downregulated, and PGAP6 suppressed by HDAC4 was up-regulated consistently in both H_2_O_2_- and UV-induced senescence models (Figure 2B). In contrast, MCM7 and OAF showed inconsistent changes in the HDAC4-modulated and senescence models. Therefore, the validity of the three genes with consistent changes as HDAC4 targets was tested using quantitative RT-PCR. Among the three genes, only DDIT4 showed significant expression changes upon HDAC4 overexpression or knockdown (Figure 2C). Taken together, we finally selected DDIT4 as a potential target of HDAC4 involved in HDAC4-dependent epigenetic regulation of skin aging.

### Reduced DDIT4 expression and senescent phenotype were restored by HDAC4 overexpression in senescent fibroblasts

**Figure 3:**
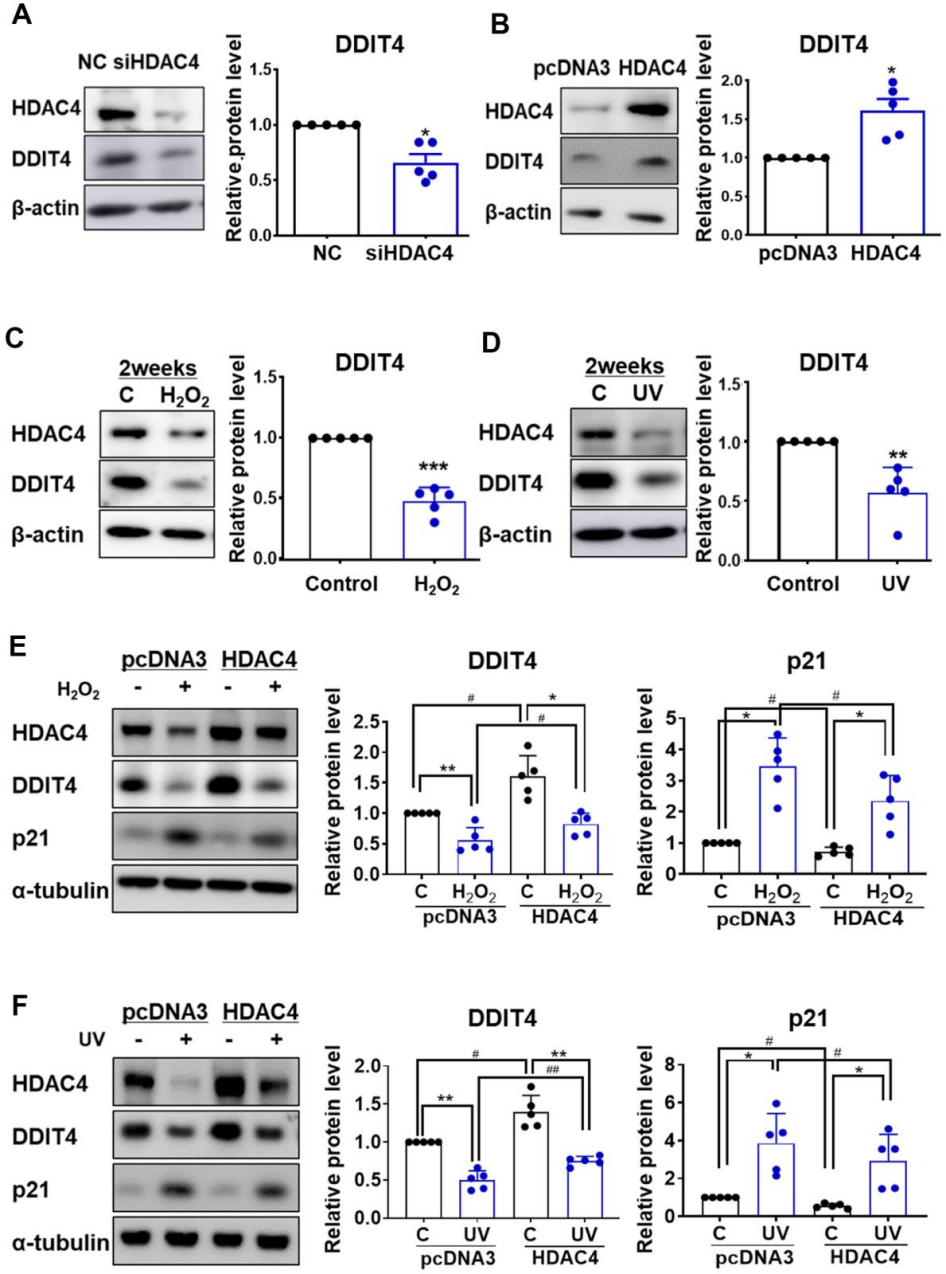
**Senescence-induced reduction of DDIT4 is restored by HDAC4 overexpression. ** (**A**) Human dermal fibroblasts were transfected with negative control scrambled siRNA (NC) or HDAC4 siRNA. (**B**) For overexpression of HDAC4, cells were treated with pcDNA3 or HDAC4 plasmid DNA and the medium was replaced after 6 h. (**A**–**D**) RT-PCR and western blotting were performed to determine the expression of DDIT4 and HDAC4. Data are presented as mean ± SE (n = 5, * P <0.05, ** P <0.01, *** P <0.001 vs. NC, pcDNA3, or Control). (**E**, **F**) After senescence was induced, cells were transfected with pcDNA3 or HDAC4 overexpression vector. DDIT4, p21, and HDAC4 protein expression were analyzed by western blotting. Beta-actin and alpha-tubulin served as a loading control. Data are presented as mean ± SE using Student’s t-test (n = 5, * P <0.05, ** P <0.01, *** P <0.001 control vs H_2_O_2_; # P <0.05, ## P <0.01, pcDNA3 vs HDAC4). C: control.

It has been reported that DDIT4 is associated with senescence [25–27], and our previous studies demonstrated that HDAC4 might be a key regulator of skin aging [17, 18]. However, little is known about the association between HDAC4 and DDIT4 during cellular senescence. We first examined whether HDAC4 regulates DDIT4. In accordance with the mRNA expression analysis, HDAC4 knockdown significantly decreased the expression of DDIT4 protein, while HDAC4 overexpression significantly increased it (Figure 3A, 3B). In contrast, overexpression or knockdown of DDIT4 did not change the expression of HDAC4, indicating that DDIT4 is a downstream target of HDAC4 (Supplementary Figure 2).

Replicative senescent HDFs showed a decreased expression of HDAC4 and DDIT4 protein (Supplementary Figure 3). Next, we analyzed whether DDIT4 expression is changed in H_2_O_2_- or UV-induced senescent fibroblasts. DDIT4 expression was significantly decreased in both H_2_O_2_- and UV-induced senescent HDFs (Figure 3C, 3D). This reduction in DDIT4 expression in both models was prevented by HDAC4 overexpression. The increase in p21 expression under the senescent condition was also reduced by HDAC4 overexpression (Figure 3E, 3F). These results suggest that the decrease in HDAC4 levels during senescence may cause a reduction of DDIT4.

**Figure 4:**
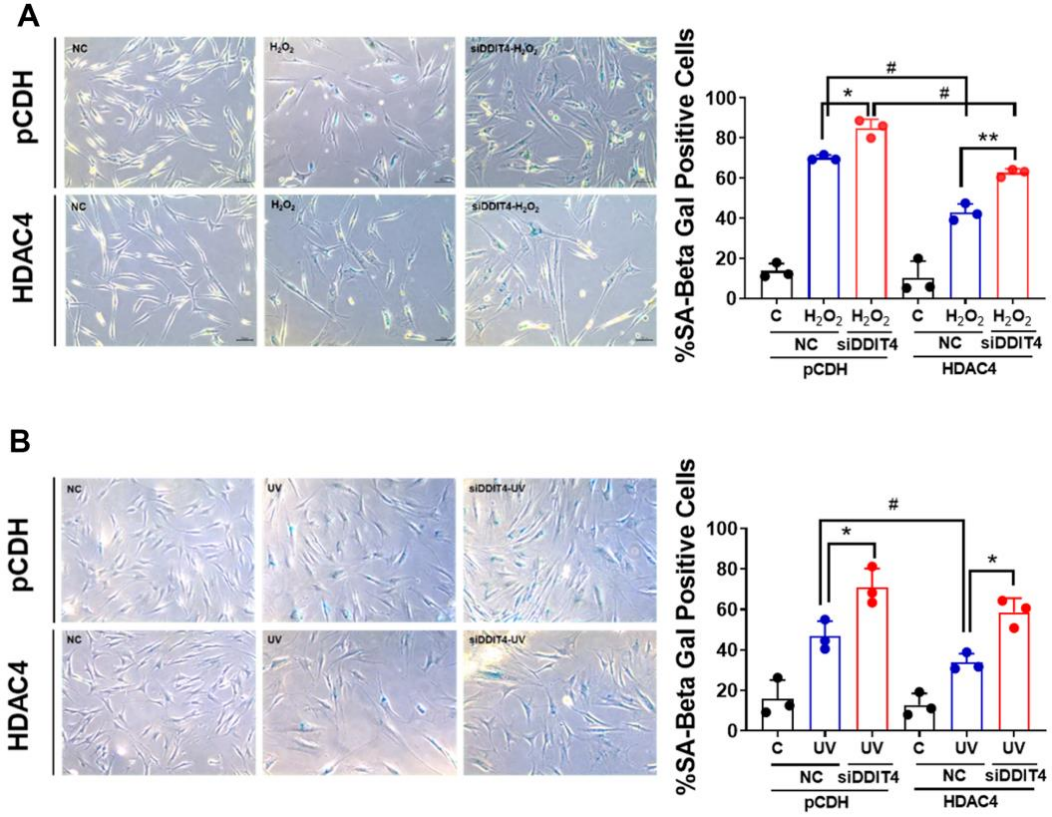
**Inhibition of DDIT4 exacerbates cellular senescence.** (**A**, **B**) Primary dermal fibroblasts were subject to senescence induction with H_2_O_2_ or UV. After senescence induction, cells were transfected with negative siRNA or DDIT4 siRNA for 48 h. Beta-galactosidase assay was performed to detect senescent cells. Original magnification: x100. Data represent the mean ± SE (n=3, * P <0.05, ** P <0.01 vs siDDIT4; # P <0.05, ## P <0.01, pCDH vs HDAC4). C: control, NC: scrambled control.

To determine whether DDIT4 is involved in senescence, we examined the proportion of SA-β-gal-positive cells in senescent cells transfected with HDAC4 overexpression vector and/or DDIT4 siRNA. The siRNA-mediated knockdown of DDIT4 reduced DDIT4 by approximately 40% (Supplementary Figure 4). Overexpression of HDAC4 significantly reduced the SA-β-gal–positive cell population in both cellular senescence models. However, knockdown of DDIT4 increased SA-β-gal-positive cells in both control senescent cells and HDAC4-overexpressed cells (Figure 4A, 4B). These results indicate that DDIT4 may play a protective role in cellular senescence.

### DDIT4 suppressed the SASP components and changes in aging-related genes

**Figure 5:**
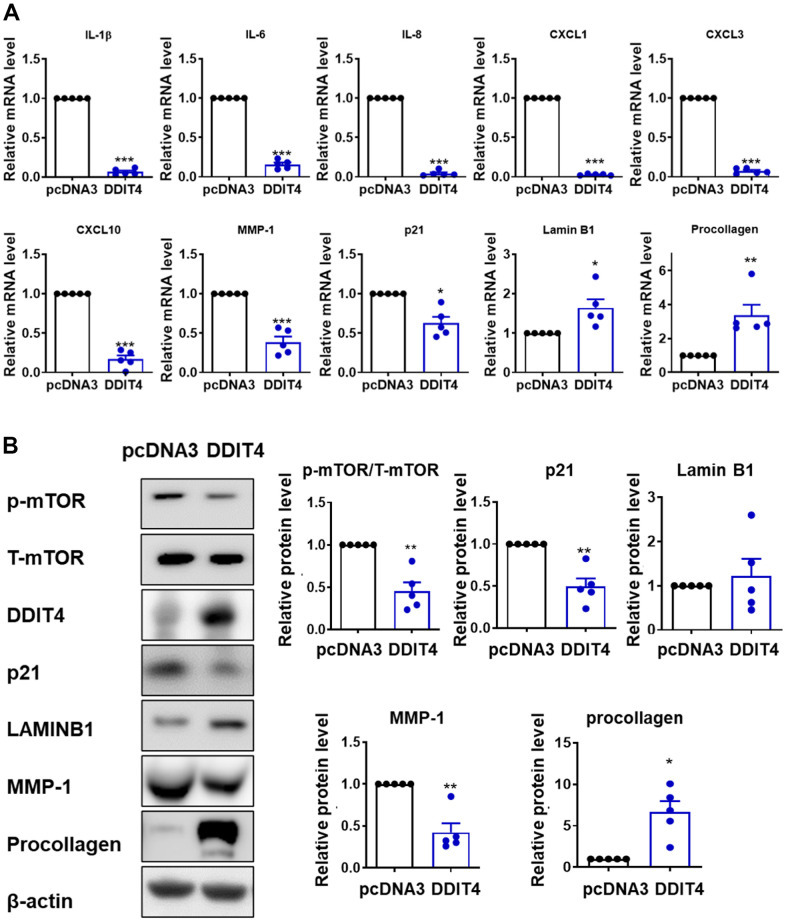
**DDIT4 regulates the expression of SASP and age-associated genes.** (**A**) The mRNA levels of senescence-associated secretory phenotype (SASP) and aging-related genes were determined by quantitative real-time PCR. (**B**) After DDIT4 overexpression, the protein levels of aging-related genes were examined using western blot analysis. Beta-actin served as a loading control. Data represent the mean ± SE (n = 5, ** P <0.01, *** P <0.001).

To investigate whether DDIT4 regulates the senescence-associated secretory phenotype (SASP) or aging-related genes, HDFs were transfected with a DDIT4 overexpression vector. Overexpression of DDIT4 significantly decreased the mRNA levels of SASP components, including *IL-1β, IL-6, IL-8, CXCL1, CXCL3, CXCL10,* and *MMP-1,* and that of the aging-related gene *p21*. The mRNA levels of the other aging-related genes, *lamin B1* and *procollagen type 1,* which are known to be reduced during aging, were significantly increased by DDIT4 overexpression (Figure 5A). Consistent with the mRNA levels, the protein levels of p21 and MMP-1 were significantly downregulated by DDIT4 overexpression, while that of type 1 procollagen was significantly increased. Moreover, DDIT4 overexpression inhibited the increased SASP components in senescent HDFs induced by H_2_O_2_ or UV (Supplementary Figure 5). Phosphorylation of mTOR, one of the key targets of aging, was reduced by DDIT4 overexpression. The protein level of lamin B1 tended to increase, but the difference was not statistically significant (Figure 5B). These results imply that DDIT4 may counteract aging by repressing SASPs or preventing changes in aging-associated genes.

### DDIT4 expression was reduced in aged human skin *in vivo*

**Figure 6:**
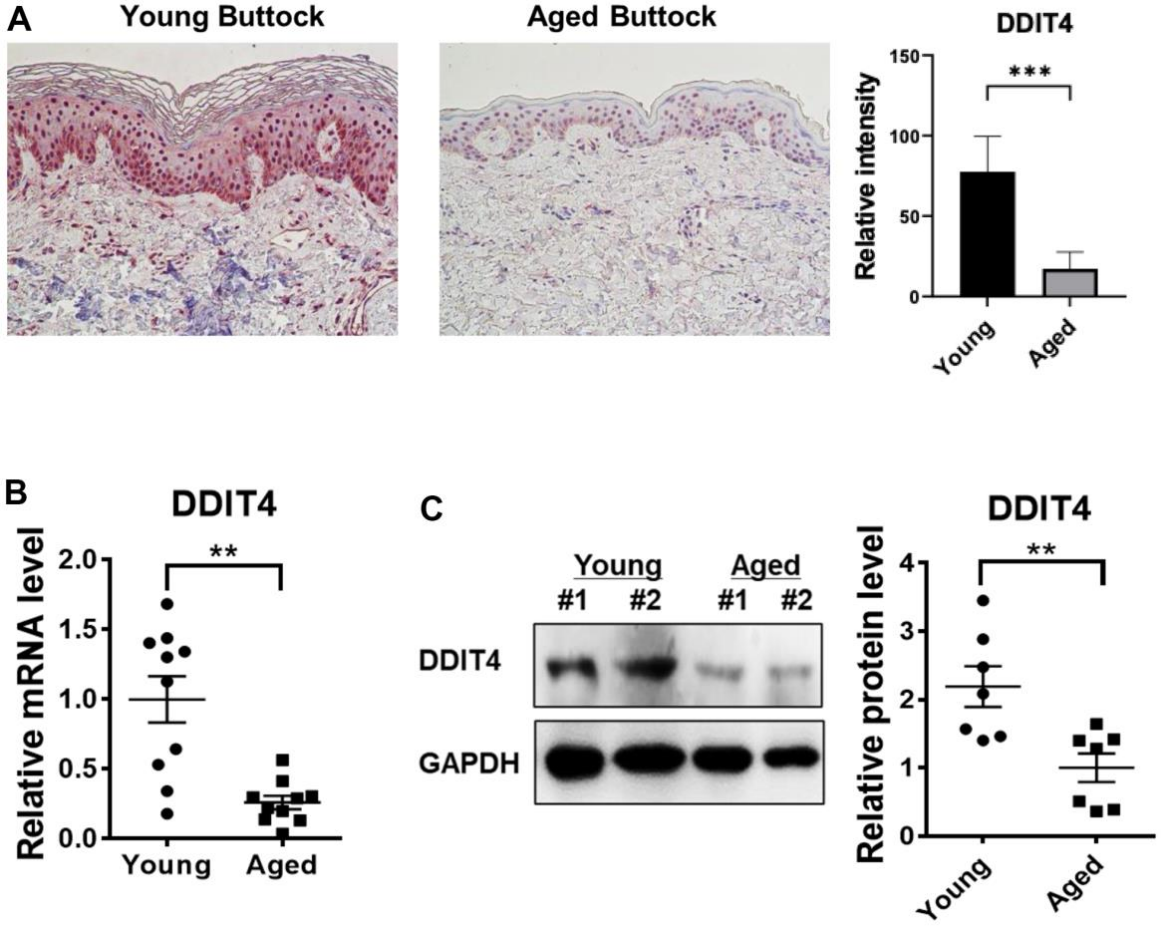
**DDIT4 expression level was decreased in aged human skin *in vivo*.** (**A**) Buttock skin samples of young and elderly subjects were obtained by punch biopsy. DDIT4 expression in the skin of individuals was examined by immunohistochemistry and quantified. Immunohistochemistry analysis was performed using paraffin-embedded sections. Original magnification: x200. Data represent the mean ± SE (n=6, *** P <0.001 vs Young) (**B**, **C**) Quantification of relative mRNA (n = 10) and protein (n = 7) levels of DDIT4 was performed using qPCR and western blotting. GAPDH served as a loading control. Data represent the mean ± SE (** P <0.01 vs Young).

Finally, we validated DDIT4 expression in the human skin of young and elderly subjects *in vivo*. Immunohistochemistry of young skin showed that DDIT4 was strongly expressed in both the epidermis and dermis. DDIT4 was present mostly in the nucleus and partially in the cytoplasm, particularly in the basal epidermal layer. Sun-protected buttock skin from elderly subjects showed remarkably decreased DDIT4 expression compared to that in young individuals (Figure 6A).

Similarly, DDIT4 mRNA and protein levels in aged buttock skin, when determined by quantitative PCR and western blotting, respectively, were significantly lower than those in young buttock skin (Figure 6B, 6C). These data showed that DDIT4 expression was also significantly reduced in aged human skin *in vivo* as in senescent fibroblasts, suggesting that reduced DDIT4 may play a crucial role in inducing epigenetic skin aging.

## DISCUSSION

Several studies have reported the potential of epigenetic regulation in delaying senescence [4, 7, 9, 10, 20, 28–31]. Our previous studies showed that UV irradiation decreased HDAC4 expression in primary HDFs, and HDAC4 expression was reduced in aged skin *in vivo* [17, 18]. These results suggest that HDAC4 may play an important role in skin aging. However, there is a paucity of research on how HDAC4 causes skin aging.

By integrating our RNA-Seq data and previously reported transcriptome datasets from UV- and H_2_O_2_-induced senescence models, we identified DDIT4 as a promising candidate target of HDAC4 involved in HDAC4-dependent epigenetic regulation of skin aging. DDIT4 mRNA and protein levels were significantly down- and up-regulated consistently upon HDAC4 knockdown and overexpression, respectively. As a stress-responsive gene, DDIT4 expression also changes during development and DNA damage responses [32, 33]. It acts as a mammalian target of rapamycin complex 1 (mTORC1) inhibitor and plays a dual role as a pro-survival or pro-apoptotic factor depending on the cell type and cellular context [27, 34]. DDIT4 regulates cell growth, oxidative stress, autophagy, mitochondrial function, and apoptosis [35–37]. Although DDIT4 function has been investigated extensively in the fields of cancer and autophagy [38, 39], little is known about its role in skin aging.

We found that DDIT4 expression was markedly reduced in aged skin *in vivo*, in replicative senescent HDFs, and in senescent fibroblasts under repeated H_2_O_2_ treatment or UV irradiation. HDAC4 expression was positively correlated with DDIT4, and also significantly decreased in aged skin *in vivo* (Supplementary Figure 6). Consistent with our results, DDIT4 was shown to be decreased in senescent mesenchymal stromal cells [25], senescent human mesenchymal stem cells [26], aged human articular cartilage [27], and HaCaT immortalized keratinocytes after apoptosis-inducing UV irradiation [40]. Our data indicate that the knockdown of DDIT4 prevented the HDAC4-induced reduction of SA-β-gal in senescent cells. Moreover, DDIT4 overexpression could restore the senescence-associated alterations of SASP components such as IL-β, IL-6, IL-8, MMP-1, and CXCLs, as well as aging-related genes, suggesting that DDIT4 may contribute to the skin aging process by regulating senescence-associated microenvironments. Consistently, Li et al. reported that DDIT4 overexpression suppressed γ-irradiation-induced SASP (IL-6 and IL-8), SA-β-gal positive cells, the expression of p21 and mTOR in human osteoblasts [41]. Therefore, DDIT4, known as an important negative regulator of mTOR, may play an essential role in suppressing cell senescence by inhibiting mTOR activity or p21. Interestingly, there is a strong expression of DDIT4 in the epidermis of young individuals, and DDIT4 may function as a regulator of re-epithelialization [42] and stem cell function [43].

DDIT4 expression is mediated by different transcription factors, such as p53, NF-κB, p63, and HIF-1 [39]. Increased DDIT4 expression by HDAC4 overexpression may be due to transcriptional activation of DDIT4 by these transcription factors. A study reported that HDAC4 increases DDIT4 transcription by modulating HIF-1 activation in ataxia-telangiectasia [44], but further investigation is needed to understand how HDAC4 regulates DDIT4 expression.

In conclusion, we demonstrated that HDAC4 was decreased in H_2_O_2_- and UV-induced cellular senescence models, and the overexpression of HDAC4 reduced senescence phenotypes and markers. DDIT4, a candidate target regulated by both HDAC4 modulation and cellular senescence, could reverse the expression of genes associated with aging, such as SASP, and SA-β-gal positive cells. These results provide novel insights into the regulatory role of the HDAC4-DDIT4 pathway in epigenetic skin aging.

## MATERIALS AND METHODS

### Reagents

Antibodies against HDAC4 and p21 were purchased from Cell Signaling Technology (Beverly, MA, USA). Antibodies against beta-actin, lamin B1, and alpha-tubulin were purchased from Thermo Fisher Scientific (Waltham, MA, USA). Antibodies against DDIT4 were purchased from Proteintech (Chicago, IL, USA). Antibodies against MMP-1 were obtained from Lab Frontier, Ltd. (Seoul, Republic of Korea), and an antibody for procollagen type 1 was purchased from Developmental Studies Hybridoma Bank (Iowa City, IA, USA).

### Cell culture and cellular senescence models

Primary human skin fibroblasts were obtained from healthy donors. Cells were cultured in Dulbecco’s modified Eagle’s medium (DMEM, Welgene, Daegu, South Korea) containing 10 % fetal bovine serum (FBS, Gibco, Rockville, MD, USA) and 1 % penicillin (Gibco) at 37° C in a humidified incubator with 5% CO_2_. For UV irradiation, cells were irradiated with UV four times at 80 mJ/cm^2^ in phosphate-buffered saline (PBS). UV irradiation was performed using a Philips TL20W/12RS SLV/25 sun lamp (Eindhoven, Netherlands) with an emission spectrum between 290 nm and 380 nm. A filter was used to block UVC wavelengths below 290 nm. The intensity of UV irradiation was measured at a broad-band UVB spectrum using a Model 585100 UV meter (Herbert Waldmann GmbH & Co.KG, Villingen-Schwenningen, Germany). For oxidative stress-induced senescence, cells were treated twice with 700 uM hydrogen peroxide for two hours.

### Human skin samples

Skin samples were obtained from elderly (mean age 76.8 y; range 70–83 y) and young (mean age 31.5 y; range 20–43 y) subjects. Korean volunteers with no current or prior skin diseases were recruited. This study was conducted in accordance with the principles of the Declaration of Helsinki. All experimental procedures were approved by the Institutional Review Board of Seoul National University Hospital, and written informed consent was obtained from each subject (IRB No. 1410-133-621).

### Senescence-associated β-gal staining

SA-β-gal was assayed using a kit by Cell Signaling Technology. Cells were fixed for 10 min in a fixative solution and washed twice with PBS. Cells were incubated in β-galactosidase staining solution at 37° C until β-gal staining became visible.

### Replicative senescence

Replicative senescence was induced by continuous passage of cells in culture. Low passage HDFs (passage 5-6) were thawed and cultured in DMEM supplemented with 10% FBS, and 1% penicillin at 37° C in a humidified incubator with 5% CO_2_. Cells were split when they reached 80-90% confluence, and continued to grow until replicative senescence (passage 40-45).

### siRNA transfection

The expressions of DDIT4 and HDAC4 were knocked down using small interfering RNAs (siRNAs). Negative control siRNA (AccuTarget™ negative control siRNA), and siRNAs targeting HDAC4 and DDIT4 were purchased from Bioneer (Daejeon, Republic of Korea). Skin fibroblasts were transfected with control scrambled siRNA or siRNAs targeting HDAC4 or DDIT4 using jetPRIME^®^ (Polyplus-transfection^®^ S, IIIkirch, France), according to the manufacturer’s instructions.

### Plasmid constructs, transfection, and lentivirus production

The mammalian expression plasmids for HDAC4 (plasmid #30485) and DDIT4 (RC202847) were purchased from Addgene (Cambridge, MA, USA) and OriGene (Rockville, MD, USA), respectively. Each plasmid was transiently transfected into human dermal fibroblasts using jetPRIME^®^ by following the manufacturer's instructions. HDAC4 lentiviral particles were produced according to the manufacturer’s protocol (System Biosciences, Mountain View, CA, USA) using pCDH-CMV-MCS-EF1-Puro plasmid or pCDH-CMV-HDAC4-EF1-Puro plasmid. The pCDH-CMV-HDAC4-EF1-Puro plasmid was produced by amplifying the HDAC4 cDNA from pcDNA3.1-HDAC4 plasmid by PCR and subcloning it into pCDH-CMV-MCS-EF1-Puro plasmid. Briefly, HEK-293TN cells were transfected with plasmids containing pGag-pol and pVSV-G using Lipofectamine 3000 (Invitrogen, Carlsbad, CA, USA). The viral supernatant was collected and transduced into the human dermal fibroblasts. Infected cells were selected using 5 μg/mL puromycin (SNUH-IBC-1903-007-004).

### Transcriptome analysis

RNA sequencing was conducted for transcriptome analysis. Total RNA was isolated from primary human dermal fibroblasts (HDFs) transfected with HDAC4 siRNA or HDAC4 overexpression vector and their controls (control siRNA and control pcDNA vector) using Trizol (Invitrogen, USA). Isolated RNA was fragmented using TruSeq Stranded Total RNA Sample Prep Kit (Illumina, San Diego, USA) according to the manufacturer’s protocol, and sequencing libraries were then generated based on reverse transcription of the fragmented RNA using Superscript II reverse transcriptase (Invitrogen, USA), followed by adaptor ligation. Libraries were sequenced using the HiSeq 2500 system. The raw data were deposited in the Gene Expression Omnibus (GEO) with accession ID GSE181329. The raw data were analyzed by the following steps: 1) adaptor trimming using Cutadapt (ver 3.4), 2) read alignment to the reference genome (GRCh38) using the STAR aligner (ver 2.7.9a) [45], and 3) gene feature (GRCh38.104.gtf) quantification of fragments per kilobase of transcript per million fragments (FPKM) using Cufflinks (version 2.2.1) [46], and 4) normalization of FPKM values across the samples using the quantile normalization method [47]. Moreover, we obtained transcriptome datasets generated from UV- (GSE60795) and H_2_O_2_-induced senescent HDFs (GSE116761) from the GEO database. Raw data from Affymetrix array platforms were normalized using the robust multiarray average (RMA) method in the oligo R package (ver 1.57.0) and summarized in the probeset intensities as mRNA expression levels. Raw data (probe intensities as mRNA expression levels) from Agilent array platforms were normalized using the quantile normalization method [47]. Detailed information for the samples used in the previous transcriptomic studies [23, 24] is summarized in Supplementary Table 2.

### Identification of differentially expressed genes (DEGs)

HDFs were obtained from three different subjects, and HDAC4 overexpression and knockdown were performed for the HDFs of each subject. For each gene in the HDF of the subject, we first computed its expression level ratios under HDAC4 overexpression (OE_ratio_ = HDAC4 overexpression/vector control) and knockdown (KD_ratio_ = HDAC4 knockdown/siRNA control) conditions. For each gene, we then compared the OE_ratio_ and KD_ratio_ to obtain a T-statistic value using a paired Student’s *t*-test and a log_2_-fold-change value. Next, we constructed empirical null distributions of T-statistic values and log_2_-fold-changes by randomly permuting the samples and computing random OE_ratio_ and KD_ratio_ values as well as random T-statistic values and log_2_-fold-changes (1000 random permutations). Based on the two empirical null distributions, adjusted P-values for the observed T-statistic value (Pt) and log_2_-fold-change value (Pf) were computed for each gene. After manual inspection of differences between OE_ratio_ and KD_ratio_ values in the HDFs of the three subjects, the genes with Pt ≤ 0.05, and Pf ≤ 0.10 were selected as DEGs (Supplementary Table 1). For the previous microarray data, the unpaired Student’s *t*-test method was employed. For each comparison (UV-induced versus control HDFs, and H_2_O_2_-induced versus control HDFs), the same method (Pt and Pf) and criteria (Pt and Pf cutoffs) mentioned above were used to identify the DEGs (Supplementary Tables 3, 4). Finally, to identify cellular processes and functions represented by the DEGs, we performed enrichment analysis of gene ontology biological processes (GOBPs) and molecular functions (GOMFs) using DAVID software [48] and then selected the GO terms with a p-value < 0.05.

### Western blotting

Cells were lysed with lysis buffer (RIPA lysis buffer, Merck KGaA, Darmstadt, Germany) containing a protease inhibitor mixture (Roche Applied Science, Penzberg, Germany) and phosphatase inhibitor mixture (Sigma-Aldrich, MO, USA). Lysates were collected after centrifugation at 12,000 rpm for 15 min at 4° C. Protein concentrations were determined using the BCA assay (Sigma-Aldrich). Protein extracts (40 μg) were separated in 8 %–12% SDS-polyacrylamide gels and then transferred onto nitrocellulose membranes (GE Healthcare Life Sciences, Buckinghamshire, UK). After blocking for 30 min in 5% skim milk, the membranes were incubated with the appropriate primary antibodies and horseradish peroxidase-conjugated secondary antibodies. Western blots were visualized using an enhanced chemiluminescence detection system (Thermo Fisher Scientific). Signal intensity was measured using ImageJ software (National Institutes of Health, Bethesda, MD, USA).

### Reverse transcription-quantitative polymerase chain reaction

Total RNA was isolated using RNAiso Plus reagent (Takara Bio Inc., Shiga, Japan) according to the manufacturer’s protocol. First, 1 μg of total RNA was converted to cDNA using the RevertAid First Strand cDNA Synthesis Kit (Thermo Fisher Scientific). Quantitation of HDAC4, DDIT4, GINS2, PGAP6, SASP, p21, lamin B1, procollagen, and endogenous reference 36B4 cDNA was then performed on a 7500 Real-time PCR system (Applied Biosystems; Thermo Fisher Scientific, Inc.) using SYBR Premix Ex Taq (Takara Bio Inc.) by following the manufacturer’s instructions. Thereafter, the 2^-∆∆Ct^ method was used to calculate the fold-change in the expression levels of the target genes, which were normalized to those of 36B4.

### Immunohistochemistry staining

Human skin tissues were fixed in 10% formalin and embedded in paraffin wax. Skin samples were cut into 4 μm sections and dewaxed in xylene, rehydrated in graded alcohol, and then washed with distilled water. Endogenous peroxidase activity was quenched with 3% hydrogen peroxide for 10 min. The sections were blocked for 30 min (UltraVision Protein Block, Thermo Fisher Scientific), stained with an anti-DDIT4 rabbit primary antibody for 18 h at 4° C, and incubated with streptavidin in horseradish peroxidase conjugate for 15 min (Golden Bridge International) in a humidified chamber. The sections were developed using AEC (3-amino-9-ethylcarbazole, Golden Bridge International) for 5 min and mounted with Faramount medium (DAKO, Carpinteria, CA, USA). The cell nuclei were counterstained with Mayer’s hematoxylin.

### Statistical analysis

Statistical analyses were performed using two-tailed paired *t*-tests or two-tailed Student’s *t*-tests. Results with P-values less than 0.05 were considered statistically significant. Data are presented as mean ± standard error.

### Institutional review board statement

The study was conducted according to the guidelines of the Declaration of Helsinki, and approved by the Institutional Review Board of Seoul National University Hospital, and written informed consent was obtained from each subject (IRB No. 1410-133-621).

### Informed consent statement

Informed consent was obtained from all subjects involved in the study.

## Supplementary Material

Supplementary Figures

Supplementary Table 1

Supplementary Table 2

Supplementary Table 3

Supplementary Table 4
