# Supplementary material for: Histone deacetylase 4 reverses cellular senescence via DDIT4 in dermal fibroblasts
Source: Aging (Albany NY). 2022 Jun 9;14(11):4653–72. doi: 10.18632/aging.204118 (PMC9217707; doi:10.18632/aging.204118)
Supplement: Supplementary Figures [file aging-14-204118-s001.pdf]

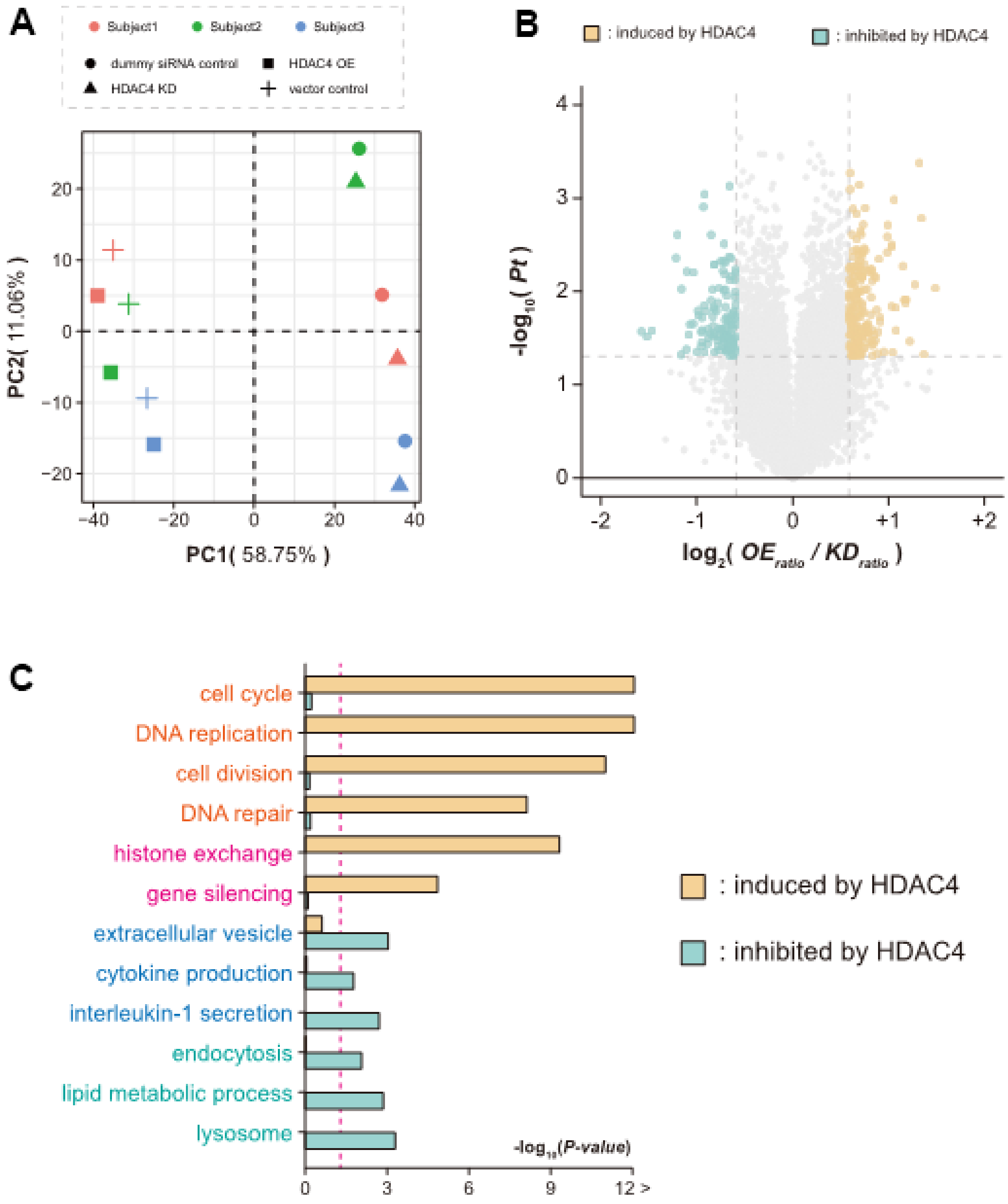

Supplementary Figure 1. Transcriptome analysis of HDAC4 modulated human dermal fibroblasts (HDFs). (**A**) Principle component analysis (PCA) plot showing the distribution of each sample. (**B**) Volcano plots displaying differentially expressed genes (DEGs) between OE_{ratio} (HDAC4 overexpression/vector control) and KD_{ratio} (HDAC4 knockdown/siRNA control) in HDFs. Green and yellow dots represent DEGs that are inhibited or induced by HDAC4, respectively. (**C**) Bar plot showing enrichment of cellular processes by DEGs. Red line represents the *P*-value cutoff (*P*=0.05).

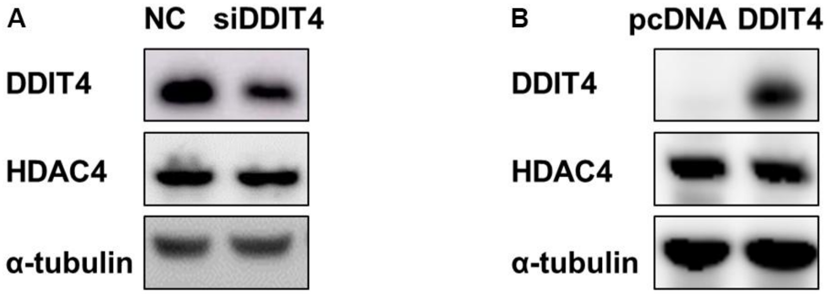

Supplementary Figure 2. DDIT4 does not change the expression of HDAC4. (**A**) Human dermal fibroblasts were transfected with negative control scrambled siRNA (NC) or DDIT4 siRNA (siDDIT4) (n=3). (**B**) For overexpression of DDIT4, cells were treated with pcDNA3 or DDIT4 plasmid DNA and the medium was replaced after 6 h (n=3). NC: scrambled control.

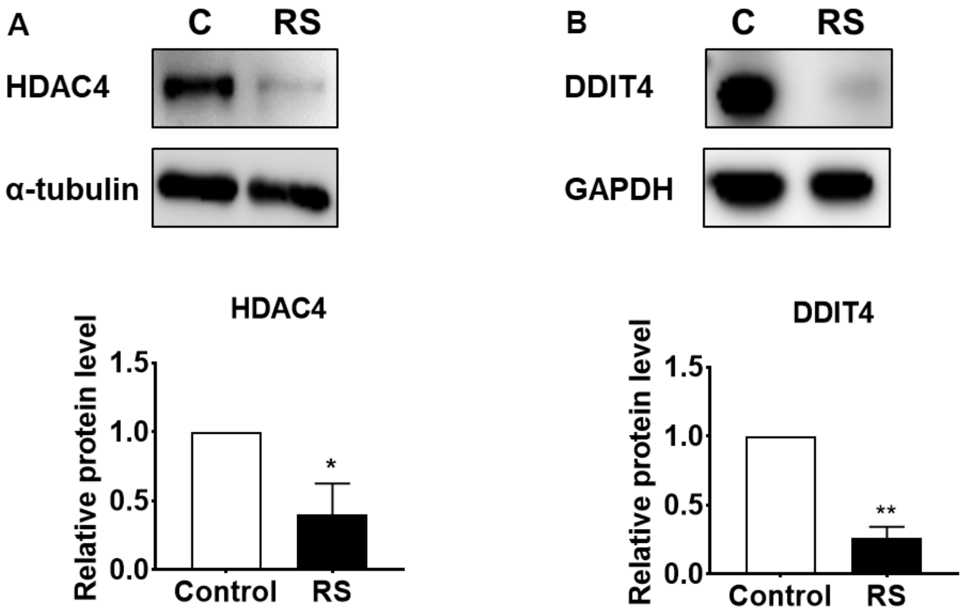

Supplementary Figure 3. Replicative senescent human dermal fibroblasts (HDFs) showed a decreased expression of HDAC4 and DDIT4 protein. (**A**, **B**) Low passage HDFs (passage 5-6) were continually cultured until replicative senescence (passage 40-45). The protein levels of HDAC4 and DDIT4 were analyzed by western blotting (n=4). Alpha-tubulin was used as a loading control. Data represent the mean ± SE (n = 4, * P <0.05, ** P <0.01). Control: low-passage HDFs, RS: replicative senescent HDFs.

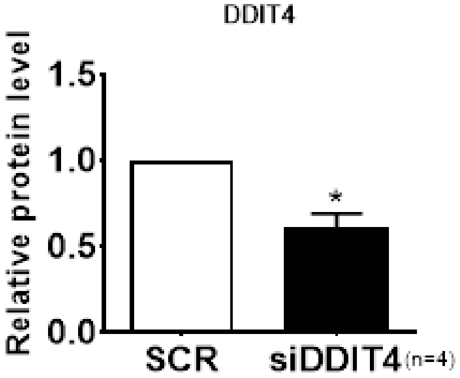

Supplementary Figure 4. The protein level of DDIT4 silenced by siDDIT4. Human dermal fibroblasts were transfected with negative control scrambled siRNA (NC) or DDIT4 siRNA (siDDIT4) (n=4). The protein level of DDIT4 was analyzed by western blotting. Data represent the mean ± SE (*P<0.05).

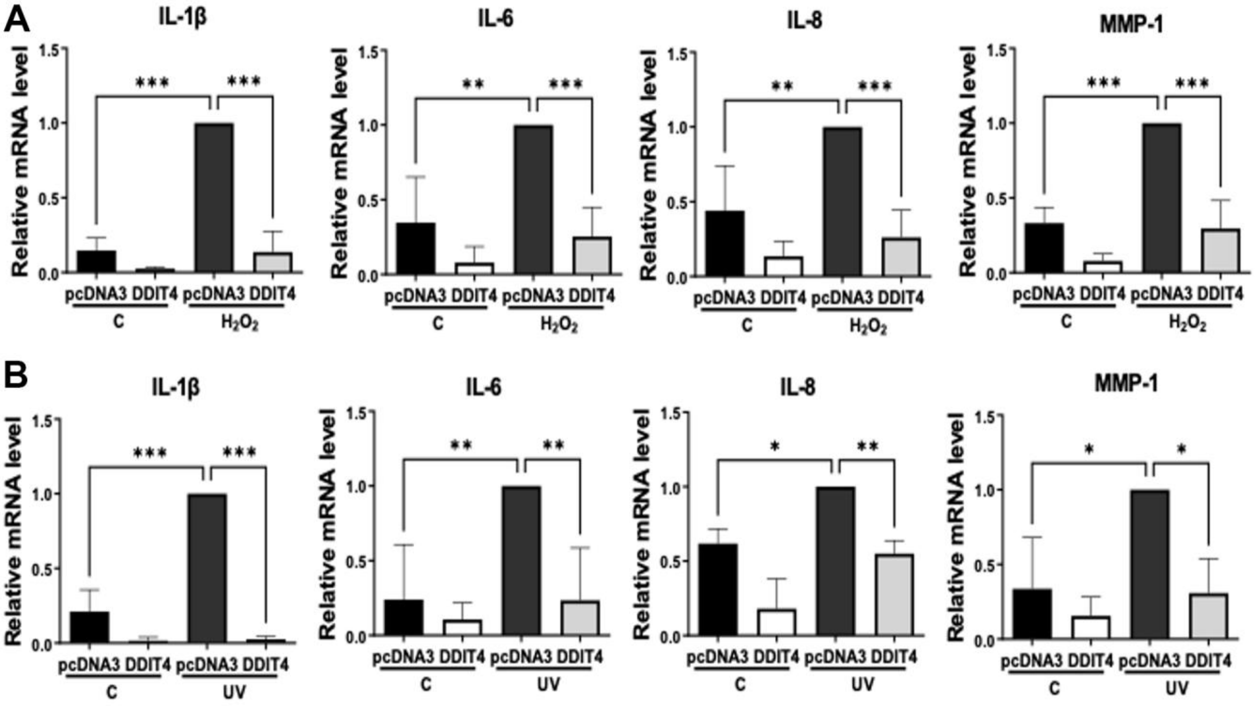

Supplementary Figure 5. DDIT4 regulates the expression of senescence-associated secretory phenotype (SASP) in senescent cells induced by H_2O_2 or UV. The mRNA levels of SASP components (IL-1β, IL-6, IL-8, and MMP-1) in human dermal fibroblasts induced by (**A**) H_2O_2 or (**B**) UV were determined by quantitative real-time PCR. Data represent the mean ± SE (n = 3-4, *P<0.05, ** P <0.01, *** P <0.001).

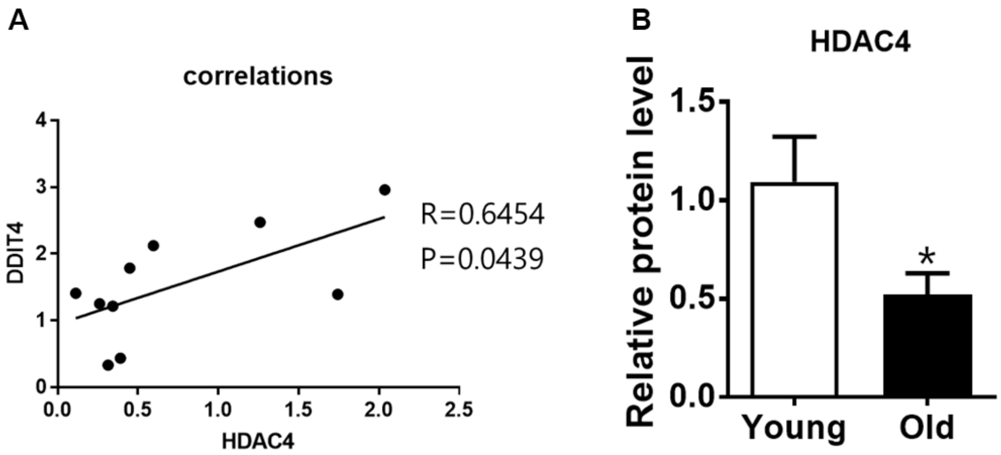

Supplementary Figure 6. HDAC4 and DDIT4 has a positive correlation in young and aged skin. (**A**) Correlation test was performed to evaluate the association between DDIT4 and HDAC4 (n=10) (**B**) The protein level of HDAC4 in skin tissues from young (n=5) and elderly (n=5) subjects. Data represent the mean ± SE (**P*<0.05).
