# Supplementary material for: Histone deacetylase 4 reverses cellular senescence via DDIT4 in dermal fibroblasts
Source: Aging (Albany NY). 2022 Jun 9;14(11):4653–72. doi: 10.18632/aging.204118 (PMC9217707; doi:10.18632/aging.204118)
Supplement: Supplementary Table 1 [file aging-14-204118-s002.docx]

Supplementary Table 1. Transcriptome analysis to identify the HDAC4 target genes.

| **Ensembl ID** | **Entrez ID** | **Symbol** | **log_2_(*FC*) [OE_ratio_ / KD_ratio_]** | **Pt [OE_ratio_ / KD_ratio_]** | **Pf [OE_ratio_ / KD_ratio_]** | **DEGs [OE_ratio_ / KD_ratio_]** |
| --- | --- | --- | --- | --- | --- | --- |
| ENSG00000013297 | 5010 | CLDN11 | 0.745 | 0.041 | 0.058 | induced by HDAC4 |
| ENSG00000023608 | 6617 | SNAPC1 | 0.738 | 0.017 | 0.059 | induced by HDAC4 |
| ENSG00000031691 | 55166 | CENPQ | 0.756 | 0.004 | 0.056 | induced by HDAC4 |
| ENSG00000040275 | 54908 | SPDL1 | 0.821 | 0.033 | 0.046 | induced by HDAC4 |
| ENSG00000065328 | 55388 | MCM10 | 0.619 | 0.024 | 0.089 | induced by HDAC4 |
| ENSG00000068024 | 9759 | HDAC4 | 5.117 | 0.000 | 0.000 | induced by HDAC4 |
| ENSG00000070831 | 998 | CDC42 | 0.817 | 0.018 | 0.046 | induced by HDAC4 |
| ENSG00000072274 | 7037 | TFRC | 1.076 | 0.015 | 0.023 | induced by HDAC4 |
| ENSG00000075336 | 29090 | TIMM21 | 0.677 | 0.012 | 0.073 | induced by HDAC4 |
| ENSG00000076382 | 10615 | SPAG5 | 0.612 | 0.019 | 0.091 | induced by HDAC4 |
| ENSG00000077152 | 29089 | UBE2T | 0.674 | 0.010 | 0.074 | induced by HDAC4 |
| ENSG00000081041 | 2920 | CXCL2 | 1.043 | 0.045 | 0.025 | induced by HDAC4 |
| ENSG00000082126 | 58538 | MPP4 | 0.645 | 0.028 | 0.081 | induced by HDAC4 |
| ENSG00000091651 | 23594 | ORC6 | 0.745 | 0.010 | 0.058 | induced by HDAC4 |
| ENSG00000092470 | 79968 | WDR76 | 0.678 | 0.041 | 0.073 | induced by HDAC4 |
| ENSG00000094804 | 990 | CDC6 | 0.818 | 0.006 | 0.046 | induced by HDAC4 |
| ENSG00000095752 | 3589 | IL11 | 1.058 | 0.001 | 0.024 | induced by HDAC4 |
| ENSG00000099860 | 4616 | GADD45B | 0.729 | 0.004 | 0.061 | induced by HDAC4 |
| ENSG00000100479 | 5427 | POLE2 | 0.719 | 0.046 | 0.063 | induced by HDAC4 |
| ENSG00000101447 | 81610 | FAM83D | 0.821 | 0.006 | 0.046 | induced by HDAC4 |
| ENSG00000101608 | 10627 | MYL12A | 0.757 | 0.024 | 0.056 | induced by HDAC4 |
| ENSG00000102218 | 6102 | RP2 | 0.992 | 0.002 | 0.028 | induced by HDAC4 |
| ENSG00000102794 | 730249 | ACOD1 | 0.904 | 0.000 | 0.036 | induced by HDAC4 |
| ENSG00000102931 | 23568 | ARL2BP | 0.635 | 0.048 | 0.084 | induced by HDAC4 |
| ENSG00000104381 | 54332 | GDAP1 | 0.616 | 0.039 | 0.090 | induced by HDAC4 |
| ENSG00000104889 | 10535 | RNASEH2A | 0.694 | 0.001 | 0.069 | induced by HDAC4 |
| ENSG00000105011 | 55723 | ASF1B | 0.652 | 0.037 | 0.079 | induced by HDAC4 |
| ENSG00000105889 | 256227 | STEAP1B | 0.666 | 0.012 | 0.076 | induced by HDAC4 |
| ENSG00000106392 | 56913 | C1GALT1 | 0.602 | 0.030 | 0.095 | induced by HDAC4 |
| ENSG00000108055 | 9126 | SMC3 | 0.598 | 0.040 | 0.096 | induced by HDAC4 |
| ENSG00000108064 | 7019 | TFAM | 1.178 | 0.013 | 0.018 | induced by HDAC4 |
| ENSG00000108342 | 1440 | CSF3 | 1.344 | 0.002 | 0.013 | induced by HDAC4 |
| ENSG00000108384 | 5889 | RAD51C | 0.617 | 0.029 | 0.090 | induced by HDAC4 |
| ENSG00000108688 | 6354 | CCL7 | 0.988 | 0.003 | 0.028 | induced by HDAC4 |
| ENSG00000108691 | 6347 | CCL2 | 1.369 | 0.047 | 0.012 | induced by HDAC4 |
| ENSG00000108854 | 64750 | SMURF2 | 0.618 | 0.048 | 0.090 | induced by HDAC4 |
| ENSG00000109881 | 91057 | CCDC34 | 0.719 | 0.033 | 0.063 | induced by HDAC4 |
| ENSG00000111247 | 10635 | RAD51AP1 | 0.719 | 0.007 | 0.063 | induced by HDAC4 |
| ENSG00000111445 | 5985 | RFC5 | 0.593 | 0.015 | 0.098 | induced by HDAC4 |
| ENSG00000111450 | 2054 | STX2 | 0.603 | 0.012 | 0.094 | induced by HDAC4 |
| ENSG00000111665 | 83461 | CDCA3 | 0.643 | 0.039 | 0.082 | induced by HDAC4 |
| ENSG00000112029 | 26271 | FBXO5 | 0.588 | 0.011 | 0.100 | induced by HDAC4 |
| ENSG00000112414 | 57211 | ADGRG6 | 1.183 | 0.012 | 0.018 | induced by HDAC4 |
| ENSG00000113368 | 4001 | LMNB1 | 0.707 | 0.049 | 0.066 | induced by HDAC4 |
| ENSG00000114450 | 59345 | GNB4 | 0.830 | 0.008 | 0.044 | induced by HDAC4 |
| ENSG00000115163 | 1058 | CENPA | 0.609 | 0.016 | 0.092 | induced by HDAC4 |
| ENSG00000115241 | 5496 | PPM1G | 0.783 | 0.022 | 0.051 | induced by HDAC4 |
| ENSG00000115520 | 80219 | COQ10B | 1.151 | 0.005 | 0.019 | induced by HDAC4 |
| ENSG00000115540 | 25843 | MOB4 | 0.625 | 0.005 | 0.087 | induced by HDAC4 |
| ENSG00000116679 | 10625 | IVNS1ABP | 0.659 | 0.033 | 0.077 | induced by HDAC4 |
| ENSG00000117650 | 4751 | NEK2 | 0.668 | 0.049 | 0.075 | induced by HDAC4 |
| ENSG00000118680 | 103910 | MYL12B | 0.719 | 0.007 | 0.063 | induced by HDAC4 |
| ENSG00000120539 | 84930 | MASTL | 0.691 | 0.028 | 0.069 | induced by HDAC4 |
| ENSG00000120802 | 7112 | TMPO | 0.772 | 0.023 | 0.053 | induced by HDAC4 |
| ENSG00000122952 | 11130 | ZWINT | 0.881 | 0.046 | 0.038 | induced by HDAC4 |
| ENSG00000123219 | 64105 | CENPK | 0.774 | 0.023 | 0.053 | induced by HDAC4 |
| ENSG00000123485 | 55355 | HJURP | 0.693 | 0.042 | 0.069 | induced by HDAC4 |
| ENSG00000124610 | 3024 | HIST1H1A | 0.819 | 0.006 | 0.046 | induced by HDAC4 |
| ENSG00000124635 | 8970 | HIST1H2BJ | 0.698 | 0.036 | 0.068 | induced by HDAC4 |
| ENSG00000124795 | 7913 | DEK | 0.624 | 0.008 | 0.088 | induced by HDAC4 |
| ENSG00000124875 | 6372 | CXCL6 | 0.775 | 0.027 | 0.053 | induced by HDAC4 |
| ENSG00000125249 | 5911 | RAP2A | 0.736 | 0.002 | 0.060 | induced by HDAC4 |
| ENSG00000125629 | 51141 | INSIG2 | 0.771 | 0.024 | 0.053 | induced by HDAC4 |
| ENSG00000128923 | 54629 | MINDY2 | 0.645 | 0.009 | 0.081 | induced by HDAC4 |
| ENSG00000128973 | 54982 | CLN6 | 0.736 | 0.041 | 0.060 | induced by HDAC4 |
| ENSG00000129355 | 1032 | CDKN2D | 0.674 | 0.034 | 0.074 | induced by HDAC4 |
| ENSG00000129810 | 151648 | SGO1 | 0.654 | 0.028 | 0.079 | induced by HDAC4 |
| ENSG00000131153 | 51659 | GINS2 | 0.667 | 0.011 | 0.075 | induced by HDAC4 |
| ENSG00000131470 | 29893 | PSMC3IP | 0.863 | 0.033 | 0.040 | induced by HDAC4 |
| ENSG00000132436 | 63979 | FIGNL1 | 0.668 | 0.003 | 0.075 | induced by HDAC4 |
| ENSG00000133119 | 5983 | RFC3 | 0.633 | 0.004 | 0.085 | induced by HDAC4 |
| ENSG00000133731 | 3612 | IMPA1 | 0.702 | 0.025 | 0.067 | induced by HDAC4 |
| ENSG00000134802 | 29015 | SLC43A3 | 0.678 | 0.006 | 0.073 | induced by HDAC4 |
| ENSG00000134970 | 51014 | TMED7 | 0.612 | 0.019 | 0.091 | induced by HDAC4 |
| ENSG00000136108 | 26586 | CKAP2 | 0.591 | 0.021 | 0.098 | induced by HDAC4 |
| ENSG00000136689 | 3557 | IL1RN | 0.635 | 0.006 | 0.084 | induced by HDAC4 |
| ENSG00000136824 | 10592 | SMC2 | 0.599 | 0.038 | 0.096 | induced by HDAC4 |
| ENSG00000137331 | 8870 | IER3 | 0.668 | 0.024 | 0.075 | induced by HDAC4 |
| ENSG00000137393 | 255488 | RNF144B | 0.646 | 0.007 | 0.081 | induced by HDAC4 |
| ENSG00000137710 | 5962 | RDX | 0.620 | 0.023 | 0.089 | induced by HDAC4 |
| ENSG00000138434 | 6744 | ITPRID2 | 0.706 | 0.010 | 0.066 | induced by HDAC4 |
| ENSG00000138604 | 26035 | GLCE | 0.661 | 0.011 | 0.077 | induced by HDAC4 |
| ENSG00000139133 | 84920 | ALG10 | 0.713 | 0.002 | 0.064 | induced by HDAC4 |
| ENSG00000142731 | 10733 | PLK4 | 0.714 | 0.004 | 0.064 | induced by HDAC4 |
| ENSG00000142945 | 11004 | KIF2C | 0.733 | 0.006 | 0.060 | induced by HDAC4 |
| ENSG00000143183 | 54499 | TMCO1 | 1.166 | 0.012 | 0.018 | induced by HDAC4 |
| ENSG00000143401 | 81611 | ANP32E | 0.662 | 0.030 | 0.077 | induced by HDAC4 |
| ENSG00000143476 | 51514 | DTL | 0.678 | 0.005 | 0.072 | induced by HDAC4 |
| ENSG00000144810 | 1295 | COL8A1 | 0.854 | 0.029 | 0.041 | induced by HDAC4 |
| ENSG00000145386 | 890 | CCNA2 | 0.842 | 0.036 | 0.043 | induced by HDAC4 |
| ENSG00000146670 | 113130 | CDCA5 | 0.676 | 0.035 | 0.073 | induced by HDAC4 |
| ENSG00000146842 | 84928 | TMEM209 | 0.655 | 0.022 | 0.078 | induced by HDAC4 |
| ENSG00000147224 | 5631 | PRPS1 | 0.649 | 0.037 | 0.080 | induced by HDAC4 |
| ENSG00000148229 | 54107 | POLE3 | 0.598 | 0.012 | 0.096 | induced by HDAC4 |
| ENSG00000148411 | 138151 | NACC2 | 0.638 | 0.007 | 0.083 | induced by HDAC4 |
| ENSG00000149636 | 79980 | DSN1 | 0.626 | 0.040 | 0.087 | induced by HDAC4 |
| ENSG00000151239 | 5756 | TWF1 | 0.899 | 0.005 | 0.036 | induced by HDAC4 |
| ENSG00000151287 | 93081 | TEX30 | 1.323 | 0.000 | 0.013 | induced by HDAC4 |
| ENSG00000152253 | 57405 | SPC25 | 0.704 | 0.029 | 0.066 | induced by HDAC4 |
| ENSG00000152689 | 25780 | RASGRP3 | 0.762 | 0.027 | 0.055 | induced by HDAC4 |
| ENSG00000153044 | 64946 | CENPH | 0.915 | 0.019 | 0.035 | induced by HDAC4 |
| ENSG00000153140 | 1070 | CETN3 | 1.078 | 0.026 | 0.023 | induced by HDAC4 |
| ENSG00000153179 | 283349 | RASSF3 | 0.626 | 0.004 | 0.087 | induced by HDAC4 |
| ENSG00000154188 | 284 | ANGPT1 | 0.605 | 0.008 | 0.094 | induced by HDAC4 |
| ENSG00000154319 | 83648 | FAM167A | 0.783 | 0.006 | 0.051 | induced by HDAC4 |
| ENSG00000154429 | 126731 | CCSAP | 0.655 | 0.012 | 0.079 | induced by HDAC4 |
| ENSG00000154839 | 220134 | SKA1 | 0.654 | 0.046 | 0.079 | induced by HDAC4 |
| ENSG00000154920 | 146956 | EME1 | 0.734 | 0.016 | 0.060 | induced by HDAC4 |
| ENSG00000155115 | 112495 | GTF3C6 | 0.591 | 0.018 | 0.099 | induced by HDAC4 |
| ENSG00000156136 | 1633 | DCK | 0.613 | 0.021 | 0.091 | induced by HDAC4 |
| ENSG00000158164 | 11013 | TMSB15A | 0.610 | 0.011 | 0.092 | induced by HDAC4 |
| ENSG00000159055 | 54069 | MIS18A | 0.692 | 0.004 | 0.069 | induced by HDAC4 |
| ENSG00000159147 | 29980 | DONSON | 0.679 | 0.037 | 0.072 | induced by HDAC4 |
| ENSG00000159200 | 1827 | RCAN1 | 0.665 | 0.004 | 0.076 | induced by HDAC4 |
| ENSG00000160113 | 2063 | NR2F6 | 0.642 | 0.027 | 0.082 | induced by HDAC4 |
| ENSG00000160131 | 203547 | VMA21 | 0.589 | 0.011 | 0.099 | induced by HDAC4 |
| ENSG00000162433 | 205 | AK4 | 0.600 | 0.005 | 0.096 | induced by HDAC4 |
| ENSG00000162521 | 5928 | RBBP4 | 0.913 | 0.021 | 0.035 | induced by HDAC4 |
| ENSG00000163808 | 56992 | KIF15 | 0.699 | 0.047 | 0.067 | induced by HDAC4 |
| ENSG00000163923 | 116832 | RPL39L | 0.711 | 0.002 | 0.065 | induced by HDAC4 |
| ENSG00000164104 | 3148 | HMGB2 | 0.819 | 0.012 | 0.046 | induced by HDAC4 |
| ENSG00000164105 | 8819 | SAP30 | 0.663 | 0.027 | 0.076 | induced by HDAC4 |
| ENSG00000164109 | 4085 | MAD2L1 | 0.798 | 0.036 | 0.049 | induced by HDAC4 |
| ENSG00000164117 | 26269 | FBXO8 | 0.620 | 0.002 | 0.089 | induced by HDAC4 |
| ENSG00000164283 | 11082 | ESM1 | 1.031 | 0.003 | 0.025 | induced by HDAC4 |
| ENSG00000164574 | 55568 | GALNT10 | 0.686 | 0.004 | 0.070 | induced by HDAC4 |
| ENSG00000164687 | 2171 | FABP5 | 0.699 | 0.005 | 0.068 | induced by HDAC4 |
| ENSG00000164985 | 11168 | PSIP1 | 0.724 | 0.014 | 0.062 | induced by HDAC4 |
| ENSG00000165244 | 195828 | ZNF367 | 0.831 | 0.026 | 0.044 | induced by HDAC4 |
| ENSG00000166002 | 56935 | SMCO4 | 0.734 | 0.030 | 0.060 | induced by HDAC4 |
| ENSG00000166508 | 4176 | MCM7 | 0.638 | 0.047 | 0.083 | induced by HDAC4 |
| ENSG00000166582 | 201161 | CENPV | 0.644 | 0.042 | 0.082 | induced by HDAC4 |
| ENSG00000166801 | 63901 | FAM111A | 0.631 | 0.024 | 0.085 | induced by HDAC4 |
| ENSG00000167513 | 81620 | CDT1 | 0.723 | 0.004 | 0.062 | induced by HDAC4 |
| ENSG00000167695 | 79850 | FAM57A | 0.661 | 0.015 | 0.077 | induced by HDAC4 |
| ENSG00000167747 | 84798 | C19orf48 | 0.621 | 0.050 | 0.089 | induced by HDAC4 |
| ENSG00000168209 | 54541 | DDIT4 | 0.639 | 0.047 | 0.083 | induced by HDAC4 |
| ENSG00000168286 | 57215 | THAP11 | 0.629 | 0.015 | 0.086 | induced by HDAC4 |
| ENSG00000168288 | 27249 | MMADHC | 0.678 | 0.018 | 0.073 | induced by HDAC4 |
| ENSG00000168411 | 55159 | RFWD3 | 0.597 | 0.029 | 0.097 | induced by HDAC4 |
| ENSG00000168496 | 2237 | FEN1 | 0.666 | 0.040 | 0.075 | induced by HDAC4 |
| ENSG00000168685 | 3575 | IL7R | 0.758 | 0.024 | 0.056 | induced by HDAC4 |
| ENSG00000169379 | 200894 | ARL13B | 0.623 | 0.026 | 0.088 | induced by HDAC4 |
| ENSG00000169599 | 27247 | NFU1 | 0.599 | 0.001 | 0.096 | induced by HDAC4 |
| ENSG00000169607 | 150468 | CKAP2L | 0.700 | 0.016 | 0.067 | induced by HDAC4 |
| ENSG00000169908 | 4071 | TM4SF1 | 0.987 | 0.004 | 0.029 | induced by HDAC4 |
| ENSG00000170312 | 983 | CDK1 | 0.701 | 0.019 | 0.067 | induced by HDAC4 |
| ENSG00000170540 | 23204 | ARL6IP1 | 0.823 | 0.038 | 0.045 | induced by HDAC4 |
| ENSG00000171848 | 6241 | RRM2 | 0.796 | 0.028 | 0.049 | induced by HDAC4 |
| ENSG00000174010 | 80311 | KLHL15 | 0.712 | 0.005 | 0.065 | induced by HDAC4 |
| ENSG00000174371 | 9156 | EXO1 | 0.785 | 0.026 | 0.051 | induced by HDAC4 |
| ENSG00000175063 | 11065 | UBE2C | 0.755 | 0.001 | 0.056 | induced by HDAC4 |
| ENSG00000176171 | 664 | BNIP3 | 0.661 | 0.029 | 0.077 | induced by HDAC4 |
| ENSG00000180198 | 1104 | RCC1 | 0.602 | 0.036 | 0.095 | induced by HDAC4 |
| ENSG00000180801 | 79642 | ARSJ | 0.691 | 0.007 | 0.069 | induced by HDAC4 |
| ENSG00000181938 | 64785 | GINS3 | 0.642 | 0.030 | 0.082 | induced by HDAC4 |
| ENSG00000182010 | 219790 | RTKN2 | 0.613 | 0.016 | 0.091 | induced by HDAC4 |
| ENSG00000183283 | 9802 | DAZAP2 | 0.746 | 0.005 | 0.058 | induced by HDAC4 |
| ENSG00000185697 | 4603 | MYBL1 | 0.635 | 0.043 | 0.084 | induced by HDAC4 |
| ENSG00000185900 | 84197 | POMK | 0.731 | 0.007 | 0.061 | induced by HDAC4 |
| ENSG00000186193 | 89958 | SAPCD2 | 0.672 | 0.005 | 0.074 | induced by HDAC4 |
| ENSG00000187193 | 4501 | MT1X | 0.631 | 0.001 | 0.086 | induced by HDAC4 |
| ENSG00000187266 | 2057 | EPOR | 0.591 | 0.007 | 0.099 | induced by HDAC4 |
| ENSG00000188486 | 3014 | H2AFX | 0.715 | 0.006 | 0.064 | induced by HDAC4 |
| ENSG00000189057 | 374393 | FAM111B | 0.833 | 0.049 | 0.044 | induced by HDAC4 |
| ENSG00000189362 | 100131211 | NEMP2 | 0.658 | 0.031 | 0.078 | induced by HDAC4 |
| ENSG00000196081 | 440519 | ZNF724 | 0.590 | 0.006 | 0.099 | induced by HDAC4 |
| ENSG00000196866 | 3013 | HIST1H2AD | 1.275 | 0.008 | 0.015 | induced by HDAC4 |
| ENSG00000196890 | 128312 | HIST3H2BB | 0.617 | 0.008 | 0.090 | induced by HDAC4 |
| ENSG00000197153 | 8356 | HIST1H3J | 0.676 | 0.019 | 0.073 | induced by HDAC4 |
| ENSG00000197632 | 5055 | SERPINB2 | 0.869 | 0.007 | 0.039 | induced by HDAC4 |
| ENSG00000197646 | 80380 | PDCD1LG2 | 0.718 | 0.013 | 0.063 | induced by HDAC4 |
| ENSG00000198056 | 5557 | PRIM1 | 0.876 | 0.016 | 0.039 | induced by HDAC4 |
| ENSG00000202444 | NA | RNU5E-6P | 1.222 | 0.034 | 0.016 | induced by HDAC4 |
| ENSG00000203814 | 440689 | HIST2H2BF | 0.599 | 0.001 | 0.096 | induced by HDAC4 |
| ENSG00000203818 | 440686 | HIST2H3PS2 | 0.951 | 0.044 | 0.031 | induced by HDAC4 |
| ENSG00000206838 | 654319 | SNORA5A | 0.663 | 0.000 | 0.076 | induced by HDAC4 |
| ENSG00000207304 | 654320 | SNORA8 | 0.895 | 0.027 | 0.037 | induced by HDAC4 |
| ENSG00000213186 | 286827 | TRIM59 | 0.605 | 0.024 | 0.094 | induced by HDAC4 |
| ENSG00000213347 | 83463 | MXD3 | 0.606 | 0.006 | 0.094 | induced by HDAC4 |
| ENSG00000222724 | NA | RNU2-63P | 0.636 | 0.046 | 0.084 | induced by HDAC4 |
| ENSG00000239268 | NA | AC092691.1 | 0.709 | 0.000 | 0.065 | induced by HDAC4 |
| ENSG00000240929 | NA | HIST2H2BB | 0.642 | 0.020 | 0.082 | induced by HDAC4 |
| ENSG00000243960 | NA | AL390195.1 | 1.037 | 0.003 | 0.025 | induced by HDAC4 |
| ENSG00000244357 | NA | RN7SL145P | 0.746 | 0.003 | 0.058 | induced by HDAC4 |
| ENSG00000249456 | NA | AL731577.2 | 0.778 | 0.028 | 0.052 | induced by HDAC4 |
| ENSG00000252906 | 677679 | SCARNA3 | 0.774 | 0.009 | 0.053 | induced by HDAC4 |
| ENSG00000255893 | NA | AP000786.1 | 0.617 | 0.047 | 0.090 | induced by HDAC4 |
| ENSG00000257219 | 105369848 | LINC02407 | 0.674 | 0.046 | 0.073 | induced by HDAC4 |
| ENSG00000258818 | 6038 | RNASE4 | 1.032 | 0.014 | 0.025 | induced by HDAC4 |
| ENSG00000272606 | NA | AC015982.1 | 0.730 | 0.031 | 0.061 | induced by HDAC4 |
| ENSG00000273802 | 8339 | HIST1H2BG | 0.587 | 0.019 | 0.100 | induced by HDAC4 |
| ENSG00000274618 | 8361 | HIST1H4F | 0.612 | 0.039 | 0.091 | induced by HDAC4 |
| ENSG00000274641 | 8348 | HIST1H2BO | 0.672 | 0.026 | 0.074 | induced by HDAC4 |
| ENSG00000274750 | 8353 | HIST1H3E | 0.712 | 0.008 | 0.065 | induced by HDAC4 |
| ENSG00000274997 | 85235 | HIST1H2AH | 0.682 | 0.031 | 0.072 | induced by HDAC4 |
| ENSG00000275126 | 8368 | HIST1H4L | 0.853 | 0.007 | 0.041 | induced by HDAC4 |
| ENSG00000275221 | 8330 | HIST1H2AK | 0.591 | 0.017 | 0.099 | induced by HDAC4 |
| ENSG00000275379 | 8354 | HIST1H3I | 0.682 | 0.010 | 0.071 | induced by HDAC4 |
| ENSG00000275713 | 8345 | HIST1H2BH | 0.678 | 0.035 | 0.073 | induced by HDAC4 |
| ENSG00000276180 | 8294 | HIST1H4I | 0.676 | 0.032 | 0.073 | induced by HDAC4 |
| ENSG00000276410 | 3018 | HIST1H2BB | 0.628 | 0.046 | 0.086 | induced by HDAC4 |
| ENSG00000276903 | 8332 | HIST1H2AL | 0.677 | 0.014 | 0.073 | induced by HDAC4 |
| ENSG00000277775 | 8968 | HIST1H3F | 0.665 | 0.001 | 0.076 | induced by HDAC4 |
| ENSG00000278272 | 8352 | HIST1H3C | 0.631 | 0.027 | 0.085 | induced by HDAC4 |
| ENSG00000278815 | 100126299 | RF00006 | 1.488 | 0.009 | 0.010 | induced by HDAC4 |
| ENSG00000280123 | NA | AC023632.6 | 0.620 | 0.015 | 0.089 | induced by HDAC4 |
| ENSG00000281383 | NA | FP671120.4 | 1.404 | 0.000 | 0.011 | induced by HDAC4 |
| ENSG00000001461 | 57185 | NIPAL3 | -0.606 | 0.016 | 0.094 | inhibited by HDAC4 |
| ENSG00000002746 | 23072 | HECW1 | -1.106 | 0.042 | 0.022 | inhibited by HDAC4 |
| ENSG00000004399 | 23129 | PLXND1 | -0.686 | 0.023 | 0.071 | inhibited by HDAC4 |
| ENSG00000009413 | 5980 | REV3L | -0.782 | 0.034 | 0.052 | inhibited by HDAC4 |
| ENSG00000024422 | 30846 | EHD2 | -0.591 | 0.023 | 0.099 | inhibited by HDAC4 |
| ENSG00000052795 | 57600 | FNIP2 | -0.676 | 0.029 | 0.074 | inhibited by HDAC4 |
| ENSG00000072121 | 23503 | ZFYVE26 | -0.640 | 0.023 | 0.083 | inhibited by HDAC4 |
| ENSG00000075568 | 23505 | TMEM131 | -0.685 | 0.043 | 0.071 | inhibited by HDAC4 |
| ENSG00000078725 | 1620 | BRINP1 | -0.888 | 0.036 | 0.038 | inhibited by HDAC4 |
| ENSG00000079102 | 862 | RUNX1T1 | -0.684 | 0.031 | 0.072 | inhibited by HDAC4 |
| ENSG00000079308 | 7145 | TNS1 | -0.646 | 0.040 | 0.082 | inhibited by HDAC4 |
| ENSG00000080845 | 22839 | DLGAP4 | -0.628 | 0.020 | 0.087 | inhibited by HDAC4 |
| ENSG00000086544 | 80271 | ITPKC | -0.616 | 0.049 | 0.091 | inhibited by HDAC4 |
| ENSG00000087076 | 51171 | HSD17B14 | -1.200 | 0.002 | 0.018 | inhibited by HDAC4 |
| ENSG00000089159 | 5829 | PXN | -0.766 | 0.009 | 0.055 | inhibited by HDAC4 |
| ENSG00000091844 | 26575 | RGS17 | -0.677 | 0.032 | 0.073 | inhibited by HDAC4 |
| ENSG00000099377 | 80270 | HSD3B7 | -0.945 | 0.035 | 0.033 | inhibited by HDAC4 |
| ENSG00000100284 | 10043 | TOM1 | -0.625 | 0.036 | 0.088 | inhibited by HDAC4 |
| ENSG00000100439 | 63874 | ABHD4 | -0.589 | 0.018 | 0.100 | inhibited by HDAC4 |
| ENSG00000103066 | 23659 | PLA2G15 | -0.699 | 0.015 | 0.068 | inhibited by HDAC4 |
| ENSG00000105953 | 4967 | OGDH | -0.701 | 0.005 | 0.068 | inhibited by HDAC4 |
| ENSG00000108846 | 8714 | ABCC3 | -0.765 | 0.043 | 0.055 | inhibited by HDAC4 |
| ENSG00000110237 | 9828 | ARHGEF17 | -0.671 | 0.034 | 0.075 | inhibited by HDAC4 |
| ENSG00000111077 | 23371 | TNS2 | -0.857 | 0.028 | 0.042 | inhibited by HDAC4 |
| ENSG00000112902 | 9037 | SEMA5A | -0.703 | 0.026 | 0.067 | inhibited by HDAC4 |
| ENSG00000113552 | 10007 | GNPDA1 | -0.594 | 0.006 | 0.098 | inhibited by HDAC4 |
| ENSG00000114270 | 1294 | COL7A1 | -0.634 | 0.042 | 0.085 | inhibited by HDAC4 |
| ENSG00000115414 | 2335 | FN1 | -0.667 | 0.034 | 0.076 | inhibited by HDAC4 |
| ENSG00000116353 | 51102 | MECR | -0.650 | 0.008 | 0.081 | inhibited by HDAC4 |
| ENSG00000116688 | 9927 | MFN2 | -0.696 | 0.040 | 0.069 | inhibited by HDAC4 |
| ENSG00000118596 | 9194 | SLC16A7 | -0.590 | 0.006 | 0.099 | inhibited by HDAC4 |
| ENSG00000118785 | 6696 | SPP1 | -1.466 | 0.026 | 0.011 | inhibited by HDAC4 |
| ENSG00000119630 | 5228 | PGF | -0.646 | 0.033 | 0.081 | inhibited by HDAC4 |
| ENSG00000119986 | 60370 | AVPI1 | -0.687 | 0.044 | 0.071 | inhibited by HDAC4 |
| ENSG00000120594 | 84898 | PLXDC2 | -0.620 | 0.017 | 0.089 | inhibited by HDAC4 |
| ENSG00000123643 | 206358 | SLC36A1 | -0.627 | 0.018 | 0.087 | inhibited by HDAC4 |
| ENSG00000124208 | 387522 | TMEM189-UBE2V1 | -0.996 | 0.044 | 0.029 | inhibited by HDAC4 |
| ENSG00000124762 | 1026 | CDKN1A | -0.950 | 0.039 | 0.032 | inhibited by HDAC4 |
| ENSG00000126005 | 101410538 | MMP24OS | -0.662 | 0.021 | 0.077 | inhibited by HDAC4 |
| ENSG00000129925 | 58986 | TMEM8A | -0.779 | 0.039 | 0.053 | inhibited by HDAC4 |
| ENSG00000133466 | 114904 | C1QTNF6 | -0.741 | 0.026 | 0.059 | inhibited by HDAC4 |
| ENSG00000133687 | 83857 | TMTC1 | -0.683 | 0.019 | 0.072 | inhibited by HDAC4 |
| ENSG00000135218 | 948 | CD36 | -0.760 | 0.031 | 0.056 | inhibited by HDAC4 |
| ENSG00000135677 | 2799 | GNS | -0.657 | 0.001 | 0.079 | inhibited by HDAC4 |
| ENSG00000135744 | 183 | AGT | -1.223 | 0.000 | 0.017 | inhibited by HDAC4 |
| ENSG00000135749 | 80003 | PCNX2 | -0.627 | 0.023 | 0.087 | inhibited by HDAC4 |
| ENSG00000136235 | 10457 | GPNMB | -0.703 | 0.010 | 0.067 | inhibited by HDAC4 |
| ENSG00000136826 | 9314 | KLF4 | -0.944 | 0.019 | 0.033 | inhibited by HDAC4 |
| ENSG00000136869 | 7099 | TLR4 | -0.848 | 0.013 | 0.043 | inhibited by HDAC4 |
| ENSG00000136960 | 5168 | ENPP2 | -0.714 | 0.010 | 0.065 | inhibited by HDAC4 |
| ENSG00000136999 | 4856 | NOV | -1.157 | 0.009 | 0.020 | inhibited by HDAC4 |
| ENSG00000138061 | 1545 | CYP1B1 | -0.929 | 0.001 | 0.034 | inhibited by HDAC4 |
| ENSG00000139636 | 55716 | LMBR1L | -0.768 | 0.025 | 0.055 | inhibited by HDAC4 |
| ENSG00000140948 | 23174 | ZCCHC14 | -0.728 | 0.031 | 0.062 | inhibited by HDAC4 |
| ENSG00000141376 | 54828 | BCAS3 | -0.725 | 0.029 | 0.063 | inhibited by HDAC4 |
| ENSG00000141458 | 4864 | NPC1 | -1.103 | 0.006 | 0.022 | inhibited by HDAC4 |
| ENSG00000143387 | 1513 | CTSK | -0.721 | 0.005 | 0.063 | inhibited by HDAC4 |
| ENSG00000143669 | 1130 | LYST | -0.692 | 0.043 | 0.070 | inhibited by HDAC4 |
| ENSG00000143878 | 388 | RHOB | -0.689 | 0.029 | 0.070 | inhibited by HDAC4 |
| ENSG00000145362 | 287 | ANK2 | -0.791 | 0.016 | 0.051 | inhibited by HDAC4 |
| ENSG00000147573 | 84675 | TRIM55 | -0.824 | 0.031 | 0.046 | inhibited by HDAC4 |
| ENSG00000147813 | 93100 | NAPRT | -0.703 | 0.020 | 0.067 | inhibited by HDAC4 |
| ENSG00000151012 | 23657 | SLC7A11 | -1.162 | 0.048 | 0.019 | inhibited by HDAC4 |
| ENSG00000151176 | 196463 | PLBD2 | -0.816 | 0.028 | 0.047 | inhibited by HDAC4 |
| ENSG00000152137 | 26353 | HSPB8 | -0.848 | 0.002 | 0.043 | inhibited by HDAC4 |
| ENSG00000152518 | 678 | ZFP36L2 | -0.638 | 0.028 | 0.084 | inhibited by HDAC4 |
| ENSG00000153395 | 79888 | LPCAT1 | -0.834 | 0.010 | 0.045 | inhibited by HDAC4 |
| ENSG00000153944 | 124540 | MSI2 | -0.823 | 0.015 | 0.046 | inhibited by HDAC4 |
| ENSG00000156804 | 114907 | FBXO32 | -0.831 | 0.019 | 0.045 | inhibited by HDAC4 |
| ENSG00000157227 | 4323 | MMP14 | -0.645 | 0.050 | 0.082 | inhibited by HDAC4 |
| ENSG00000159388 | 7832 | BTG2 | -1.033 | 0.022 | 0.026 | inhibited by HDAC4 |
| ENSG00000159674 | 10417 | SPON2 | -0.915 | 0.045 | 0.036 | inhibited by HDAC4 |
| ENSG00000159792 | 5681 | PSKH1 | -0.626 | 0.033 | 0.087 | inhibited by HDAC4 |
| ENSG00000160145 | 8997 | KALRN | -0.980 | 0.018 | 0.030 | inhibited by HDAC4 |
| ENSG00000160404 | 27433 | TOR2A | -0.624 | 0.039 | 0.088 | inhibited by HDAC4 |
| ENSG00000161249 | 93099 | DMKN | -0.828 | 0.023 | 0.045 | inhibited by HDAC4 |
| ENSG00000162804 | 25992 | SNED1 | -0.664 | 0.039 | 0.077 | inhibited by HDAC4 |
| ENSG00000163071 | 132671 | SPATA18 | -0.676 | 0.020 | 0.074 | inhibited by HDAC4 |
| ENSG00000163947 | 50650 | ARHGEF3 | -0.597 | 0.007 | 0.097 | inhibited by HDAC4 |
| ENSG00000164877 | 79778 | MICALL2 | -0.660 | 0.004 | 0.078 | inhibited by HDAC4 |
| ENSG00000164938 | 94241 | TP53INP1 | -0.616 | 0.035 | 0.090 | inhibited by HDAC4 |
| ENSG00000165029 | 19 | ABCA1 | -1.215 | 0.004 | 0.017 | inhibited by HDAC4 |
| ENSG00000165861 | 53349 | ZFYVE1 | -0.683 | 0.011 | 0.072 | inhibited by HDAC4 |
| ENSG00000166340 | 1200 | TPP1 | -0.948 | 0.027 | 0.032 | inhibited by HDAC4 |
| ENSG00000166922 | 6447 | SCG5 | -0.944 | 0.026 | 0.033 | inhibited by HDAC4 |
| ENSG00000167566 | 57701 | NCKAP5L | -0.598 | 0.010 | 0.097 | inhibited by HDAC4 |
| ENSG00000167615 | 114823 | LENG8 | -0.743 | 0.025 | 0.059 | inhibited by HDAC4 |
| ENSG00000167632 | 83696 | TRAPPC9 | -0.642 | 0.007 | 0.083 | inhibited by HDAC4 |
| ENSG00000168477 | 7148 | TNXB | -0.985 | 0.016 | 0.030 | inhibited by HDAC4 |
| ENSG00000168610 | 6774 | STAT3 | -0.718 | 0.032 | 0.064 | inhibited by HDAC4 |
| ENSG00000168961 | 3965 | LGALS9 | -0.812 | 0.045 | 0.048 | inhibited by HDAC4 |
| ENSG00000169184 | 4330 | MN1 | -0.776 | 0.013 | 0.053 | inhibited by HDAC4 |
| ENSG00000170903 | 84437 | MSANTD4 | -0.601 | 0.020 | 0.095 | inhibited by HDAC4 |
| ENSG00000171444 | 4163 | MCC | -0.589 | 0.044 | 0.100 | inhibited by HDAC4 |
| ENSG00000173267 | 6623 | SNCG | -0.883 | 0.026 | 0.039 | inhibited by HDAC4 |
| ENSG00000173535 | 8794 | TNFRSF10C | -1.034 | 0.006 | 0.026 | inhibited by HDAC4 |
| ENSG00000174456 | 400073 | C12orf76 | -0.589 | 0.014 | 0.100 | inhibited by HDAC4 |
| ENSG00000177000 | 4524 | MTHFR | -0.671 | 0.039 | 0.075 | inhibited by HDAC4 |
| ENSG00000177106 | 64787 | EPS8L2 | -0.707 | 0.035 | 0.066 | inhibited by HDAC4 |
| ENSG00000177337 | 649446 | DLGAP1-AS1 | -0.593 | 0.012 | 0.098 | inhibited by HDAC4 |
| ENSG00000180155 | 66004 | LYNX1 | -0.724 | 0.006 | 0.063 | inhibited by HDAC4 |
| ENSG00000183508 | 54855 | TENT5C | -0.816 | 0.016 | 0.047 | inhibited by HDAC4 |
| ENSG00000184232 | 220323 | OAF | -0.624 | 0.025 | 0.088 | inhibited by HDAC4 |
| ENSG00000185567 | 113146 | AHNAK2 | -1.576 | 0.027 | 0.009 | inhibited by HDAC4 |
| ENSG00000186174 | 283149 | BCL9L | -0.705 | 0.007 | 0.067 | inhibited by HDAC4 |
| ENSG00000187134 | 1645 | AKR1C1 | -0.905 | 0.016 | 0.036 | inhibited by HDAC4 |
| ENSG00000187391 | 9863 | MAGI2 | -0.626 | 0.047 | 0.087 | inhibited by HDAC4 |
| ENSG00000196498 | 9612 | NCOR2 | -0.590 | 0.031 | 0.099 | inhibited by HDAC4 |
| ENSG00000197081 | 3482 | IGF2R | -1.069 | 0.023 | 0.024 | inhibited by HDAC4 |
| ENSG00000197635 | 1803 | DPP4 | -0.608 | 0.032 | 0.093 | inhibited by HDAC4 |
| ENSG00000198074 | 57016 | AKR1B10 | -1.095 | 0.000 | 0.023 | inhibited by HDAC4 |
| ENSG00000198715 | 112770 | GLMP | -0.649 | 0.031 | 0.081 | inhibited by HDAC4 |
| ENSG00000198959 | 7052 | TGM2 | -0.813 | 0.005 | 0.047 | inhibited by HDAC4 |
| ENSG00000201955 | NA | RNY3P1 | -1.021 | 0.000 | 0.027 | inhibited by HDAC4 |
| ENSG00000214575 | 64506 | CPEB1 | -0.642 | 0.004 | 0.083 | inhibited by HDAC4 |
| ENSG00000214655 | 23053 | ZSWIM8 | -0.618 | 0.038 | 0.090 | inhibited by HDAC4 |
| ENSG00000223701 | 100652739 | RAET1E-AS1 | -0.738 | 0.039 | 0.060 | inhibited by HDAC4 |
| ENSG00000225968 | 392617 | ELFN1 | -0.711 | 0.003 | 0.065 | inhibited by HDAC4 |
| ENSG00000226318 | NA | RPS3AP38 | -0.691 | 0.029 | 0.070 | inhibited by HDAC4 |
| ENSG00000231721 | 378805 | LINC-PINT | -0.602 | 0.041 | 0.095 | inhibited by HDAC4 |
| ENSG00000231924 | 5669 | PSG1 | -0.922 | 0.014 | 0.035 | inhibited by HDAC4 |
| ENSG00000238045 | NA | AC009133.1 | -1.082 | 0.029 | 0.023 | inhibited by HDAC4 |
| ENSG00000241529 | NA | RN7SL767P | -0.747 | 0.005 | 0.058 | inhibited by HDAC4 |
| ENSG00000241973 | 5297 | PI4KA | -0.813 | 0.005 | 0.047 | inhibited by HDAC4 |
| ENSG00000243260 | NA | RN7SL558P | -0.999 | 0.000 | 0.029 | inhibited by HDAC4 |
| ENSG00000252577 | 677681 | SCARNA20 | -0.989 | 0.000 | 0.029 | inhibited by HDAC4 |
| ENSG00000259171 | NA | AL163636.2 | -0.879 | 0.032 | 0.039 | inhibited by HDAC4 |
| ENSG00000261832 | NA | AC138894.1 | -1.518 | 0.031 | 0.010 | inhibited by HDAC4 |
| ENSG00000265735 | NA | RN7SL5P | -0.917 | 0.001 | 0.035 | inhibited by HDAC4 |
| ENSG00000269997 | NA | AC068790.3 | -0.814 | 0.029 | 0.047 | inhibited by HDAC4 |
| ENSG00000280498 | 692073 | SNORA16A | -1.041 | 0.000 | 0.026 | inhibited by HDAC4 |
