# Supplementary material for: Histone deacetylase 4 reverses cellular senescence via DDIT4 in dermal fibroblasts
Source: Aging (Albany NY). 2022 Jun 9;14(11):4653–72. doi: 10.18632/aging.204118 (PMC9217707; doi:10.18632/aging.204118)
Supplement: Supplementary Table 2 [file aging-14-204118-s003.pdf]

Supplementary Table 2. Sample information for public microarray datasets.

Senescence type	Paper	GEO ID	Sample accession	Sample name	Organism	Cell type	Treatment	Detail information for treatment	Platform
UV-induced model	Solar-simulated ultraviolet radiation induces histone 3 methylation changes in the gene promoters of matrix metalloproteinases 1 and 3 in primary human dermal fibroblasts.	GSE60795	GSM1488774	UV sample1	Human	Primary neonatal human dermal fibroblasts	ssUVR irradiation exposure	The cells were exposed to 12 J/cm^2 ssUVR a single time on a modified Hand Foot II phototherapy instrument (National Biological Corporation, Beachwood, OH). This instrument was fitted with 8 UVA bulbs (HOUVALITE F24T12/BL/HO [PUVA], National Biological Corporation) that emit 95% UVA and 5% UVB. UVA intensity was measured with a UVX digital handheld radiometer using the UVX-36 UVA sensor (UVP, Upland, CA). UVB intensity was measured with an ILT1400 Photo detector using the SEL240 UVB sensor (International Light, Newburyport, MA). The cells recovered in fresh medium under normal culture conditions for 24 hours prior to collection.	[HuGene-1_0-st] Affymetrix GeneChip Human Exon 1.0 ST array
UV-induced model		GSE60795	GSM1488775	UV sample2	Human	Primary neonatal human dermal fibroblasts	ssUVR irradiation exposure		[HuGene-1_0-st] Affymetrix GeneChip Human Exon 1.0 ST array
UV-induced model		GSE60795	GSM1488778	Sham sample1	Human	Primary neonatal human dermal fibroblasts	Sham exposure	A sham exposure group (0 J/cm^2 ssUVR). The cells recovered in fresh medium under normal culture conditions for 24 hours prior to collection.	[HuGene-1_0-st] Affymetrix GeneChip Human Exon 1.0 ST array
UV-induced model		GSE60795	GSM1488779	Sham sample2	Human	Primary neonatal human dermal fibroblasts	Sham exposure		[HuGene-1_0-st] Affymetrix GeneChip Human Exon 1.0 ST array
H_2O_2-induced model	Pan-senescence transcriptome analysis identified RRAD as a marker and negative regulator of cellular senescence	GSE116761	GSM3260257	H_2O_2 sample1	Human	normal human fibroblasts (NHFs from circumcised foreskin of a juvenile)	H_2O_2 treatment	The cells were treated in medium with H_2O_2 for 2 h and kept in medium without H_2O_2 for 48 h.	Arraystar Human LncRNA microarray V2.0
H_2O_2-induced model		GSE116761	GSM3260258	H_2O_2 sample2	Human	normal human fibroblasts (NHFs from	H_2O_2 treatment		Arraystar Human LncRNA microarray V2.0

					circumcised foreskin of a juvenile)			
H_2O_2-induced model	GSE116761	GSM3260259	H_2O_2 sample3	Human	normal human fibroblasts (NHFs from circumcised foreskin of a juvenile)	H_2O_2 treatment		Arraystar Human LncRNA microarray V2.0
H_2O_2-induced model	GSE116761	GSM3260260	control sample1	Human	normal human fibroblasts (NHFs from circumcised foreskin of a juvenile)	normal NHFs		Arraystar Human LncRNA microarray V2.0
H_2O_2-induced model	GSE116761	GSM3260261	control sample2	Human	normal human fibroblasts (NHFs from circumcised foreskin of a juvenile)	normal NHFs	NHFs was cultured in DMEM supplemented with 10% FBS (Gibco).	Arraystar Human LncRNA microarray V2.0
H_2O_2-induced model	GSE116761	GSM3260262	control sample3	Human	normal human fibroblasts (NHFs from circumcised foreskin of a juvenile)	normal NHFs		Arraystar Human LncRNA microarray V2.0
