# Supplementary material for: Histone deacetylase 4 reverses cellular senescence via DDIT4 in dermal fibroblasts
Source: Aging (Albany NY). 2022 Jun 9;14(11):4653–72. doi: 10.18632/aging.204118 (PMC9217707; doi:10.18632/aging.204118)
Supplement: Supplementary Table 3 [file aging-14-204118-s004.docx]

Supplementary Table 3. Transcriptome analysis of UV-induced senescence HDFs.

| **Entrez ID** | **Symbol** | **log_2_(*FC*) [UV / Sham]** | **Pt [UV / Sham]** | **Pf [UV / Sham]** | **DEGs [UV / Sham]** | **normalized UV1** | **normalized UV2** | **normalized Sham1** | **normalized Sham2** |
| --- | --- | --- | --- | --- | --- | --- | --- | --- | --- |
| 19 | ABCA1 | 0.355 | 0.008 | 0.025 | up-reg. | 9.693 | 9.744 | 9.351 | 9.377 |
| 20 | ABCA2 | 0.236 | 0.035 | 0.081 | up-reg. | 8.721 | 8.800 | 8.526 | 8.524 |
| 94 | ACVRL1 | 0.350 | 0.024 | 0.026 | up-reg. | 8.025 | 8.083 | 7.664 | 7.742 |
| 100 | ADA | 0.352 | 0.036 | 0.026 | up-reg. | 8.339 | 8.460 | 8.048 | 8.046 |
| 114 | ADCY8 | 0.299 | 0.014 | 0.043 | up-reg. | 5.965 | 6.019 | 5.709 | 5.676 |
| 165 | AEBP1 | 0.218 | 0.040 | 0.099 | up-reg. | 10.016 | 10.094 | 9.840 | 9.833 |
| 190 | NR0B1 | 0.286 | 0.026 | 0.049 | up-reg. | 5.715 | 5.797 | 5.472 | 5.468 |
| 273 | AMPH | 0.313 | 0.034 | 0.037 | up-reg. | 5.375 | 5.476 | 5.100 | 5.125 |
| 320 | APBA1 | 0.369 | 0.046 | 0.022 | up-reg. | 7.813 | 7.832 | 7.525 | 7.383 |
| 390 | RND3 | 0.276 | 0.037 | 0.054 | up-reg. | 11.989 | 12.065 | 11.780 | 11.722 |
| 427 | ASAH1 | 0.536 | 0.002 | 0.006 | up-reg. | 9.966 | 10.012 | 9.454 | 9.452 |
| 775 | CACNA1C | 0.402 | 0.012 | 0.016 | up-reg. | 7.559 | 7.595 | 7.208 | 7.141 |
| 796 | CALCA | 0.246 | 0.000 | 0.072 | up-reg. | 6.140 | 6.137 | 5.889 | 5.895 |
| 958 | CD40 | 0.222 | 0.016 | 0.095 | up-reg. | 6.782 | 6.763 | 6.574 | 6.528 |
| 968 | CD68 | 0.249 | 0.020 | 0.070 | up-reg. | 10.959 | 10.975 | 10.688 | 10.748 |
| 1003 | CDH5 | 0.320 | 0.004 | 0.034 | up-reg. | 5.416 | 5.439 | 5.123 | 5.093 |
| 1026 | CDKN1A | 0.670 | 0.003 | 0.003 | up-reg. | 9.660 | 9.709 | 9.039 | 8.989 |
| 1041 | CDSN | 0.348 | 0.005 | 0.027 | up-reg. | 7.751 | 7.743 | 7.376 | 7.421 |
| 1060 | CENPC | 0.301 | 0.043 | 0.042 | up-reg. | 8.465 | 8.533 | 8.243 | 8.153 |
| 1130 | LYST | 0.447 | 0.010 | 0.011 | up-reg. | 9.089 | 9.109 | 8.691 | 8.613 |
| 1135 | CHRNA2 | 0.309 | 0.002 | 0.038 | up-reg. | 6.470 | 6.489 | 6.178 | 6.162 |
| 1200 | TPP1 | 0.318 | 0.017 | 0.035 | up-reg. | 10.633 | 10.568 | 10.264 | 10.301 |
| 1293 | COL6A3 | 0.231 | 0.009 | 0.085 | up-reg. | 12.298 | 12.335 | 12.081 | 12.089 |
| 1294 | COL7A1 | 0.248 | 0.007 | 0.071 | up-reg. | 7.642 | 7.607 | 7.381 | 7.372 |
| 1357 | CPA1 | 0.328 | 0.024 | 0.032 | up-reg. | 6.291 | 6.349 | 5.957 | 6.026 |
| 1410 | CRYAB | 0.357 | 0.021 | 0.024 | up-reg. | 9.597 | 9.597 | 9.194 | 9.286 |
| 1509 | CTSD | 0.351 | 0.004 | 0.026 | up-reg. | 11.263 | 11.297 | 10.919 | 10.939 |
| 1513 | CTSK | 0.342 | 0.025 | 0.028 | up-reg. | 11.905 | 11.923 | 11.525 | 11.619 |
| 1540 | CYLD | 0.327 | 0.031 | 0.032 | up-reg. | 9.003 | 9.072 | 8.672 | 8.749 |
| 1543 | CYP1A1 | 0.224 | 0.045 | 0.093 | up-reg. | 5.362 | 5.283 | 5.083 | 5.115 |
| 1588 | CYP19A1 | 0.445 | 0.016 | 0.012 | up-reg. | 6.124 | 6.214 | 5.744 | 5.705 |
| 1805 | DPT | 0.304 | 0.023 | 0.040 | up-reg. | 6.160 | 6.241 | 5.901 | 5.892 |
| 1840 | DTX1 | 0.335 | 0.004 | 0.030 | up-reg. | 6.713 | 6.741 | 6.380 | 6.404 |
| 1909 | EDNRA | 0.474 | 0.015 | 0.009 | up-reg. | 10.063 | 10.129 | 9.662 | 9.583 |
| 1958 | EGR1 | 0.294 | 0.045 | 0.045 | up-reg. | 11.643 | 11.532 | 11.281 | 11.305 |
| 1990 | CELA1 | 0.273 | 0.000 | 0.055 | up-reg. | 5.425 | 5.431 | 5.158 | 5.151 |
| 2034 | EPAS1 | 0.485 | 0.002 | 0.009 | up-reg. | 11.027 | 11.057 | 10.570 | 10.544 |
| 2044 | EPHA5 | 0.258 | 0.008 | 0.064 | up-reg. | 5.289 | 5.259 | 5.001 | 5.030 |
| 2299 | FOXI1 | 0.280 | 0.004 | 0.051 | up-reg. | 7.119 | 7.131 | 6.829 | 6.860 |
| 2309 | FOXO3 | 0.250 | 0.024 | 0.070 | up-reg. | 8.874 | 8.944 | 8.657 | 8.661 |
| 2530 | FUT8 | 0.217 | 0.018 | 0.100 | up-reg. | 10.066 | 10.116 | 9.879 | 9.868 |
| 2570 | GABRR2 | 0.306 | 0.045 | 0.040 | up-reg. | 6.602 | 6.680 | 6.379 | 6.291 |
| 2629 | GBA | 0.371 | 0.034 | 0.022 | up-reg. | 9.056 | 9.144 | 8.773 | 8.685 |
| 2668 | GDNF | 0.423 | 0.001 | 0.014 | up-reg. | 7.316 | 7.332 | 6.903 | 6.898 |
| 2744 | GLS | 0.388 | 0.006 | 0.019 | up-reg. | 9.999 | 10.051 | 9.632 | 9.643 |
| 2817 | GPC1 | 0.249 | 0.037 | 0.070 | up-reg. | 9.767 | 9.711 | 9.457 | 9.522 |
| 2827 | GPR3 | 0.252 | 0.002 | 0.068 | up-reg. | 9.790 | 9.771 | 9.535 | 9.522 |
| 2845 | GPR22 | 0.355 | 0.044 | 0.025 | up-reg. | 4.253 | 4.234 | 3.822 | 3.956 |
| 2896 | GRN | 0.410 | 0.025 | 0.016 | up-reg. | 10.936 | 11.008 | 10.517 | 10.607 |
| 3195 | TLX1 | 0.274 | 0.046 | 0.055 | up-reg. | 6.450 | 6.535 | 6.186 | 6.251 |
| 3290 | HSD11B1 | 0.275 | 0.043 | 0.054 | up-reg. | 7.062 | 6.972 | 6.767 | 6.716 |
| 3371 | TNC | 0.429 | 0.000 | 0.013 | up-reg. | 11.445 | 11.452 | 11.014 | 11.025 |
| 3482 | IGF2R | 0.278 | 0.031 | 0.052 | up-reg. | 10.380 | 10.405 | 10.072 | 10.156 |
| 3568 | IL5RA | 0.246 | 0.010 | 0.072 | up-reg. | 4.699 | 4.725 | 4.448 | 4.483 |
| 3598 | IL13RA2 | 0.622 | 0.027 | 0.003 | up-reg. | 7.195 | 7.066 | 6.572 | 6.446 |
| 3631 | INPP4A | 0.221 | 0.020 | 0.096 | up-reg. | 8.510 | 8.564 | 8.320 | 8.311 |
| 3673 | ITGA2 | 0.396 | 0.016 | 0.017 | up-reg. | 11.954 | 12.031 | 11.618 | 11.575 |
| 3690 | ITGB3 | 0.722 | 0.012 | 0.002 | up-reg. | 10.217 | 10.350 | 9.582 | 9.540 |
| 3732 | CD82 | 0.331 | 0.025 | 0.031 | up-reg. | 9.463 | 9.444 | 9.077 | 9.169 |
| 3772 | KCNJ15 | 0.588 | 0.005 | 0.004 | up-reg. | 7.278 | 7.251 | 6.713 | 6.641 |
| 3854 | KRT6B | 0.327 | 0.047 | 0.033 | up-reg. | 6.154 | 6.055 | 5.736 | 5.819 |
| 3984 | LIMK1 | 0.268 | 0.044 | 0.058 | up-reg. | 10.040 | 10.127 | 9.842 | 9.789 |
| 4061 | LY6E | 0.297 | 0.019 | 0.044 | up-reg. | 10.929 | 10.860 | 10.610 | 10.586 |
| 4130 | MAP1A | 0.353 | 0.025 | 0.025 | up-reg. | 10.006 | 10.079 | 9.655 | 9.723 |
| 4193 | MDM2 | 0.272 | 0.040 | 0.055 | up-reg. | 11.248 | 11.168 | 10.906 | 10.964 |
| 4312 | MMP1 | 0.684 | 0.020 | 0.002 | up-reg. | 12.555 | 12.595 | 11.808 | 11.974 |
| 4313 | MMP2 | 0.445 | 0.001 | 0.012 | up-reg. | 11.786 | 11.802 | 11.357 | 11.342 |
| 4314 | MMP3 | 1.420 | 0.001 | 0.000 | up-reg. | 9.771 | 9.814 | 8.352 | 8.393 |
| 4319 | MMP10 | 0.238 | 0.041 | 0.079 | up-reg. | 5.494 | 5.408 | 5.208 | 5.219 |
| 4323 | MMP14 | 0.644 | 0.008 | 0.003 | up-reg. | 10.840 | 10.939 | 10.239 | 10.251 |
| 4711 | NDUFB5 | 0.228 | 0.047 | 0.088 | up-reg. | 10.536 | 10.456 | 10.248 | 10.288 |
| 4864 | NPC1 | 0.422 | 0.020 | 0.014 | up-reg. | 10.731 | 10.788 | 10.383 | 10.292 |
| 4987 | OPRL1 | 0.325 | 0.039 | 0.033 | up-reg. | 6.650 | 6.657 | 6.270 | 6.386 |
| 5045 | FURIN | 0.284 | 0.038 | 0.050 | up-reg. | 9.264 | 9.340 | 8.985 | 9.051 |
| 5168 | ENPP2 | 0.262 | 0.005 | 0.062 | up-reg. | 10.210 | 10.216 | 9.935 | 9.968 |
| 5218 | CDK14 | 0.292 | 0.035 | 0.046 | up-reg. | 9.473 | 9.396 | 9.173 | 9.112 |
| 5264 | PHYH | 0.512 | 0.032 | 0.007 | up-reg. | 8.358 | 8.320 | 7.907 | 7.746 |
| 5295 | PIK3R1 | 0.235 | 0.041 | 0.082 | up-reg. | 10.556 | 10.632 | 10.379 | 10.340 |
| 5296 | PIK3R2 | 0.272 | 0.032 | 0.056 | up-reg. | 9.525 | 9.506 | 9.286 | 9.201 |
| 5360 | PLTP | 0.377 | 0.030 | 0.021 | up-reg. | 9.621 | 9.738 | 9.300 | 9.304 |
| 5396 | PRRX1 | 0.247 | 0.028 | 0.071 | up-reg. | 11.909 | 11.940 | 11.711 | 11.644 |
| 5476 | CTSA | 0.241 | 0.013 | 0.076 | up-reg. | 10.486 | 10.531 | 10.276 | 10.259 |
| 5593 | PRKG2 | 0.684 | 0.017 | 0.002 | up-reg. | 8.024 | 7.933 | 7.361 | 7.230 |
| 5627 | PROS1 | 0.359 | 0.041 | 0.024 | up-reg. | 8.067 | 8.005 | 7.735 | 7.619 |
| 5646 | PRSS3 | 0.239 | 0.028 | 0.078 | up-reg. | 8.429 | 8.497 | 8.234 | 8.215 |
| 5783 | PTPN13 | 0.258 | 0.021 | 0.064 | up-reg. | 10.503 | 10.491 | 10.271 | 10.206 |
| 5793 | PTPRG | 0.250 | 0.004 | 0.070 | up-reg. | 9.751 | 9.722 | 9.491 | 9.482 |
| 5937 | RBMS1 | 0.280 | 0.024 | 0.052 | up-reg. | 10.444 | 10.502 | 10.220 | 10.167 |
| 5993 | RFX5 | 0.265 | 0.023 | 0.060 | up-reg. | 8.342 | 8.352 | 8.118 | 8.047 |
| 6103 | RPGR | 0.227 | 0.014 | 0.089 | up-reg. | 8.066 | 8.019 | 7.818 | 7.814 |
| 6303 | SAT1 | 0.440 | 0.032 | 0.012 | up-reg. | 9.516 | 9.653 | 9.127 | 9.162 |
| 6423 | SFRP2 | 0.252 | 0.009 | 0.068 | up-reg. | 8.061 | 8.086 | 7.805 | 7.838 |
| 6526 | SLC5A3 | 0.235 | 0.039 | 0.082 | up-reg. | 11.102 | 11.167 | 10.926 | 10.873 |
| 6608 | SMO | 0.237 | 0.008 | 0.080 | up-reg. | 6.990 | 6.958 | 6.745 | 6.728 |
| 6610 | SMPD2 | 0.246 | 0.031 | 0.072 | up-reg. | 8.229 | 8.300 | 8.003 | 8.034 |
| 6722 | SRF | 0.236 | 0.013 | 0.080 | up-reg. | 9.927 | 9.964 | 9.724 | 9.693 |
| 6953 | TCP10L3 | 0.252 | 0.009 | 0.068 | up-reg. | 5.721 | 5.701 | 5.441 | 5.477 |
| 7040 | TGFB1 | 0.262 | 0.042 | 0.061 | up-reg. | 9.317 | 9.408 | 9.082 | 9.119 |
| 7056 | THBD | 0.542 | 0.031 | 0.006 | up-reg. | 7.337 | 7.169 | 6.699 | 6.723 |
| 7058 | THBS2 | 0.290 | 0.028 | 0.047 | up-reg. | 10.882 | 10.945 | 10.593 | 10.653 |
| 7101 | NR2E1 | 0.326 | 0.015 | 0.033 | up-reg. | 5.492 | 5.504 | 5.206 | 5.137 |
| 7444 | VRK2 | 0.386 | 0.004 | 0.019 | up-reg. | 8.819 | 8.859 | 8.443 | 8.462 |
| 7508 | XPC | 0.244 | 0.001 | 0.074 | up-reg. | 8.164 | 8.167 | 7.914 | 7.929 |
| 7643 | ZNF90 | 0.362 | 0.039 | 0.023 | up-reg. | 6.755 | 6.638 | 6.308 | 6.362 |
| 8091 | HMGA2 | 0.333 | 0.021 | 0.031 | up-reg. | 11.591 | 11.600 | 11.305 | 11.220 |
| 8382 | NME5 | 0.276 | 0.019 | 0.053 | up-reg. | 5.276 | 5.222 | 4.993 | 4.953 |
| 8406 | SRPX | 0.299 | 0.044 | 0.043 | up-reg. | 11.392 | 11.444 | 11.068 | 11.170 |
| 8638 | OASL | 0.232 | 0.036 | 0.085 | up-reg. | 6.367 | 6.368 | 6.097 | 6.175 |
| 8663 | EIF3C | 0.246 | 0.045 | 0.072 | up-reg. | 9.132 | 9.042 | 8.854 | 8.827 |
| 8831 | SYNGAP1 | 0.252 | 0.031 | 0.068 | up-reg. | 7.770 | 7.781 | 7.484 | 7.562 |
| 8838 | CCN6 | 0.275 | 0.010 | 0.054 | up-reg. | 5.427 | 5.421 | 5.125 | 5.173 |
| 8877 | SPHK1 | 0.300 | 0.015 | 0.042 | up-reg. | 8.585 | 8.647 | 8.305 | 8.327 |
| 9120 | SLC16A6 | 0.810 | 0.027 | 0.001 | up-reg. | 8.721 | 8.695 | 8.015 | 7.781 |
| 9213 | XPR1 | 0.225 | 0.030 | 0.091 | up-reg. | 9.807 | 9.847 | 9.630 | 9.574 |
| 9472 | AKAP6 | 0.257 | 0.012 | 0.065 | up-reg. | 5.733 | 5.783 | 5.504 | 5.498 |
| 9518 | GDF15 | 0.539 | 0.009 | 0.006 | up-reg. | 10.171 | 10.238 | 9.634 | 9.697 |
| 9536 | PTGES | 0.253 | 0.037 | 0.067 | up-reg. | 8.288 | 8.287 | 8.078 | 7.990 |
| 9552 | SPAG7 | 0.260 | 0.033 | 0.063 | up-reg. | 8.698 | 8.706 | 8.484 | 8.399 |
| 9653 | HS2ST1 | 0.347 | 0.040 | 0.027 | up-reg. | 9.659 | 9.780 | 9.387 | 9.358 |
| 9725 | TMEM63A | 0.371 | 0.003 | 0.022 | up-reg. | 8.542 | 8.581 | 8.193 | 8.189 |
| 9859 | CEP170 | 0.297 | 0.036 | 0.044 | up-reg. | 11.528 | 11.628 | 11.289 | 11.274 |
| 9911 | TMCC2 | 0.426 | 0.022 | 0.013 | up-reg. | 6.925 | 6.820 | 6.467 | 6.425 |
| 10014 | HDAC5 | 0.273 | 0.005 | 0.055 | up-reg. | 8.463 | 8.475 | 8.181 | 8.212 |
| 10154 | PLXNC1 | 0.306 | 0.043 | 0.039 | up-reg. | 5.568 | 5.614 | 5.338 | 5.232 |
| 10158 | PDZK1IP1 | 0.268 | 0.046 | 0.058 | up-reg. | 6.667 | 6.757 | 6.418 | 6.471 |
| 10221 | TRIB1 | 0.236 | 0.007 | 0.081 | up-reg. | 9.055 | 9.083 | 8.842 | 8.824 |
| 10253 | SPRY2 | 0.240 | 0.047 | 0.077 | up-reg. | 10.399 | 10.441 | 10.137 | 10.222 |
| 10417 | SPON2 | 0.593 | 0.011 | 0.004 | up-reg. | 9.437 | 9.544 | 8.896 | 8.900 |
| 10423 | CDIPT | 0.302 | 0.016 | 0.041 | up-reg. | 9.871 | 9.934 | 9.613 | 9.588 |
| 10443 | N4BP2L2 | 0.258 | 0.004 | 0.064 | up-reg. | 9.343 | 9.362 | 9.105 | 9.083 |
| 10457 | GPNMB | 0.533 | 0.011 | 0.006 | up-reg. | 10.507 | 10.591 | 9.989 | 10.044 |
| 10493 | VAT1 | 0.263 | 0.024 | 0.061 | up-reg. | 11.518 | 11.568 | 11.254 | 11.306 |
| 10516 | FBLN5 | 0.236 | 0.047 | 0.081 | up-reg. | 11.940 | 11.900 | 11.726 | 11.643 |
| 10544 | PROCR | 0.311 | 0.037 | 0.038 | up-reg. | 9.725 | 9.650 | 9.338 | 9.416 |
| 10577 | NPC2 | 0.249 | 0.004 | 0.070 | up-reg. | 12.159 | 12.182 | 11.913 | 11.930 |
| 10900 | RUNDC3A | 0.240 | 0.035 | 0.077 | up-reg. | 6.059 | 6.056 | 5.858 | 5.777 |
| 10901 | DHRS4 | 0.818 | 0.017 | 0.001 | up-reg. | 7.423 | 7.312 | 6.624 | 6.474 |
| 10953 | TOMM34 | 0.227 | 0.045 | 0.089 | up-reg. | 10.733 | 10.814 | 10.529 | 10.563 |
| 11031 | RAB31 | 0.415 | 0.007 | 0.015 | up-reg. | 10.350 | 10.387 | 9.978 | 9.929 |
| 11043 | MID2 | 0.303 | 0.012 | 0.041 | up-reg. | 8.533 | 8.579 | 8.235 | 8.270 |
| 11189 | CELF3 | 0.267 | 0.047 | 0.059 | up-reg. | 6.895 | 6.847 | 6.557 | 6.652 |
| 11226 | GALNT6 | 0.327 | 0.009 | 0.032 | up-reg. | 8.155 | 8.158 | 7.857 | 7.802 |
| 11253 | MAN1B1 | 0.239 | 0.041 | 0.078 | up-reg. | 9.213 | 9.201 | 9.011 | 8.925 |
| 22808 | MRAS | 0.448 | 0.008 | 0.011 | up-reg. | 7.622 | 7.643 | 7.151 | 7.219 |
| 22993 | HMGXB3 | 0.253 | 0.020 | 0.067 | up-reg. | 9.816 | 9.879 | 9.594 | 9.594 |
| 23012 | STK38L | 0.238 | 0.006 | 0.079 | up-reg. | 9.018 | 9.046 | 8.802 | 8.785 |
| 23077 | MYCBP2 | 0.235 | 0.049 | 0.082 | up-reg. | 10.031 | 10.039 | 9.847 | 9.753 |
| 23233 | EXOC6B | 0.232 | 0.026 | 0.084 | up-reg. | 10.616 | 10.553 | 10.343 | 10.363 |
| 23266 | ADGRL2 | 0.230 | 0.049 | 0.087 | up-reg. | 8.262 | 8.324 | 8.098 | 8.029 |
| 23428 | SLC7A8 | 0.837 | 0.010 | 0.001 | up-reg. | 7.669 | 7.809 | 6.929 | 6.876 |
| 23533 | PIK3R5 | 0.223 | 0.041 | 0.094 | up-reg. | 6.425 | 6.442 | 6.171 | 6.251 |
| 23539 | SLC16A8 | 0.225 | 0.022 | 0.091 | up-reg. | 7.398 | 7.352 | 7.168 | 7.131 |
| 25809 | TTLL1 | 0.227 | 0.016 | 0.089 | up-reg. | 8.166 | 8.191 | 7.928 | 7.973 |
| 25960 | ADGRA2 | 0.218 | 0.040 | 0.099 | up-reg. | 10.038 | 10.116 | 9.854 | 9.864 |
| 26012 | NSMF | 0.291 | 0.005 | 0.046 | up-reg. | 9.446 | 9.481 | 9.164 | 9.180 |
| 26020 | LRP10 | 0.336 | 0.014 | 0.029 | up-reg. | 9.995 | 9.934 | 9.610 | 9.646 |
| 26231 | LRRC29 | 0.259 | 0.004 | 0.064 | up-reg. | 7.276 | 7.305 | 7.027 | 7.035 |
| 26275 | HIBCH | 0.283 | 0.034 | 0.050 | up-reg. | 7.912 | 7.821 | 7.594 | 7.574 |
| 26301 | GBGT1 | 0.296 | 0.006 | 0.044 | up-reg. | 7.482 | 7.511 | 7.187 | 7.214 |
| 26750 | RPS6KC1 | 0.242 | 0.008 | 0.076 | up-reg. | 8.637 | 8.651 | 8.420 | 8.384 |
| 27132 | CPNE7 | 0.254 | 0.044 | 0.067 | up-reg. | 8.681 | 8.721 | 8.404 | 8.491 |
| 27237 | ARHGEF16 | 0.224 | 0.003 | 0.093 | up-reg. | 6.577 | 6.595 | 6.368 | 6.357 |
| 27244 | SESN1 | 0.471 | 0.002 | 0.009 | up-reg. | 8.263 | 8.231 | 7.785 | 7.767 |
| 28996 | HIPK2 | 0.226 | 0.011 | 0.090 | up-reg. | 10.532 | 10.574 | 10.323 | 10.332 |
| 29070 | CCDC113 | 0.319 | 0.009 | 0.035 | up-reg. | 6.717 | 6.715 | 6.370 | 6.424 |
| 29800 | ZDHHC1 | 0.348 | 0.032 | 0.027 | up-reg. | 7.701 | 7.790 | 7.363 | 7.432 |
| 30845 | EHD3 | 0.253 | 0.041 | 0.067 | up-reg. | 10.924 | 10.898 | 10.613 | 10.702 |
| 50632 | CALY | 0.277 | 0.021 | 0.053 | up-reg. | 7.959 | 7.941 | 7.639 | 7.708 |
| 51088 | KLHL5 | 0.477 | 0.040 | 0.009 | up-reg. | 9.341 | 9.510 | 8.961 | 8.936 |
| 51257 | MARCHF2 | 0.393 | 0.044 | 0.018 | up-reg. | 8.118 | 8.069 | 7.629 | 7.772 |
| 51351 | ZNF117 | 0.526 | 0.036 | 0.007 | up-reg. | 7.269 | 7.447 | 6.848 | 6.816 |
| 51635 | DHRS7 | 0.296 | 0.045 | 0.044 | up-reg. | 8.823 | 8.828 | 8.473 | 8.586 |
| 51715 | RAB23 | 0.293 | 0.007 | 0.046 | up-reg. | 9.801 | 9.842 | 9.527 | 9.530 |
| 51744 | CD244 | 0.321 | 0.047 | 0.034 | up-reg. | 5.174 | 5.209 | 4.931 | 4.810 |
| 55057 | CRYBG2 | 0.240 | 0.020 | 0.077 | up-reg. | 6.504 | 6.492 | 6.288 | 6.228 |
| 55062 | WIPI1 | 0.234 | 0.043 | 0.083 | up-reg. | 11.010 | 11.074 | 10.838 | 10.779 |
| 55117 | SLC6A15 | 0.558 | 0.003 | 0.005 | up-reg. | 11.209 | 11.183 | 10.663 | 10.612 |
| 55151 | TMEM38B | 0.442 | 0.027 | 0.012 | up-reg. | 8.969 | 9.035 | 8.615 | 8.505 |
| 55283 | MCOLN3 | 0.559 | 0.037 | 0.005 | up-reg. | 6.766 | 6.900 | 6.204 | 6.344 |
| 55296 | TBC1D19 | 0.298 | 0.036 | 0.043 | up-reg. | 9.731 | 9.825 | 9.500 | 9.459 |
| 55609 | ZNF280C | 0.304 | 0.047 | 0.041 | up-reg. | 9.959 | 9.898 | 9.677 | 9.573 |
| 55728 | N4BP2 | 0.807 | 0.040 | 0.001 | up-reg. | 6.520 | 6.622 | 5.900 | 5.629 |
| 56107 | PCDHGA9 | 0.381 | 0.018 | 0.020 | up-reg. | 8.982 | 9.036 | 8.665 | 8.592 |
| 56122 | PCDHB14 | 0.390 | 0.006 | 0.018 | up-reg. | 5.133 | 5.180 | 4.777 | 4.756 |
| 56907 | SPIRE1 | 0.267 | 0.030 | 0.058 | up-reg. | 9.051 | 9.028 | 8.733 | 8.812 |
| 57175 | CORO1B | 0.482 | 0.035 | 0.009 | up-reg. | 10.093 | 10.183 | 9.589 | 9.723 |
| 57406 | ABHD6 | 0.326 | 0.015 | 0.033 | up-reg. | 7.980 | 7.921 | 7.645 | 7.603 |
| 57541 | ZNF398 | 0.233 | 0.046 | 0.083 | up-reg. | 8.120 | 8.199 | 7.949 | 7.903 |
| 57655 | GRAMD1A | 0.225 | 0.002 | 0.091 | up-reg. | 9.322 | 9.302 | 9.088 | 9.088 |
| 57722 | IGDCC4 | 0.286 | 0.012 | 0.048 | up-reg. | 6.437 | 6.414 | 6.114 | 6.165 |
| 57830 | KRTAP5-8 | 0.488 | 0.026 | 0.009 | up-reg. | 8.046 | 7.906 | 7.491 | 7.484 |
| 58530 | LY6G6D | 0.494 | 0.007 | 0.008 | up-reg. | 6.754 | 6.750 | 6.293 | 6.222 |
| 58986 | PGAP6 | 0.315 | 0.021 | 0.036 | up-reg. | 10.077 | 10.132 | 9.759 | 9.820 |
| 60468 | BACH2 | 0.320 | 0.028 | 0.035 | up-reg. | 6.239 | 6.197 | 5.941 | 5.856 |
| 63027 | SLC22A23 | 0.430 | 0.029 | 0.013 | up-reg. | 6.901 | 6.769 | 6.406 | 6.405 |
| 64073 | C19orf33 | 0.273 | 0.025 | 0.055 | up-reg. | 8.116 | 8.164 | 7.897 | 7.836 |
| 64344 | HIF3A | 0.219 | 0.031 | 0.098 | up-reg. | 8.404 | 8.458 | 8.233 | 8.191 |
| 64699 | TMPRSS3 | 0.271 | 0.046 | 0.056 | up-reg. | 5.145 | 5.160 | 4.934 | 4.829 |
| 64840 | PORCN | 0.328 | 0.012 | 0.032 | up-reg. | 9.480 | 9.541 | 9.192 | 9.172 |
| 64866 | CDCP1 | 0.229 | 0.029 | 0.087 | up-reg. | 9.655 | 9.685 | 9.472 | 9.409 |
| 65997 | RASL11B | 0.294 | 0.002 | 0.045 | up-reg. | 5.719 | 5.716 | 5.435 | 5.411 |
| 79364 | ZXDC | 0.238 | 0.005 | 0.079 | up-reg. | 8.396 | 8.423 | 8.178 | 8.165 |
| 79544 | OR4K1 | 0.260 | 0.019 | 0.063 | up-reg. | 4.815 | 4.764 | 4.510 | 4.549 |
| 79586 | CHPF | 0.258 | 0.021 | 0.064 | up-reg. | 9.770 | 9.726 | 9.465 | 9.515 |
| 79611 | ACSS3 | 0.234 | 0.001 | 0.083 | up-reg. | 7.813 | 7.810 | 7.572 | 7.581 |
| 79772 | MCTP1 | 0.770 | 0.037 | 0.001 | up-reg. | 7.231 | 7.154 | 6.551 | 6.294 |
| 79783 | SUGCT | 0.477 | 0.049 | 0.009 | up-reg. | 7.884 | 7.854 | 7.487 | 7.297 |
| 79839 | CCDC102B | 1.010 | 0.014 | 0.000 | up-reg. | 7.393 | 7.491 | 6.525 | 6.338 |
| 79963 | ABCA11P | 1.040 | 0.030 | 0.000 | up-reg. | 5.568 | 5.316 | 4.502 | 4.303 |
| 79971 | WLS | 0.293 | 0.006 | 0.045 | up-reg. | 11.930 | 11.917 | 11.650 | 11.612 |
| 80055 | PGAP1 | 0.601 | 0.031 | 0.004 | up-reg. | 8.724 | 8.595 | 8.128 | 7.989 |
| 80144 | FRAS1 | 0.587 | 0.002 | 0.004 | up-reg. | 7.622 | 7.576 | 7.012 | 7.012 |
| 80267 | EDEM3 | 0.365 | 0.038 | 0.023 | up-reg. | 10.419 | 10.498 | 10.144 | 10.043 |
| 80313 | LRRC27 | 0.308 | 0.038 | 0.039 | up-reg. | 7.120 | 7.166 | 6.786 | 6.884 |
| 81793 | TLR10 | 0.234 | 0.043 | 0.083 | up-reg. | 4.670 | 4.596 | 4.376 | 4.422 |
| 81847 | RNF146 | 0.235 | 0.032 | 0.082 | up-reg. | 8.564 | 8.640 | 8.366 | 8.368 |
| 81848 | SPRY4 | 0.224 | 0.003 | 0.092 | up-reg. | 10.427 | 10.450 | 10.212 | 10.216 |
| 83868 | TTTY13 | 0.220 | 0.011 | 0.097 | up-reg. | 5.640 | 5.634 | 5.397 | 5.437 |
| 84168 | ANTXR1 | 0.293 | 0.001 | 0.046 | up-reg. | 11.124 | 11.120 | 10.835 | 10.825 |
| 84264 | HAGHL | 0.250 | 0.017 | 0.070 | up-reg. | 7.771 | 7.715 | 7.488 | 7.499 |
| 84527 | ZNF559 | 0.290 | 0.042 | 0.047 | up-reg. | 7.132 | 7.103 | 6.777 | 6.880 |
| 84542 | KIAA1841 | 0.379 | 0.049 | 0.020 | up-reg. | 7.058 | 7.123 | 6.643 | 6.781 |
| 84925 | SLC49A4 | 0.290 | 0.042 | 0.047 | up-reg. | 10.115 | 10.196 | 9.901 | 9.830 |
| 89978 | DPH6 | 0.231 | 0.023 | 0.085 | up-reg. | 9.209 | 9.218 | 9.013 | 8.952 |
| 90865 | IL33 | 0.529 | 0.017 | 0.006 | up-reg. | 5.940 | 6.003 | 5.493 | 5.391 |
| 91179 | SCARF2 | 0.235 | 0.001 | 0.082 | up-reg. | 8.717 | 8.705 | 8.476 | 8.476 |
| 91768 | CABLES1 | 0.466 | 0.026 | 0.010 | up-reg. | 6.101 | 6.229 | 5.721 | 5.677 |
| 93099 | DMKN | 0.422 | 0.032 | 0.014 | up-reg. | 6.903 | 6.989 | 6.576 | 6.472 |
| 94234 | FOXQ1 | 0.509 | 0.030 | 0.008 | up-reg. | 9.673 | 9.711 | 9.259 | 9.107 |
| 112744 | IL17F | 0.266 | 0.025 | 0.059 | up-reg. | 5.528 | 5.557 | 5.311 | 5.242 |
| 112770 | GLMP | 0.309 | 0.029 | 0.038 | up-reg. | 10.132 | 10.224 | 9.862 | 9.876 |
| 113451 | AZIN2 | 0.343 | 0.006 | 0.028 | up-reg. | 7.012 | 6.985 | 6.676 | 6.636 |
| 113655 | MFSD3 | 0.309 | 0.002 | 0.039 | up-reg. | 8.592 | 8.615 | 8.294 | 8.296 |
| 114757 | CYGB | 0.445 | 0.014 | 0.011 | up-reg. | 9.745 | 9.824 | 9.316 | 9.363 |
| 114800 | CCDC85A | 0.306 | 0.046 | 0.040 | up-reg. | 5.803 | 5.745 | 5.520 | 5.416 |
| 114824 | PNMA5 | 0.494 | 0.033 | 0.008 | up-reg. | 6.517 | 6.572 | 6.127 | 5.974 |
| 115584 | SLC5A11 | 0.245 | 0.032 | 0.073 | up-reg. | 5.989 | 5.915 | 5.721 | 5.692 |
| 116236 | ABHD15 | 0.371 | 0.047 | 0.022 | up-reg. | 7.574 | 7.468 | 7.202 | 7.099 |
| 124274 | GPR139 | 0.334 | 0.025 | 0.030 | up-reg. | 8.179 | 8.231 | 7.910 | 7.832 |
| 124411 | ZNF720 | 0.476 | 0.000 | 0.009 | up-reg. | 8.579 | 8.568 | 8.097 | 8.097 |
| 125111 | GJD3 | 0.613 | 0.021 | 0.004 | up-reg. | 6.572 | 6.422 | 5.907 | 5.861 |
| 126123 | IZUMO2 | 0.341 | 0.026 | 0.028 | up-reg. | 5.765 | 5.810 | 5.491 | 5.404 |
| 129303 | TMEM150A | 0.303 | 0.044 | 0.041 | up-reg. | 8.120 | 8.114 | 7.871 | 7.757 |
| 129607 | CMPK2 | 0.295 | 0.003 | 0.044 | up-reg. | 6.030 | 6.018 | 5.714 | 5.744 |
| 132203 | SNTN | 0.229 | 0.024 | 0.087 | up-reg. | 4.043 | 4.050 | 3.849 | 3.786 |
| 132864 | CPEB2 | 0.399 | 0.048 | 0.017 | up-reg. | 10.343 | 10.419 | 10.053 | 9.912 |
| 134957 | STXBP5 | 0.508 | 0.041 | 0.008 | up-reg. | 10.460 | 10.567 | 10.082 | 9.929 |
| 135228 | CD109 | 0.342 | 0.022 | 0.028 | up-reg. | 10.891 | 10.825 | 10.485 | 10.547 |
| 136242 | PRSS37 | 0.386 | 0.038 | 0.019 | up-reg. | 5.133 | 5.065 | 4.655 | 4.772 |
| 138882 | OR1N2 | 0.308 | 0.033 | 0.039 | up-reg. | 5.603 | 5.702 | 5.349 | 5.340 |
| 140606 | SELENOM | 0.289 | 0.003 | 0.047 | up-reg. | 10.940 | 10.928 | 10.630 | 10.660 |
| 140862 | ISM1 | 0.274 | 0.028 | 0.055 | up-reg. | 7.694 | 7.744 | 7.414 | 7.477 |
| 146325 | PRR35 | 0.289 | 0.047 | 0.047 | up-reg. | 7.844 | 7.847 | 7.499 | 7.613 |
| 146894 | CD300LG | 0.268 | 0.021 | 0.058 | up-reg. | 7.536 | 7.529 | 7.230 | 7.299 |
| 147906 | DACT3 | 0.354 | 0.016 | 0.025 | up-reg. | 7.815 | 7.816 | 7.422 | 7.501 |
| 149111 | CNIH3 | 0.318 | 0.028 | 0.035 | up-reg. | 10.120 | 10.149 | 9.771 | 9.861 |
| 150165 | XKR3 | 0.356 | 0.033 | 0.024 | up-reg. | 4.350 | 4.247 | 3.969 | 3.914 |
| 151636 | DTX3L | 0.261 | 0.003 | 0.062 | up-reg. | 8.375 | 8.403 | 8.131 | 8.125 |
| 153222 | CREBRF | 0.279 | 0.002 | 0.052 | up-reg. | 8.309 | 8.296 | 8.033 | 8.014 |
| 157285 | PRAG1 | 0.231 | 0.040 | 0.085 | up-reg. | 8.148 | 8.080 | 7.907 | 7.859 |
| 157506 | RDH10 | 0.353 | 0.028 | 0.025 | up-reg. | 7.703 | 7.793 | 7.424 | 7.367 |
| 164284 | APCDD1L | 0.344 | 0.005 | 0.028 | up-reg. | 8.513 | 8.556 | 8.192 | 8.189 |
| 164668 | APOBEC3H | 0.438 | 0.015 | 0.012 | up-reg. | 5.441 | 5.517 | 5.013 | 5.069 |
| 165140 | OXER1 | 0.437 | 0.035 | 0.012 | up-reg. | 6.977 | 7.006 | 6.483 | 6.627 |
| 170589 | GPHA2 | 0.277 | 0.005 | 0.053 | up-reg. | 7.055 | 7.021 | 6.767 | 6.756 |
| 219348 | PLAC9 | 0.559 | 0.037 | 0.005 | up-reg. | 7.361 | 7.548 | 6.921 | 6.870 |
| 219437 | OR5L1 | 0.402 | 0.030 | 0.017 | up-reg. | 4.756 | 4.753 | 4.291 | 4.414 |
| 220323 | OAF | 0.227 | 0.044 | 0.089 | up-reg. | 9.209 | 9.286 | 9.002 | 9.040 |
| 220441 | RNF152 | 0.273 | 0.023 | 0.055 | up-reg. | 9.775 | 9.743 | 9.519 | 9.453 |
| 220963 | SLC16A9 | 0.223 | 0.014 | 0.093 | up-reg. | 4.806 | 4.850 | 4.596 | 4.613 |
| 221078 | NSUN6 | 0.271 | 0.007 | 0.056 | up-reg. | 8.385 | 8.389 | 8.136 | 8.097 |
| 221656 | KDM1B | 0.261 | 0.014 | 0.062 | up-reg. | 8.932 | 8.977 | 8.709 | 8.678 |
| 252983 | STXBP4 | 0.353 | 0.028 | 0.025 | up-reg. | 8.741 | 8.712 | 8.425 | 8.323 |
| 253959 | RALGAPA1 | 0.251 | 0.033 | 0.069 | up-reg. | 8.886 | 8.925 | 8.618 | 8.690 |
| 256471 | MFSD8 | 0.238 | 0.034 | 0.079 | up-reg. | 9.107 | 9.101 | 8.906 | 8.827 |
| 266722 | HS6ST3 | 0.325 | 0.041 | 0.033 | up-reg. | 5.753 | 5.769 | 5.378 | 5.495 |
| 282679 | AQP11 | 0.423 | 0.031 | 0.014 | up-reg. | 6.346 | 6.464 | 5.950 | 6.013 |
| 283209 | PGM2L1 | 0.507 | 0.019 | 0.008 | up-reg. | 7.453 | 7.458 | 7.010 | 6.888 |
| 283777 | FAM169B | 0.381 | 0.024 | 0.020 | up-reg. | 5.728 | 5.808 | 5.421 | 5.354 |
| 285590 | SH3PXD2B | 0.311 | 0.013 | 0.038 | up-reg. | 9.671 | 9.688 | 9.339 | 9.398 |
| 338328 | GPIHBP1 | 0.387 | 0.045 | 0.019 | up-reg. | 7.132 | 7.110 | 6.808 | 6.661 |
| 338755 | OR2AG2 | 0.415 | 0.029 | 0.015 | up-reg. | 6.148 | 6.025 | 5.656 | 5.686 |
| 339983 | NAT8L | 0.279 | 0.016 | 0.052 | up-reg. | 7.204 | 7.264 | 6.962 | 6.948 |
| 340152 | ZC3H12D | 0.251 | 0.022 | 0.069 | up-reg. | 7.145 | 7.094 | 6.890 | 6.847 |
| 340595 | RTL4 | 0.259 | 0.035 | 0.063 | up-reg. | 6.634 | 6.677 | 6.358 | 6.434 |
| 345222 | MSANTD1 | 0.359 | 0.033 | 0.024 | up-reg. | 6.630 | 6.516 | 6.225 | 6.202 |
| 353144 | LCE3C | 0.243 | 0.004 | 0.075 | up-reg. | 6.770 | 6.788 | 6.525 | 6.548 |
| 387338 | NSUN4 | 0.221 | 0.017 | 0.096 | up-reg. | 9.113 | 9.062 | 8.865 | 8.869 |
| 388121 | TNFAIP8L3 | 0.394 | 0.001 | 0.018 | up-reg. | 8.380 | 8.366 | 7.969 | 7.989 |
| 388946 | TMEM247 | 0.310 | 0.041 | 0.038 | up-reg. | 6.429 | 6.316 | 6.071 | 6.055 |
| 388951 | TSPYL6 | 0.236 | 0.012 | 0.081 | up-reg. | 6.632 | 6.587 | 6.369 | 6.378 |
| 399665 | FAM102A | 0.349 | 0.004 | 0.026 | up-reg. | 8.765 | 8.752 | 8.390 | 8.428 |
| 400986 | ANKRD36C | 0.416 | 0.019 | 0.015 | up-reg. | 8.600 | 8.527 | 8.184 | 8.111 |
| 406957 | MIR181C | 0.293 | 0.037 | 0.045 | up-reg. | 7.053 | 6.982 | 6.688 | 6.760 |
| 440248 | HERC2P9 | 0.550 | 0.043 | 0.005 | up-reg. | 7.497 | 7.398 | 6.988 | 6.807 |
| 441061 | MARCHF11 | 0.220 | 0.045 | 0.097 | up-reg. | 4.735 | 4.792 | 4.575 | 4.512 |
| 442213 | PTCHD4 | 0.427 | 0.030 | 0.013 | up-reg. | 7.838 | 7.930 | 7.504 | 7.409 |
| 554202 | MIR31HG | 0.359 | 0.012 | 0.024 | up-reg. | 9.690 | 9.761 | 9.370 | 9.364 |
| 619556 | MIR455 | 0.320 | 0.049 | 0.035 | up-reg. | 4.527 | 4.640 | 4.234 | 4.294 |
| 642987 | TMEM232 | 0.363 | 0.022 | 0.023 | up-reg. | 6.090 | 6.019 | 5.723 | 5.660 |
| 645843 | TMEM14EP | 1.117 | 0.014 | 0.000 | up-reg. | 6.994 | 7.206 | 6.036 | 5.930 |
| 677769 | SNHG22 | 0.263 | 0.038 | 0.061 | up-reg. | 9.674 | 9.726 | 9.475 | 9.399 |
| 727957 | MROH1 | 0.310 | 0.002 | 0.038 | up-reg. | 8.376 | 8.381 | 8.083 | 8.055 |
| 728621 | CCDC30 | 0.244 | 0.046 | 0.074 | up-reg. | 4.720 | 4.781 | 4.543 | 4.470 |
| 100130967 | C6orf99 | 0.600 | 0.037 | 0.004 | up-reg. | 6.260 | 6.207 | 5.533 | 5.733 |
| 100506271 | Z94160.1 | 0.235 | 0.036 | 0.082 | up-reg. | 6.762 | 6.691 | 6.511 | 6.472 |
| 100506658 | OCLN | 0.385 | 0.023 | 0.019 | up-reg. | 6.311 | 6.208 | 5.880 | 5.869 |
| 100507388 | PARM1-AS1 | 0.359 | 0.021 | 0.024 | up-reg. | 3.987 | 3.904 | 3.607 | 3.566 |
| 102800317 | TPTEP2-CSNK1E | 0.633 | 0.026 | 0.003 | up-reg. | 6.037 | 5.975 | 5.286 | 5.459 |
| 110599564 | EEF1AKMT4 | 0.233 | 0.030 | 0.084 | up-reg. | 8.550 | 8.620 | 8.362 | 8.342 |
| 133 | ADM | -0.283 | 0.034 | 0.050 | down-reg. | 10.967 | 10.957 | 11.291 | 11.199 |
| 157 | GRK3 | -0.278 | 0.017 | 0.052 | down-reg. | 6.018 | 5.955 | 6.264 | 6.265 |
| 213 | ALB | -0.252 | 0.034 | 0.068 | down-reg. | 4.644 | 4.601 | 4.839 | 4.909 |
| 216 | ALDH1A1 | -0.762 | 0.037 | 0.001 | down-reg. | 8.451 | 8.363 | 9.292 | 9.044 |
| 306 | ANXA3 | -0.471 | 0.025 | 0.009 | down-reg. | 6.415 | 6.284 | 6.816 | 6.825 |
| 309 | ANXA6 | -0.229 | 0.001 | 0.087 | down-reg. | 12.081 | 12.089 | 12.311 | 12.318 |
| 332 | BIRC5 | -0.368 | 0.036 | 0.022 | down-reg. | 8.395 | 8.344 | 8.794 | 8.682 |
| 409 | ARRB2 | -0.319 | 0.026 | 0.035 | down-reg. | 8.367 | 8.315 | 8.623 | 8.698 |
| 539 | ATP5PO | -0.219 | 0.011 | 0.098 | down-reg. | 10.012 | 10.020 | 10.255 | 10.215 |
| 641 | BLM | -0.357 | 0.022 | 0.024 | down-reg. | 8.966 | 8.875 | 9.289 | 9.266 |
| 652 | BMP4 | -0.532 | 0.024 | 0.006 | down-reg. | 7.736 | 7.644 | 8.279 | 8.166 |
| 664 | BNIP3 | -0.265 | 0.009 | 0.060 | down-reg. | 12.318 | 12.274 | 12.558 | 12.562 |
| 680 | BRS3 | -0.363 | 0.041 | 0.023 | down-reg. | 4.308 | 4.428 | 4.756 | 4.705 |
| 686 | BTD | -0.280 | 0.002 | 0.052 | down-reg. | 8.055 | 8.071 | 8.333 | 8.352 |
| 701 | BUB1B | -0.240 | 0.030 | 0.077 | down-reg. | 9.798 | 9.725 | 10.008 | 9.994 |
| 768 | CA9 | -0.576 | 0.006 | 0.004 | down-reg. | 7.346 | 7.276 | 7.901 | 7.871 |
| 978 | CDA | -0.416 | 0.021 | 0.014 | down-reg. | 8.173 | 8.144 | 8.523 | 8.627 |
| 990 | CDC6 | -0.297 | 0.026 | 0.043 | down-reg. | 9.281 | 9.343 | 9.637 | 9.580 |
| 991 | CDC20 | -0.402 | 0.019 | 0.016 | down-reg. | 9.914 | 9.823 | 10.252 | 10.289 |
| 993 | CDC25A | -0.388 | 0.011 | 0.019 | down-reg. | 7.344 | 7.277 | 7.684 | 7.711 |
| 995 | CDC25C | -0.534 | 0.042 | 0.006 | down-reg. | 7.764 | 7.577 | 8.233 | 8.176 |
| 1000 | CDH2 | -0.322 | 0.011 | 0.034 | down-reg. | 9.971 | 9.999 | 10.333 | 10.282 |
| 1002 | CDH4 | -0.599 | 0.002 | 0.004 | down-reg. | 7.428 | 7.383 | 8.009 | 8.002 |
| 1017 | CDK2 | -0.237 | 0.034 | 0.080 | down-reg. | 8.986 | 9.050 | 9.233 | 9.278 |
| 1075 | CTSC | -0.295 | 0.030 | 0.044 | down-reg. | 10.329 | 10.413 | 10.649 | 10.683 |
| 1163 | CKS1B | -0.270 | 0.043 | 0.057 | down-reg. | 10.396 | 10.466 | 10.738 | 10.665 |
| 1198 | CLK3 | -0.252 | 0.017 | 0.068 | down-reg. | 9.533 | 9.586 | 9.802 | 9.821 |
| 1359 | CPA3 | -0.445 | 0.013 | 0.011 | down-reg. | 5.137 | 5.214 | 5.645 | 5.597 |
| 1491 | CTH | -0.272 | 0.033 | 0.056 | down-reg. | 6.819 | 6.742 | 7.031 | 7.073 |
| 1741 | DLG3 | -0.237 | 0.043 | 0.080 | down-reg. | 6.681 | 6.652 | 6.861 | 6.944 |
| 1749 | DLX5 | -0.333 | 0.016 | 0.031 | down-reg. | 5.560 | 5.487 | 5.852 | 5.861 |
| 1786 | DNMT1 | -0.270 | 0.038 | 0.056 | down-reg. | 10.219 | 10.131 | 10.462 | 10.429 |
| 1794 | DOCK2 | -0.472 | 0.009 | 0.009 | down-reg. | 6.413 | 6.343 | 6.833 | 6.865 |
| 1809 | DPYSL3 | -0.275 | 0.014 | 0.054 | down-reg. | 9.288 | 9.296 | 9.539 | 9.596 |
| 1841 | DTYMK | -0.366 | 0.018 | 0.022 | down-reg. | 9.697 | 9.779 | 10.115 | 10.093 |
| 1850 | DUSP8 | -0.311 | 0.040 | 0.038 | down-reg. | 6.311 | 6.364 | 6.698 | 6.599 |
| 1854 | DUT | -0.380 | 0.004 | 0.020 | down-reg. | 9.028 | 9.060 | 9.410 | 9.438 |
| 1906 | EDN1 | -1.001 | 0.016 | 0.000 | down-reg. | 6.152 | 5.937 | 7.025 | 7.065 |
| 2146 | EZH2 | -0.307 | 0.028 | 0.039 | down-reg. | 9.054 | 8.985 | 9.355 | 9.297 |
| 2177 | FANCD2 | -0.420 | 0.012 | 0.014 | down-reg. | 8.302 | 8.225 | 8.673 | 8.693 |
| 2201 | FBN2 | -0.410 | 0.031 | 0.015 | down-reg. | 10.816 | 10.754 | 11.250 | 11.138 |
| 2202 | EFEMP1 | -0.417 | 0.038 | 0.014 | down-reg. | 8.959 | 8.861 | 9.381 | 9.273 |
| 2246 | FGF1 | -0.556 | 0.039 | 0.005 | down-reg. | 6.833 | 6.996 | 7.416 | 7.525 |
| 2256 | FGF11 | -0.378 | 0.040 | 0.020 | down-reg. | 6.533 | 6.600 | 6.885 | 7.003 |
| 2273 | FHL1 | -0.239 | 0.042 | 0.078 | down-reg. | 9.200 | 9.114 | 9.389 | 9.402 |
| 2305 | FOXM1 | -0.269 | 0.008 | 0.057 | down-reg. | 9.919 | 9.926 | 10.170 | 10.213 |
| 2326 | FMO1 | -0.273 | 0.013 | 0.055 | down-reg. | 3.856 | 3.823 | 4.135 | 4.091 |
| 2487 | FRZB | -0.333 | 0.047 | 0.031 | down-reg. | 4.942 | 5.072 | 5.334 | 5.344 |
| 2650 | GCNT1 | -0.281 | 0.004 | 0.051 | down-reg. | 10.086 | 10.057 | 10.359 | 10.346 |
| 2688 | GH1 | -0.465 | 0.025 | 0.010 | down-reg. | 6.071 | 5.939 | 6.474 | 6.466 |
| 2707 | GJB3 | -0.293 | 0.043 | 0.045 | down-reg. | 5.953 | 5.981 | 6.312 | 6.207 |
| 2747 | GLUD2 | -0.254 | 0.048 | 0.067 | down-reg. | 5.561 | 5.486 | 5.745 | 5.810 |
| 2997 | GYS1 | -0.237 | 0.020 | 0.080 | down-reg. | 10.582 | 10.642 | 10.850 | 10.850 |
| 3003 | GZMK | -0.475 | 0.024 | 0.009 | down-reg. | 3.501 | 3.458 | 4.016 | 3.893 |
| 3009 | H1-5 | -0.289 | 0.013 | 0.047 | down-reg. | 10.256 | 10.277 | 10.582 | 10.529 |
| 3018 | H2BC3 | -0.259 | 0.041 | 0.064 | down-reg. | 9.975 | 9.903 | 10.167 | 10.228 |
| 3024 | H1-1 | -0.566 | 0.047 | 0.005 | down-reg. | 6.745 | 6.951 | 7.452 | 7.375 |
| 3148 | HMGB2 | -0.458 | 0.033 | 0.010 | down-reg. | 8.809 | 8.673 | 9.171 | 9.228 |
| 3151 | HMGN2 | -0.352 | 0.020 | 0.026 | down-reg. | 8.530 | 8.535 | 8.841 | 8.928 |
| 3161 | HMMR | -0.340 | 0.038 | 0.028 | down-reg. | 8.791 | 8.738 | 9.158 | 9.051 |
| 3183 | HNRNPC | -0.218 | 0.019 | 0.098 | down-reg. | 11.234 | 11.264 | 11.445 | 11.490 |
| 3207 | HOXA11 | -0.416 | 0.002 | 0.014 | down-reg. | 6.754 | 6.725 | 7.166 | 7.145 |
| 3213 | HOXB3 | -0.291 | 0.049 | 0.046 | down-reg. | 5.590 | 5.705 | 5.948 | 5.928 |
| 3225 | HOXC9 | -0.298 | 0.041 | 0.043 | down-reg. | 6.261 | 6.165 | 6.537 | 6.486 |
| 3283 | HSD3B1 | -0.297 | 0.015 | 0.044 | down-reg. | 4.813 | 4.775 | 5.066 | 5.117 |
| 3383 | ICAM1 | -0.475 | 0.010 | 0.009 | down-reg. | 8.144 | 8.200 | 8.615 | 8.679 |
| 3426 | CFI | -0.282 | 0.029 | 0.050 | down-reg. | 4.430 | 4.363 | 4.652 | 4.704 |
| 3486 | IGFBP3 | -0.701 | 0.020 | 0.002 | down-reg. | 8.482 | 8.650 | 9.243 | 9.291 |
| 3488 | IGFBP5 | -0.373 | 0.028 | 0.021 | down-reg. | 6.955 | 7.027 | 7.323 | 7.405 |
| 3625 | INHBB | -0.219 | 0.031 | 0.097 | down-reg. | 7.662 | 7.657 | 7.844 | 7.913 |
| 3730 | ANOS1 | -0.254 | 0.050 | 0.067 | down-reg. | 6.864 | 6.928 | 7.110 | 7.190 |
| 3776 | KCNK2 | -0.269 | 0.036 | 0.057 | down-reg. | 8.692 | 8.725 | 9.020 | 8.935 |
| 3835 | KIF22 | -0.280 | 0.013 | 0.051 | down-reg. | 8.877 | 8.826 | 9.121 | 9.142 |
| 3880 | KRT19 | -0.501 | 0.046 | 0.008 | down-reg. | 7.813 | 7.962 | 8.325 | 8.451 |
| 3885 | KRT34 | -0.916 | 0.019 | 0.001 | down-reg. | 7.459 | 7.673 | 8.455 | 8.510 |
| 3897 | L1CAM | -0.387 | 0.018 | 0.019 | down-reg. | 7.710 | 7.791 | 8.119 | 8.157 |
| 3930 | LBR | -0.223 | 0.022 | 0.094 | down-reg. | 9.395 | 9.396 | 9.647 | 9.589 |
| 4001 | LMNB1 | -0.287 | 0.013 | 0.048 | down-reg. | 8.967 | 8.956 | 9.221 | 9.277 |
| 4015 | LOX | -0.230 | 0.041 | 0.087 | down-reg. | 11.885 | 11.968 | 12.156 | 12.156 |
| 4017 | LOXL2 | -0.218 | 0.034 | 0.099 | down-reg. | 11.212 | 11.146 | 11.383 | 11.410 |
| 4069 | LYZ | -0.226 | 0.012 | 0.090 | down-reg. | 4.614 | 4.610 | 4.860 | 4.817 |
| 4081 | MAB21L1 | -0.226 | 0.046 | 0.090 | down-reg. | 8.880 | 8.964 | 9.135 | 9.161 |
| 4082 | MARCKS | -0.376 | 0.006 | 0.021 | down-reg. | 10.718 | 10.674 | 11.057 | 11.086 |
| 4091 | SMAD6 | -0.365 | 0.002 | 0.023 | down-reg. | 7.111 | 7.124 | 7.467 | 7.497 |
| 4171 | MCM2 | -0.376 | 0.036 | 0.021 | down-reg. | 8.552 | 8.613 | 8.903 | 9.014 |
| 4173 | MCM4 | -0.335 | 0.032 | 0.030 | down-reg. | 9.771 | 9.781 | 10.164 | 10.058 |
| 4174 | MCM5 | -0.501 | 0.005 | 0.008 | down-reg. | 9.617 | 9.648 | 10.161 | 10.106 |
| 4175 | MCM6 | -0.382 | 0.015 | 0.020 | down-reg. | 9.467 | 9.388 | 9.822 | 9.796 |
| 4176 | MCM7 | -0.389 | 0.031 | 0.018 | down-reg. | 9.862 | 9.753 | 10.170 | 10.223 |
| 4214 | MAP3K1 | -0.278 | 0.036 | 0.052 | down-reg. | 8.868 | 8.791 | 9.134 | 9.082 |
| 4234 | METTL1 | -0.423 | 0.001 | 0.013 | down-reg. | 7.648 | 7.642 | 8.079 | 8.058 |
| 4282 | MIF | -0.267 | 0.007 | 0.059 | down-reg. | 11.331 | 11.331 | 11.617 | 11.579 |
| 4292 | MLH1 | -0.340 | 0.039 | 0.028 | down-reg. | 9.727 | 9.626 | 9.984 | 10.047 |
| 4436 | MSH2 | -0.278 | 0.010 | 0.052 | down-reg. | 8.506 | 8.495 | 8.755 | 8.802 |
| 4601 | MXI1 | -0.273 | 0.041 | 0.055 | down-reg. | 8.395 | 8.407 | 8.723 | 8.625 |
| 4605 | MYBL2 | -0.387 | 0.018 | 0.019 | down-reg. | 8.852 | 8.943 | 9.289 | 9.280 |
| 4678 | NASP | -0.369 | 0.008 | 0.022 | down-reg. | 7.828 | 7.787 | 8.197 | 8.155 |
| 4739 | NEDD9 | -0.534 | 0.032 | 0.006 | down-reg. | 6.882 | 6.964 | 7.383 | 7.531 |
| 4802 | NFYC | -0.226 | 0.016 | 0.090 | down-reg. | 8.542 | 8.505 | 8.767 | 8.734 |
| 4909 | NTF4 | -0.381 | 0.048 | 0.020 | down-reg. | 5.160 | 5.024 | 5.442 | 5.505 |
| 4940 | OAS3 | -0.256 | 0.025 | 0.066 | down-reg. | 6.503 | 6.477 | 6.780 | 6.712 |
| 4998 | ORC1 | -0.617 | 0.005 | 0.003 | down-reg. | 7.276 | 7.354 | 7.930 | 7.935 |
| 5010 | CLDN11 | -0.344 | 0.012 | 0.028 | down-reg. | 10.755 | 10.731 | 11.056 | 11.117 |
| 5028 | P2RY1 | -0.321 | 0.042 | 0.034 | down-reg. | 5.278 | 5.162 | 5.547 | 5.535 |
| 5046 | PCSK6 | -0.237 | 0.015 | 0.080 | down-reg. | 5.775 | 5.739 | 5.976 | 6.012 |
| 5091 | PC | -0.314 | 0.030 | 0.036 | down-reg. | 7.191 | 7.272 | 7.520 | 7.572 |
| 5111 | PCNA | -0.259 | 0.003 | 0.064 | down-reg. | 9.470 | 9.447 | 9.721 | 9.713 |
| 5205 | ATP8B1 | -0.243 | 0.044 | 0.074 | down-reg. | 10.167 | 10.108 | 10.345 | 10.416 |
| 5209 | PFKFB3 | -0.269 | 0.012 | 0.057 | down-reg. | 9.086 | 9.116 | 9.349 | 9.392 |
| 5210 | PFKFB4 | -0.852 | 0.006 | 0.001 | down-reg. | 7.688 | 7.755 | 8.525 | 8.621 |
| 5332 | PLCB4 | -0.765 | 0.019 | 0.001 | down-reg. | 7.621 | 7.701 | 8.343 | 8.509 |
| 5341 | PLEK | -0.219 | 0.023 | 0.098 | down-reg. | 4.235 | 4.197 | 4.457 | 4.412 |
| 5352 | PLOD2 | -0.259 | 0.024 | 0.063 | down-reg. | 11.247 | 11.266 | 11.550 | 11.481 |
| 5424 | POLD1 | -0.234 | 0.005 | 0.082 | down-reg. | 7.464 | 7.435 | 7.680 | 7.688 |
| 5437 | POLR2H | -0.245 | 0.048 | 0.073 | down-reg. | 10.980 | 10.942 | 11.162 | 11.251 |
| 5493 | PPL | -0.368 | 0.021 | 0.022 | down-reg. | 6.514 | 6.435 | 6.869 | 6.817 |
| 5552 | SRGN | -0.614 | 0.007 | 0.004 | down-reg. | 7.759 | 7.845 | 8.431 | 8.400 |
| 5604 | MAP2K1 | -0.291 | 0.030 | 0.047 | down-reg. | 9.556 | 9.626 | 9.855 | 9.909 |
| 5669 | PSG1 | -0.380 | 0.035 | 0.020 | down-reg. | 6.105 | 6.223 | 6.566 | 6.522 |
| 5740 | PTGIS | -0.928 | 0.014 | 0.001 | down-reg. | 8.053 | 7.883 | 8.847 | 8.946 |
| 5743 | PTGS2 | -0.358 | 0.020 | 0.024 | down-reg. | 7.610 | 7.698 | 8.017 | 8.007 |
| 5806 | PTX3 | -0.439 | 0.031 | 0.012 | down-reg. | 10.208 | 10.120 | 10.551 | 10.657 |
| 5827 | PXMP2 | -0.265 | 0.003 | 0.060 | down-reg. | 10.134 | 10.144 | 10.392 | 10.416 |
| 5902 | RANBP1 | -0.225 | 0.049 | 0.091 | down-reg. | 10.004 | 10.076 | 10.237 | 10.292 |
| 5911 | RAP2A | -0.260 | 0.040 | 0.063 | down-reg. | 9.266 | 9.357 | 9.581 | 9.562 |
| 5982 | RFC2 | -0.283 | 0.042 | 0.050 | down-reg. | 9.412 | 9.329 | 9.623 | 9.685 |
| 5999 | RGS4 | -0.455 | 0.014 | 0.010 | down-reg. | 9.216 | 9.130 | 9.610 | 9.647 |
| 6038 | RNASE4 | -0.716 | 0.040 | 0.002 | down-reg. | 8.347 | 8.549 | 9.086 | 9.242 |
| 6086 | RNY4 | -0.238 | 0.040 | 0.079 | down-reg. | 5.984 | 5.927 | 6.225 | 6.161 |
| 6101 | RP1 | -0.382 | 0.010 | 0.020 | down-reg. | 3.980 | 3.930 | 4.314 | 4.361 |
| 6118 | RPA2 | -0.290 | 0.037 | 0.047 | down-reg. | 7.707 | 7.793 | 8.066 | 8.015 |
| 6192 | RPS4Y1 | -0.298 | 0.028 | 0.043 | down-reg. | 9.769 | 9.697 | 10.006 | 10.057 |
| 6240 | RRM1 | -0.233 | 0.045 | 0.084 | down-reg. | 11.332 | 11.250 | 11.541 | 11.507 |
| 6347 | CCL2 | -0.567 | 0.011 | 0.005 | down-reg. | 9.640 | 9.538 | 10.145 | 10.166 |
| 6387 | CXCL12 | -0.395 | 0.027 | 0.017 | down-reg. | 8.279 | 8.227 | 8.598 | 8.699 |
| 6502 | SKP2 | -0.253 | 0.012 | 0.067 | down-reg. | 10.492 | 10.487 | 10.767 | 10.718 |
| 6513 | SLC2A1 | -0.374 | 0.003 | 0.021 | down-reg. | 10.988 | 11.015 | 11.388 | 11.364 |
| 6540 | SLC6A13 | -0.320 | 0.020 | 0.034 | down-reg. | 5.655 | 5.716 | 5.980 | 6.032 |
| 6586 | SLIT3 | -0.263 | 0.012 | 0.061 | down-reg. | 7.732 | 7.682 | 7.964 | 7.976 |
| 6626 | SNRPA | -0.252 | 0.012 | 0.068 | down-reg. | 10.655 | 10.693 | 10.912 | 10.940 |
| 6629 | SNRPB2 | -0.218 | 0.050 | 0.099 | down-reg. | 9.788 | 9.702 | 9.972 | 9.954 |
| 6668 | SP2 | -0.313 | 0.002 | 0.037 | down-reg. | 7.165 | 7.172 | 7.469 | 7.493 |
| 6676 | SPAG4 | -0.481 | 0.009 | 0.009 | down-reg. | 7.376 | 7.453 | 7.882 | 7.909 |
| 6749 | SSRP1 | -0.271 | 0.050 | 0.056 | down-reg. | 9.837 | 9.764 | 10.031 | 10.113 |
| 6876 | TAGLN | -0.684 | 0.007 | 0.002 | down-reg. | 7.234 | 7.310 | 7.924 | 7.988 |
| 6882 | TAF11 | -0.313 | 0.028 | 0.037 | down-reg. | 6.749 | 6.837 | 7.091 | 7.120 |
| 6993 | DYNLT1 | -0.225 | 0.012 | 0.091 | down-reg. | 9.607 | 9.640 | 9.863 | 9.836 |
| 7083 | TK1 | -0.447 | 0.009 | 0.011 | down-reg. | 10.271 | 10.336 | 10.770 | 10.732 |
| 7099 | TLR4 | -0.521 | 0.019 | 0.007 | down-reg. | 7.707 | 7.584 | 8.182 | 8.150 |
| 7298 | TYMS | -0.427 | 0.036 | 0.013 | down-reg. | 11.101 | 10.959 | 11.470 | 11.444 |
| 7351 | UCP2 | -0.473 | 0.001 | 0.009 | down-reg. | 7.746 | 7.723 | 8.198 | 8.216 |
| 7357 | UGCG | -0.364 | 0.021 | 0.023 | down-reg. | 9.660 | 9.651 | 9.974 | 10.065 |
| 7422 | VEGFA | -0.234 | 0.004 | 0.083 | down-reg. | 10.097 | 10.108 | 10.349 | 10.324 |
| 7430 | EZR | -0.246 | 0.022 | 0.072 | down-reg. | 10.212 | 10.212 | 10.427 | 10.491 |
| 7436 | VLDLR | -0.682 | 0.024 | 0.002 | down-reg. | 7.402 | 7.573 | 8.133 | 8.206 |
| 7516 | XRCC2 | -0.425 | 0.012 | 0.013 | down-reg. | 7.584 | 7.636 | 8.067 | 8.002 |
| 7541 | ZBTB14 | -0.292 | 0.008 | 0.046 | down-reg. | 7.340 | 7.361 | 7.623 | 7.662 |
| 7570 | ZNF22 | -0.255 | 0.014 | 0.066 | down-reg. | 8.272 | 8.224 | 8.513 | 8.493 |
| 7678 | ZNF124 | -0.379 | 0.011 | 0.020 | down-reg. | 7.338 | 7.333 | 7.749 | 7.681 |
| 7739 | ZNF185 | -0.651 | 0.024 | 0.003 | down-reg. | 6.730 | 6.809 | 7.340 | 7.501 |
| 7773 | ZNF230 | -0.524 | 0.023 | 0.007 | down-reg. | 5.882 | 5.806 | 6.309 | 6.427 |
| 8076 | MFAP5 | -0.559 | 0.031 | 0.005 | down-reg. | 6.386 | 6.473 | 6.913 | 7.064 |
| 8200 | GDF5 | -0.410 | 0.011 | 0.015 | down-reg. | 5.896 | 5.828 | 6.255 | 6.288 |
| 8208 | CHAF1B | -0.462 | 0.001 | 0.010 | down-reg. | 8.323 | 8.308 | 8.764 | 8.791 |
| 8243 | SMC1A | -0.315 | 0.029 | 0.036 | down-reg. | 9.410 | 9.320 | 9.695 | 9.664 |
| 8329 | H2AC13 | -0.284 | 0.031 | 0.050 | down-reg. | 10.976 | 10.889 | 11.222 | 11.210 |
| 8330 | H2AC15 | -0.390 | 0.031 | 0.018 | down-reg. | 9.809 | 9.739 | 10.214 | 10.114 |
| 8336 | H2AC17 | -0.500 | 0.003 | 0.008 | down-reg. | 7.581 | 7.599 | 8.068 | 8.113 |
| 8347 | H2BC4 | -0.383 | 0.017 | 0.020 | down-reg. | 8.799 | 8.828 | 9.155 | 9.237 |
| 8356 | H3C12 | -0.313 | 0.037 | 0.037 | down-reg. | 10.008 | 10.116 | 10.375 | 10.376 |
| 8357 | H3C10 | -0.477 | 0.021 | 0.009 | down-reg. | 8.510 | 8.448 | 8.904 | 9.008 |
| 8436 | CAVIN2 | -0.329 | 0.008 | 0.032 | down-reg. | 5.149 | 5.166 | 5.511 | 5.462 |
| 8483 | CILP | -0.249 | 0.012 | 0.070 | down-reg. | 5.796 | 5.791 | 6.066 | 6.019 |
| 8553 | BHLHE40 | -0.283 | 0.036 | 0.050 | down-reg. | 8.728 | 8.722 | 8.961 | 9.056 |
| 8671 | SLC4A4 | -0.228 | 0.019 | 0.088 | down-reg. | 5.714 | 5.768 | 5.962 | 5.976 |
| 8727 | CTNNAL1 | -0.339 | 0.035 | 0.029 | down-reg. | 10.431 | 10.344 | 10.690 | 10.762 |
| 8914 | TIMELESS | -0.379 | 0.035 | 0.020 | down-reg. | 7.673 | 7.555 | 7.971 | 8.016 |
| 8970 | H2BC11 | -0.345 | 0.000 | 0.027 | down-reg. | 7.204 | 7.215 | 7.557 | 7.552 |
| 8987 | STBD1 | -0.414 | 0.007 | 0.015 | down-reg. | 6.188 | 6.200 | 6.638 | 6.577 |
| 9052 | GPRC5A | -0.672 | 0.026 | 0.003 | down-reg. | 7.957 | 7.873 | 8.501 | 8.673 |
| 9055 | PRC1 | -0.414 | 0.027 | 0.015 | down-reg. | 10.081 | 9.962 | 10.442 | 10.430 |
| 9122 | SLC16A4 | -0.424 | 0.004 | 0.013 | down-reg. | 8.535 | 8.556 | 8.948 | 8.992 |
| 9124 | PDLIM1 | -0.282 | 0.028 | 0.050 | down-reg. | 10.764 | 10.715 | 10.988 | 11.056 |
| 9175 | MAP3K13 | -0.226 | 0.031 | 0.090 | down-reg. | 6.642 | 6.605 | 6.880 | 6.820 |
| 9232 | PTTG1 | -0.517 | 0.003 | 0.007 | down-reg. | 9.492 | 9.445 | 9.977 | 9.995 |
| 9249 | DHRS3 | -0.558 | 0.032 | 0.005 | down-reg. | 6.058 | 6.230 | 6.724 | 6.679 |
| 9308 | CD83 | -0.248 | 0.044 | 0.070 | down-reg. | 6.362 | 6.455 | 6.659 | 6.654 |
| 9319 | TRIP13 | -0.352 | 0.045 | 0.025 | down-reg. | 9.734 | 9.629 | 9.991 | 10.076 |
| 9337 | CNOT8 | -0.281 | 0.036 | 0.051 | down-reg. | 9.396 | 9.361 | 9.615 | 9.703 |
| 9397 | NMT2 | -0.286 | 0.013 | 0.048 | down-reg. | 9.822 | 9.846 | 10.094 | 10.146 |
| 9476 | NAPSA | -0.330 | 0.045 | 0.031 | down-reg. | 5.738 | 5.780 | 6.148 | 6.030 |
| 9582 | APOBEC3B | -0.504 | 0.003 | 0.008 | down-reg. | 10.329 | 10.282 | 10.818 | 10.801 |
| 9633 | TESMIN | -0.282 | 0.020 | 0.050 | down-reg. | 6.101 | 6.071 | 6.401 | 6.336 |
| 9735 | KNTC1 | -0.307 | 0.025 | 0.039 | down-reg. | 8.998 | 8.919 | 9.249 | 9.282 |
| 9770 | RASSF2 | -0.497 | 0.047 | 0.008 | down-reg. | 5.068 | 5.089 | 5.479 | 5.673 |
| 9787 | DLGAP5 | -0.479 | 0.011 | 0.009 | down-reg. | 9.847 | 9.762 | 10.297 | 10.270 |
| 9837 | GINS1 | -0.232 | 0.015 | 0.084 | down-reg. | 9.065 | 9.021 | 9.263 | 9.286 |
| 9846 | GAB2 | -0.222 | 0.032 | 0.095 | down-reg. | 6.944 | 7.013 | 7.209 | 7.191 |
| 9918 | NCAPD2 | -0.400 | 0.014 | 0.017 | down-reg. | 10.894 | 10.817 | 11.273 | 11.239 |
| 10055 | SAE1 | -0.266 | 0.003 | 0.059 | down-reg. | 10.897 | 10.875 | 11.160 | 11.146 |
| 10058 | ABCB6 | -0.328 | 0.033 | 0.032 | down-reg. | 6.871 | 6.841 | 7.133 | 7.236 |
| 10082 | GPC6 | -0.463 | 0.011 | 0.010 | down-reg. | 8.893 | 8.864 | 9.381 | 9.301 |
| 10105 | PPIF | -0.246 | 0.049 | 0.072 | down-reg. | 9.465 | 9.479 | 9.669 | 9.766 |
| 10112 | KIF20A | -0.673 | 0.022 | 0.003 | down-reg. | 10.312 | 10.135 | 10.901 | 10.892 |
| 10129 | FRY | -0.245 | 0.048 | 0.072 | down-reg. | 7.377 | 7.421 | 7.601 | 7.687 |
| 10165 | SLC25A13 | -0.223 | 0.019 | 0.093 | down-reg. | 9.118 | 9.096 | 9.355 | 9.305 |
| 10207 | PATJ | -0.307 | 0.032 | 0.039 | down-reg. | 6.710 | 6.688 | 6.959 | 7.053 |
| 10234 | LRRC17 | -0.475 | 0.046 | 0.009 | down-reg. | 9.207 | 9.238 | 9.607 | 9.788 |
| 10236 | HNRNPR | -0.220 | 0.005 | 0.097 | down-reg. | 10.949 | 10.969 | 11.189 | 11.169 |
| 10252 | SPRY1 | -0.275 | 0.035 | 0.054 | down-reg. | 7.653 | 7.633 | 7.873 | 7.962 |
| 10351 | ABCA8 | -0.672 | 0.047 | 0.003 | down-reg. | 5.943 | 5.755 | 6.612 | 6.431 |
| 10397 | NDRG1 | -0.433 | 0.008 | 0.012 | down-reg. | 10.542 | 10.480 | 10.932 | 10.957 |
| 10424 | PGRMC2 | -0.232 | 0.044 | 0.084 | down-reg. | 10.298 | 10.384 | 10.581 | 10.566 |
| 10445 | MCRS1 | -0.284 | 0.041 | 0.049 | down-reg. | 8.540 | 8.453 | 8.808 | 8.753 |
| 10460 | TACC3 | -0.230 | 0.010 | 0.086 | down-reg. | 8.317 | 8.357 | 8.568 | 8.566 |
| 10465 | PPIH | -0.377 | 0.044 | 0.021 | down-reg. | 8.121 | 7.986 | 8.452 | 8.407 |
| 10486 | CAP2 | -0.410 | 0.006 | 0.015 | down-reg. | 8.372 | 8.340 | 8.743 | 8.790 |
| 10570 | DPYSL4 | -0.443 | 0.015 | 0.011 | down-reg. | 7.505 | 7.601 | 7.997 | 7.995 |
| 10615 | SPAG5 | -0.546 | 0.005 | 0.005 | down-reg. | 8.839 | 8.819 | 9.406 | 9.343 |
| 10762 | NUP50 | -0.254 | 0.031 | 0.066 | down-reg. | 8.534 | 8.548 | 8.834 | 8.757 |
| 10814 | CPLX2 | -0.231 | 0.001 | 0.085 | down-reg. | 6.332 | 6.333 | 6.570 | 6.558 |
| 10877 | CFHR4 | -0.342 | 0.013 | 0.028 | down-reg. | 4.323 | 4.277 | 4.667 | 4.617 |
| 10969 | EBNA1BP2 | -0.223 | 0.015 | 0.093 | down-reg. | 10.528 | 10.547 | 10.738 | 10.783 |
| 11004 | KIF2C | -0.319 | 0.010 | 0.035 | down-reg. | 8.714 | 8.665 | 9.023 | 8.996 |
| 11065 | UBE2C | -0.243 | 0.019 | 0.074 | down-reg. | 9.186 | 9.130 | 9.409 | 9.393 |
| 11073 | TOPBP1 | -0.218 | 0.027 | 0.099 | down-reg. | 9.985 | 10.044 | 10.244 | 10.222 |
| 11082 | ESM1 | -0.416 | 0.045 | 0.014 | down-reg. | 6.690 | 6.836 | 7.147 | 7.209 |
| 11098 | PRSS23 | -0.219 | 0.036 | 0.098 | down-reg. | 9.633 | 9.570 | 9.802 | 9.840 |
| 11113 | CIT | -0.341 | 0.020 | 0.028 | down-reg. | 9.538 | 9.453 | 9.843 | 9.830 |
| 11200 | CHEK2 | -0.391 | 0.028 | 0.018 | down-reg. | 6.489 | 6.474 | 6.816 | 6.929 |
| 11211 | FZD10 | -0.254 | 0.008 | 0.066 | down-reg. | 6.573 | 6.543 | 6.799 | 6.826 |
| 11245 | GPR176 | -0.224 | 0.046 | 0.092 | down-reg. | 9.856 | 9.773 | 10.026 | 10.051 |
| 11266 | DUSP12 | -0.316 | 0.039 | 0.036 | down-reg. | 8.387 | 8.311 | 8.625 | 8.707 |
| 11333 | PDAP1 | -0.293 | 0.035 | 0.045 | down-reg. | 8.907 | 8.965 | 9.268 | 9.190 |
| 11339 | OIP5 | -0.292 | 0.026 | 0.046 | down-reg. | 7.708 | 7.790 | 8.037 | 8.046 |
| 11340 | EXOSC8 | -0.406 | 0.049 | 0.016 | down-reg. | 9.824 | 9.667 | 10.131 | 10.172 |
| 22895 | RPH3A | -0.242 | 0.004 | 0.075 | down-reg. | 5.202 | 5.190 | 5.425 | 5.451 |
| 23029 | RBM34 | -0.584 | 0.044 | 0.004 | down-reg. | 3.672 | 3.884 | 4.333 | 4.393 |
| 23210 | JMJD6 | -0.374 | 0.019 | 0.021 | down-reg. | 7.972 | 7.955 | 8.293 | 8.383 |
| 23236 | PLCB1 | -0.325 | 0.020 | 0.033 | down-reg. | 7.226 | 7.227 | 7.592 | 7.510 |
| 23327 | NEDD4L | -0.564 | 0.034 | 0.005 | down-reg. | 7.890 | 7.704 | 8.359 | 8.363 |
| 23409 | SIRT4 | -0.306 | 0.023 | 0.040 | down-reg. | 5.395 | 5.362 | 5.722 | 5.647 |
| 23435 | TARDBP | -0.294 | 0.010 | 0.045 | down-reg. | 9.301 | 9.252 | 9.563 | 9.577 |
| 23519 | ANP32D | -0.294 | 0.014 | 0.045 | down-reg. | 4.312 | 4.325 | 4.582 | 4.642 |
| 23647 | ARFIP2 | -0.234 | 0.040 | 0.083 | down-reg. | 8.944 | 8.870 | 9.122 | 9.159 |
| 23649 | POLA2 | -0.255 | 0.024 | 0.066 | down-reg. | 8.647 | 8.593 | 8.898 | 8.852 |
| 23682 | RAB38 | -0.241 | 0.048 | 0.076 | down-reg. | 9.588 | 9.511 | 9.819 | 9.762 |
| 24141 | LAMP5 | -0.359 | 0.048 | 0.024 | down-reg. | 5.362 | 5.235 | 5.690 | 5.624 |
| 24147 | FJX1 | -0.318 | 0.029 | 0.035 | down-reg. | 9.284 | 9.285 | 9.651 | 9.555 |
| 25818 | KLK5 | -0.338 | 0.032 | 0.029 | down-reg. | 6.087 | 6.193 | 6.488 | 6.468 |
| 25906 | ANAPC15 | -0.330 | 0.044 | 0.031 | down-reg. | 9.608 | 9.666 | 10.023 | 9.912 |
| 26147 | PHF19 | -0.388 | 0.040 | 0.018 | down-reg. | 8.914 | 8.800 | 9.285 | 9.206 |
| 26227 | PHGDH | -0.495 | 0.005 | 0.008 | down-reg. | 10.206 | 10.162 | 10.656 | 10.702 |
| 26233 | FBXL6 | -0.375 | 0.010 | 0.021 | down-reg. | 6.991 | 7.034 | 7.412 | 7.363 |
| 26257 | NKX2-8 | -0.262 | 0.048 | 0.062 | down-reg. | 6.175 | 6.143 | 6.470 | 6.371 |
| 26996 | GPR160 | -0.339 | 0.006 | 0.029 | down-reg. | 5.054 | 5.056 | 5.371 | 5.418 |
| 27229 | TUBGCP4 | -0.219 | 0.043 | 0.097 | down-reg. | 9.651 | 9.633 | 9.822 | 9.901 |
| 27258 | LSM3 | -0.258 | 0.021 | 0.064 | down-reg. | 11.794 | 11.859 | 12.077 | 12.093 |
| 28990 | ASTE1 | -0.255 | 0.006 | 0.066 | down-reg. | 6.780 | 6.746 | 7.015 | 7.022 |
| 29091 | STXBP6 | -0.351 | 0.018 | 0.026 | down-reg. | 7.955 | 7.931 | 8.255 | 8.333 |
| 29923 | HILPDA | -0.762 | 0.014 | 0.001 | down-reg. | 8.208 | 8.065 | 8.933 | 8.864 |
| 29968 | PSAT1 | -0.354 | 0.032 | 0.025 | down-reg. | 9.323 | 9.221 | 9.652 | 9.601 |
| 51016 | EMC9 | -0.347 | 0.007 | 0.027 | down-reg. | 7.057 | 7.097 | 7.407 | 7.441 |
| 51060 | TXNDC12 | -0.249 | 0.009 | 0.070 | down-reg. | 10.820 | 10.822 | 11.050 | 11.091 |
| 51065 | RPS27L | -0.223 | 0.006 | 0.094 | down-reg. | 8.938 | 8.962 | 9.163 | 9.183 |
| 51121 | RPL26L1 | -0.327 | 0.049 | 0.032 | down-reg. | 6.803 | 6.710 | 7.130 | 7.037 |
| 51129 | ANGPTL4 | -0.490 | 0.044 | 0.008 | down-reg. | 9.064 | 9.231 | 9.597 | 9.677 |
| 51141 | INSIG2 | -0.720 | 0.021 | 0.002 | down-reg. | 8.600 | 8.500 | 9.346 | 9.194 |
| 51155 | JPT1 | -0.223 | 0.014 | 0.093 | down-reg. | 9.870 | 9.909 | 10.100 | 10.125 |
| 51181 | DCXR | -0.235 | 0.016 | 0.082 | down-reg. | 10.249 | 10.223 | 10.494 | 10.448 |
| 51200 | CPA4 | -0.628 | 0.006 | 0.003 | down-reg. | 9.443 | 9.504 | 10.073 | 10.132 |
| 51228 | GLTP | -0.288 | 0.009 | 0.048 | down-reg. | 9.232 | 9.193 | 9.514 | 9.485 |
| 51265 | CDKL3 | -0.354 | 0.020 | 0.025 | down-reg. | 5.065 | 5.023 | 5.360 | 5.436 |
| 51460 | SFMBT1 | -0.461 | 0.047 | 0.010 | down-reg. | 6.532 | 6.449 | 6.872 | 7.031 |
| 51491 | NOP16 | -0.399 | 0.019 | 0.017 | down-reg. | 8.067 | 8.146 | 8.532 | 8.479 |
| 51514 | DTL | -0.324 | 0.012 | 0.033 | down-reg. | 9.894 | 9.905 | 10.255 | 10.193 |
| 51659 | GINS2 | -0.371 | 0.015 | 0.022 | down-reg. | 9.404 | 9.381 | 9.725 | 9.801 |
| 53340 | SPA17 | -0.395 | 0.033 | 0.017 | down-reg. | 9.855 | 9.881 | 10.200 | 10.325 |
| 54108 | CHRAC1 | -0.262 | 0.021 | 0.061 | down-reg. | 9.732 | 9.794 | 10.012 | 10.038 |
| 54332 | GDAP1 | -0.322 | 0.016 | 0.034 | down-reg. | 6.582 | 6.568 | 6.932 | 6.863 |
| 54478 | PIMREG | -0.307 | 0.019 | 0.039 | down-reg. | 8.316 | 8.372 | 8.626 | 8.676 |
| 54522 | ANKRD16 | -0.301 | 0.016 | 0.042 | down-reg. | 7.313 | 7.253 | 7.597 | 7.569 |
| 54541 | DDIT4 | -0.528 | 0.013 | 0.006 | down-reg. | 9.174 | 9.237 | 9.777 | 9.690 |
| 54583 | EGLN1 | -0.336 | 0.016 | 0.030 | down-reg. | 10.561 | 10.487 | 10.853 | 10.868 |
| 54707 | GPN2 | -0.258 | 0.034 | 0.064 | down-reg. | 8.184 | 8.174 | 8.395 | 8.479 |
| 54802 | TRIT1 | -0.219 | 0.041 | 0.098 | down-reg. | 8.350 | 8.429 | 8.608 | 8.608 |
| 54820 | NDE1 | -0.221 | 0.019 | 0.096 | down-reg. | 9.009 | 8.992 | 9.196 | 9.247 |
| 54843 | SYTL2 | -0.268 | 0.018 | 0.058 | down-reg. | 7.259 | 7.269 | 7.563 | 7.500 |
| 54880 | BCOR | -0.252 | 0.024 | 0.068 | down-reg. | 7.906 | 7.973 | 8.200 | 8.184 |
| 54892 | NCAPG2 | -0.382 | 0.041 | 0.020 | down-reg. | 8.972 | 8.837 | 9.272 | 9.301 |
| 54956 | PARP16 | -0.664 | 0.046 | 0.003 | down-reg. | 7.421 | 7.289 | 7.909 | 8.129 |
| 55013 | MCUB | -0.304 | 0.003 | 0.040 | down-reg. | 8.817 | 8.808 | 9.130 | 9.103 |
| 55051 | NRDE2 | -0.233 | 0.040 | 0.084 | down-reg. | 6.645 | 6.566 | 6.852 | 6.824 |
| 55055 | ZWILCH | -0.247 | 0.006 | 0.071 | down-reg. | 9.942 | 9.970 | 10.193 | 10.212 |
| 55076 | TMEM45A | -0.610 | 0.002 | 0.004 | down-reg. | 9.079 | 9.053 | 9.698 | 9.654 |
| 55139 | ANKZF1 | -0.410 | 0.042 | 0.015 | down-reg. | 7.262 | 7.411 | 7.741 | 7.751 |
| 55157 | DARS2 | -0.234 | 0.004 | 0.083 | down-reg. | 9.565 | 9.558 | 9.782 | 9.808 |
| 55214 | P3H2 | -0.239 | 0.011 | 0.078 | down-reg. | 7.502 | 7.479 | 7.711 | 7.748 |
| 55215 | FANCI | -0.375 | 0.013 | 0.021 | down-reg. | 9.001 | 9.052 | 9.430 | 9.373 |
| 55246 | CCDC25 | -0.240 | 0.022 | 0.077 | down-reg. | 9.460 | 9.509 | 9.744 | 9.705 |
| 55308 | DDX19A | -0.318 | 0.001 | 0.035 | down-reg. | 8.500 | 8.512 | 8.815 | 8.833 |
| 55634 | KRBOX4 | -0.374 | 0.001 | 0.021 | down-reg. | 8.111 | 8.096 | 8.488 | 8.467 |
| 55647 | RAB20 | -0.302 | 0.017 | 0.041 | down-reg. | 7.265 | 7.225 | 7.519 | 7.576 |
| 55689 | YEATS2 | -0.257 | 0.037 | 0.064 | down-reg. | 10.117 | 10.083 | 10.317 | 10.398 |
| 55706 | NDC1 | -0.269 | 0.026 | 0.058 | down-reg. | 9.924 | 9.871 | 10.194 | 10.138 |
| 55771 | PRR11 | -0.485 | 0.025 | 0.009 | down-reg. | 9.687 | 9.560 | 10.083 | 10.134 |
| 55775 | TDP1 | -0.278 | 0.026 | 0.052 | down-reg. | 9.592 | 9.629 | 9.853 | 9.924 |
| 55789 | DEPDC1B | -0.240 | 0.019 | 0.077 | down-reg. | 7.929 | 7.872 | 8.134 | 8.147 |
| 55816 | DOK5 | -0.426 | 0.028 | 0.013 | down-reg. | 6.665 | 6.768 | 7.105 | 7.179 |
| 55818 | KDM3A | -0.385 | 0.008 | 0.019 | down-reg. | 9.032 | 9.082 | 9.425 | 9.458 |
| 55859 | BEX1 | -0.263 | 0.040 | 0.061 | down-reg. | 9.471 | 9.556 | 9.797 | 9.755 |
| 56133 | PCDHB2 | -0.231 | 0.017 | 0.086 | down-reg. | 5.609 | 5.597 | 5.859 | 5.808 |
| 56662 | VTRNA1-3 | -0.511 | 0.029 | 0.007 | down-reg. | 8.552 | 8.513 | 9.119 | 8.969 |
| 56992 | KIF15 | -0.302 | 0.028 | 0.041 | down-reg. | 8.448 | 8.360 | 8.715 | 8.696 |
| 57122 | NUP107 | -0.385 | 0.019 | 0.019 | down-reg. | 8.965 | 8.877 | 9.290 | 9.322 |
| 57136 | APMAP | -0.258 | 0.018 | 0.064 | down-reg. | 8.691 | 8.747 | 8.966 | 8.987 |
| 57221 | ARFGEF3 | -0.303 | 0.017 | 0.041 | down-reg. | 5.986 | 5.994 | 6.328 | 6.260 |
| 57481 | KIAA1210 | -0.273 | 0.009 | 0.055 | down-reg. | 4.517 | 4.518 | 4.813 | 4.767 |
| 57482 | CRACD | -0.553 | 0.013 | 0.005 | down-reg. | 5.813 | 5.908 | 6.384 | 6.444 |
| 57484 | RNF150 | -0.383 | 0.001 | 0.019 | down-reg. | 6.967 | 6.940 | 7.340 | 7.334 |
| 57493 | HEG1 | -0.263 | 0.015 | 0.061 | down-reg. | 10.387 | 10.415 | 10.639 | 10.688 |
| 57643 | ZSWIM5 | -0.728 | 0.003 | 0.002 | down-reg. | 6.835 | 6.767 | 7.541 | 7.517 |
| 57646 | USP28 | -0.220 | 0.034 | 0.097 | down-reg. | 9.068 | 9.004 | 9.239 | 9.273 |
| 57698 | SHTN1 | -0.389 | 0.008 | 0.018 | down-reg. | 7.183 | 7.162 | 7.592 | 7.532 |
| 57709 | SLC7A14 | -0.380 | 0.042 | 0.020 | down-reg. | 7.370 | 7.377 | 7.823 | 7.684 |
| 58191 | CXCL16 | -0.314 | 0.021 | 0.037 | down-reg. | 5.710 | 5.770 | 6.081 | 6.027 |
| 58475 | MS4A7 | -0.268 | 0.017 | 0.058 | down-reg. | 4.147 | 4.111 | 4.371 | 4.422 |
| 58494 | JAM2 | -0.640 | 0.010 | 0.003 | down-reg. | 6.570 | 6.504 | 7.132 | 7.221 |
| 58538 | MPP4 | -0.720 | 0.008 | 0.002 | down-reg. | 6.923 | 6.811 | 7.600 | 7.574 |
| 59277 | NTN4 | -0.511 | 0.010 | 0.007 | down-reg. | 9.371 | 9.279 | 9.837 | 9.836 |
| 59339 | PLEKHA2 | -0.235 | 0.007 | 0.082 | down-reg. | 9.986 | 9.968 | 10.198 | 10.226 |
| 63931 | MRPS14 | -0.263 | 0.019 | 0.061 | down-reg. | 9.203 | 9.159 | 9.467 | 9.421 |
| 63934 | ZNF667 | -0.413 | 0.039 | 0.015 | down-reg. | 7.420 | 7.413 | 7.903 | 7.757 |
| 63967 | CLSPN | -0.292 | 0.006 | 0.046 | down-reg. | 7.327 | 7.290 | 7.606 | 7.594 |
| 64081 | PBLD | -0.314 | 0.046 | 0.037 | down-reg. | 5.905 | 5.961 | 6.193 | 6.301 |
| 64149 | C17orf75 | -0.247 | 0.043 | 0.071 | down-reg. | 7.459 | 7.383 | 7.693 | 7.642 |
| 64172 | OSGEPL1 | -0.330 | 0.004 | 0.031 | down-reg. | 7.778 | 7.738 | 8.086 | 8.090 |
| 64240 | ABCG5 | -0.253 | 0.029 | 0.067 | down-reg. | 5.300 | 5.225 | 5.521 | 5.511 |
| 64420 | SUSD1 | -0.227 | 0.047 | 0.088 | down-reg. | 8.478 | 8.405 | 8.694 | 8.644 |
| 64682 | ANAPC1 | -0.228 | 0.005 | 0.088 | down-reg. | 9.764 | 9.756 | 9.974 | 10.001 |
| 64975 | MRPL41 | -0.642 | 0.002 | 0.003 | down-reg. | 8.569 | 8.563 | 9.183 | 9.233 |
| 65080 | MRPL44 | -0.219 | 0.024 | 0.097 | down-reg. | 8.381 | 8.376 | 8.568 | 8.628 |
| 79019 | CENPM | -0.400 | 0.010 | 0.017 | down-reg. | 7.936 | 7.920 | 8.363 | 8.294 |
| 79029 | SPATA5L1 | -0.261 | 0.029 | 0.062 | down-reg. | 7.436 | 7.434 | 7.736 | 7.657 |
| 79365 | BHLHE41 | -0.698 | 0.021 | 0.002 | down-reg. | 7.533 | 7.366 | 8.118 | 8.179 |
| 79625 | NDNF | -0.469 | 0.022 | 0.009 | down-reg. | 6.550 | 6.653 | 7.036 | 7.104 |
| 79656 | BEND5 | -0.321 | 0.026 | 0.034 | down-reg. | 5.232 | 5.307 | 5.564 | 5.618 |
| 79672 | FN3KRP | -0.327 | 0.038 | 0.032 | down-reg. | 9.051 | 8.980 | 9.386 | 9.298 |
| 79689 | STEAP4 | -0.647 | 0.013 | 0.003 | down-reg. | 4.631 | 4.598 | 5.324 | 5.200 |
| 79805 | VASH2 | -0.299 | 0.006 | 0.043 | down-reg. | 6.653 | 6.695 | 6.971 | 6.974 |
| 79845 | RNF122 | -0.753 | 0.010 | 0.002 | down-reg. | 6.475 | 6.425 | 7.264 | 7.140 |
| 79850 | TLCD3A | -0.259 | 0.017 | 0.063 | down-reg. | 10.192 | 10.170 | 10.467 | 10.413 |
| 79929 | MAP6D1 | -0.225 | 0.030 | 0.091 | down-reg. | 8.564 | 8.571 | 8.826 | 8.758 |
| 79968 | WDR76 | -0.413 | 0.000 | 0.015 | down-reg. | 9.724 | 9.719 | 10.134 | 10.135 |
| 79987 | SVEP1 | -0.549 | 0.022 | 0.005 | down-reg. | 8.659 | 8.519 | 9.158 | 9.118 |
| 79991 | STN1 | -0.271 | 0.022 | 0.056 | down-reg. | 7.983 | 7.988 | 8.220 | 8.291 |
| 79998 | ANKRD53 | -0.289 | 0.037 | 0.047 | down-reg. | 6.485 | 6.409 | 6.703 | 6.768 |
| 80006 | TRAPPC13 | -0.225 | 0.024 | 0.091 | down-reg. | 7.500 | 7.452 | 7.682 | 7.720 |
| 80008 | TMEM156 | -0.265 | 0.046 | 0.060 | down-reg. | 5.606 | 5.504 | 5.817 | 5.824 |
| 80011 | PSME3IP1 | -0.253 | 0.038 | 0.067 | down-reg. | 8.485 | 8.398 | 8.688 | 8.702 |
| 80031 | SEMA6D | -0.241 | 0.043 | 0.076 | down-reg. | 7.834 | 7.878 | 8.058 | 8.136 |
| 80099 | C7orf69 | -0.755 | 0.016 | 0.002 | down-reg. | 5.005 | 4.889 | 5.764 | 5.640 |
| 80316 | PPP1R2C | -0.386 | 0.002 | 0.019 | down-reg. | 5.764 | 5.769 | 6.137 | 6.168 |
| 81610 | FAM83D | -0.225 | 0.015 | 0.091 | down-reg. | 9.954 | 9.913 | 10.172 | 10.147 |
| 81611 | ANP32E | -0.345 | 0.024 | 0.027 | down-reg. | 8.992 | 8.898 | 9.284 | 9.297 |
| 81691 | REXO5 | -0.268 | 0.049 | 0.058 | down-reg. | 7.477 | 7.461 | 7.790 | 7.684 |
| 81844 | TRIM56 | -0.564 | 0.012 | 0.005 | down-reg. | 6.555 | 6.626 | 7.197 | 7.111 |
| 83755 | KRTAP4-12 | -0.224 | 0.009 | 0.092 | down-reg. | 5.993 | 5.972 | 6.222 | 6.191 |
| 83879 | CDCA7 | -0.403 | 0.009 | 0.016 | down-reg. | 7.823 | 7.771 | 8.179 | 8.221 |
| 83895 | KRTAP1-5 | -0.440 | 0.009 | 0.012 | down-reg. | 12.525 | 12.535 | 12.935 | 13.006 |
| 83931 | STK40 | -0.229 | 0.042 | 0.087 | down-reg. | 7.864 | 7.923 | 8.093 | 8.153 |
| 84058 | WDR54 | -0.311 | 0.009 | 0.038 | down-reg. | 8.759 | 8.707 | 9.050 | 9.038 |
| 84203 | TXNDC2 | -0.355 | 0.032 | 0.025 | down-reg. | 5.371 | 5.465 | 5.742 | 5.804 |
| 84318 | CCDC77 | -0.250 | 0.007 | 0.070 | down-reg. | 7.538 | 7.566 | 7.813 | 7.790 |
| 84337 | ELOF1 | -0.324 | 0.018 | 0.033 | down-reg. | 9.165 | 9.151 | 9.521 | 9.445 |
| 84775 | ZNF607 | -0.260 | 0.036 | 0.063 | down-reg. | 6.905 | 6.987 | 7.221 | 7.189 |
| 84823 | LMNB2 | -0.291 | 0.007 | 0.047 | down-reg. | 10.858 | 10.839 | 11.158 | 11.121 |
| 84996 | URB1-AS1 | -0.250 | 0.023 | 0.069 | down-reg. | 7.837 | 7.860 | 8.131 | 8.067 |
| 89891 | DYNC2I2 | -0.292 | 0.002 | 0.046 | down-reg. | 8.607 | 8.588 | 8.880 | 8.898 |
| 89894 | TMEM116 | -0.273 | 0.042 | 0.055 | down-reg. | 8.100 | 8.190 | 8.441 | 8.395 |
| 90121 | TSR2 | -0.393 | 0.028 | 0.018 | down-reg. | 7.816 | 7.705 | 8.136 | 8.171 |
| 91057 | CCDC34 | -0.455 | 0.012 | 0.010 | down-reg. | 7.343 | 7.387 | 7.781 | 7.858 |
| 91392 | ZNF502 | -0.247 | 0.010 | 0.072 | down-reg. | 6.082 | 6.039 | 6.308 | 6.307 |
| 91607 | SLFN11 | -0.358 | 0.027 | 0.024 | down-reg. | 9.612 | 9.521 | 9.900 | 9.949 |
| 91749 | MFSD4B | -0.327 | 0.040 | 0.032 | down-reg. | 7.611 | 7.586 | 7.869 | 7.983 |
| 94039 | ZNF101 | -0.332 | 0.035 | 0.031 | down-reg. | 7.292 | 7.382 | 7.637 | 7.701 |
| 94239 | H2AZ2 | -0.243 | 0.049 | 0.074 | down-reg. | 10.539 | 10.456 | 10.766 | 10.715 |
| 112714 | TUBA3E | -0.636 | 0.000 | 0.003 | down-reg. | 5.494 | 5.498 | 6.123 | 6.140 |
| 114034 | TOE1 | -0.351 | 0.035 | 0.026 | down-reg. | 8.279 | 8.176 | 8.550 | 8.607 |
| 114932 | MRFAP1L1 | -0.386 | 0.014 | 0.019 | down-reg. | 7.317 | 7.235 | 7.664 | 7.661 |
| 115701 | ALPK2 | -0.476 | 0.029 | 0.009 | down-reg. | 6.995 | 6.980 | 7.392 | 7.535 |
| 116441 | TM4SF18 | -0.581 | 0.026 | 0.004 | down-reg. | 6.791 | 6.823 | 7.470 | 7.306 |
| 116496 | NIBAN1 | -0.436 | 0.041 | 0.012 | down-reg. | 7.563 | 7.486 | 8.030 | 7.892 |
| 117248 | GALNT15 | -0.457 | 0.049 | 0.010 | down-reg. | 8.150 | 7.971 | 8.540 | 8.496 |
| 118980 | SFXN2 | -0.332 | 0.000 | 0.031 | down-reg. | 7.605 | 7.607 | 7.933 | 7.943 |
| 120224 | TMEM45B | -0.297 | 0.029 | 0.044 | down-reg. | 5.229 | 5.308 | 5.585 | 5.545 |
| 120379 | PIH1D2 | -0.527 | 0.044 | 0.006 | down-reg. | 5.178 | 5.168 | 5.601 | 5.798 |
| 121214 | SDR9C7 | -0.381 | 0.032 | 0.020 | down-reg. | 5.274 | 5.190 | 5.657 | 5.570 |
| 124637 | CYB5D1 | -0.384 | 0.026 | 0.019 | down-reg. | 8.910 | 8.843 | 9.303 | 9.217 |
| 124961 | ZFP3 | -0.320 | 0.031 | 0.034 | down-reg. | 7.321 | 7.234 | 7.622 | 7.573 |
| 124975 | GGT6 | -0.250 | 0.021 | 0.070 | down-reg. | 5.902 | 5.841 | 6.112 | 6.130 |
| 130367 | SGPP2 | -0.220 | 0.039 | 0.096 | down-reg. | 5.473 | 5.538 | 5.704 | 5.747 |
| 131076 | CCDC58 | -0.262 | 0.011 | 0.062 | down-reg. | 9.803 | 9.758 | 10.049 | 10.035 |
| 133396 | IL31RA | -0.302 | 0.043 | 0.041 | down-reg. | 5.422 | 5.311 | 5.658 | 5.678 |
| 134265 | AFAP1L1 | -0.426 | 0.015 | 0.013 | down-reg. | 7.284 | 7.204 | 7.647 | 7.694 |
| 134285 | TMEM171 | -0.360 | 0.008 | 0.024 | down-reg. | 7.792 | 7.844 | 8.165 | 8.192 |
| 135293 | PM20D2 | -0.433 | 0.038 | 0.012 | down-reg. | 9.504 | 9.619 | 10.043 | 9.947 |
| 137835 | TMEM71 | -0.539 | 0.036 | 0.006 | down-reg. | 5.170 | 5.005 | 5.590 | 5.663 |
| 147040 | KCTD11 | -0.318 | 0.022 | 0.035 | down-reg. | 7.598 | 7.682 | 7.960 | 7.957 |
| 147372 | CCBE1 | -0.269 | 0.025 | 0.057 | down-reg. | 10.424 | 10.429 | 10.658 | 10.734 |
| 147495 | APCDD1 | -0.288 | 0.050 | 0.048 | down-reg. | 8.700 | 8.717 | 9.054 | 8.939 |
| 148103 | ZNF599 | -0.355 | 0.030 | 0.025 | down-reg. | 6.391 | 6.474 | 6.753 | 6.824 |
| 148304 | C1orf74 | -0.277 | 0.004 | 0.053 | down-reg. | 7.963 | 7.945 | 8.217 | 8.245 |
| 148523 | CIART | -0.488 | 0.042 | 0.008 | down-reg. | 7.334 | 7.272 | 7.707 | 7.876 |
| 148741 | ANKRD35 | -0.353 | 0.007 | 0.025 | down-reg. | 5.899 | 5.951 | 6.284 | 6.273 |
| 151887 | CCDC80 | -0.639 | 0.002 | 0.003 | down-reg. | 10.545 | 10.581 | 11.180 | 11.224 |
| 154007 | SNRNP48 | -0.259 | 0.035 | 0.063 | down-reg. | 8.635 | 8.722 | 8.935 | 8.941 |
| 154141 | MBOAT1 | -0.335 | 0.016 | 0.030 | down-reg. | 7.999 | 8.055 | 8.386 | 8.339 |
| 158471 | PRUNE2 | -0.368 | 0.038 | 0.022 | down-reg. | 6.739 | 6.786 | 7.190 | 7.071 |
| 165055 | CCDC138 | -0.242 | 0.021 | 0.075 | down-reg. | 6.395 | 6.360 | 6.645 | 6.594 |
| 170384 | FUT11 | -0.438 | 0.006 | 0.012 | down-reg. | 9.684 | 9.742 | 10.156 | 10.145 |
| 195828 | ZNF367 | -0.434 | 0.043 | 0.012 | down-reg. | 8.264 | 8.162 | 8.709 | 8.584 |
| 200845 | KCTD6 | -0.430 | 0.013 | 0.013 | down-reg. | 7.708 | 7.668 | 8.155 | 8.080 |
| 200879 | LIPH | -0.615 | 0.005 | 0.003 | down-reg. | 4.824 | 4.835 | 5.405 | 5.485 |
| 200909 | HTR3D | -0.282 | 0.017 | 0.050 | down-reg. | 5.416 | 5.354 | 5.675 | 5.657 |
| 203245 | NAIF1 | -0.313 | 0.021 | 0.037 | down-reg. | 7.196 | 7.118 | 7.461 | 7.480 |
| 221786 | FAM200A | -0.254 | 0.035 | 0.067 | down-reg. | 7.385 | 7.458 | 7.653 | 7.697 |
| 221935 | SDK1 | -0.474 | 0.024 | 0.009 | down-reg. | 7.040 | 7.001 | 7.433 | 7.556 |
| 222256 | CDHR3 | -0.218 | 0.024 | 0.098 | down-reg. | 5.491 | 5.463 | 5.722 | 5.669 |
| 253017 | TECRL | -0.278 | 0.047 | 0.052 | down-reg. | 3.896 | 3.908 | 4.126 | 4.234 |
| 253190 | SERHL2 | -0.442 | 0.043 | 0.012 | down-reg. | 5.489 | 5.542 | 6.035 | 5.880 |
| 254427 | PROSER2 | -0.314 | 0.031 | 0.036 | down-reg. | 6.692 | 6.781 | 7.030 | 7.071 |
| 255488 | RNF144B | -0.389 | 0.037 | 0.018 | down-reg. | 6.590 | 6.709 | 7.009 | 7.067 |
| 256691 | MAMDC2 | -0.631 | 0.021 | 0.003 | down-reg. | 7.698 | 7.586 | 8.215 | 8.332 |
| 256714 | MAP7D2 | -0.229 | 0.046 | 0.088 | down-reg. | 5.085 | 5.158 | 5.375 | 5.325 |
| 284029 | LINC00324 | -0.532 | 0.038 | 0.006 | down-reg. | 4.598 | 4.613 | 5.230 | 5.044 |
| 284370 | ZNF615 | -0.247 | 0.010 | 0.071 | down-reg. | 7.527 | 7.490 | 7.743 | 7.768 |
| 285679 | AC270227.1 | -0.345 | 0.041 | 0.027 | down-reg. | 4.922 | 4.989 | 5.353 | 5.248 |
| 285966 | TCAF2 | -0.525 | 0.016 | 0.007 | down-reg. | 8.480 | 8.401 | 9.009 | 8.921 |
| 339778 | FAM166C | -0.257 | 0.017 | 0.065 | down-reg. | 6.227 | 6.239 | 6.461 | 6.518 |
| 347527 | ARSH | -0.267 | 0.005 | 0.058 | down-reg. | 5.612 | 5.581 | 5.859 | 5.869 |
| 347862 | GATD1 | -0.245 | 0.040 | 0.073 | down-reg. | 7.863 | 7.844 | 8.141 | 8.055 |
| 352999 | C6orf58 | -0.335 | 0.042 | 0.030 | down-reg. | 5.082 | 5.159 | 5.408 | 5.504 |
| 387032 | ZKSCAN4 | -0.257 | 0.011 | 0.065 | down-reg. | 7.024 | 7.025 | 7.258 | 7.305 |
| 387700 | SLC16A12 | -0.581 | 0.022 | 0.004 | down-reg. | 5.332 | 5.321 | 5.831 | 5.984 |
| 388566 | ZNF470 | -0.887 | 0.008 | 0.001 | down-reg. | 8.189 | 8.325 | 9.137 | 9.151 |
| 389741 | GLIDR | -0.768 | 0.013 | 0.001 | down-reg. | 4.524 | 4.678 | 5.365 | 5.373 |
| 389792 | IER5L | -0.221 | 0.010 | 0.096 | down-reg. | 7.970 | 7.937 | 8.184 | 8.164 |
| 390535 | GOLGA8EP | -1.324 | 0.011 | 0.000 | down-reg. | 5.615 | 5.516 | 7.003 | 6.776 |
| 391121 | OR10J4 | -0.521 | 0.032 | 0.007 | down-reg. | 5.919 | 5.937 | 6.366 | 6.531 |
| 392255 | GDF6 | -0.679 | 0.009 | 0.002 | down-reg. | 6.471 | 6.417 | 7.073 | 7.173 |
| 399669 | ZNF321P | -0.528 | 0.038 | 0.006 | down-reg. | 8.095 | 8.067 | 8.518 | 8.701 |
| 401152 | C4orf3 | -0.595 | 0.018 | 0.004 | down-reg. | 7.032 | 6.989 | 7.539 | 7.671 |
| 406881 | MIRLET7A1 | -0.245 | 0.043 | 0.073 | down-reg. | 6.319 | 6.402 | 6.587 | 6.624 |
| 406960 | MIR184 | -0.228 | 0.023 | 0.088 | down-reg. | 4.333 | 4.383 | 4.569 | 4.603 |
| 407033 | MIR30D | -0.423 | 0.043 | 0.013 | down-reg. | 3.454 | 3.588 | 3.904 | 3.985 |
| 440695 | ETV3L | -0.349 | 0.008 | 0.026 | down-reg. | 5.786 | 5.791 | 6.166 | 6.109 |
| 441525 | SPANXN4 | -0.373 | 0.025 | 0.021 | down-reg. | 5.572 | 5.583 | 6.002 | 5.898 |
| 677882 | SNORA59A | -0.263 | 0.018 | 0.061 | down-reg. | 6.842 | 6.892 | 7.112 | 7.148 |
| 692092 | SNORD32B | -0.317 | 0.046 | 0.035 | down-reg. | 6.563 | 6.637 | 6.868 | 6.965 |
| 692148 | SCARNA10 | -0.288 | 0.022 | 0.048 | down-reg. | 8.313 | 8.248 | 8.549 | 8.587 |
| 790955 | UQCC3 | -0.326 | 0.041 | 0.033 | down-reg. | 7.779 | 7.660 | 8.044 | 8.048 |
| 100101267 | POM121C | -0.509 | 0.027 | 0.007 | down-reg. | 8.969 | 8.846 | 9.458 | 9.375 |
| 100131211 | NEMP2 | -0.360 | 0.011 | 0.024 | down-reg. | 6.558 | 6.623 | 6.957 | 6.945 |
| 100151684 | RNU6ATAC | -0.252 | 0.042 | 0.068 | down-reg. | 6.787 | 6.745 | 7.058 | 6.976 |
| 100272147 | CMC4 | -0.350 | 0.016 | 0.026 | down-reg. | 8.049 | 7.979 | 8.349 | 8.380 |
| 100506127 | GVQW3 | -0.281 | 0.045 | 0.051 | down-reg. | 5.801 | 5.741 | 6.008 | 6.097 |
| 101447996 | RNVU1-8 | -0.336 | 0.032 | 0.030 | down-reg. | 5.900 | 5.806 | 6.164 | 6.214 |
