# Supplementary material for: Histone deacetylase 4 reverses cellular senescence via DDIT4 in dermal fibroblasts
Source: Aging (Albany NY). 2022 Jun 9;14(11):4653–72. doi: 10.18632/aging.204118 (PMC9217707; doi:10.18632/aging.204118)
Supplement: Supplementary Table 4 [file aging-14-204118-s005.docx]

Supplementary Table 4. Transcriptome analysis of H_2_O_2_-induced senescence HDFs.

| **Entrez ID** | **Symbol** | **log_2_(*FC*) [H_2_O_2_ / control]** | **Pt [H_2_O_2_ / control]** | **Pf [H_2_O_2_ / control]** | **DEGs [H_2_O_2_ / control]** | **H_2_O_2_ sample1** | **H_2_O_2_ sample2** | **H_2_O_2_ sample3** | **control sample1** | **control sample2** | **control sample3** |
| --- | --- | --- | --- | --- | --- | --- | --- | --- | --- | --- | --- |
| 24 | ABCA4 | 0.931 | 0.041 | 0.040 | up-reg. | 8.655 | 8.455 | 8.651 | 7.077 | 7.938 | 7.955 |
| 27 | ABL2 | 1.656 | 0.009 | 0.005 | up-reg. | 6.438 | 6.058 | 6.340 | 4.223 | 4.673 | 4.972 |
| 55 | ACP3 | 2.245 | 0.000 | 0.001 | up-reg. | 5.667 | 5.505 | 5.648 | 3.282 | 3.453 | 3.350 |
| 81 | ACTN4 | 1.429 | 0.003 | 0.008 | up-reg. | 8.219 | 7.921 | 8.211 | 6.607 | 6.810 | 6.647 |
| 103 | ADAR | 0.901 | 0.017 | 0.045 | up-reg. | 8.851 | 8.488 | 8.721 | 7.472 | 7.995 | 7.890 |
| 133 | ADM | 0.731 | 0.007 | 0.087 | up-reg. | 15.007 | 15.268 | 15.043 | 14.469 | 14.318 | 14.337 |
| 148 | ADRA1A | 0.886 | 0.041 | 0.048 | up-reg. | 6.656 | 6.525 | 6.776 | 5.224 | 6.094 | 5.981 |
| 154 | ADRB2 | 2.566 | 0.002 | 0.001 | up-reg. | 7.243 | 7.393 | 6.926 | 4.552 | 4.508 | 4.806 |
| 205 | AK4 | 0.760 | 0.009 | 0.078 | up-reg. | 7.120 | 7.073 | 7.399 | 6.458 | 6.339 | 6.516 |
| 231 | AKR1B1 | 1.648 | 0.002 | 0.005 | up-reg. | 13.011 | 13.142 | 13.370 | 11.522 | 11.587 | 11.470 |
| 240 | ALOX5 | 1.790 | 0.012 | 0.003 | up-reg. | 8.196 | 7.466 | 8.251 | 5.930 | 6.045 | 6.567 |
| 303 | 0 | 0.806 | 0.033 | 0.065 | up-reg. | 5.932 | 5.861 | 6.227 | 4.789 | 5.361 | 5.452 |
| 310 | ANXA7 | 0.713 | 0.022 | 0.093 | up-reg. | 5.154 | 5.030 | 4.822 | 4.012 | 4.358 | 4.496 |
| 317 | APAF1 | 1.130 | 0.039 | 0.019 | up-reg. | 8.887 | 8.650 | 8.636 | 6.919 | 7.811 | 8.052 |
| 321 | APBA2 | 0.825 | 0.011 | 0.061 | up-reg. | 6.217 | 6.145 | 6.058 | 5.473 | 5.048 | 5.424 |
| 333 | APLP1 | 1.481 | 0.015 | 0.007 | up-reg. | 5.869 | 5.917 | 5.595 | 4.618 | 3.746 | 4.573 |
| 389 | RHOC | 1.175 | 0.010 | 0.017 | up-reg. | 7.095 | 6.707 | 7.260 | 5.693 | 5.826 | 6.017 |
| 487 | ATP2A1 | 1.002 | 0.015 | 0.031 | up-reg. | 10.107 | 9.867 | 10.161 | 8.851 | 8.880 | 9.395 |
| 490 | ATP2B1 | 0.936 | 0.024 | 0.039 | up-reg. | 5.588 | 5.423 | 5.564 | 4.163 | 4.662 | 4.942 |
| 522 | ATP5PF | 0.780 | 0.035 | 0.072 | up-reg. | 5.641 | 5.342 | 5.531 | 4.977 | 4.292 | 4.905 |
| 567 | B2M | 1.118 | 0.046 | 0.020 | up-reg. | 6.033 | 5.963 | 5.704 | 4.922 | 4.093 | 5.330 |
| 624 | BDKRB2 | 1.605 | 0.015 | 0.005 | up-reg. | 6.397 | 6.274 | 6.128 | 4.123 | 4.656 | 5.203 |
| 670 | BPHL | 0.721 | 0.008 | 0.090 | up-reg. | 6.313 | 6.188 | 6.027 | 5.540 | 5.344 | 5.480 |
| 676 | BRDT | 0.835 | 0.043 | 0.058 | up-reg. | 4.790 | 4.449 | 4.446 | 4.197 | 3.626 | 3.357 |
| 684 | BST2 | 4.598 | 0.001 | 0.000 | up-reg. | 9.915 | 9.966 | 9.920 | 5.194 | 5.000 | 5.813 |
| 686 | BTD | 1.100 | 0.010 | 0.022 | up-reg. | 9.634 | 9.403 | 9.817 | 8.358 | 8.787 | 8.408 |
| 770 | CA11 | 1.653 | 0.002 | 0.005 | up-reg. | 9.164 | 9.237 | 9.023 | 7.361 | 7.628 | 7.478 |
| 773 | CACNA1A | 1.072 | 0.020 | 0.024 | up-reg. | 5.230 | 4.626 | 5.152 | 4.237 | 3.842 | 3.711 |
| 783 | CACNB2 | 0.824 | 0.006 | 0.061 | up-reg. | 5.999 | 5.943 | 5.960 | 5.152 | 4.970 | 5.307 |
| 857 | CAV1 | 0.761 | 0.012 | 0.077 | up-reg. | 5.987 | 5.858 | 5.813 | 4.962 | 5.040 | 5.373 |
| 873 | CBR1 | 1.151 | 0.023 | 0.018 | up-reg. | 6.932 | 6.558 | 6.787 | 5.174 | 5.566 | 6.085 |
| 881 | CCIN | 0.825 | 0.019 | 0.061 | up-reg. | 5.955 | 5.749 | 5.663 | 4.679 | 4.962 | 5.250 |
| 894 | CCND2 | 1.778 | 0.009 | 0.004 | up-reg. | 4.731 | 3.816 | 4.291 | 2.384 | 2.574 | 2.548 |
| 948 | CD36 | 0.909 | 0.008 | 0.044 | up-reg. | 6.090 | 6.495 | 6.357 | 5.297 | 5.499 | 5.420 |
| 951 | CD37 | 2.046 | 0.013 | 0.002 | up-reg. | 6.566 | 5.931 | 6.246 | 3.669 | 4.791 | 4.147 |
| 987 | LRBA | 0.945 | 0.015 | 0.038 | up-reg. | 4.656 | 4.630 | 5.028 | 4.046 | 3.569 | 3.863 |
| 1021 | CDK6 | 1.222 | 0.008 | 0.014 | up-reg. | 9.362 | 9.023 | 9.307 | 7.864 | 8.287 | 7.874 |
| 1044 | CDX1 | 1.228 | 0.021 | 0.014 | up-reg. | 6.336 | 6.378 | 5.882 | 5.047 | 4.528 | 5.337 |
| 1050 | CEBPA | 1.116 | 0.007 | 0.021 | up-reg. | 8.070 | 8.078 | 7.835 | 6.878 | 6.658 | 7.099 |
| 1062 | CENPE | 0.775 | 0.029 | 0.073 | up-reg. | 6.637 | 6.565 | 6.364 | 5.376 | 5.816 | 6.049 |
| 1140 | CHRNB1 | 1.226 | 0.012 | 0.014 | up-reg. | 5.736 | 5.621 | 6.109 | 4.541 | 4.354 | 4.891 |
| 1176 | AP3S1 | 0.906 | 0.040 | 0.044 | up-reg. | 4.849 | 4.610 | 5.342 | 3.726 | 4.384 | 3.973 |
| 1263 | PLK3 | 0.722 | 0.009 | 0.090 | up-reg. | 9.897 | 10.145 | 9.910 | 9.280 | 9.120 | 9.388 |
| 1295 | COL8A1 | 1.878 | 0.002 | 0.003 | up-reg. | 7.507 | 7.515 | 7.303 | 5.472 | 5.494 | 5.726 |
| 1373 | CPS1 | 1.363 | 0.020 | 0.010 | up-reg. | 11.488 | 11.305 | 11.552 | 9.507 | 10.207 | 10.542 |
| 1381 | CRABP1 | 3.469 | 0.002 | 0.000 | up-reg. | 6.406 | 6.375 | 5.933 | 2.930 | 2.493 | 2.885 |
| 1388 | ATF6B | 0.754 | 0.039 | 0.080 | up-reg. | 4.325 | 4.109 | 4.533 | 3.700 | 3.173 | 3.833 |
| 1404 | HAPLN1 | 1.175 | 0.014 | 0.017 | up-reg. | 5.027 | 4.798 | 5.396 | 3.626 | 3.924 | 4.146 |
| 1414 | CRYBB1 | 1.206 | 0.027 | 0.015 | up-reg. | 8.774 | 8.675 | 8.955 | 7.048 | 8.126 | 7.613 |
| 1432 | MAPK14 | 0.877 | 0.013 | 0.050 | up-reg. | 5.934 | 5.534 | 5.791 | 4.665 | 4.897 | 5.067 |
| 1436 | CSF1R | 1.643 | 0.024 | 0.005 | up-reg. | 6.555 | 6.344 | 6.543 | 4.157 | 4.776 | 5.580 |
| 1453 | CSNK1D | 0.955 | 0.013 | 0.037 | up-reg. | 6.836 | 6.556 | 6.723 | 6.046 | 5.504 | 5.703 |
| 1459 | CSNK2A2 | 0.842 | 0.025 | 0.057 | up-reg. | 3.923 | 3.854 | 4.454 | 3.177 | 3.082 | 3.447 |
| 1490 | CCN2 | 2.973 | 0.001 | 0.000 | up-reg. | 11.918 | 12.199 | 11.931 | 9.206 | 8.827 | 9.097 |
| 1508 | CTSB | 0.763 | 0.014 | 0.077 | up-reg. | 8.082 | 7.884 | 8.124 | 7.204 | 7.514 | 7.084 |
| 1509 | CTSD | 1.138 | 0.006 | 0.019 | up-reg. | 5.766 | 5.599 | 5.701 | 4.771 | 4.346 | 4.535 |
| 1616 | DAXX | 1.515 | 0.015 | 0.007 | up-reg. | 11.438 | 11.426 | 11.676 | 9.434 | 10.186 | 10.374 |
| 1634 | DCN | 1.142 | 0.021 | 0.019 | up-reg. | 7.976 | 7.962 | 8.307 | 6.623 | 7.410 | 6.784 |
| 1638 | DCT | 1.401 | 0.014 | 0.009 | up-reg. | 9.569 | 9.472 | 9.891 | 7.780 | 8.519 | 8.430 |
| 1647 | GADD45A | 1.777 | 0.013 | 0.004 | up-reg. | 6.210 | 6.128 | 7.160 | 4.768 | 4.701 | 4.699 |
| 1776 | DNASE1L3 | 0.909 | 0.015 | 0.044 | up-reg. | 6.455 | 5.986 | 6.500 | 5.336 | 5.306 | 5.570 |
| 1801 | DPH1 | 0.836 | 0.022 | 0.058 | up-reg. | 7.047 | 6.891 | 6.916 | 5.927 | 5.915 | 6.504 |
| 1820 | ARID3A | 0.781 | 0.040 | 0.072 | up-reg. | 6.260 | 6.313 | 6.375 | 5.561 | 5.101 | 5.946 |
| 1843 | DUSP1 | 2.335 | 0.006 | 0.001 | up-reg. | 7.096 | 7.089 | 6.969 | 4.908 | 4.176 | 5.065 |
| 1901 | S1PR1 | 0.879 | 0.041 | 0.049 | up-reg. | 4.976 | 4.549 | 4.182 | 3.401 | 3.699 | 3.969 |
| 1903 | S1PR3 | 1.094 | 0.003 | 0.022 | up-reg. | 11.296 | 11.506 | 11.305 | 10.386 | 10.164 | 10.276 |
| 1909 | EDNRA | 0.926 | 0.040 | 0.041 | up-reg. | 6.757 | 6.508 | 6.913 | 5.271 | 5.988 | 6.140 |
| 1946 | EFNA5 | 1.361 | 0.010 | 0.010 | up-reg. | 6.410 | 6.017 | 6.250 | 4.521 | 4.903 | 5.172 |
| 1956 | EGFR | 0.732 | 0.023 | 0.086 | up-reg. | 6.036 | 5.558 | 5.457 | 4.914 | 4.963 | 4.977 |
| 1958 | EGR1 | 1.191 | 0.007 | 0.016 | up-reg. | 8.430 | 8.871 | 8.455 | 7.453 | 7.269 | 7.462 |
| 2055 | CLN8 | 1.366 | 0.015 | 0.010 | up-reg. | 5.034 | 4.428 | 5.193 | 3.497 | 3.274 | 3.786 |
| 2064 | ERBB2 | 0.990 | 0.036 | 0.032 | up-reg. | 7.816 | 7.575 | 6.874 | 6.431 | 6.610 | 6.256 |
| 2081 | ERN1 | 0.709 | 0.017 | 0.095 | up-reg. | 6.281 | 6.488 | 6.112 | 5.518 | 5.792 | 5.444 |
| 2101 | ESRRA | 1.327 | 0.006 | 0.011 | up-reg. | 6.386 | 6.498 | 6.079 | 4.874 | 4.909 | 5.201 |
| 2103 | ESRRB | 0.730 | 0.031 | 0.087 | up-reg. | 6.154 | 5.892 | 5.814 | 4.938 | 5.182 | 5.550 |
| 2113 | ETS1 | 0.870 | 0.027 | 0.051 | up-reg. | 9.091 | 8.590 | 8.337 | 7.937 | 7.737 | 7.734 |
| 2118 | ETV4 | 0.864 | 0.037 | 0.052 | up-reg. | 8.810 | 8.923 | 9.015 | 7.595 | 8.486 | 8.077 |
| 2152 | F3 | 1.812 | 0.019 | 0.003 | up-reg. | 12.645 | 12.436 | 12.774 | 10.065 | 10.959 | 11.395 |
| 2191 | FAP | 1.433 | 0.014 | 0.008 | up-reg. | 6.934 | 7.112 | 7.127 | 5.376 | 5.331 | 6.168 |
| 2213 | FCGR2B | 0.944 | 0.016 | 0.038 | up-reg. | 8.059 | 7.771 | 8.030 | 6.726 | 7.321 | 6.980 |
| 2230 | FDX1 | 1.252 | 0.017 | 0.013 | up-reg. | 5.974 | 6.041 | 6.090 | 4.529 | 4.509 | 5.311 |
| 2254 | FGF9 | 0.949 | 0.020 | 0.038 | up-reg. | 8.400 | 8.211 | 8.340 | 6.947 | 7.550 | 7.608 |
| 2294 | FOXF1 | 1.321 | 0.002 | 0.011 | up-reg. | 10.084 | 10.300 | 10.091 | 8.871 | 8.719 | 8.920 |
| 2297 | FOXD1 | 0.846 | 0.023 | 0.056 | up-reg. | 3.965 | 3.742 | 3.819 | 3.382 | 2.885 | 2.721 |
| 2321 | FLT1 | 1.489 | 0.007 | 0.007 | up-reg. | 7.332 | 7.546 | 7.067 | 5.544 | 6.034 | 5.900 |
| 2339 | FNTA | 1.134 | 0.016 | 0.019 | up-reg. | 7.063 | 7.067 | 7.275 | 5.571 | 6.131 | 6.302 |
| 2352 | FOLR3 | 4.622 | 0.000 | 0.000 | up-reg. | 10.682 | 10.603 | 10.575 | 5.821 | 5.877 | 6.297 |
| 2353 | FOS | 1.574 | 0.023 | 0.006 | up-reg. | 6.482 | 6.677 | 5.846 | 4.949 | 4.171 | 5.164 |
| 2495 | FTH1 | 2.434 | 0.007 | 0.001 | up-reg. | 10.967 | 10.061 | 9.965 | 8.012 | 7.785 | 7.893 |
| 2558 | GABRA5 | 1.311 | 0.047 | 0.011 | up-reg. | 6.138 | 5.953 | 5.824 | 5.451 | 3.936 | 4.594 |
| 2565 | GABRG1 | 1.431 | 0.018 | 0.008 | up-reg. | 9.518 | 9.025 | 9.475 | 7.380 | 8.282 | 8.062 |
| 2571 | GAD1 | 0.970 | 0.030 | 0.035 | up-reg. | 5.670 | 5.701 | 5.408 | 4.120 | 4.825 | 4.925 |
| 2618 | GART | 1.384 | 0.011 | 0.009 | up-reg. | 12.388 | 12.325 | 12.309 | 10.500 | 11.228 | 11.142 |
| 2619 | GAS1 | 2.200 | 0.004 | 0.002 | up-reg. | 8.273 | 8.122 | 8.001 | 6.040 | 5.542 | 6.214 |
| 2626 | GATA4 | 1.805 | 0.030 | 0.003 | up-reg. | 6.695 | 5.878 | 6.525 | 4.023 | 4.232 | 5.428 |
| 2627 | GATA6 | 1.086 | 0.039 | 0.023 | up-reg. | 5.521 | 5.559 | 6.298 | 4.460 | 4.493 | 5.166 |
| 2668 | GDNF | 0.771 | 0.030 | 0.075 | up-reg. | 6.013 | 5.733 | 5.697 | 5.022 | 4.725 | 5.383 |
| 2675 | GFRA2 | 1.102 | 0.013 | 0.022 | up-reg. | 8.361 | 7.996 | 8.183 | 7.000 | 7.417 | 6.818 |
| 2690 | GHR | 0.776 | 0.030 | 0.073 | up-reg. | 7.552 | 7.434 | 7.642 | 6.367 | 7.072 | 6.860 |
| 2745 | GLRX | 0.861 | 0.010 | 0.053 | up-reg. | 7.374 | 7.433 | 7.519 | 6.808 | 6.362 | 6.572 |
| 2769 | GNA15 | 1.324 | 0.015 | 0.011 | up-reg. | 6.681 | 6.579 | 6.166 | 4.913 | 4.966 | 5.575 |
| 2781 | GNAZ | 1.353 | 0.004 | 0.010 | up-reg. | 10.575 | 10.788 | 10.349 | 9.300 | 9.206 | 9.148 |
| 2824 | GPM6B | 0.765 | 0.028 | 0.076 | up-reg. | 7.281 | 7.403 | 7.060 | 6.192 | 6.816 | 6.439 |
| 2827 | GPR3 | 1.010 | 0.022 | 0.030 | up-reg. | 8.494 | 8.020 | 8.558 | 7.159 | 7.182 | 7.701 |
| 2876 | GPX1 | 1.323 | 0.027 | 0.011 | up-reg. | 10.422 | 10.147 | 10.433 | 8.331 | 9.374 | 9.328 |
| 2877 | GPX2 | 1.267 | 0.020 | 0.013 | up-reg. | 6.554 | 6.356 | 6.735 | 4.844 | 5.225 | 5.775 |
| 2894 | GRID1 | 1.932 | 0.013 | 0.003 | up-reg. | 9.826 | 9.460 | 9.838 | 7.133 | 8.225 | 7.969 |
| 2919 | CXCL1 | 10.002 | 0.000 | 0.000 | up-reg. | 16.520 | 16.628 | 16.323 | 6.579 | 6.368 | 6.518 |
| 2971 | GTF3A | 0.840 | 0.044 | 0.057 | up-reg. | 6.934 | 6.790 | 6.842 | 5.638 | 5.858 | 6.549 |
| 2977 | GUCY1A2 | 2.726 | 0.002 | 0.001 | up-reg. | 6.541 | 6.464 | 6.385 | 3.482 | 3.668 | 4.063 |
| 2980 | GUCA2A | 1.344 | 0.014 | 0.010 | up-reg. | 10.720 | 10.204 | 10.941 | 9.144 | 9.562 | 9.126 |
| 2992 | GYG1 | 0.906 | 0.020 | 0.045 | up-reg. | 7.531 | 7.663 | 7.484 | 6.360 | 7.037 | 6.564 |
| 3060 | HCRT | 1.512 | 0.012 | 0.007 | up-reg. | 10.210 | 9.688 | 10.441 | 8.455 | 8.903 | 8.444 |
| 3075 | CFH | 1.006 | 0.004 | 0.030 | up-reg. | 6.050 | 6.063 | 6.260 | 5.095 | 5.000 | 5.260 |
| 3084 | NRG1 | 1.128 | 0.003 | 0.020 | up-reg. | 6.350 | 6.539 | 6.278 | 5.330 | 5.183 | 5.270 |
| 3140 | MR1 | 1.066 | 0.023 | 0.025 | up-reg. | 7.715 | 8.018 | 7.368 | 6.727 | 6.283 | 6.893 |
| 3198 | HOXA1 | 0.955 | 0.023 | 0.037 | up-reg. | 7.428 | 7.126 | 7.529 | 6.014 | 6.496 | 6.707 |
| 3201 | HOXA4 | 1.454 | 0.018 | 0.008 | up-reg. | 4.796 | 4.665 | 3.914 | 2.740 | 2.969 | 3.304 |
| 3204 | HOXA7 | 0.965 | 0.016 | 0.035 | up-reg. | 6.035 | 5.730 | 5.764 | 4.613 | 4.811 | 5.209 |
| 3214 | HOXB4 | 0.867 | 0.030 | 0.052 | up-reg. | 5.421 | 4.657 | 5.107 | 4.107 | 4.092 | 4.385 |
| 3234 | HOXD8 | 1.139 | 0.024 | 0.019 | up-reg. | 5.552 | 4.929 | 4.829 | 3.807 | 3.774 | 4.311 |
| 3237 | HOXD11 | 0.918 | 0.003 | 0.042 | up-reg. | 6.636 | 6.690 | 6.517 | 5.589 | 5.701 | 5.798 |
| 3305 | HSPA1L | 1.293 | 0.011 | 0.012 | up-reg. | 6.503 | 6.614 | 6.146 | 5.260 | 4.797 | 5.328 |
| 3308 | HSPA4 | 0.709 | 0.037 | 0.095 | up-reg. | 5.833 | 5.624 | 5.374 | 4.561 | 5.115 | 5.029 |
| 3320 | HSP90AA1 | 0.947 | 0.036 | 0.038 | up-reg. | 6.393 | 6.394 | 6.202 | 5.895 | 4.935 | 5.319 |
| 3423 | IDS | 0.858 | 0.021 | 0.053 | up-reg. | 6.250 | 6.449 | 5.951 | 5.269 | 5.172 | 5.635 |
| 3487 | IGFBP4 | 0.941 | 0.008 | 0.039 | up-reg. | 16.628 | 16.323 | 16.628 | 15.745 | 15.465 | 15.546 |
| 3490 | IGFBP7 | 2.875 | 0.003 | 0.001 | up-reg. | 6.539 | 6.477 | 6.417 | 3.626 | 3.207 | 3.974 |
| 3549 | IHH | 1.447 | 0.045 | 0.008 | up-reg. | 4.179 | 4.350 | 5.408 | 3.158 | 2.713 | 3.727 |
| 3557 | IL1RN | 0.986 | 0.011 | 0.033 | up-reg. | 8.722 | 8.313 | 8.629 | 7.333 | 7.715 | 7.659 |
| 3575 | IL7R | 1.162 | 0.012 | 0.017 | up-reg. | 9.155 | 9.070 | 9.001 | 7.520 | 8.031 | 8.187 |
| 3577 | CXCR1 | 1.516 | 0.008 | 0.007 | up-reg. | 12.594 | 12.232 | 12.636 | 10.715 | 10.895 | 11.305 |
| 3579 | CXCR2 | 1.391 | 0.018 | 0.009 | up-reg. | 7.959 | 7.559 | 8.117 | 6.006 | 6.864 | 6.594 |
| 3597 | IL13RA1 | 1.386 | 0.016 | 0.009 | up-reg. | 8.842 | 8.174 | 8.081 | 6.696 | 7.219 | 7.025 |
| 3635 | INPP5D | 2.807 | 0.000 | 0.001 | up-reg. | 7.826 | 7.807 | 7.648 | 4.949 | 5.022 | 4.890 |
| 3670 | ISL1 | 1.999 | 0.001 | 0.002 | up-reg. | 6.540 | 6.395 | 6.458 | 4.553 | 4.312 | 4.532 |
| 3672 | ITGA1 | 1.480 | 0.013 | 0.007 | up-reg. | 7.055 | 7.017 | 6.843 | 5.629 | 4.974 | 5.872 |
| 3787 | KCNS1 | 1.157 | 0.017 | 0.018 | up-reg. | 11.771 | 11.347 | 11.611 | 10.544 | 10.001 | 10.711 |
| 3790 | KCNS3 | 1.396 | 0.015 | 0.009 | up-reg. | 5.358 | 4.796 | 4.488 | 3.661 | 3.307 | 3.486 |
| 3796 | KIF2A | 1.316 | 0.046 | 0.011 | up-reg. | 10.141 | 9.457 | 10.788 | 8.579 | 9.259 | 8.600 |
| 3884 | KRT33B | 2.466 | 0.007 | 0.001 | up-reg. | 7.633 | 7.395 | 7.513 | 4.996 | 4.510 | 5.635 |
| 3948 | LDHC | 1.509 | 0.011 | 0.007 | up-reg. | 5.088 | 5.051 | 4.945 | 3.371 | 4.014 | 3.173 |
| 4041 | LRP5 | 1.016 | 0.006 | 0.029 | up-reg. | 8.855 | 9.049 | 8.658 | 7.884 | 7.877 | 7.751 |
| 4082 | MARCKS | 1.333 | 0.018 | 0.011 | up-reg. | 6.294 | 5.840 | 6.318 | 4.695 | 4.472 | 5.286 |
| 4090 | SMAD5 | 0.750 | 0.046 | 0.081 | up-reg. | 5.751 | 5.771 | 5.959 | 4.612 | 5.178 | 5.441 |
| 4115 | MAGEB4 | 1.497 | 0.010 | 0.007 | up-reg. | 7.703 | 7.184 | 7.572 | 5.643 | 6.031 | 6.293 |
| 4135 | MAP6 | 2.018 | 0.012 | 0.002 | up-reg. | 6.547 | 6.706 | 6.267 | 4.385 | 3.977 | 5.104 |
| 4142 | MAS1 | 1.384 | 0.006 | 0.009 | up-reg. | 4.481 | 4.072 | 4.454 | 2.755 | 3.032 | 3.068 |
| 4176 | MCM7 | 0.960 | 0.006 | 0.036 | up-reg. | 7.135 | 6.837 | 7.193 | 6.041 | 6.147 | 6.098 |
| 4188 | MDFI | 0.700 | 0.024 | 0.098 | up-reg. | 10.445 | 10.316 | 10.664 | 9.551 | 9.725 | 10.048 |
| 4217 | MAP3K5 | 1.606 | 0.041 | 0.005 | up-reg. | 6.353 | 6.656 | 7.557 | 4.560 | 5.825 | 5.363 |
| 4248 | MGAT3 | 1.196 | 0.008 | 0.016 | up-reg. | 9.494 | 9.123 | 9.280 | 7.833 | 8.299 | 8.176 |
| 4249 | MGAT5 | 2.072 | 0.001 | 0.002 | up-reg. | 12.037 | 12.205 | 12.080 | 10.169 | 9.871 | 10.065 |
| 4289 | MKLN1 | 1.124 | 0.025 | 0.020 | up-reg. | 8.408 | 8.396 | 8.508 | 6.783 | 7.763 | 7.395 |
| 4306 | NR3C2 | 1.154 | 0.027 | 0.018 | up-reg. | 4.163 | 4.432 | 3.933 | 3.439 | 3.117 | 2.508 |
| 4324 | MMP15 | 1.050 | 0.011 | 0.026 | up-reg. | 7.921 | 8.070 | 7.755 | 6.750 | 6.681 | 7.166 |
| 4335 | MNT | 1.191 | 0.033 | 0.016 | up-reg. | 11.904 | 11.721 | 12.037 | 10.028 | 11.087 | 10.974 |
| 4336 | MOBP | 0.799 | 0.020 | 0.067 | up-reg. | 5.405 | 5.068 | 5.492 | 4.265 | 4.609 | 4.693 |
| 4437 | MSH3 | 1.143 | 0.019 | 0.019 | up-reg. | 8.043 | 7.838 | 8.115 | 6.556 | 6.677 | 7.334 |
| 4489 | MT1A | 1.008 | 0.031 | 0.030 | up-reg. | 4.597 | 5.175 | 4.664 | 4.059 | 3.366 | 3.988 |
| 4490 | MT1B | 0.881 | 0.029 | 0.049 | up-reg. | 4.878 | 4.354 | 5.046 | 3.999 | 4.011 | 3.626 |
| 4494 | MT1F | 1.279 | 0.007 | 0.012 | up-reg. | 8.001 | 7.777 | 7.893 | 6.745 | 6.296 | 6.792 |
| 4495 | MT1G | 2.481 | 0.019 | 0.001 | up-reg. | 6.193 | 6.017 | 5.789 | 3.124 | 2.843 | 4.589 |
| 4500 | MT1L | 2.029 | 0.000 | 0.002 | up-reg. | 11.196 | 11.269 | 11.175 | 9.213 | 9.128 | 9.211 |
| 4502 | MT2A | 1.198 | 0.000 | 0.016 | up-reg. | 17.183 | 17.183 | 17.183 | 16.012 | 16.012 | 15.934 |
| 4664 | NAB1 | 1.799 | 0.001 | 0.003 | up-reg. | 11.152 | 11.078 | 10.995 | 9.338 | 9.162 | 9.330 |
| 4675 | NAP1L3 | 1.325 | 0.010 | 0.011 | up-reg. | 6.970 | 6.657 | 6.848 | 5.274 | 5.331 | 5.893 |
| 4680 | CEACAM6 | 1.338 | 0.009 | 0.010 | up-reg. | 9.189 | 8.649 | 8.982 | 7.331 | 7.744 | 7.730 |
| 4688 | NCF2 | 0.777 | 0.022 | 0.073 | up-reg. | 5.220 | 5.469 | 4.863 | 4.302 | 4.396 | 4.523 |
| 4762 | NEUROG1 | 1.585 | 0.036 | 0.005 | up-reg. | 5.581 | 4.545 | 4.423 | 2.664 | 3.445 | 3.687 |
| 4773 | NFATC2 | 1.005 | 0.035 | 0.030 | up-reg. | 9.213 | 8.837 | 9.518 | 8.517 | 8.289 | 7.747 |
| 4781 | NFIB | 0.923 | 0.005 | 0.042 | up-reg. | 5.503 | 5.282 | 5.489 | 4.351 | 4.574 | 4.582 |
| 4792 | NFKBIA | 2.819 | 0.001 | 0.001 | up-reg. | 12.703 | 12.864 | 12.601 | 9.988 | 9.734 | 9.988 |
| 4794 | NFKBIE | 1.401 | 0.002 | 0.009 | up-reg. | 10.361 | 10.462 | 10.207 | 9.059 | 8.955 | 8.814 |
| 4814 | NINJ1 | 2.123 | 0.001 | 0.002 | up-reg. | 13.782 | 13.893 | 13.752 | 11.810 | 11.727 | 11.522 |
| 4887 | NPY2R | 1.355 | 0.048 | 0.010 | up-reg. | 10.169 | 10.044 | 10.312 | 7.916 | 9.409 | 9.136 |
| 4916 | NTRK3 | 1.161 | 0.003 | 0.017 | up-reg. | 5.778 | 5.720 | 5.545 | 4.523 | 4.611 | 4.428 |
| 4938 | OAS1 | 3.741 | 0.001 | 0.000 | up-reg. | 8.763 | 8.889 | 8.787 | 4.864 | 5.386 | 4.966 |
| 4939 | OAS2 | 2.678 | 0.005 | 0.001 | up-reg. | 8.292 | 8.347 | 8.231 | 5.398 | 5.272 | 6.165 |
| 4956 | ODF1 | 1.183 | 0.015 | 0.016 | up-reg. | 11.442 | 11.050 | 11.416 | 9.739 | 10.409 | 10.210 |
| 5137 | PDE1C | 2.379 | 0.006 | 0.001 | up-reg. | 5.914 | 6.053 | 5.661 | 3.315 | 3.157 | 4.018 |
| 5144 | PDE4D | 0.795 | 0.048 | 0.068 | up-reg. | 10.983 | 10.983 | 11.269 | 9.818 | 10.698 | 10.331 |
| 5229 | PGGT1B | 1.251 | 0.015 | 0.013 | up-reg. | 5.039 | 4.375 | 4.714 | 3.718 | 3.179 | 3.478 |
| 5260 | PHKG1 | 0.924 | 0.005 | 0.041 | up-reg. | 9.419 | 9.231 | 9.576 | 8.419 | 8.538 | 8.494 |
| 5271 | SERPINB8 | 0.963 | 0.044 | 0.035 | up-reg. | 10.606 | 10.453 | 10.928 | 9.244 | 10.229 | 9.624 |
| 5297 | PI4KA | 1.365 | 0.018 | 0.010 | up-reg. | 7.352 | 7.043 | 7.155 | 5.261 | 6.189 | 6.006 |
| 5352 | PLOD2 | 0.872 | 0.038 | 0.051 | up-reg. | 7.553 | 7.431 | 7.493 | 6.094 | 6.820 | 6.950 |
| 5407 | PNLIPRP1 | 1.553 | 0.013 | 0.006 | up-reg. | 9.706 | 9.693 | 9.941 | 7.701 | 8.344 | 8.636 |
| 5452 | POU2F2 | 2.938 | 0.000 | 0.000 | up-reg. | 12.027 | 12.006 | 11.977 | 9.216 | 8.976 | 9.004 |
| 5498 | PPOX | 2.060 | 0.004 | 0.002 | up-reg. | 5.638 | 4.992 | 5.081 | 3.098 | 3.158 | 3.277 |
| 5507 | PPP1R3C | 1.091 | 0.001 | 0.022 | up-reg. | 11.853 | 11.909 | 11.816 | 10.836 | 10.682 | 10.787 |
| 5511 | PPP1R8 | 0.873 | 0.014 | 0.050 | up-reg. | 11.646 | 11.158 | 11.657 | 10.573 | 10.668 | 10.603 |
| 5519 | PPP2R1B | 1.581 | 0.010 | 0.005 | up-reg. | 6.964 | 7.127 | 7.488 | 5.633 | 5.264 | 5.937 |
| 5523 | PPP2R3A | 2.044 | 0.008 | 0.002 | up-reg. | 7.992 | 7.586 | 7.963 | 5.317 | 6.136 | 5.955 |
| 5564 | PRKAB1 | 1.731 | 0.011 | 0.004 | up-reg. | 6.364 | 5.925 | 5.543 | 4.218 | 3.896 | 4.526 |
| 5569 | PKIA | 1.922 | 0.018 | 0.003 | up-reg. | 5.612 | 5.530 | 4.621 | 3.822 | 2.864 | 3.310 |
| 5596 | MAPK4 | 1.416 | 0.007 | 0.009 | up-reg. | 10.306 | 9.971 | 10.453 | 8.598 | 8.909 | 8.975 |
| 5648 | MASP1 | 1.215 | 0.003 | 0.015 | up-reg. | 8.479 | 8.403 | 8.547 | 7.140 | 7.460 | 7.186 |
| 5675 | PSG6 | 1.380 | 0.027 | 0.009 | up-reg. | 12.362 | 11.973 | 12.653 | 10.364 | 11.422 | 11.062 |
| 5725 | PTBP1 | 0.751 | 0.048 | 0.081 | up-reg. | 8.862 | 8.763 | 9.364 | 8.137 | 8.594 | 8.005 |
| 5753 | PTK6 | 1.557 | 0.004 | 0.006 | up-reg. | 9.241 | 8.853 | 9.280 | 7.529 | 7.687 | 7.489 |
| 5778 | PTPN7 | 1.102 | 0.009 | 0.022 | up-reg. | 9.685 | 9.289 | 9.398 | 8.112 | 8.529 | 8.424 |
| 5865 | RAB3B | 0.934 | 0.004 | 0.040 | up-reg. | 10.394 | 10.223 | 10.186 | 9.457 | 9.209 | 9.337 |
| 5880 | RAC2 | 1.152 | 0.003 | 0.018 | up-reg. | 8.509 | 8.536 | 8.492 | 7.358 | 7.217 | 7.507 |
| 5893 | RAD52 | 1.339 | 0.042 | 0.010 | up-reg. | 5.831 | 5.244 | 4.965 | 3.631 | 3.679 | 4.712 |
| 5954 | RCN1 | 0.891 | 0.044 | 0.047 | up-reg. | 5.638 | 5.196 | 5.724 | 4.479 | 5.109 | 4.298 |
| 5971 | RELB | 2.274 | 0.001 | 0.001 | up-reg. | 12.022 | 12.142 | 11.955 | 9.895 | 9.647 | 9.756 |
| 5984 | RFC4 | 0.931 | 0.038 | 0.040 | up-reg. | 11.796 | 11.263 | 11.535 | 10.646 | 10.999 | 10.155 |
| 5993 | RFX5 | 1.146 | 0.007 | 0.018 | up-reg. | 6.839 | 7.040 | 7.013 | 5.886 | 6.018 | 5.549 |
| 6023 | RMRP | 1.351 | 0.014 | 0.010 | up-reg. | 7.455 | 7.068 | 7.210 | 5.737 | 5.583 | 6.358 |
| 6102 | RP2 | 0.943 | 0.025 | 0.038 | up-reg. | 5.904 | 5.337 | 5.910 | 4.482 | 4.905 | 4.936 |
| 6133 | RPL9 | 0.791 | 0.043 | 0.069 | up-reg. | 7.935 | 8.082 | 8.111 | 7.537 | 6.750 | 7.469 |
| 6236 | RRAD | 4.858 | 0.001 | 0.000 | up-reg. | 11.342 | 11.447 | 10.992 | 6.286 | 6.113 | 6.809 |
| 6238 | RRBP1 | 0.719 | 0.014 | 0.091 | up-reg. | 5.950 | 6.156 | 5.974 | 5.542 | 5.236 | 5.146 |
| 6283 | S100A12 | 2.122 | 0.001 | 0.002 | up-reg. | 6.339 | 6.000 | 6.018 | 4.008 | 4.072 | 3.913 |
| 6288 | SAA1 | 2.201 | 0.014 | 0.001 | up-reg. | 6.640 | 5.709 | 6.482 | 3.540 | 4.092 | 4.595 |
| 6295 | SAG | 1.152 | 0.023 | 0.018 | up-reg. | 6.560 | 6.126 | 6.106 | 5.331 | 4.621 | 5.384 |
| 6299 | SALL1 | 0.834 | 0.027 | 0.058 | up-reg. | 6.770 | 6.213 | 6.431 | 5.386 | 5.619 | 5.907 |
| 6327 | SCN2B | 1.109 | 0.028 | 0.021 | up-reg. | 13.030 | 12.909 | 13.290 | 11.461 | 12.418 | 12.022 |
| 6352 | CCL5 | 8.046 | 0.001 | 0.000 | up-reg. | 11.721 | 11.678 | 11.590 | 3.368 | 4.439 | 3.046 |
| 6356 | CCL11 | 8.149 | 0.000 | 0.000 | up-reg. | 11.087 | 11.212 | 10.817 | 3.121 | 2.521 | 3.027 |
| 6415 | SELENOW | 0.766 | 0.004 | 0.076 | up-reg. | 13.505 | 13.640 | 13.412 | 12.792 | 12.680 | 12.787 |
| 6447 | SCG5 | 0.931 | 0.010 | 0.040 | up-reg. | 8.987 | 8.944 | 8.561 | 7.843 | 7.828 | 8.029 |
| 6473 | SHOX | 1.278 | 0.019 | 0.012 | up-reg. | 4.846 | 5.004 | 4.575 | 3.561 | 3.064 | 3.965 |
| 6545 | SLC7A4 | 3.944 | 0.003 | 0.000 | up-reg. | 8.340 | 8.475 | 8.176 | 4.853 | 4.492 | 3.814 |
| 6567 | SLC16A2 | 0.979 | 0.035 | 0.033 | up-reg. | 5.610 | 4.922 | 4.836 | 3.933 | 4.045 | 4.452 |
| 6590 | SLPI | 0.866 | 0.042 | 0.052 | up-reg. | 5.522 | 5.433 | 5.065 | 4.100 | 4.396 | 4.926 |
| 6616 | SNAP25 | 1.411 | 0.038 | 0.009 | up-reg. | 5.135 | 4.272 | 5.221 | 4.082 | 3.039 | 3.273 |
| 6645 | SNTB2 | 1.240 | 0.006 | 0.014 | up-reg. | 6.375 | 6.062 | 6.257 | 4.969 | 5.223 | 4.782 |
| 6649 | SOD3 | 2.964 | 0.001 | 0.000 | up-reg. | 14.337 | 14.557 | 14.337 | 11.528 | 11.313 | 11.499 |
| 6662 | SOX9 | 1.159 | 0.040 | 0.018 | up-reg. | 13.025 | 12.659 | 11.900 | 11.156 | 11.676 | 11.277 |
| 6697 | SPR | 0.989 | 0.003 | 0.032 | up-reg. | 12.057 | 12.217 | 12.106 | 11.231 | 11.022 | 11.158 |
| 6706 | SPRR2G | 2.040 | 0.028 | 0.002 | up-reg. | 10.349 | 9.911 | 10.195 | 7.037 | 8.675 | 8.624 |
| 6710 | SPTB | 0.999 | 0.034 | 0.031 | up-reg. | 4.365 | 4.674 | 4.720 | 3.598 | 3.109 | 4.057 |
| 6722 | SRF | 0.876 | 0.045 | 0.050 | up-reg. | 5.757 | 5.487 | 5.481 | 4.224 | 4.682 | 5.192 |
| 6727 | SRP14 | 0.743 | 0.006 | 0.083 | up-reg. | 10.257 | 10.334 | 10.092 | 9.577 | 9.388 | 9.487 |
| 6737 | TRIM21 | 1.271 | 0.029 | 0.013 | up-reg. | 7.258 | 6.572 | 7.154 | 5.166 | 5.952 | 6.052 |
| 6746 | SSR2 | 1.516 | 0.017 | 0.007 | up-reg. | 7.081 | 6.751 | 7.094 | 5.264 | 5.070 | 6.043 |
| 6750 | SST | 2.429 | 0.006 | 0.001 | up-reg. | 6.161 | 6.333 | 5.764 | 3.318 | 3.490 | 4.163 |
| 6772 | STAT1 | 1.837 | 0.000 | 0.003 | up-reg. | 9.475 | 9.359 | 9.393 | 7.648 | 7.542 | 7.526 |
| 6795 | AURKC | 0.971 | 0.045 | 0.034 | up-reg. | 8.716 | 8.631 | 9.033 | 7.224 | 8.070 | 8.174 |
| 6850 | SYK | 0.708 | 0.021 | 0.095 | up-reg. | 5.411 | 5.024 | 5.024 | 4.312 | 4.366 | 4.656 |
| 6856 | SYPL1 | 1.187 | 0.012 | 0.016 | up-reg. | 13.950 | 13.729 | 13.987 | 12.525 | 12.493 | 13.087 |
| 6916 | TBXAS1 | 0.710 | 0.026 | 0.095 | up-reg. | 5.046 | 4.478 | 4.900 | 3.989 | 4.231 | 4.073 |
| 6990 | DYNLT3 | 0.912 | 0.002 | 0.043 | up-reg. | 8.418 | 8.427 | 8.392 | 7.575 | 7.371 | 7.553 |
| 7006 | TEC | 0.882 | 0.001 | 0.049 | up-reg. | 6.763 | 6.709 | 6.784 | 5.827 | 5.944 | 5.839 |
| 7012 | 0 | 1.141 | 0.006 | 0.019 | up-reg. | 10.836 | 10.488 | 10.442 | 9.356 | 9.515 | 9.472 |
| 7038 | TG | 3.299 | 0.008 | 0.000 | up-reg. | 7.370 | 7.213 | 7.108 | 4.187 | 3.041 | 4.566 |
| 7052 | TGM2 | 1.323 | 0.014 | 0.011 | up-reg. | 7.063 | 6.960 | 6.578 | 5.644 | 5.143 | 5.848 |
| 7057 | THBS1 | 0.820 | 0.003 | 0.062 | up-reg. | 11.232 | 11.276 | 11.108 | 10.488 | 10.326 | 10.342 |
| 7135 | TNNI1 | 1.907 | 0.009 | 0.003 | up-reg. | 5.937 | 5.222 | 5.216 | 3.430 | 3.319 | 3.904 |
| 7166 | TPH1 | 1.012 | 0.043 | 0.029 | up-reg. | 5.434 | 5.194 | 5.615 | 4.157 | 4.037 | 5.014 |
| 7170 | TPM3 | 1.059 | 0.028 | 0.025 | up-reg. | 5.996 | 5.904 | 5.361 | 4.292 | 4.801 | 4.992 |
| 7226 | TRPM2 | 0.798 | 0.015 | 0.067 | up-reg. | 5.028 | 5.092 | 4.615 | 4.163 | 4.018 | 4.159 |
| 7249 | TSC2 | 0.798 | 0.008 | 0.067 | up-reg. | 6.474 | 6.573 | 6.552 | 5.510 | 5.819 | 5.875 |
| 7259 | TSPYL1 | 0.992 | 0.018 | 0.032 | up-reg. | 4.647 | 4.512 | 4.052 | 3.227 | 3.621 | 3.387 |
| 7262 | PHLDA2 | 0.787 | 0.003 | 0.070 | up-reg. | 12.084 | 12.203 | 12.142 | 11.385 | 11.263 | 11.419 |
| 7273 | TTN | 1.119 | 0.008 | 0.020 | up-reg. | 5.703 | 5.441 | 5.754 | 4.295 | 4.539 | 4.706 |
| 7292 | TNFSF4 | 0.884 | 0.021 | 0.048 | up-reg. | 7.038 | 6.665 | 7.068 | 5.733 | 6.117 | 6.269 |
| 7324 | UBE2E1 | 0.905 | 0.021 | 0.045 | up-reg. | 6.557 | 6.381 | 6.477 | 5.218 | 5.556 | 5.927 |
| 7374 | UNG | 0.990 | 0.032 | 0.032 | up-reg. | 12.653 | 12.636 | 12.858 | 11.189 | 11.904 | 12.084 |
| 7379 | UPK2 | 1.037 | 0.019 | 0.027 | up-reg. | 7.431 | 7.266 | 7.549 | 6.049 | 6.293 | 6.793 |
| 7425 | VGF | 0.874 | 0.011 | 0.050 | up-reg. | 6.193 | 6.531 | 6.238 | 5.441 | 5.269 | 5.631 |
| 7466 | WFS1 | 1.399 | 0.023 | 0.009 | up-reg. | 6.368 | 5.844 | 5.682 | 4.063 | 4.642 | 4.993 |
| 7474 | WNT5A | 1.780 | 0.016 | 0.003 | up-reg. | 6.714 | 6.004 | 6.445 | 4.458 | 4.171 | 5.193 |
| 7486 | WRN | 0.716 | 0.022 | 0.092 | up-reg. | 5.333 | 5.388 | 5.208 | 4.283 | 4.663 | 4.835 |
| 7514 | XPO1 | 1.895 | 0.009 | 0.003 | up-reg. | 7.764 | 7.682 | 7.837 | 5.608 | 5.551 | 6.440 |
| 7579 | ZSCAN20 | 2.045 | 0.011 | 0.002 | up-reg. | 7.687 | 6.991 | 7.705 | 5.129 | 5.193 | 5.925 |
| 7771 | ZNF112 | 1.146 | 0.004 | 0.018 | up-reg. | 6.903 | 6.778 | 6.960 | 5.738 | 5.561 | 5.904 |
| 7784 | ZP3 | 0.930 | 0.049 | 0.041 | up-reg. | 7.870 | 7.711 | 7.905 | 6.325 | 6.959 | 7.413 |
| 7805 | LAPTM5 | 1.420 | 0.003 | 0.009 | up-reg. | 8.332 | 8.508 | 8.175 | 6.932 | 6.795 | 7.030 |
| 7873 | MANF | 1.359 | 0.047 | 0.010 | up-reg. | 5.080 | 4.950 | 4.300 | 4.172 | 2.831 | 3.248 |
| 7920 | ABHD16A | 0.799 | 0.027 | 0.067 | up-reg. | 8.047 | 8.043 | 7.921 | 6.793 | 7.332 | 7.490 |
| 7975 | MAFK | 1.703 | 0.000 | 0.004 | up-reg. | 11.504 | 11.554 | 11.456 | 9.838 | 9.711 | 9.857 |
| 7991 | TUSC3 | 0.947 | 0.022 | 0.038 | up-reg. | 4.411 | 3.653 | 3.985 | 2.955 | 3.073 | 3.180 |
| 8086 | AAAS | 1.483 | 0.014 | 0.007 | up-reg. | 12.378 | 12.227 | 12.297 | 10.309 | 10.844 | 11.299 |
| 8292 | COLQ | 2.926 | 0.025 | 0.000 | up-reg. | 5.976 | 6.056 | 7.171 | 2.417 | 3.360 | 4.649 |
| 8322 | FZD4 | 1.825 | 0.008 | 0.003 | up-reg. | 6.995 | 7.108 | 6.814 | 5.237 | 4.668 | 5.537 |
| 8323 | FZD6 | 0.911 | 0.014 | 0.043 | up-reg. | 6.231 | 5.694 | 5.752 | 4.932 | 5.010 | 5.000 |
| 8351 | H3C4 | 3.401 | 0.001 | 0.000 | up-reg. | 7.502 | 7.636 | 7.418 | 3.942 | 4.016 | 4.396 |
| 8399 | PLA2G10 | 1.344 | 0.016 | 0.010 | up-reg. | 9.181 | 8.990 | 9.266 | 7.502 | 7.574 | 8.330 |
| 8409 | UXT | 0.973 | 0.007 | 0.034 | up-reg. | 13.122 | 12.846 | 12.725 | 11.863 | 12.048 | 11.863 |
| 8416 | ANXA9 | 1.536 | 0.010 | 0.006 | up-reg. | 9.997 | 9.746 | 10.048 | 7.946 | 8.618 | 8.619 |
| 8419 | BFSP2 | 1.117 | 0.026 | 0.021 | up-reg. | 9.209 | 8.820 | 9.313 | 7.592 | 8.452 | 7.946 |
| 8526 | DGKE | 1.254 | 0.023 | 0.013 | up-reg. | 6.464 | 6.155 | 6.302 | 4.488 | 5.202 | 5.469 |
| 8535 | CBX4 | 0.921 | 0.004 | 0.042 | up-reg. | 8.120 | 8.227 | 7.969 | 7.147 | 7.296 | 7.110 |
| 8635 | RNASET2 | 0.880 | 0.015 | 0.049 | up-reg. | 10.101 | 10.207 | 9.879 | 9.379 | 8.893 | 9.276 |
| 8651 | SOCS1 | 1.049 | 0.005 | 0.026 | up-reg. | 7.340 | 7.231 | 7.092 | 5.989 | 6.224 | 6.304 |
| 8711 | TNK1 | 1.615 | 0.015 | 0.005 | up-reg. | 12.890 | 12.601 | 13.052 | 10.870 | 11.014 | 11.816 |
| 8722 | CTSF | 0.833 | 0.006 | 0.059 | up-reg. | 12.088 | 12.106 | 12.069 | 11.434 | 11.100 | 11.231 |
| 8743 | TNFSF10 | 0.982 | 0.037 | 0.033 | up-reg. | 10.512 | 10.265 | 10.791 | 9.111 | 10.005 | 9.505 |
| 8747 | ADAM21 | 1.924 | 0.008 | 0.003 | up-reg. | 8.103 | 7.854 | 8.159 | 5.652 | 6.178 | 6.515 |
| 8828 | NRP2 | 0.835 | 0.033 | 0.058 | up-reg. | 5.118 | 4.967 | 4.945 | 3.863 | 4.024 | 4.637 |
| 8837 | CFLAR | 1.507 | 0.036 | 0.007 | up-reg. | 5.370 | 5.001 | 4.657 | 3.334 | 2.895 | 4.278 |
| 8840 | CCN4 | 0.790 | 0.009 | 0.070 | up-reg. | 6.088 | 6.282 | 6.205 | 5.577 | 5.207 | 5.423 |
| 8851 | CDK5R1 | 2.077 | 0.025 | 0.002 | up-reg. | 9.266 | 9.036 | 9.326 | 6.096 | 7.446 | 7.855 |
| 8870 | IER3 | 5.532 | 0.000 | 0.000 | up-reg. | 16.323 | 16.520 | 16.520 | 11.025 | 10.783 | 10.959 |
| 8875 | VNN2 | 0.734 | 0.036 | 0.086 | up-reg. | 9.758 | 9.541 | 10.084 | 8.799 | 9.342 | 9.041 |
| 8935 | SKAP2 | 1.178 | 0.039 | 0.017 | up-reg. | 5.033 | 5.053 | 4.560 | 3.044 | 3.930 | 4.136 |
| 8992 | ATP6V0E1 | 0.919 | 0.000 | 0.042 | up-reg. | 14.504 | 14.469 | 14.469 | 13.593 | 13.518 | 13.575 |
| 8997 | KALRN | 1.542 | 0.005 | 0.006 | up-reg. | 6.016 | 5.951 | 5.952 | 4.563 | 4.114 | 4.615 |
| 9023 | CH25H | 1.805 | 0.006 | 0.003 | up-reg. | 7.327 | 7.321 | 7.072 | 5.045 | 5.636 | 5.624 |
| 9051 | PSTPIP1 | 1.165 | 0.024 | 0.017 | up-reg. | 8.882 | 8.422 | 8.994 | 7.272 | 8.060 | 7.472 |
| 9055 | PRC1 | 1.035 | 0.044 | 0.027 | up-reg. | 5.382 | 4.909 | 5.334 | 4.706 | 3.639 | 4.174 |
| 9056 | SLC7A7 | 1.129 | 0.038 | 0.020 | up-reg. | 8.452 | 8.340 | 8.671 | 6.690 | 7.613 | 7.773 |
| 9077 | DIRAS3 | 1.381 | 0.044 | 0.009 | up-reg. | 6.581 | 6.734 | 6.108 | 5.075 | 4.380 | 5.825 |
| 9086 | EIF1AY | 1.322 | 0.016 | 0.011 | up-reg. | 6.315 | 6.428 | 5.986 | 4.915 | 4.505 | 5.342 |
| 9120 | SLC16A6 | 1.089 | 0.017 | 0.023 | up-reg. | 4.945 | 4.479 | 4.537 | 3.406 | 3.371 | 3.917 |
| 9121 | SLC16A5 | 0.907 | 0.010 | 0.044 | up-reg. | 8.051 | 7.649 | 7.690 | 7.005 | 6.890 | 6.774 |
| 9144 | SYNGR2 | 1.618 | 0.014 | 0.005 | up-reg. | 6.344 | 6.295 | 5.951 | 4.298 | 4.283 | 5.153 |
| 9166 | EBAG9 | 0.811 | 0.003 | 0.064 | up-reg. | 5.838 | 5.755 | 5.724 | 5.029 | 4.845 | 5.010 |
| 9179 | AP4M1 | 1.855 | 0.019 | 0.003 | up-reg. | 8.077 | 7.783 | 8.254 | 5.453 | 6.305 | 6.790 |
| 9241 | NOG | 1.752 | 0.010 | 0.004 | up-reg. | 6.338 | 6.481 | 5.669 | 4.209 | 4.616 | 4.408 |
| 9296 | ATP6V1F | 0.801 | 0.015 | 0.067 | up-reg. | 13.421 | 13.650 | 13.142 | 12.697 | 12.476 | 12.636 |
| 9344 | TAOK2 | 1.076 | 0.038 | 0.024 | up-reg. | 7.059 | 6.849 | 7.279 | 5.419 | 6.057 | 6.484 |
| 9355 | LHX2 | 2.189 | 0.007 | 0.002 | up-reg. | 9.513 | 9.212 | 9.681 | 6.783 | 7.426 | 7.628 |
| 9365 | KL | 1.552 | 0.017 | 0.006 | up-reg. | 9.185 | 8.831 | 9.300 | 6.995 | 7.997 | 7.667 |
| 9372 | ZFYVE9 | 1.085 | 0.006 | 0.023 | up-reg. | 9.076 | 8.658 | 8.824 | 7.698 | 7.809 | 7.795 |
| 9391 | CIAO1 | 1.240 | 0.043 | 0.014 | up-reg. | 5.464 | 4.470 | 4.954 | 4.212 | 3.226 | 3.731 |
| 9402 | GRAP2 | 1.121 | 0.012 | 0.020 | up-reg. | 7.769 | 7.756 | 7.759 | 6.279 | 6.685 | 6.956 |
| 9419 | CRIPT | 0.982 | 0.040 | 0.033 | up-reg. | 5.396 | 4.869 | 4.721 | 3.929 | 3.657 | 4.454 |
| 9421 | HAND1 | 0.945 | 0.025 | 0.038 | up-reg. | 12.621 | 12.388 | 12.683 | 11.184 | 11.716 | 11.955 |
| 9435 | CHST2 | 0.948 | 0.005 | 0.038 | up-reg. | 11.096 | 11.232 | 10.983 | 10.281 | 10.009 | 10.175 |
| 9447 | AIM2 | 1.394 | 0.029 | 0.009 | up-reg. | 5.578 | 5.147 | 6.375 | 4.248 | 4.575 | 4.094 |
| 9450 | LY86 | 1.281 | 0.009 | 0.012 | up-reg. | 7.247 | 6.805 | 6.741 | 5.463 | 5.878 | 5.609 |
| 9464 | HAND2 | 1.276 | 0.032 | 0.012 | up-reg. | 5.992 | 5.864 | 5.542 | 4.690 | 3.864 | 5.015 |
| 9514 | GAL3ST1 | 1.704 | 0.021 | 0.004 | up-reg. | 6.274 | 5.562 | 6.173 | 3.720 | 4.304 | 4.872 |
| 9518 | GDF15 | 1.407 | 0.004 | 0.009 | up-reg. | 10.468 | 10.511 | 10.279 | 9.228 | 8.889 | 8.919 |
| 9527 | GOSR1 | 1.629 | 0.026 | 0.005 | up-reg. | 9.059 | 8.874 | 9.105 | 6.632 | 7.419 | 8.098 |
| 9536 | PTGES | 0.854 | 0.012 | 0.054 | up-reg. | 13.284 | 13.711 | 13.235 | 12.532 | 12.568 | 12.568 |
| 9541 | CIR1 | 1.187 | 0.043 | 0.016 | up-reg. | 4.452 | 4.469 | 4.174 | 2.612 | 3.035 | 3.887 |
| 9546 | APBA3 | 1.159 | 0.022 | 0.018 | up-reg. | 5.847 | 5.605 | 5.059 | 4.297 | 4.113 | 4.622 |
| 9550 | ATP6V1G1 | 1.878 | 0.014 | 0.003 | up-reg. | 6.287 | 5.870 | 6.390 | 4.547 | 3.651 | 4.714 |
| 9553 | MRPL33 | 1.623 | 0.001 | 0.005 | up-reg. | 9.130 | 9.206 | 9.153 | 7.469 | 7.645 | 7.505 |
| 9589 | WTAP | 1.165 | 0.022 | 0.017 | up-reg. | 8.056 | 8.130 | 8.725 | 6.880 | 7.492 | 7.042 |
| 9603 | NFE2L3 | 2.440 | 0.003 | 0.001 | up-reg. | 5.540 | 5.173 | 4.825 | 2.848 | 2.682 | 2.688 |
| 9620 | CELSR1 | 0.909 | 0.033 | 0.044 | up-reg. | 4.399 | 3.805 | 4.337 | 3.035 | 3.144 | 3.636 |
| 9622 | KLK4 | 1.602 | 0.047 | 0.005 | up-reg. | 6.044 | 5.687 | 5.859 | 4.064 | 3.445 | 5.274 |
| 9636 | ISG15 | 2.897 | 0.000 | 0.001 | up-reg. | 15.745 | 15.859 | 15.859 | 13.025 | 12.808 | 12.938 |
| 9639 | ARHGEF10 | 1.360 | 0.005 | 0.010 | up-reg. | 5.914 | 5.833 | 5.726 | 4.331 | 4.321 | 4.740 |
| 9653 | HS2ST1 | 0.888 | 0.024 | 0.048 | up-reg. | 6.949 | 7.331 | 6.700 | 6.156 | 5.862 | 6.299 |
| 9705 | ST18 | 1.360 | 0.025 | 0.010 | up-reg. | 10.777 | 10.445 | 10.859 | 8.733 | 9.869 | 9.398 |
| 9751 | SNPH | 1.043 | 0.014 | 0.027 | up-reg. | 7.224 | 7.407 | 7.175 | 6.096 | 5.992 | 6.587 |
| 9765 | ZFYVE16 | 1.126 | 0.033 | 0.020 | up-reg. | 9.784 | 9.642 | 10.234 | 8.215 | 9.065 | 9.001 |
| 9791 | PTDSS1 | 1.371 | 0.014 | 0.010 | up-reg. | 6.984 | 6.581 | 6.927 | 5.079 | 5.421 | 5.878 |
| 9792 | SERTAD2 | 0.701 | 0.015 | 0.098 | up-reg. | 9.991 | 10.268 | 9.844 | 9.468 | 9.280 | 9.252 |
| 9833 | MELK | 1.465 | 0.014 | 0.007 | up-reg. | 10.115 | 9.583 | 10.024 | 8.009 | 8.762 | 8.558 |
| 9841 | ZBTB24 | 1.080 | 0.021 | 0.023 | up-reg. | 6.718 | 6.348 | 6.493 | 5.546 | 4.995 | 5.778 |
| 9848 | MFAP3L | 1.651 | 0.012 | 0.005 | up-reg. | 8.143 | 8.561 | 8.007 | 6.091 | 6.877 | 6.790 |
| 9854 | C2CD2L | 1.465 | 0.005 | 0.007 | up-reg. | 10.243 | 10.529 | 10.145 | 9.004 | 8.647 | 8.874 |
| 9924 | PAN2 | 1.260 | 0.046 | 0.013 | up-reg. | 8.857 | 8.795 | 9.395 | 7.009 | 8.231 | 8.027 |
| 9927 | MFN2 | 1.013 | 0.007 | 0.029 | up-reg. | 5.260 | 4.835 | 4.892 | 4.029 | 3.970 | 3.949 |
| 9935 | MAFB | 2.743 | 0.003 | 0.001 | up-reg. | 6.289 | 6.384 | 6.052 | 3.563 | 3.140 | 3.792 |
| 9946 | CRYZL1 | 1.050 | 0.017 | 0.026 | up-reg. | 4.757 | 4.560 | 4.860 | 4.010 | 3.296 | 3.723 |
| 9958 | USP15 | 1.240 | 0.014 | 0.014 | up-reg. | 4.506 | 4.904 | 4.510 | 3.690 | 3.017 | 3.494 |
| 10021 | HCN4 | 0.802 | 0.012 | 0.066 | up-reg. | 6.351 | 5.970 | 6.371 | 5.542 | 5.393 | 5.351 |
| 10045 | SH2D3A | 0.845 | 0.018 | 0.056 | up-reg. | 6.971 | 7.145 | 6.695 | 5.840 | 6.178 | 6.260 |
| 10050 | SLC17A4 | 0.927 | 0.021 | 0.041 | up-reg. | 5.380 | 4.794 | 5.274 | 4.019 | 4.449 | 4.199 |
| 10196 | PRMT3 | 0.775 | 0.028 | 0.074 | up-reg. | 13.593 | 13.381 | 13.802 | 12.510 | 13.104 | 12.838 |
| 10205 | MPZL2 | 1.948 | 0.014 | 0.002 | up-reg. | 8.080 | 7.351 | 8.035 | 5.322 | 6.023 | 6.278 |
| 10250 | SRRM1 | 0.856 | 0.041 | 0.054 | up-reg. | 5.789 | 5.608 | 5.577 | 4.429 | 4.658 | 5.320 |
| 10333 | TLR6 | 1.376 | 0.036 | 0.010 | up-reg. | 6.034 | 5.266 | 5.625 | 4.489 | 3.574 | 4.735 |
| 10336 | PCGF3 | 1.124 | 0.010 | 0.020 | up-reg. | 7.926 | 7.767 | 8.227 | 6.605 | 7.040 | 6.902 |
| 10344 | CCL26 | 3.165 | 0.000 | 0.000 | up-reg. | 11.061 | 11.025 | 11.014 | 7.954 | 7.844 | 7.807 |
| 10544 | PROCR | 0.711 | 0.013 | 0.094 | up-reg. | 9.637 | 9.697 | 9.482 | 9.105 | 8.703 | 8.876 |
| 10607 | TBL3 | 0.698 | 0.008 | 0.099 | up-reg. | 5.605 | 5.775 | 5.738 | 4.865 | 4.988 | 5.170 |
| 10617 | STAMBP | 1.178 | 0.016 | 0.017 | up-reg. | 9.720 | 9.097 | 9.613 | 8.006 | 8.382 | 8.508 |
| 10647 | SCGB1D2 | 1.053 | 0.038 | 0.026 | up-reg. | 5.436 | 4.941 | 5.118 | 4.298 | 3.538 | 4.499 |
| 10651 | MTX2 | 0.832 | 0.024 | 0.059 | up-reg. | 5.863 | 5.942 | 6.037 | 4.813 | 5.029 | 5.502 |
| 10658 | CELF1 | 1.083 | 0.007 | 0.023 | up-reg. | 10.872 | 10.747 | 11.189 | 9.806 | 9.974 | 9.777 |
| 10661 | KLF1 | 1.457 | 0.008 | 0.008 | up-reg. | 10.895 | 10.473 | 10.902 | 9.015 | 9.482 | 9.403 |
| 10678 | B3GNT2 | 1.302 | 0.004 | 0.011 | up-reg. | 8.764 | 8.857 | 8.511 | 7.560 | 7.260 | 7.406 |
| 10683 | DLL3 | 0.779 | 0.013 | 0.072 | up-reg. | 5.678 | 5.202 | 5.350 | 4.661 | 4.581 | 4.649 |
| 10749 | KIF1C | 1.268 | 0.030 | 0.013 | up-reg. | 7.009 | 6.942 | 6.647 | 5.759 | 4.954 | 6.081 |
| 10775 | POP4 | 1.124 | 0.023 | 0.020 | up-reg. | 12.297 | 11.504 | 11.918 | 10.568 | 11.081 | 10.698 |
| 10803 | CCR9 | 0.827 | 0.007 | 0.060 | up-reg. | 5.860 | 5.677 | 5.811 | 4.769 | 4.993 | 5.106 |
| 10826 | FAXDC2 | 1.628 | 0.018 | 0.005 | up-reg. | 4.882 | 3.895 | 4.924 | 2.924 | 2.757 | 3.136 |
| 10856 | RUVBL2 | 1.150 | 0.007 | 0.018 | up-reg. | 5.832 | 5.375 | 5.813 | 4.580 | 4.536 | 4.455 |
| 10870 | HCST | 0.931 | 0.013 | 0.040 | up-reg. | 7.508 | 7.346 | 7.238 | 6.654 | 6.135 | 6.510 |
| 10886 | NPFFR2 | 1.481 | 0.031 | 0.007 | up-reg. | 5.314 | 4.730 | 4.155 | 2.749 | 3.557 | 3.449 |
| 10893 | MMP24 | 1.084 | 0.006 | 0.023 | up-reg. | 4.785 | 5.166 | 4.865 | 3.927 | 3.725 | 3.912 |
| 10903 | MTMR11 | 0.867 | 0.016 | 0.052 | up-reg. | 6.698 | 6.413 | 6.791 | 5.521 | 5.794 | 5.987 |
| 10915 | TCERG1 | 1.464 | 0.010 | 0.007 | up-reg. | 7.013 | 6.455 | 6.994 | 5.186 | 5.217 | 5.667 |
| 10919 | EHMT2 | 1.341 | 0.035 | 0.010 | up-reg. | 11.565 | 11.404 | 11.846 | 9.515 | 10.711 | 10.564 |
| 10975 | UQCR11 | 1.507 | 0.017 | 0.007 | up-reg. | 7.646 | 7.863 | 7.481 | 6.232 | 5.597 | 6.640 |
| 11017 | SNRNP27 | 0.729 | 0.029 | 0.088 | up-reg. | 4.905 | 4.720 | 4.396 | 3.996 | 3.694 | 4.146 |
| 11035 | RIPK3 | 0.735 | 0.007 | 0.085 | up-reg. | 7.406 | 7.471 | 7.536 | 6.850 | 6.564 | 6.793 |
| 11085 | ADAM30 | 1.088 | 0.011 | 0.023 | up-reg. | 6.068 | 5.719 | 6.095 | 5.049 | 4.973 | 4.597 |
| 11086 | ADAM29 | 1.040 | 0.007 | 0.027 | up-reg. | 6.141 | 6.033 | 6.465 | 5.127 | 5.255 | 5.136 |
| 11153 | FICD | 2.232 | 0.005 | 0.001 | up-reg. | 5.607 | 5.112 | 5.291 | 3.076 | 3.438 | 2.801 |
| 11166 | SOX21 | 1.529 | 0.016 | 0.006 | up-reg. | 10.900 | 10.326 | 11.032 | 8.859 | 9.640 | 9.171 |
| 11170 | FAM107A | 0.906 | 0.009 | 0.044 | up-reg. | 6.792 | 6.537 | 6.632 | 5.697 | 5.584 | 5.963 |
| 11219 | TREX2 | 1.019 | 0.031 | 0.029 | up-reg. | 7.986 | 7.810 | 8.049 | 6.512 | 7.455 | 6.821 |
| 11221 | DUSP10 | 1.278 | 0.011 | 0.012 | up-reg. | 10.404 | 10.164 | 10.596 | 8.760 | 9.308 | 9.263 |
| 11227 | GALNT5 | 0.754 | 0.005 | 0.080 | up-reg. | 7.276 | 7.374 | 7.219 | 6.407 | 6.558 | 6.641 |
| 11262 | SP140 | 1.621 | 0.031 | 0.005 | up-reg. | 5.162 | 5.128 | 5.055 | 2.630 | 4.190 | 3.662 |
| 11333 | PDAP1 | 0.746 | 0.019 | 0.082 | up-reg. | 5.112 | 4.867 | 5.233 | 4.074 | 4.498 | 4.400 |
| 11334 | TUSC2 | 1.170 | 0.000 | 0.017 | up-reg. | 10.726 | 10.807 | 10.711 | 9.554 | 9.581 | 9.598 |
| 22824 | HSPA4L | 0.920 | 0.048 | 0.042 | up-reg. | 4.794 | 4.549 | 3.872 | 3.722 | 3.192 | 3.540 |
| 22826 | DNAJC8 | 1.576 | 0.006 | 0.006 | up-reg. | 5.107 | 5.300 | 4.857 | 3.228 | 3.759 | 3.549 |
| 22838 | RNF44 | 1.052 | 0.007 | 0.026 | up-reg. | 7.586 | 7.773 | 7.463 | 6.748 | 6.411 | 6.506 |
| 22901 | ARSG | 1.503 | 0.008 | 0.007 | up-reg. | 10.438 | 9.980 | 10.378 | 8.465 | 8.876 | 8.947 |
| 22943 | DKK1 | 1.469 | 0.013 | 0.007 | up-reg. | 10.844 | 10.141 | 10.578 | 8.884 | 9.408 | 8.864 |
| 23014 | FBXO21 | 1.020 | 0.033 | 0.029 | up-reg. | 6.207 | 6.412 | 5.629 | 5.124 | 4.725 | 5.339 |
| 23019 | CNOT1 | 0.868 | 0.021 | 0.051 | up-reg. | 7.022 | 6.408 | 6.696 | 5.781 | 5.698 | 6.045 |
| 23023 | TMCC1 | 0.930 | 0.018 | 0.040 | up-reg. | 13.619 | 12.936 | 13.400 | 12.378 | 12.399 | 12.388 |
| 23132 | RAD54L2 | 1.146 | 0.016 | 0.018 | up-reg. | 10.164 | 9.993 | 10.316 | 8.608 | 9.084 | 9.343 |
| 23142 | DCUN1D4 | 1.004 | 0.004 | 0.030 | up-reg. | 4.365 | 4.267 | 4.478 | 3.215 | 3.401 | 3.483 |
| 23154 | NCDN | 0.707 | 0.026 | 0.096 | up-reg. | 5.704 | 5.645 | 5.587 | 5.140 | 4.575 | 5.100 |
| 23184 | MESD | 0.741 | 0.009 | 0.084 | up-reg. | 7.529 | 7.831 | 7.501 | 6.787 | 6.943 | 6.906 |
| 23209 | MLC1 | 1.139 | 0.013 | 0.019 | up-reg. | 6.462 | 6.064 | 6.188 | 4.749 | 5.258 | 5.290 |
| 23214 | XPO6 | 0.769 | 0.031 | 0.075 | up-reg. | 5.435 | 4.900 | 4.906 | 4.269 | 4.549 | 4.114 |
| 23225 | NUP210 | 1.166 | 0.013 | 0.017 | up-reg. | 8.544 | 8.202 | 8.398 | 6.832 | 7.471 | 7.344 |
| 23228 | PLCL2 | 1.110 | 0.015 | 0.021 | up-reg. | 8.742 | 8.289 | 8.680 | 7.120 | 7.646 | 7.615 |
| 23258 | DENND5A | 1.285 | 0.013 | 0.012 | up-reg. | 6.266 | 6.154 | 6.708 | 5.205 | 4.761 | 5.307 |
| 23305 | ACSL6 | 1.573 | 0.009 | 0.006 | up-reg. | 8.998 | 8.479 | 9.168 | 7.127 | 7.502 | 7.298 |
| 23327 | NEDD4L | 1.935 | 0.006 | 0.002 | up-reg. | 8.631 | 8.689 | 8.408 | 6.253 | 6.984 | 6.687 |
| 23396 | PIP5K1C | 0.910 | 0.019 | 0.044 | up-reg. | 11.142 | 11.108 | 11.118 | 9.834 | 10.519 | 10.286 |
| 23484 | LEPROTL1 | 0.968 | 0.005 | 0.035 | up-reg. | 8.380 | 8.265 | 8.112 | 7.298 | 7.144 | 7.411 |
| 23500 | DAAM2 | 0.904 | 0.036 | 0.045 | up-reg. | 6.191 | 6.427 | 6.808 | 5.254 | 5.503 | 5.957 |
| 23507 | LRRC8B | 1.678 | 0.027 | 0.004 | up-reg. | 5.765 | 5.213 | 5.244 | 2.914 | 4.063 | 4.213 |
| 23516 | SLC39A14 | 0.820 | 0.040 | 0.062 | up-reg. | 5.930 | 5.352 | 5.703 | 4.990 | 4.448 | 5.087 |
| 23520 | ANP32C | 0.726 | 0.035 | 0.089 | up-reg. | 4.895 | 5.273 | 4.787 | 4.437 | 3.945 | 4.395 |
| 23546 | SYNGR4 | 1.420 | 0.013 | 0.009 | up-reg. | 12.731 | 12.498 | 12.796 | 10.797 | 11.628 | 11.339 |
| 23547 | LILRA4 | 1.602 | 0.019 | 0.005 | up-reg. | 8.073 | 7.479 | 8.065 | 5.684 | 6.471 | 6.658 |
| 23560 | GTPBP4 | 1.142 | 0.042 | 0.019 | up-reg. | 12.333 | 12.060 | 12.584 | 10.648 | 11.805 | 11.096 |
| 23568 | ARL2BP | 0.852 | 0.009 | 0.055 | up-reg. | 8.714 | 8.458 | 8.764 | 7.658 | 7.947 | 7.775 |
| 23593 | HEBP2 | 0.887 | 0.011 | 0.048 | up-reg. | 9.984 | 9.865 | 10.079 | 8.812 | 9.195 | 9.261 |
| 23635 | SSBP2 | 0.980 | 0.021 | 0.033 | up-reg. | 5.324 | 5.069 | 4.576 | 4.151 | 3.992 | 3.885 |
| 23645 | PPP1R15A | 0.987 | 0.047 | 0.032 | up-reg. | 6.104 | 5.956 | 5.955 | 4.493 | 4.931 | 5.630 |
| 23710 | GABARAPL1 | 1.571 | 0.003 | 0.006 | up-reg. | 12.825 | 13.135 | 12.703 | 11.366 | 11.358 | 11.225 |
| 23766 | GABARAPL3 | 1.594 | 0.006 | 0.005 | up-reg. | 9.934 | 9.379 | 9.784 | 7.893 | 8.267 | 8.154 |
| 24147 | FJX1 | 1.043 | 0.047 | 0.027 | up-reg. | 11.495 | 10.858 | 11.757 | 9.957 | 10.753 | 10.271 |
| 25769 | SLC24A2 | 1.295 | 0.011 | 0.012 | up-reg. | 11.366 | 11.255 | 11.504 | 9.671 | 10.330 | 10.239 |
| 25806 | VAX2 | 1.256 | 0.001 | 0.013 | up-reg. | 7.954 | 8.062 | 7.858 | 6.706 | 6.721 | 6.679 |
| 25825 | BACE2 | 0.796 | 0.018 | 0.068 | up-reg. | 4.792 | 4.898 | 5.062 | 4.246 | 3.814 | 4.302 |
| 25831 | HECTD1 | 0.899 | 0.027 | 0.046 | up-reg. | 5.809 | 5.625 | 5.837 | 4.575 | 4.687 | 5.310 |
| 25850 | ZNF345 | 1.553 | 0.007 | 0.006 | up-reg. | 11.035 | 10.682 | 11.096 | 9.076 | 9.627 | 9.452 |
| 25907 | TMEM158 | 2.608 | 0.000 | 0.001 | up-reg. | 14.941 | 15.148 | 14.969 | 12.432 | 12.382 | 12.418 |
| 25946 | ZNF385A | 0.889 | 0.006 | 0.048 | up-reg. | 6.732 | 6.375 | 6.546 | 5.663 | 5.730 | 5.594 |
| 25992 | SNED1 | 0.787 | 0.042 | 0.070 | up-reg. | 8.684 | 8.106 | 8.900 | 7.636 | 7.962 | 7.731 |
| 26019 | UPF2 | 1.829 | 0.011 | 0.003 | up-reg. | 5.928 | 5.198 | 5.417 | 3.571 | 4.113 | 3.371 |
| 26053 | AUTS2 | 1.022 | 0.044 | 0.028 | up-reg. | 5.073 | 4.354 | 4.278 | 3.487 | 3.196 | 3.956 |
| 26084 | ARHGEF26 | 1.485 | 0.041 | 0.007 | up-reg. | 10.788 | 10.451 | 11.581 | 8.893 | 10.056 | 9.418 |
| 26099 | SZRD1 | 0.865 | 0.043 | 0.052 | up-reg. | 11.339 | 11.035 | 11.339 | 9.887 | 10.788 | 10.442 |
| 26207 | PITPNC1 | 0.713 | 0.020 | 0.093 | up-reg. | 6.572 | 6.201 | 6.684 | 5.640 | 5.777 | 5.901 |
| 26211 | OR2F1 | 0.816 | 0.016 | 0.063 | up-reg. | 5.938 | 5.927 | 6.025 | 4.833 | 5.216 | 5.392 |
| 26263 | FBXO22 | 1.443 | 0.018 | 0.008 | up-reg. | 6.508 | 6.483 | 5.985 | 4.504 | 4.745 | 5.396 |
| 26270 | FBXO6 | 1.311 | 0.031 | 0.011 | up-reg. | 6.649 | 6.627 | 6.671 | 5.695 | 4.594 | 5.727 |
| 26297 | SERGEF | 1.260 | 0.026 | 0.013 | up-reg. | 10.113 | 10.132 | 10.329 | 8.370 | 9.480 | 8.944 |
| 26508 | HEYL | 2.285 | 0.007 | 0.001 | up-reg. | 6.241 | 6.079 | 5.797 | 4.161 | 3.889 | 3.213 |
| 26576 | SRPK3 | 1.162 | 0.026 | 0.017 | up-reg. | 11.184 | 10.971 | 11.081 | 9.363 | 9.997 | 10.388 |
| 26648 | OR7E24 | 1.813 | 0.017 | 0.003 | up-reg. | 6.591 | 5.847 | 6.568 | 4.255 | 4.203 | 5.110 |
| 26762 | HAVCR1 | 0.805 | 0.001 | 0.066 | up-reg. | 10.401 | 10.438 | 10.404 | 9.631 | 9.669 | 9.528 |
| 26994 | RNF11 | 1.183 | 0.035 | 0.016 | up-reg. | 6.904 | 6.742 | 6.434 | 5.252 | 5.126 | 6.152 |
| 27033 | ZBTB32 | 1.080 | 0.021 | 0.023 | up-reg. | 6.480 | 6.371 | 6.570 | 5.066 | 5.239 | 5.874 |
| 27068 | PPA2 | 0.798 | 0.017 | 0.068 | up-reg. | 6.150 | 6.057 | 6.046 | 5.075 | 5.170 | 5.614 |
| 27086 | FOXP1 | 0.884 | 0.047 | 0.048 | up-reg. | 6.775 | 6.660 | 6.213 | 5.451 | 5.386 | 6.159 |
| 27094 | KCNMB3 | 0.798 | 0.046 | 0.068 | up-reg. | 6.230 | 7.084 | 6.549 | 5.626 | 5.935 | 5.909 |
| 27131 | SNX5 | 0.761 | 0.018 | 0.078 | up-reg. | 9.730 | 9.486 | 9.956 | 8.903 | 9.144 | 8.842 |
| 27148 | STK36 | 1.043 | 0.020 | 0.027 | up-reg. | 7.392 | 7.019 | 7.442 | 5.866 | 6.546 | 6.313 |
| 27180 | SIGLEC9 | 1.231 | 0.046 | 0.014 | up-reg. | 10.632 | 9.647 | 10.868 | 8.930 | 9.509 | 9.015 |
| 27183 | VPS4A | 0.813 | 0.008 | 0.064 | up-reg. | 5.960 | 5.800 | 5.679 | 4.846 | 5.104 | 5.050 |
| 27189 | IL17C | 2.209 | 0.035 | 0.001 | up-reg. | 6.170 | 5.848 | 5.732 | 2.441 | 4.099 | 4.581 |
| 27237 | ARHGEF16 | 1.577 | 0.006 | 0.006 | up-reg. | 6.004 | 6.436 | 5.815 | 4.431 | 4.489 | 4.603 |
| 27253 | PCDH17 | 0.769 | 0.021 | 0.075 | up-reg. | 4.371 | 4.003 | 4.336 | 3.303 | 3.738 | 3.364 |
| 27289 | RND1 | 1.078 | 0.027 | 0.023 | up-reg. | 5.733 | 5.307 | 5.413 | 4.182 | 4.117 | 4.918 |
| 27345 | KCNMB4 | 4.224 | 0.001 | 0.000 | up-reg. | 7.818 | 7.741 | 7.615 | 3.679 | 3.134 | 3.689 |
| 27351 | DESI1 | 0.976 | 0.005 | 0.034 | up-reg. | 10.239 | 10.358 | 10.087 | 9.377 | 9.136 | 9.244 |
| 27445 | PCLO | 1.689 | 0.005 | 0.004 | up-reg. | 6.362 | 6.023 | 6.104 | 4.349 | 4.313 | 4.759 |
| 28227 | PPP2R3B | 1.536 | 0.024 | 0.006 | up-reg. | 6.677 | 6.513 | 6.135 | 4.888 | 4.306 | 5.524 |
| 28965 | SLC27A6 | 0.852 | 0.006 | 0.055 | up-reg. | 10.150 | 9.871 | 10.115 | 9.091 | 9.252 | 9.237 |
| 28992 | MACROD1 | 1.204 | 0.016 | 0.015 | up-reg. | 10.281 | 10.289 | 10.388 | 8.651 | 9.495 | 9.201 |
| 29104 | N6AMT1 | 0.979 | 0.028 | 0.033 | up-reg. | 7.827 | 7.789 | 8.215 | 6.591 | 6.922 | 7.380 |
| 29942 | PURG | 1.490 | 0.009 | 0.007 | up-reg. | 9.018 | 8.834 | 9.030 | 7.028 | 7.718 | 7.665 |
| 29943 | PADI1 | 0.957 | 0.046 | 0.036 | up-reg. | 5.141 | 4.637 | 5.095 | 4.056 | 3.494 | 4.454 |
| 30001 | ERO1A | 1.075 | 0.018 | 0.024 | up-reg. | 6.771 | 6.632 | 6.640 | 5.437 | 5.316 | 6.065 |
| 43849 | KLK12 | 0.931 | 0.004 | 0.040 | up-reg. | 8.134 | 7.964 | 8.102 | 7.005 | 7.131 | 7.269 |
| 50486 | G0S2 | 7.118 | 0.000 | 0.000 | up-reg. | 12.590 | 12.508 | 12.580 | 5.124 | 5.625 | 5.575 |
| 50834 | TAS2R1 | 0.727 | 0.048 | 0.088 | up-reg. | 4.982 | 5.002 | 5.051 | 4.084 | 3.989 | 4.781 |
| 50855 | PARD6A | 0.827 | 0.010 | 0.060 | up-reg. | 7.583 | 7.646 | 7.599 | 6.624 | 6.677 | 7.046 |
| 51014 | TMED7 | 0.818 | 0.012 | 0.062 | up-reg. | 7.653 | 7.826 | 7.388 | 6.894 | 6.656 | 6.864 |
| 51026 | GOLT1B | 1.777 | 0.006 | 0.004 | up-reg. | 8.459 | 7.912 | 8.189 | 6.147 | 6.592 | 6.488 |
| 51028 | VPS36 | 1.926 | 0.003 | 0.003 | up-reg. | 9.168 | 8.933 | 9.234 | 6.967 | 7.280 | 7.311 |
| 51062 | ATL1 | 1.150 | 0.026 | 0.018 | up-reg. | 8.973 | 8.362 | 8.862 | 7.141 | 7.953 | 7.651 |
| 51094 | ADIPOR1 | 0.851 | 0.012 | 0.055 | up-reg. | 7.704 | 7.715 | 7.411 | 6.934 | 6.542 | 6.799 |
| 51162 | EGFL7 | 1.371 | 0.001 | 0.010 | up-reg. | 10.947 | 10.918 | 10.936 | 9.609 | 9.460 | 9.618 |
| 51297 | BPIFA1 | 0.738 | 0.017 | 0.084 | up-reg. | 7.709 | 7.811 | 7.707 | 6.735 | 7.012 | 7.265 |
| 51298 | THEG | 1.179 | 0.028 | 0.017 | up-reg. | 12.486 | 12.237 | 12.816 | 10.868 | 11.816 | 11.321 |
| 51306 | FAM13B | 0.906 | 0.048 | 0.044 | up-reg. | 5.041 | 4.883 | 4.269 | 3.817 | 3.475 | 4.183 |
| 51316 | PLAC8 | 0.927 | 0.027 | 0.041 | up-reg. | 6.948 | 6.593 | 6.694 | 5.479 | 5.738 | 6.239 |
| 51339 | DACT1 | 0.888 | 0.013 | 0.048 | up-reg. | 6.646 | 6.591 | 6.523 | 5.393 | 5.756 | 5.946 |
| 51341 | ZBTB7A | 1.242 | 0.006 | 0.014 | up-reg. | 9.126 | 8.891 | 8.998 | 7.500 | 7.851 | 7.937 |
| 51360 | MBTPS2 | 0.771 | 0.033 | 0.075 | up-reg. | 4.335 | 3.755 | 3.707 | 3.277 | 3.234 | 2.973 |
| 51421 | AMOTL2 | 1.299 | 0.014 | 0.012 | up-reg. | 7.147 | 7.429 | 6.970 | 5.893 | 5.516 | 6.240 |
| 51458 | RHCG | 3.126 | 0.005 | 0.000 | up-reg. | 6.673 | 6.377 | 6.150 | 3.465 | 2.649 | 3.707 |
| 51513 | ETV7 | 0.945 | 0.016 | 0.038 | up-reg. | 6.773 | 6.718 | 6.620 | 5.377 | 5.988 | 5.911 |
| 51560 | RAB6B | 1.449 | 0.016 | 0.008 | up-reg. | 6.224 | 5.874 | 5.772 | 4.259 | 5.031 | 4.234 |
| 51594 | NBAS | 1.467 | 0.016 | 0.007 | up-reg. | 5.917 | 5.503 | 5.013 | 4.182 | 3.718 | 4.130 |
| 51645 | PPIL1 | 1.050 | 0.008 | 0.026 | up-reg. | 9.527 | 9.717 | 9.252 | 8.560 | 8.449 | 8.337 |
| 51665 | ASB1 | 0.754 | 0.012 | 0.080 | up-reg. | 11.590 | 11.805 | 11.390 | 10.959 | 10.777 | 10.788 |
| 51678 | MPP6 | 0.885 | 0.030 | 0.048 | up-reg. | 5.617 | 5.170 | 5.064 | 4.445 | 4.065 | 4.686 |
| 51704 | GPRC5B | 2.495 | 0.002 | 0.001 | up-reg. | 8.330 | 8.245 | 8.240 | 5.775 | 5.489 | 6.066 |
| 51706 | CYB5R1 | 1.223 | 0.003 | 0.014 | up-reg. | 11.128 | 11.277 | 11.017 | 9.847 | 9.882 | 10.023 |
| 51714 | SELENOT | 1.753 | 0.003 | 0.004 | up-reg. | 10.167 | 10.273 | 9.932 | 8.530 | 8.138 | 8.445 |
| 51760 | SYT17 | 1.069 | 0.005 | 0.024 | up-reg. | 7.539 | 7.469 | 7.337 | 6.174 | 6.435 | 6.527 |
| 51807 | TUBA8 | 0.955 | 0.007 | 0.036 | up-reg. | 7.587 | 7.926 | 7.568 | 6.647 | 6.826 | 6.742 |
| 51816 | ADA2 | 0.751 | 0.012 | 0.080 | up-reg. | 6.963 | 6.997 | 7.332 | 6.304 | 6.467 | 6.270 |
| 53358 | SHC3 | 1.093 | 0.031 | 0.022 | up-reg. | 6.458 | 6.694 | 5.716 | 5.060 | 5.155 | 5.374 |
| 53616 | ADAM22 | 1.021 | 0.011 | 0.028 | up-reg. | 5.024 | 4.621 | 5.031 | 3.800 | 3.727 | 4.087 |
| 53828 | FXYD4 | 1.098 | 0.025 | 0.022 | up-reg. | 12.508 | 12.291 | 12.756 | 10.999 | 11.861 | 11.399 |
| 53917 | RAB24 | 1.190 | 0.018 | 0.016 | up-reg. | 8.412 | 7.946 | 7.916 | 7.300 | 6.723 | 6.680 |
| 53919 | SLCO1C1 | 1.218 | 0.036 | 0.015 | up-reg. | 8.391 | 8.113 | 8.675 | 6.519 | 7.462 | 7.542 |
| 53942 | CNTN5 | 1.350 | 0.039 | 0.010 | up-reg. | 5.321 | 5.480 | 5.414 | 3.375 | 3.969 | 4.822 |
| 54434 | SSH1 | 0.735 | 0.027 | 0.085 | up-reg. | 6.778 | 6.753 | 6.495 | 5.769 | 5.759 | 6.292 |
| 54435 | 0 | 0.922 | 0.024 | 0.042 | up-reg. | 9.640 | 8.989 | 9.422 | 8.511 | 8.608 | 8.168 |
| 54456 | MOV10L1 | 1.070 | 0.015 | 0.024 | up-reg. | 8.460 | 7.954 | 7.925 | 6.795 | 7.191 | 7.143 |
| 54543 | TOMM7 | 1.318 | 0.003 | 0.011 | up-reg. | 7.279 | 7.000 | 7.265 | 5.838 | 5.771 | 5.980 |
| 54626 | HES2 | 0.711 | 0.025 | 0.094 | up-reg. | 4.735 | 4.471 | 4.271 | 3.772 | 3.998 | 3.575 |
| 54819 | ZCCHC10 | 1.008 | 0.022 | 0.030 | up-reg. | 7.318 | 7.230 | 7.096 | 5.854 | 6.119 | 6.648 |
| 54863 | TOR4A | 0.825 | 0.008 | 0.061 | up-reg. | 12.191 | 12.370 | 12.014 | 11.488 | 11.318 | 11.293 |
| 54884 | RETSAT | 0.727 | 0.036 | 0.088 | up-reg. | 5.694 | 5.597 | 5.784 | 4.943 | 4.606 | 5.344 |
| 54891 | INO80D | 1.924 | 0.009 | 0.003 | up-reg. | 5.817 | 4.824 | 5.523 | 3.532 | 3.385 | 3.477 |
| 54938 | SARS2 | 1.462 | 0.017 | 0.008 | up-reg. | 8.869 | 8.199 | 8.684 | 6.643 | 7.317 | 7.405 |
| 54972 | TMEM132A | 4.072 | 0.000 | 0.000 | up-reg. | 13.933 | 14.020 | 13.902 | 9.882 | 9.778 | 9.980 |
| 54977 | SLC25A38 | 0.883 | 0.032 | 0.049 | up-reg. | 6.969 | 7.211 | 6.685 | 5.672 | 6.282 | 6.263 |
| 54981 | NMRK1 | 1.604 | 0.017 | 0.005 | up-reg. | 14.909 | 14.801 | 15.007 | 13.011 | 12.915 | 13.978 |
| 55026 | TMEM255A | 0.998 | 0.039 | 0.031 | up-reg. | 5.791 | 6.603 | 5.833 | 4.771 | 5.114 | 5.348 |
| 55054 | ATG16L1 | 1.406 | 0.013 | 0.009 | up-reg. | 5.982 | 5.522 | 5.551 | 4.457 | 3.852 | 4.530 |
| 55193 | PBRM1 | 0.828 | 0.047 | 0.060 | up-reg. | 6.178 | 5.992 | 6.640 | 5.647 | 5.647 | 5.031 |
| 55217 | TMLHE | 1.442 | 0.006 | 0.008 | up-reg. | 12.745 | 12.715 | 12.909 | 11.211 | 11.156 | 11.676 |
| 55227 | LRRC1 | 1.528 | 0.010 | 0.006 | up-reg. | 11.522 | 11.057 | 11.292 | 9.408 | 10.135 | 9.744 |
| 55287 | TMEM40 | 2.239 | 0.014 | 0.001 | up-reg. | 5.675 | 5.149 | 4.799 | 3.598 | 2.876 | 2.432 |
| 55330 | BLOC1S4 | 1.235 | 0.027 | 0.014 | up-reg. | 6.960 | 6.927 | 6.462 | 5.696 | 4.990 | 5.958 |
| 55332 | DRAM1 | 1.461 | 0.011 | 0.008 | up-reg. | 5.837 | 6.029 | 5.546 | 4.604 | 3.922 | 4.504 |
| 55356 | SLC22A15 | 0.719 | 0.019 | 0.091 | up-reg. | 7.213 | 7.200 | 7.472 | 6.305 | 6.703 | 6.718 |
| 55534 | MAML3 | 0.830 | 0.009 | 0.059 | up-reg. | 5.601 | 5.810 | 5.608 | 4.659 | 4.853 | 5.016 |
| 55653 | BCAS4 | 0.821 | 0.030 | 0.062 | up-reg. | 10.807 | 10.113 | 10.260 | 9.431 | 9.548 | 9.737 |
| 55659 | ZNF416 | 0.734 | 0.014 | 0.086 | up-reg. | 4.297 | 4.319 | 4.341 | 3.848 | 3.530 | 3.379 |
| 55703 | POLR3B | 1.536 | 0.032 | 0.006 | up-reg. | 7.400 | 6.662 | 7.340 | 4.931 | 6.227 | 5.637 |
| 55704 | CCDC88A | 1.113 | 0.011 | 0.021 | up-reg. | 7.314 | 6.885 | 7.427 | 5.913 | 6.225 | 6.149 |
| 55741 | EDEM2 | 1.082 | 0.026 | 0.023 | up-reg. | 9.865 | 9.551 | 10.141 | 8.365 | 9.158 | 8.788 |
| 55844 | PPP2R2D | 1.794 | 0.004 | 0.003 | up-reg. | 8.736 | 8.213 | 8.560 | 6.604 | 6.692 | 6.830 |
| 55863 | TMEM126B | 0.907 | 0.013 | 0.044 | up-reg. | 4.962 | 5.425 | 5.280 | 4.491 | 4.180 | 4.275 |
| 55890 | GPRC5C | 1.109 | 0.006 | 0.021 | up-reg. | 7.317 | 7.365 | 7.302 | 6.329 | 5.964 | 6.364 |
| 55897 | MESP1 | 1.583 | 0.017 | 0.005 | up-reg. | 6.430 | 6.828 | 5.907 | 4.805 | 4.454 | 5.157 |
| 56137 | PCDHA12 | 0.709 | 0.039 | 0.095 | up-reg. | 6.826 | 6.761 | 7.147 | 5.846 | 6.485 | 6.275 |
| 56252 | YLPM1 | 0.747 | 0.030 | 0.082 | up-reg. | 5.941 | 5.710 | 5.713 | 4.912 | 4.787 | 5.424 |
| 56259 | CTNNBL1 | 1.477 | 0.010 | 0.007 | up-reg. | 8.604 | 8.195 | 8.689 | 6.681 | 7.097 | 7.280 |
| 56603 | CYP26B1 | 2.798 | 0.001 | 0.001 | up-reg. | 10.192 | 10.330 | 10.158 | 7.554 | 7.208 | 7.524 |
| 56654 | NPDC1 | 0.903 | 0.002 | 0.045 | up-reg. | 13.861 | 13.950 | 13.875 | 13.073 | 12.990 | 12.915 |
| 56663 | VTRNA1-2 | 2.495 | 0.004 | 0.001 | up-reg. | 5.448 | 5.500 | 5.117 | 3.258 | 2.595 | 2.727 |
| 56704 | JPH1 | 1.900 | 0.005 | 0.003 | up-reg. | 6.542 | 5.927 | 6.526 | 4.451 | 4.301 | 4.544 |
| 56850 | GRIPAP1 | 0.823 | 0.003 | 0.061 | up-reg. | 10.595 | 10.595 | 10.496 | 9.786 | 9.800 | 9.631 |
| 56913 | C1GALT1 | 0.740 | 0.013 | 0.084 | up-reg. | 7.450 | 7.717 | 7.316 | 6.694 | 6.682 | 6.885 |
| 56947 | MFF | 0.823 | 0.006 | 0.061 | up-reg. | 5.142 | 5.391 | 5.459 | 4.454 | 4.515 | 4.554 |
| 56961 | SHD | 1.158 | 0.014 | 0.018 | up-reg. | 9.486 | 9.203 | 9.489 | 7.830 | 8.493 | 8.380 |
| 57026 | PDXP | 0.771 | 0.033 | 0.075 | up-reg. | 7.014 | 6.714 | 6.888 | 5.918 | 5.872 | 6.513 |
| 57053 | CHRNA10 | 1.111 | 0.005 | 0.021 | up-reg. | 6.358 | 6.032 | 6.330 | 5.049 | 5.087 | 5.252 |
| 57088 | PLSCR4 | 1.773 | 0.032 | 0.004 | up-reg. | 5.677 | 5.812 | 5.646 | 4.869 | 3.138 | 3.809 |
| 57104 | PNPLA2 | 0.725 | 0.003 | 0.089 | up-reg. | 12.779 | 12.825 | 12.779 | 12.172 | 11.973 | 12.063 |
| 57132 | CHMP1B | 1.107 | 0.040 | 0.021 | up-reg. | 6.178 | 6.287 | 5.887 | 5.123 | 4.390 | 5.518 |
| 57182 | ANKRD50 | 0.759 | 0.015 | 0.078 | up-reg. | 6.190 | 5.878 | 6.033 | 5.519 | 5.135 | 5.172 |
| 57326 | PBXIP1 | 1.508 | 0.002 | 0.007 | up-reg. | 10.573 | 10.756 | 10.516 | 9.219 | 9.047 | 9.055 |
| 57332 | CBX8 | 0.996 | 0.024 | 0.031 | up-reg. | 11.871 | 11.693 | 11.993 | 10.392 | 11.045 | 11.131 |
| 57419 | SLC24A3 | 1.633 | 0.013 | 0.005 | up-reg. | 6.994 | 6.383 | 6.145 | 4.580 | 4.918 | 5.126 |
| 57463 | AMIGO1 | 1.660 | 0.011 | 0.005 | up-reg. | 6.587 | 6.760 | 6.229 | 4.950 | 4.424 | 5.221 |
| 57471 | ERMN | 1.280 | 0.018 | 0.012 | up-reg. | 5.906 | 5.320 | 5.351 | 4.205 | 3.906 | 4.624 |
| 57504 | MTA3 | 0.817 | 0.034 | 0.063 | up-reg. | 6.085 | 5.607 | 5.297 | 4.868 | 4.701 | 4.970 |
| 57545 | CC2D2A | 0.857 | 0.009 | 0.054 | up-reg. | 6.316 | 5.968 | 6.240 | 5.155 | 5.409 | 5.390 |
| 57589 | RIC1 | 0.726 | 0.045 | 0.089 | up-reg. | 5.305 | 5.280 | 5.059 | 4.214 | 4.310 | 4.944 |
| 57595 | PDZD4 | 1.633 | 0.008 | 0.005 | up-reg. | 6.864 | 6.382 | 6.630 | 4.745 | 4.876 | 5.355 |
| 57604 | TRMT9B | 0.770 | 0.043 | 0.075 | up-reg. | 6.444 | 5.932 | 6.364 | 5.358 | 5.214 | 5.859 |
| 57717 | PCDHB16 | 1.229 | 0.011 | 0.014 | up-reg. | 4.227 | 3.873 | 3.576 | 2.708 | 2.477 | 2.804 |
| 57718 | PPP4R4 | 0.719 | 0.000 | 0.091 | up-reg. | 3.962 | 3.941 | 3.986 | 3.218 | 3.244 | 3.271 |
| 57724 | EPG5 | 1.157 | 0.033 | 0.018 | up-reg. | 5.005 | 4.456 | 4.852 | 3.430 | 3.218 | 4.192 |
| 57795 | BRINP2 | 0.997 | 0.005 | 0.031 | up-reg. | 8.102 | 7.935 | 8.046 | 6.825 | 7.135 | 7.132 |
| 58189 | WFDC1 | 1.833 | 0.000 | 0.003 | up-reg. | 11.402 | 11.434 | 11.370 | 9.534 | 9.631 | 9.541 |
| 58494 | JAM2 | 1.599 | 0.009 | 0.005 | up-reg. | 6.473 | 6.445 | 6.113 | 4.552 | 4.509 | 5.174 |
| 58529 | MYOZ1 | 1.955 | 0.007 | 0.002 | up-reg. | 5.522 | 4.941 | 5.218 | 3.236 | 2.947 | 3.631 |
| 58986 | PGAP6 | 0.748 | 0.010 | 0.082 | up-reg. | 9.530 | 9.744 | 9.369 | 8.885 | 8.684 | 8.830 |
| 60493 | FASTKD5 | 1.088 | 0.017 | 0.023 | up-reg. | 7.025 | 7.045 | 6.738 | 5.870 | 5.473 | 6.202 |
| 60560 | NAA35 | 0.705 | 0.026 | 0.096 | up-reg. | 5.953 | 5.593 | 5.370 | 5.067 | 4.808 | 4.925 |
| 60598 | KCNK15 | 1.114 | 0.012 | 0.021 | up-reg. | 13.684 | 13.535 | 13.820 | 12.232 | 12.853 | 12.613 |
| 63874 | ABHD4 | 1.101 | 0.023 | 0.022 | up-reg. | 12.382 | 11.732 | 12.504 | 10.971 | 11.366 | 10.978 |
| 64061 | TSPYL2 | 0.977 | 0.017 | 0.034 | up-reg. | 5.337 | 5.379 | 4.995 | 4.028 | 4.159 | 4.594 |
| 64081 | PBLD | 0.809 | 0.016 | 0.065 | up-reg. | 6.919 | 6.848 | 6.716 | 5.860 | 5.866 | 6.331 |
| 64089 | SNX16 | 0.872 | 0.037 | 0.051 | up-reg. | 4.745 | 4.400 | 4.286 | 4.024 | 3.561 | 3.231 |
| 64114 | TMBIM1 | 0.971 | 0.043 | 0.034 | up-reg. | 5.915 | 5.493 | 5.134 | 4.118 | 4.646 | 4.866 |
| 64115 | VSIR | 1.390 | 0.010 | 0.009 | up-reg. | 9.808 | 9.236 | 9.874 | 8.036 | 8.365 | 8.350 |
| 64130 | LIN7B | 1.127 | 0.005 | 0.020 | up-reg. | 8.744 | 8.490 | 8.668 | 7.606 | 7.314 | 7.601 |
| 64131 | XYLT1 | 1.419 | 0.013 | 0.009 | up-reg. | 6.829 | 6.503 | 7.000 | 4.939 | 5.461 | 5.676 |
| 64184 | EDDM3B | 1.963 | 0.010 | 0.002 | up-reg. | 12.709 | 12.493 | 12.787 | 10.414 | 10.386 | 11.301 |
| 64595 | USP9Y | 1.576 | 0.041 | 0.006 | up-reg. | 10.161 | 10.040 | 10.304 | 7.608 | 9.237 | 8.933 |
| 64651 | CSRNP1 | 1.100 | 0.001 | 0.022 | up-reg. | 12.284 | 12.362 | 12.237 | 11.277 | 11.139 | 11.166 |
| 64699 | TMPRSS3 | 1.278 | 0.025 | 0.012 | up-reg. | 9.941 | 9.794 | 10.555 | 8.814 | 9.228 | 8.414 |
| 64761 | PARP12 | 0.817 | 0.019 | 0.063 | up-reg. | 6.214 | 5.797 | 5.676 | 4.909 | 5.174 | 5.152 |
| 64776 | C11orf1 | 0.868 | 0.014 | 0.051 | up-reg. | 7.209 | 6.925 | 7.365 | 6.106 | 6.483 | 6.305 |
| 64866 | CDCP1 | 1.207 | 0.008 | 0.015 | up-reg. | 8.506 | 8.361 | 8.604 | 6.975 | 7.424 | 7.450 |
| 65018 | PINK1 | 1.816 | 0.003 | 0.003 | up-reg. | 8.378 | 8.233 | 8.242 | 6.537 | 6.177 | 6.690 |
| 65059 | RAPH1 | 0.755 | 0.036 | 0.079 | up-reg. | 7.019 | 6.814 | 6.878 | 5.740 | 6.215 | 6.489 |
| 65124 | SOWAHC | 1.689 | 0.003 | 0.004 | up-reg. | 9.544 | 9.771 | 9.469 | 8.080 | 7.771 | 7.868 |
| 65249 | ZSWIM4 | 1.277 | 0.004 | 0.012 | up-reg. | 9.064 | 8.980 | 8.831 | 7.489 | 7.761 | 7.796 |
| 65983 | GRAMD2B | 0.821 | 0.015 | 0.062 | up-reg. | 7.005 | 6.686 | 6.990 | 5.931 | 5.958 | 6.328 |
| 65992 | DDRGK1 | 0.734 | 0.008 | 0.086 | up-reg. | 9.212 | 9.375 | 9.216 | 8.708 | 8.422 | 8.471 |
| 79001 | VKORC1 | 0.854 | 0.034 | 0.054 | up-reg. | 6.023 | 5.928 | 5.820 | 5.450 | 4.616 | 5.145 |
| 79648 | MCPH1 | 1.063 | 0.038 | 0.025 | up-reg. | 6.870 | 6.493 | 6.742 | 5.057 | 5.732 | 6.125 |
| 79650 | USB1 | 0.822 | 0.008 | 0.062 | up-reg. | 12.148 | 12.378 | 12.027 | 11.438 | 11.269 | 11.381 |
| 79674 | VEPH1 | 1.325 | 0.013 | 0.011 | up-reg. | 12.137 | 12.084 | 12.284 | 10.385 | 10.952 | 11.196 |
| 79693 | YRDC | 1.731 | 0.003 | 0.004 | up-reg. | 6.707 | 6.729 | 6.635 | 4.859 | 4.798 | 5.222 |
| 79772 | MCTP1 | 0.800 | 0.021 | 0.067 | up-reg. | 7.043 | 7.021 | 7.417 | 6.464 | 6.532 | 6.087 |
| 79820 | CATSPERB | 0.736 | 0.035 | 0.085 | up-reg. | 5.248 | 4.953 | 4.891 | 3.919 | 4.546 | 4.419 |
| 79829 | NAA40 | 1.053 | 0.008 | 0.026 | up-reg. | 8.350 | 8.350 | 8.422 | 7.065 | 7.316 | 7.580 |
| 79847 | MFSD13A | 1.578 | 0.012 | 0.006 | up-reg. | 6.018 | 5.466 | 5.657 | 3.721 | 4.528 | 4.159 |
| 79896 | THNSL1 | 1.004 | 0.039 | 0.030 | up-reg. | 5.172 | 4.956 | 5.923 | 4.154 | 4.322 | 4.562 |
| 79939 | SLC35E1 | 0.721 | 0.015 | 0.090 | up-reg. | 9.782 | 9.923 | 9.691 | 9.285 | 8.849 | 9.098 |
| 79993 | ELOVL7 | 0.889 | 0.012 | 0.047 | up-reg. | 7.730 | 7.809 | 7.607 | 6.723 | 6.632 | 7.122 |
| 80023 | NRSN2 | 0.764 | 0.041 | 0.077 | up-reg. | 4.861 | 5.335 | 4.838 | 3.878 | 4.422 | 4.442 |
| 80149 | ZC3H12A | 1.689 | 0.000 | 0.004 | up-reg. | 8.969 | 9.001 | 8.917 | 7.374 | 7.233 | 7.215 |
| 80185 | TTI2 | 0.948 | 0.010 | 0.038 | up-reg. | 6.866 | 6.520 | 6.952 | 5.774 | 5.732 | 5.988 |
| 80207 | OPA3 | 0.961 | 0.018 | 0.036 | up-reg. | 6.303 | 6.358 | 5.793 | 4.995 | 5.241 | 5.335 |
| 80235 | PIGZ | 1.585 | 0.002 | 0.005 | up-reg. | 7.173 | 7.147 | 6.988 | 5.467 | 5.433 | 5.653 |
| 80255 | SLC35F5 | 1.633 | 0.039 | 0.005 | up-reg. | 5.759 | 5.291 | 4.700 | 3.923 | 2.801 | 4.127 |
| 80256 | FAM214B | 1.466 | 0.001 | 0.007 | up-reg. | 10.171 | 10.293 | 10.175 | 8.864 | 8.694 | 8.681 |
| 80274 | SCUBE1 | 0.984 | 0.031 | 0.033 | up-reg. | 7.772 | 7.703 | 7.751 | 6.231 | 6.879 | 7.165 |
| 80309 | SPHKAP | 1.449 | 0.004 | 0.008 | up-reg. | 5.577 | 5.101 | 5.290 | 3.859 | 3.939 | 3.824 |
| 80320 | SP6 | 1.678 | 0.031 | 0.004 | up-reg. | 6.808 | 6.694 | 6.418 | 4.037 | 5.454 | 5.396 |
| 80331 | DNAJC5 | 1.111 | 0.001 | 0.021 | up-reg. | 10.304 | 10.312 | 10.239 | 9.266 | 9.118 | 9.137 |
| 80764 | THAP7 | 1.462 | 0.019 | 0.008 | up-reg. | 5.891 | 5.409 | 5.734 | 3.814 | 4.046 | 4.787 |
| 80816 | ASXL3 | 1.484 | 0.014 | 0.007 | up-reg. | 6.670 | 6.166 | 6.131 | 4.493 | 4.776 | 5.246 |
| 80820 | EEPD1 | 1.044 | 0.041 | 0.027 | up-reg. | 5.235 | 5.387 | 5.113 | 3.880 | 3.871 | 4.852 |
| 80896 | NPL | 1.203 | 0.011 | 0.015 | up-reg. | 3.734 | 4.411 | 4.017 | 2.857 | 2.754 | 2.941 |
| 81282 | OR51G2 | 1.034 | 0.037 | 0.027 | up-reg. | 5.119 | 4.674 | 5.479 | 3.869 | 3.814 | 4.486 |
| 81793 | TLR10 | 0.780 | 0.046 | 0.072 | up-reg. | 4.679 | 4.341 | 3.941 | 3.837 | 3.310 | 3.472 |
| 81854 | 0 | 1.316 | 0.029 | 0.011 | up-reg. | 4.744 | 4.708 | 4.580 | 3.539 | 2.670 | 3.873 |
| 81870 | KRTAP9-9 | 1.553 | 0.023 | 0.006 | up-reg. | 6.747 | 6.203 | 6.839 | 4.393 | 5.287 | 5.450 |
| 83445 | GSG1 | 1.113 | 0.040 | 0.021 | up-reg. | 7.069 | 6.556 | 7.162 | 5.263 | 5.906 | 6.278 |
| 83593 | RASSF5 | 1.010 | 0.007 | 0.030 | up-reg. | 6.957 | 6.786 | 6.666 | 5.603 | 5.971 | 5.805 |
| 83594 | NUDT12 | 1.673 | 0.019 | 0.004 | up-reg. | 7.030 | 6.827 | 7.299 | 4.677 | 5.721 | 5.740 |
| 83607 | AMMECR1L | 0.900 | 0.012 | 0.045 | up-reg. | 12.427 | 11.955 | 12.191 | 11.133 | 11.447 | 11.292 |
| 83636 | C19orf12 | 0.895 | 0.018 | 0.046 | up-reg. | 7.937 | 7.385 | 7.768 | 7.011 | 6.671 | 6.722 |
| 83641 | FAM107B | 1.364 | 0.010 | 0.010 | up-reg. | 9.081 | 8.740 | 9.162 | 7.375 | 7.957 | 7.557 |
| 83648 | FAM167A | 0.919 | 0.011 | 0.042 | up-reg. | 8.308 | 7.947 | 8.095 | 7.319 | 6.957 | 7.318 |
| 83657 | DYNLRB2 | 1.549 | 0.013 | 0.006 | up-reg. | 5.960 | 5.486 | 5.129 | 4.271 | 3.741 | 3.917 |
| 83658 | DYNLRB1 | 1.449 | 0.007 | 0.008 | up-reg. | 7.395 | 7.096 | 7.387 | 5.516 | 6.099 | 5.915 |
| 83666 | PARP9 | 0.977 | 0.013 | 0.034 | up-reg. | 6.727 | 6.648 | 6.849 | 5.487 | 5.742 | 6.067 |
| 83873 | GPR61 | 0.832 | 0.022 | 0.059 | up-reg. | 4.807 | 4.758 | 5.125 | 4.364 | 4.031 | 3.799 |
| 83874 | TBC1D10A | 0.963 | 0.012 | 0.035 | up-reg. | 10.244 | 10.475 | 9.993 | 9.480 | 9.142 | 9.203 |
| 83876 | MRO | 0.892 | 0.012 | 0.047 | up-reg. | 6.144 | 6.075 | 6.261 | 5.157 | 5.558 | 5.088 |
| 83895 | KRTAP1-5 | 2.591 | 0.001 | 0.001 | up-reg. | 11.834 | 11.980 | 11.709 | 9.324 | 9.053 | 9.375 |
| 83896 | KRTAP3-1 | 4.445 | 0.005 | 0.000 | up-reg. | 9.792 | 9.904 | 9.768 | 4.573 | 5.350 | 6.207 |
| 83931 | STK40 | 1.635 | 0.009 | 0.005 | up-reg. | 11.434 | 11.131 | 11.548 | 9.362 | 9.756 | 10.092 |
| 83942 | TSSK1B | 1.501 | 0.005 | 0.007 | up-reg. | 12.314 | 12.057 | 11.980 | 10.439 | 10.853 | 10.557 |
| 83990 | BRIP1 | 2.209 | 0.019 | 0.001 | up-reg. | 6.038 | 6.205 | 5.817 | 3.145 | 3.545 | 4.741 |
| 84236 | RHBDD1 | 1.108 | 0.032 | 0.021 | up-reg. | 13.073 | 12.871 | 13.471 | 11.625 | 12.525 | 11.942 |
| 84275 | SLC25A33 | 0.823 | 0.027 | 0.061 | up-reg. | 6.510 | 6.371 | 6.301 | 5.609 | 5.186 | 5.920 |
| 84322 | C18orf12 | 1.464 | 0.011 | 0.008 | up-reg. | 12.302 | 12.027 | 12.406 | 10.369 | 11.123 | 10.850 |
| 84331 | MCRIP2 | 0.831 | 0.000 | 0.059 | up-reg. | 10.937 | 10.987 | 10.978 | 10.164 | 10.115 | 10.131 |
| 84365 | NIFK | 0.873 | 0.027 | 0.050 | up-reg. | 4.446 | 4.251 | 4.899 | 3.500 | 3.552 | 3.926 |
| 84439 | HHIPL1 | 1.023 | 0.005 | 0.028 | up-reg. | 8.955 | 9.225 | 9.111 | 8.153 | 7.897 | 8.170 |
| 84513 | PLPP5 | 0.699 | 0.007 | 0.099 | up-reg. | 7.761 | 7.573 | 7.542 | 6.870 | 6.851 | 7.059 |
| 84614 | ZBTB37 | 1.293 | 0.018 | 0.012 | up-reg. | 7.127 | 6.674 | 6.797 | 5.354 | 5.303 | 6.063 |
| 84649 | DGAT2 | 1.174 | 0.011 | 0.017 | up-reg. | 5.165 | 5.132 | 5.395 | 4.417 | 3.904 | 3.849 |
| 84657 | LINC00852 | 1.497 | 0.008 | 0.007 | up-reg. | 6.824 | 6.268 | 6.503 | 4.946 | 4.856 | 5.301 |
| 84667 | HES7 | 0.804 | 0.035 | 0.066 | up-reg. | 9.405 | 9.150 | 9.509 | 8.176 | 8.549 | 8.926 |
| 84675 | TRIM55 | 1.217 | 0.005 | 0.015 | up-reg. | 10.155 | 9.844 | 10.150 | 8.834 | 8.701 | 8.965 |
| 84680 | ACCS | 1.403 | 0.043 | 0.009 | up-reg. | 5.466 | 4.090 | 4.247 | 3.203 | 2.955 | 3.435 |
| 84769 | MPV17L2 | 1.176 | 0.024 | 0.017 | up-reg. | 6.431 | 6.260 | 6.046 | 5.029 | 4.614 | 5.568 |
| 84808 | PERM1 | 1.283 | 0.011 | 0.012 | up-reg. | 8.891 | 8.430 | 9.062 | 7.351 | 7.704 | 7.481 |
| 84934 | RITA1 | 0.808 | 0.025 | 0.065 | up-reg. | 7.164 | 6.981 | 6.733 | 5.819 | 6.299 | 6.338 |
| 84970 | C1orf94 | 1.058 | 0.011 | 0.025 | up-reg. | 13.893 | 13.593 | 14.069 | 12.673 | 13.035 | 12.673 |
| 84987 | COX14 | 1.047 | 0.003 | 0.026 | up-reg. | 12.964 | 13.035 | 12.838 | 11.977 | 11.786 | 11.931 |
| 84996 | URB1-AS1 | 0.734 | 0.044 | 0.086 | up-reg. | 6.537 | 6.747 | 6.258 | 5.836 | 5.414 | 6.088 |
| 85027 | SMIM3 | 1.007 | 0.006 | 0.030 | up-reg. | 12.309 | 12.504 | 12.121 | 11.373 | 11.231 | 11.308 |
| 85300 | ATCAY | 1.039 | 0.027 | 0.027 | up-reg. | 4.610 | 4.356 | 4.239 | 3.050 | 3.868 | 3.171 |
| 85414 | SLC45A3 | 1.934 | 0.002 | 0.002 | up-reg. | 6.049 | 6.136 | 5.770 | 4.035 | 4.105 | 4.012 |
| 85457 | CIPC | 1.063 | 0.004 | 0.025 | up-reg. | 6.265 | 5.954 | 6.188 | 5.051 | 5.039 | 5.128 |
| 89777 | SERPINB12 | 1.110 | 0.035 | 0.021 | up-reg. | 9.356 | 9.131 | 9.627 | 7.665 | 8.629 | 8.488 |
| 89822 | KCNK17 | 1.350 | 0.004 | 0.010 | up-reg. | 10.621 | 10.236 | 10.642 | 9.174 | 9.123 | 9.153 |
| 90203 | SNX21 | 1.170 | 0.001 | 0.017 | up-reg. | 9.678 | 9.616 | 9.573 | 8.452 | 8.544 | 8.360 |
| 90407 | TMEM41A | 1.160 | 0.032 | 0.018 | up-reg. | 4.032 | 3.754 | 4.831 | 3.131 | 2.867 | 3.140 |
| 90693 | CCDC126 | 0.900 | 0.042 | 0.045 | up-reg. | 8.427 | 8.090 | 8.380 | 6.858 | 7.676 | 7.662 |
| 90809 | PIP4P1 | 0.720 | 0.011 | 0.091 | up-reg. | 8.669 | 8.513 | 8.820 | 7.875 | 8.120 | 7.846 |
| 90933 | TRIM41 | 1.100 | 0.047 | 0.022 | up-reg. | 4.498 | 3.872 | 4.865 | 2.933 | 3.250 | 3.752 |
| 91107 | TRIM47 | 0.820 | 0.007 | 0.062 | up-reg. | 9.515 | 9.255 | 9.577 | 8.532 | 8.710 | 8.646 |
| 91147 | TMEM67 | 0.950 | 0.037 | 0.037 | up-reg. | 6.733 | 6.401 | 6.204 | 5.016 | 5.641 | 5.830 |
| 91181 | NUP210L | 1.306 | 0.016 | 0.011 | up-reg. | 8.513 | 8.173 | 8.619 | 6.686 | 7.229 | 7.472 |
| 91355 | LRP5L | 1.084 | 0.003 | 0.023 | up-reg. | 5.296 | 5.055 | 5.100 | 4.095 | 4.135 | 3.969 |
| 91662 | NLRP12 | 1.291 | 0.009 | 0.012 | up-reg. | 9.567 | 9.468 | 9.884 | 8.058 | 8.465 | 8.521 |
| 91937 | TIMD4 | 1.577 | 0.029 | 0.006 | up-reg. | 6.235 | 5.745 | 6.107 | 3.755 | 4.436 | 5.166 |
| 92017 | SNX29 | 0.726 | 0.023 | 0.089 | up-reg. | 5.148 | 4.990 | 5.072 | 4.299 | 4.066 | 4.666 |
| 92140 | MTDH | 0.738 | 0.002 | 0.085 | up-reg. | 6.990 | 7.006 | 6.997 | 6.334 | 6.184 | 6.261 |
| 92255 | LMBRD2 | 1.536 | 0.009 | 0.006 | up-reg. | 4.702 | 4.547 | 4.558 | 3.512 | 2.910 | 2.774 |
| 92340 | PRR29 | 1.387 | 0.006 | 0.009 | up-reg. | 9.771 | 9.422 | 9.834 | 8.081 | 8.475 | 8.311 |
| 92689 | FAM114A1 | 1.519 | 0.009 | 0.006 | up-reg. | 6.592 | 6.419 | 6.030 | 5.166 | 4.681 | 4.636 |
| 93621 | MRFAP1 | 2.103 | 0.007 | 0.002 | up-reg. | 7.366 | 6.604 | 7.095 | 4.763 | 4.737 | 5.257 |
| 94086 | HSPB9 | 0.911 | 0.029 | 0.043 | up-reg. | 7.100 | 6.795 | 6.386 | 5.709 | 5.707 | 6.131 |
| 94103 | ORMDL3 | 0.971 | 0.003 | 0.034 | up-reg. | 11.747 | 11.871 | 11.738 | 10.920 | 10.720 | 10.804 |
| 94122 | SYTL5 | 1.047 | 0.020 | 0.026 | up-reg. | 6.583 | 6.029 | 6.086 | 4.931 | 5.482 | 5.145 |
| 95681 | CEP41 | 1.252 | 0.013 | 0.013 | up-reg. | 6.112 | 5.817 | 5.740 | 4.791 | 4.239 | 4.886 |
| 112611 | RWDD2A | 0.967 | 0.028 | 0.035 | up-reg. | 6.058 | 6.162 | 5.725 | 4.946 | 4.659 | 5.439 |
| 113115 | MTFR2 | 0.860 | 0.022 | 0.053 | up-reg. | 6.086 | 5.620 | 5.643 | 4.964 | 4.664 | 5.140 |
| 113540 | CMTM1 | 1.293 | 0.010 | 0.012 | up-reg. | 6.880 | 6.420 | 6.547 | 5.229 | 5.096 | 5.645 |
| 114034 | TOE1 | 1.616 | 0.012 | 0.005 | up-reg. | 8.065 | 7.424 | 7.984 | 5.812 | 6.291 | 6.524 |
| 114819 | CROCCP3 | 1.125 | 0.010 | 0.020 | up-reg. | 6.229 | 6.080 | 6.137 | 5.097 | 4.680 | 5.293 |
| 114904 | C1QTNF6 | 0.911 | 0.007 | 0.043 | up-reg. | 8.959 | 8.790 | 8.963 | 7.933 | 8.207 | 7.839 |
| 115265 | DDIT4L | 1.277 | 0.004 | 0.012 | up-reg. | 8.698 | 8.794 | 8.472 | 7.360 | 7.259 | 7.514 |
| 116236 | ABHD15 | 1.427 | 0.011 | 0.008 | up-reg. | 11.597 | 11.592 | 11.863 | 9.881 | 10.657 | 10.234 |
| 116369 | SLC26A8 | 0.908 | 0.035 | 0.044 | up-reg. | 4.893 | 4.222 | 4.939 | 3.701 | 3.579 | 4.051 |
| 116444 | GRIN3B | 0.733 | 0.006 | 0.086 | up-reg. | 6.980 | 7.030 | 6.999 | 6.218 | 6.143 | 6.450 |
| 116729 | PPP1R27 | 1.088 | 0.016 | 0.023 | up-reg. | 5.301 | 4.876 | 4.552 | 3.903 | 3.713 | 3.847 |
| 120796 | OR56A1 | 1.304 | 0.039 | 0.011 | up-reg. | 6.282 | 5.882 | 6.142 | 4.029 | 5.057 | 5.308 |
| 121227 | LRIG3 | 1.119 | 0.023 | 0.020 | up-reg. | 9.144 | 8.529 | 9.236 | 7.765 | 8.170 | 7.618 |
| 121599 | SPIC | 0.884 | 0.013 | 0.048 | up-reg. | 15.465 | 14.909 | 15.268 | 14.276 | 14.337 | 14.375 |
| 123775 | C16orf46 | 0.713 | 0.038 | 0.094 | up-reg. | 5.558 | 4.942 | 5.213 | 4.275 | 4.566 | 4.732 |
| 123879 | DCUN1D3 | 1.923 | 0.002 | 0.003 | up-reg. | 11.001 | 11.211 | 10.921 | 9.307 | 8.987 | 9.070 |
| 124359 | CDYL2 | 1.275 | 0.014 | 0.012 | up-reg. | 6.806 | 7.416 | 6.934 | 5.736 | 5.522 | 6.073 |
| 124590 | USH1G | 1.777 | 0.015 | 0.004 | up-reg. | 6.988 | 6.380 | 6.459 | 4.408 | 4.694 | 5.395 |
| 124602 | KIF19 | 1.558 | 0.006 | 0.006 | up-reg. | 7.204 | 6.889 | 6.720 | 5.237 | 5.247 | 5.657 |
| 124961 | ZFP3 | 1.479 | 0.003 | 0.007 | up-reg. | 9.480 | 9.216 | 9.377 | 7.693 | 8.013 | 7.929 |
| 126259 | TMIGD2 | 1.115 | 0.014 | 0.021 | up-reg. | 10.108 | 9.760 | 10.331 | 8.677 | 9.131 | 9.047 |
| 126298 | IRGQ | 1.313 | 0.009 | 0.011 | up-reg. | 9.583 | 9.098 | 9.505 | 7.803 | 8.246 | 8.198 |
| 126393 | HSPB6 | 1.671 | 0.012 | 0.004 | up-reg. | 7.918 | 7.602 | 7.941 | 5.766 | 5.992 | 6.691 |
| 126410 | CYP4F22 | 1.221 | 0.043 | 0.014 | up-reg. | 5.561 | 4.620 | 4.344 | 3.933 | 3.508 | 3.420 |
| 126433 | FBXO27 | 1.388 | 0.002 | 0.009 | up-reg. | 8.487 | 8.390 | 8.516 | 6.934 | 7.071 | 7.225 |
| 126767 | AADACL3 | 1.663 | 0.011 | 0.005 | up-reg. | 6.478 | 5.713 | 6.038 | 4.527 | 4.048 | 4.664 |
| 126859 | AXDND1 | 1.445 | 0.005 | 0.008 | up-reg. | 9.559 | 9.250 | 9.527 | 7.758 | 8.122 | 8.120 |
| 128826 | MIR1-1HG | 1.034 | 0.021 | 0.027 | up-reg. | 5.639 | 5.645 | 5.485 | 4.279 | 4.364 | 5.023 |
| 128977 | C22orf39 | 0.873 | 0.008 | 0.050 | up-reg. | 7.420 | 7.312 | 7.126 | 6.233 | 6.481 | 6.525 |
| 129607 | CMPK2 | 0.800 | 0.040 | 0.067 | up-reg. | 4.827 | 4.154 | 4.964 | 3.872 | 3.768 | 3.905 |
| 130075 | OR9A4 | 1.414 | 0.030 | 0.009 | up-reg. | 6.153 | 5.987 | 6.384 | 4.507 | 4.274 | 5.501 |
| 130162 | CLHC1 | 0.993 | 0.025 | 0.032 | up-reg. | 13.142 | 12.774 | 12.463 | 11.548 | 12.101 | 11.753 |
| 131578 | LRRC15 | 4.604 | 0.000 | 0.000 | up-reg. | 12.097 | 12.279 | 11.942 | 7.521 | 7.392 | 7.592 |
| 131583 | FAM43A | 1.163 | 0.002 | 0.017 | up-reg. | 9.832 | 9.847 | 9.746 | 8.790 | 8.600 | 8.547 |
| 131909 | 0 | 0.918 | 0.020 | 0.042 | up-reg. | 3.873 | 3.474 | 3.615 | 2.614 | 2.508 | 3.085 |
| 133874 | C5orf58 | 1.032 | 0.020 | 0.027 | up-reg. | 7.290 | 6.755 | 6.599 | 5.656 | 6.052 | 5.841 |
| 133923 | ZNF474 | 1.448 | 0.027 | 0.008 | up-reg. | 9.932 | 9.581 | 10.147 | 7.749 | 8.844 | 8.722 |
| 134111 | UBE2QL1 | 0.820 | 0.032 | 0.062 | up-reg. | 15.043 | 14.969 | 14.874 | 13.711 | 14.504 | 14.210 |
| 135398 | C6orf141 | 1.164 | 0.047 | 0.017 | up-reg. | 12.770 | 12.756 | 13.073 | 10.941 | 12.006 | 12.159 |
| 135458 | HUS1B | 0.993 | 0.015 | 0.032 | up-reg. | 4.982 | 4.856 | 5.044 | 4.098 | 3.595 | 4.209 |
| 135932 | TMEM139 | 1.459 | 0.008 | 0.008 | up-reg. | 5.749 | 5.259 | 5.549 | 4.372 | 3.862 | 3.947 |
| 139562 | OTUD6A | 1.516 | 0.007 | 0.007 | up-reg. | 9.627 | 9.269 | 9.683 | 7.699 | 8.235 | 8.097 |
| 140461 | ASB8 | 1.503 | 0.025 | 0.007 | up-reg. | 5.630 | 5.300 | 5.774 | 3.447 | 4.062 | 4.688 |
| 140606 | SELENOM | 1.109 | 0.000 | 0.021 | up-reg. | 13.997 | 13.966 | 13.914 | 12.808 | 12.841 | 12.903 |
| 140609 | NEK7 | 0.717 | 0.041 | 0.092 | up-reg. | 7.407 | 7.427 | 7.266 | 6.425 | 6.427 | 7.098 |
| 140683 | BPIFA2 | 0.942 | 0.009 | 0.038 | up-reg. | 6.239 | 6.118 | 6.255 | 5.043 | 5.229 | 5.513 |
| 140706 | CCM2L | 1.268 | 0.023 | 0.013 | up-reg. | 11.014 | 10.498 | 11.354 | 9.640 | 10.040 | 9.382 |
| 140710 | SOGA1 | 1.380 | 0.009 | 0.009 | up-reg. | 9.379 | 9.113 | 9.452 | 7.587 | 8.204 | 8.013 |
| 140735 | DYNLL2 | 1.205 | 0.031 | 0.015 | up-reg. | 6.900 | 6.528 | 6.876 | 4.942 | 6.011 | 5.735 |
| 140893 | RBBP8NL | 1.201 | 0.022 | 0.016 | up-reg. | 11.347 | 10.654 | 11.049 | 9.756 | 10.210 | 9.480 |
| 143425 | SYT9 | 1.004 | 0.023 | 0.030 | up-reg. | 12.584 | 11.839 | 12.163 | 11.022 | 11.416 | 11.136 |
| 143678 | C11orf94 | 0.907 | 0.038 | 0.044 | up-reg. | 6.299 | 6.247 | 6.005 | 5.418 | 4.759 | 5.654 |
| 143872 | ARHGAP42 | 0.890 | 0.017 | 0.047 | up-reg. | 5.142 | 4.779 | 4.547 | 3.790 | 4.000 | 4.010 |
| 144165 | PRICKLE1 | 0.971 | 0.003 | 0.034 | up-reg. | 9.097 | 8.864 | 9.014 | 8.010 | 8.067 | 7.984 |
| 144406 | CFAP251 | 1.066 | 0.017 | 0.024 | up-reg. | 5.159 | 5.451 | 4.864 | 3.804 | 4.324 | 4.147 |
| 144481 | SOCS2-AS1 | 1.076 | 0.015 | 0.024 | up-reg. | 8.157 | 7.920 | 8.260 | 6.688 | 7.085 | 7.335 |
| 144501 | KRT80 | 1.124 | 0.013 | 0.020 | up-reg. | 7.346 | 6.769 | 7.359 | 5.951 | 6.198 | 5.952 |
| 144699 | FBXL14 | 0.700 | 0.031 | 0.098 | up-reg. | 4.568 | 3.948 | 4.146 | 3.651 | 3.514 | 3.397 |
| 145474 | 0 | 1.678 | 0.043 | 0.004 | up-reg. | 6.061 | 5.038 | 4.676 | 3.425 | 3.052 | 4.264 |
| 145645 | TERB2 | 1.116 | 0.013 | 0.021 | up-reg. | 10.026 | 9.838 | 10.103 | 8.548 | 9.201 | 8.869 |
| 146691 | TOM1L2 | 1.112 | 0.016 | 0.021 | up-reg. | 9.594 | 9.300 | 9.655 | 8.077 | 8.778 | 8.356 |
| 146956 | EME1 | 0.826 | 0.010 | 0.061 | up-reg. | 5.592 | 5.276 | 5.443 | 4.596 | 4.792 | 4.446 |
| 147920 | IGFL2 | 1.677 | 0.014 | 0.004 | up-reg. | 6.651 | 5.910 | 6.361 | 4.951 | 4.142 | 4.798 |
| 148014 | TTC9B | 1.407 | 0.031 | 0.009 | up-reg. | 5.934 | 6.098 | 6.177 | 4.171 | 4.381 | 5.436 |
| 148203 | ZNF738 | 0.748 | 0.027 | 0.081 | up-reg. | 6.477 | 6.467 | 6.908 | 6.095 | 5.630 | 5.881 |
| 148281 | SYT6 | 0.785 | 0.037 | 0.071 | up-reg. | 4.900 | 4.611 | 4.575 | 4.002 | 3.496 | 4.231 |
| 148930 | KNCN | 1.560 | 0.019 | 0.006 | up-reg. | 7.592 | 7.391 | 7.700 | 5.360 | 6.124 | 6.517 |
| 149371 | EXOC8 | 0.713 | 0.008 | 0.093 | up-reg. | 8.668 | 8.673 | 8.434 | 7.978 | 7.774 | 7.883 |
| 149420 | PDIK1L | 1.231 | 0.009 | 0.014 | up-reg. | 13.257 | 13.113 | 13.257 | 11.622 | 12.217 | 12.097 |
| 149685 | ADIG | 1.688 | 0.016 | 0.004 | up-reg. | 8.927 | 8.299 | 8.962 | 6.563 | 7.041 | 7.519 |
| 150159 | SLC9B1 | 0.735 | 0.005 | 0.086 | up-reg. | 5.666 | 5.576 | 5.698 | 4.775 | 5.015 | 4.946 |
| 150165 | XKR3 | 1.678 | 0.043 | 0.004 | up-reg. | 14.825 | 14.636 | 14.941 | 12.248 | 13.004 | 14.116 |
| 150384 | GTSE1-DT | 1.293 | 0.032 | 0.012 | up-reg. | 6.610 | 6.210 | 5.978 | 4.600 | 4.700 | 5.619 |
| 150726 | FBXO41 | 1.167 | 0.005 | 0.017 | up-reg. | 7.699 | 7.648 | 7.507 | 6.210 | 6.591 | 6.552 |
| 151449 | GDF7 | 1.150 | 0.003 | 0.018 | up-reg. | 5.975 | 5.729 | 5.849 | 4.608 | 4.802 | 4.692 |
| 151903 | CCDC12 | 1.091 | 0.010 | 0.022 | up-reg. | 9.923 | 9.730 | 10.189 | 8.636 | 8.962 | 8.970 |
| 152098 | ZCWPW2 | 1.400 | 0.019 | 0.009 | up-reg. | 7.176 | 6.752 | 6.692 | 4.977 | 5.513 | 5.931 |
| 152573 | SHISA3 | 2.040 | 0.003 | 0.002 | up-reg. | 5.327 | 5.303 | 5.463 | 3.107 | 3.271 | 3.594 |
| 152926 | PPM1K | 0.806 | 0.023 | 0.065 | up-reg. | 6.028 | 6.288 | 5.836 | 5.547 | 5.083 | 5.103 |
| 153020 | RASGEF1B | 2.022 | 0.009 | 0.002 | up-reg. | 5.720 | 4.778 | 5.097 | 3.407 | 3.169 | 2.953 |
| 153643 | FAM81B | 0.941 | 0.027 | 0.039 | up-reg. | 6.005 | 5.430 | 5.664 | 4.850 | 4.401 | 5.026 |
| 153768 | PRELID2 | 1.449 | 0.017 | 0.008 | up-reg. | 7.437 | 6.564 | 7.115 | 5.262 | 5.696 | 5.810 |
| 154091 | SLC2A12 | 1.006 | 0.029 | 0.030 | up-reg. | 6.145 | 5.722 | 5.589 | 4.663 | 4.526 | 5.248 |
| 155185 | AMZ1 | 2.773 | 0.002 | 0.001 | up-reg. | 7.612 | 7.791 | 7.563 | 4.995 | 4.573 | 5.079 |
| 155370 | 0 | 1.575 | 0.033 | 0.006 | up-reg. | 6.059 | 6.200 | 5.250 | 4.419 | 3.586 | 4.779 |
| 158035 | LINC00032 | 1.045 | 0.029 | 0.026 | up-reg. | 5.715 | 4.827 | 4.989 | 4.157 | 3.956 | 4.283 |
| 161145 | TMEM229B | 1.276 | 0.007 | 0.012 | up-reg. | 7.671 | 7.753 | 7.725 | 6.273 | 6.285 | 6.762 |
| 161882 | ZFPM1 | 1.082 | 0.016 | 0.023 | up-reg. | 5.896 | 5.658 | 5.385 | 4.338 | 4.474 | 4.880 |
| 163933 | FAM43B | 3.396 | 0.003 | 0.000 | up-reg. | 7.230 | 7.193 | 6.881 | 3.954 | 3.246 | 3.916 |
| 164153 | UBL4B | 3.099 | 0.001 | 0.000 | up-reg. | 10.117 | 10.388 | 9.997 | 7.168 | 6.986 | 7.049 |
| 164633 | CABP7 | 0.879 | 0.040 | 0.049 | up-reg. | 12.493 | 12.453 | 12.825 | 11.238 | 12.106 | 11.791 |
| 165186 | TOGARAM2 | 1.238 | 0.042 | 0.014 | up-reg. | 9.869 | 9.609 | 10.232 | 7.977 | 9.179 | 8.841 |
| 165257 | C1QL2 | 1.502 | 0.027 | 0.007 | up-reg. | 7.911 | 7.967 | 7.971 | 5.641 | 6.793 | 6.908 |
| 166815 | TIGD2 | 0.839 | 0.027 | 0.057 | up-reg. | 3.617 | 3.581 | 3.726 | 2.809 | 2.417 | 3.180 |
| 166968 | MIER3 | 0.756 | 0.042 | 0.079 | up-reg. | 3.958 | 3.306 | 4.035 | 2.865 | 3.127 | 3.039 |
| 171177 | RHOV | 2.896 | 0.003 | 0.001 | up-reg. | 6.879 | 6.781 | 6.530 | 4.214 | 3.570 | 3.717 |
| 192668 | CYS1 | 2.027 | 0.002 | 0.002 | up-reg. | 7.689 | 7.780 | 7.460 | 5.570 | 5.486 | 5.792 |
| 196074 | METTL15 | 1.042 | 0.006 | 0.027 | up-reg. | 7.795 | 7.664 | 7.508 | 6.560 | 6.798 | 6.483 |
| 196374 | KRT78 | 1.032 | 0.013 | 0.027 | up-reg. | 13.902 | 13.684 | 13.593 | 12.406 | 12.997 | 12.680 |
| 196996 | GRAMD2A | 1.341 | 0.009 | 0.010 | up-reg. | 7.056 | 6.784 | 7.166 | 5.367 | 5.658 | 5.957 |
| 199857 | ALG14 | 0.885 | 0.032 | 0.048 | up-reg. | 6.605 | 6.495 | 6.481 | 5.519 | 5.284 | 6.124 |
| 200035 | NUDT17 | 1.316 | 0.007 | 0.011 | up-reg. | 6.543 | 5.961 | 6.223 | 4.983 | 4.803 | 4.993 |
| 200197 | TMEM51-AS1 | 1.286 | 0.033 | 0.012 | up-reg. | 9.977 | 9.469 | 10.089 | 7.915 | 8.927 | 8.834 |
| 200845 | KCTD6 | 1.225 | 0.006 | 0.014 | up-reg. | 7.071 | 6.712 | 6.938 | 5.813 | 5.458 | 5.774 |
| 200895 | DHFR2 | 1.046 | 0.034 | 0.026 | up-reg. | 6.106 | 6.078 | 5.284 | 4.678 | 4.581 | 5.070 |
| 201617 | LINC00870 | 1.318 | 0.009 | 0.011 | up-reg. | 9.274 | 8.967 | 9.148 | 7.587 | 8.167 | 7.682 |
| 203260 | CCDC107 | 0.943 | 0.003 | 0.038 | up-reg. | 13.201 | 13.421 | 13.322 | 12.453 | 12.314 | 12.348 |
| 203414 | 0 | 1.441 | 0.013 | 0.008 | up-reg. | 6.066 | 5.662 | 6.053 | 4.115 | 4.415 | 4.926 |
| 219285 | SAMD9L | 0.778 | 0.012 | 0.073 | up-reg. | 5.643 | 5.356 | 5.737 | 4.730 | 4.951 | 4.722 |
| 219333 | USP12 | 1.282 | 0.009 | 0.012 | up-reg. | 5.964 | 6.078 | 5.470 | 4.546 | 4.478 | 4.642 |
| 219438 | OR5D18 | 0.962 | 0.024 | 0.036 | up-reg. | 4.925 | 4.958 | 5.597 | 4.023 | 4.395 | 4.177 |
| 220004 | PPP1R32 | 0.953 | 0.017 | 0.037 | up-reg. | 8.081 | 7.878 | 7.827 | 7.019 | 7.277 | 6.630 |
| 220441 | RNF152 | 0.994 | 0.008 | 0.032 | up-reg. | 7.858 | 8.067 | 7.699 | 7.032 | 6.690 | 6.921 |
| 221468 | AL353579.1 | 3.445 | 0.001 | 0.000 | up-reg. | 7.866 | 7.859 | 7.753 | 4.449 | 4.059 | 4.635 |
| 221496 | LEMD2 | 1.677 | 0.024 | 0.004 | up-reg. | 11.874 | 11.622 | 12.042 | 9.364 | 10.460 | 10.682 |
| 221687 | RNF182 | 0.993 | 0.029 | 0.032 | up-reg. | 6.057 | 5.304 | 5.831 | 4.746 | 4.462 | 5.005 |
| 221710 | SMIM13 | 1.037 | 0.011 | 0.027 | up-reg. | 8.276 | 8.592 | 8.134 | 7.509 | 7.096 | 7.287 |
| 221833 | SP8 | 1.379 | 0.011 | 0.009 | up-reg. | 9.367 | 9.130 | 9.289 | 7.929 | 7.479 | 8.242 |
| 222068 | TMED4 | 1.191 | 0.012 | 0.016 | up-reg. | 8.444 | 8.754 | 8.315 | 7.439 | 6.978 | 7.522 |
| 222171 | PRR15 | 3.086 | 0.001 | 0.000 | up-reg. | 6.012 | 6.067 | 5.946 | 2.876 | 2.688 | 3.203 |
| 222236 | NAPEPLD | 0.943 | 0.023 | 0.038 | up-reg. | 5.589 | 5.810 | 5.071 | 4.547 | 4.690 | 4.404 |
| 246754 | 0 | 0.905 | 0.036 | 0.045 | up-reg. | 7.232 | 7.256 | 7.165 | 5.771 | 6.592 | 6.575 |
| 252954 | TTTY22 | 1.905 | 0.007 | 0.003 | up-reg. | 7.375 | 6.774 | 7.048 | 5.176 | 4.844 | 5.463 |
| 253430 | IPMK | 0.776 | 0.008 | 0.073 | up-reg. | 6.867 | 6.858 | 6.899 | 6.050 | 6.306 | 5.941 |
| 253962 | CACNA1G-AS1 | 0.736 | 0.049 | 0.085 | up-reg. | 4.183 | 4.066 | 3.759 | 3.326 | 2.853 | 3.620 |
| 254099 | LINC01342 | 1.130 | 0.027 | 0.019 | up-reg. | 6.011 | 5.746 | 5.752 | 4.371 | 4.463 | 5.286 |
| 254295 | PHYHD1 | 1.077 | 0.046 | 0.024 | up-reg. | 11.463 | 11.175 | 11.871 | 10.081 | 11.032 | 10.167 |
| 255027 | AC140504.1 | 1.459 | 0.022 | 0.008 | up-reg. | 4.802 | 3.612 | 4.014 | 2.589 | 2.761 | 2.702 |
| 255631 | COL24A1 | 0.876 | 0.039 | 0.050 | up-reg. | 9.911 | 9.897 | 10.051 | 8.574 | 9.507 | 9.151 |
| 283089 | WDR11-AS1 | 1.086 | 0.024 | 0.023 | up-reg. | 5.413 | 4.738 | 5.213 | 4.374 | 4.005 | 3.726 |
| 283143 | 0 | 0.897 | 0.011 | 0.046 | up-reg. | 12.159 | 11.900 | 11.683 | 10.899 | 11.152 | 10.999 |
| 283575 | LINC02288 | 1.096 | 0.013 | 0.022 | up-reg. | 7.249 | 6.916 | 7.233 | 5.744 | 6.344 | 6.020 |
| 283687 | 0 | 0.943 | 0.019 | 0.038 | up-reg. | 6.573 | 6.605 | 6.112 | 5.456 | 5.266 | 5.739 |
| 283692 | CPEB1-AS1 | 1.958 | 0.017 | 0.002 | up-reg. | 13.978 | 13.802 | 13.978 | 11.581 | 11.529 | 12.774 |
| 283982 | 0 | 1.019 | 0.005 | 0.029 | up-reg. | 8.552 | 8.345 | 8.445 | 7.241 | 7.495 | 7.549 |
| 284029 | LINC00324 | 1.490 | 0.008 | 0.007 | up-reg. | 8.312 | 8.008 | 8.594 | 6.574 | 6.941 | 6.928 |
| 284124 | FLJ36000 | 1.237 | 0.012 | 0.014 | up-reg. | 13.528 | 13.270 | 12.897 | 11.777 | 12.203 | 12.002 |
| 284161 | GDPD1 | 1.204 | 0.033 | 0.015 | up-reg. | 3.802 | 3.303 | 4.497 | 2.679 | 2.725 | 2.586 |
| 284266 | SIGLEC15 | 0.780 | 0.006 | 0.072 | up-reg. | 6.779 | 6.741 | 6.831 | 5.867 | 5.962 | 6.182 |
| 284276 | LINC00683 | 1.098 | 0.012 | 0.022 | up-reg. | 7.743 | 7.904 | 7.473 | 6.470 | 6.903 | 6.453 |
| 284307 | ZIK1 | 1.082 | 0.007 | 0.023 | up-reg. | 5.541 | 5.498 | 5.741 | 4.667 | 4.279 | 4.588 |
| 284325 | C19orf54 | 1.362 | 0.014 | 0.010 | up-reg. | 10.913 | 10.696 | 11.242 | 9.374 | 9.999 | 9.393 |
| 284346 | ZNF575 | 1.316 | 0.031 | 0.011 | up-reg. | 10.987 | 10.481 | 10.999 | 8.891 | 10.018 | 9.609 |
| 284358 | MAMSTR | 0.979 | 0.017 | 0.033 | up-reg. | 12.002 | 11.810 | 12.159 | 10.654 | 11.204 | 11.175 |
| 284412 | 0 | 1.118 | 0.009 | 0.020 | up-reg. | 6.858 | 6.626 | 6.748 | 5.393 | 5.573 | 5.911 |
| 284424 | MIR7-3HG | 1.301 | 0.007 | 0.012 | up-reg. | 12.172 | 11.965 | 11.766 | 10.433 | 10.834 | 10.735 |
| 284565 | NBPF15 | 1.076 | 0.026 | 0.024 | up-reg. | 6.717 | 6.771 | 7.279 | 5.505 | 5.787 | 6.247 |
| 284612 | SYPL2 | 1.118 | 0.007 | 0.020 | up-reg. | 9.148 | 9.388 | 9.010 | 8.207 | 7.875 | 8.111 |
| 284649 | 0 | 0.840 | 0.035 | 0.057 | up-reg. | 9.632 | 9.618 | 9.826 | 8.412 | 9.248 | 8.897 |
| 284656 | EPHA10 | 0.887 | 0.011 | 0.048 | up-reg. | 5.044 | 5.315 | 5.146 | 4.391 | 4.430 | 4.022 |
| 284757 | MIR646HG | 1.028 | 0.047 | 0.028 | up-reg. | 5.640 | 4.629 | 5.122 | 3.783 | 4.078 | 4.448 |
| 284835 | LINC00323 | 2.071 | 0.012 | 0.002 | up-reg. | 9.626 | 9.108 | 9.811 | 6.859 | 7.826 | 7.648 |
| 285343 | TCAIM | 1.199 | 0.039 | 0.016 | up-reg. | 5.125 | 4.292 | 4.453 | 3.890 | 2.941 | 3.443 |
| 285601 | GPR150 | 0.812 | 0.044 | 0.064 | up-reg. | 5.386 | 4.641 | 5.126 | 4.121 | 4.055 | 4.544 |
| 285622 | 0 | 1.520 | 0.018 | 0.006 | up-reg. | 4.936 | 4.500 | 4.475 | 2.938 | 2.721 | 3.690 |
| 285641 | SLC36A3 | 1.030 | 0.012 | 0.028 | up-reg. | 8.895 | 8.869 | 8.923 | 7.524 | 8.164 | 7.909 |
| 285643 | KIF4B | 1.822 | 0.044 | 0.003 | up-reg. | 8.067 | 7.750 | 8.157 | 5.023 | 6.488 | 6.996 |
| 285762 | 0 | 0.900 | 0.009 | 0.045 | up-reg. | 6.115 | 5.733 | 5.700 | 4.885 | 4.949 | 5.013 |
| 285848 | PNPLA1 | 0.872 | 0.028 | 0.051 | up-reg. | 6.952 | 6.864 | 6.659 | 5.796 | 5.676 | 6.388 |
| 285877 | POM121L12 | 1.014 | 0.046 | 0.029 | up-reg. | 6.563 | 6.179 | 6.642 | 4.838 | 5.630 | 5.873 |
| 286023 | FLJ40288 | 2.059 | 0.030 | 0.002 | up-reg. | 6.535 | 5.836 | 6.583 | 3.216 | 4.793 | 4.768 |
| 286183 | NKAIN3 | 1.098 | 0.038 | 0.022 | up-reg. | 5.049 | 4.851 | 4.148 | 3.440 | 3.324 | 3.989 |
| 286554 | BCORP1 | 1.398 | 0.020 | 0.009 | up-reg. | 8.517 | 8.249 | 8.561 | 6.994 | 6.543 | 7.596 |
| 327657 | SERPINA9 | 1.274 | 0.043 | 0.012 | up-reg. | 7.692 | 7.472 | 8.092 | 5.740 | 6.934 | 6.761 |
| 333926 | PPM1J | 0.916 | 0.050 | 0.043 | up-reg. | 4.862 | 5.064 | 5.766 | 4.397 | 4.554 | 3.992 |
| 338321 | NLRP9 | 1.246 | 0.008 | 0.013 | up-reg. | 12.134 | 12.002 | 12.182 | 10.552 | 11.133 | 10.895 |
| 338817 | LINC01252 | 1.087 | 0.027 | 0.023 | up-reg. | 9.563 | 9.270 | 9.988 | 8.428 | 8.907 | 8.224 |
| 338949 | TMEM202 | 2.091 | 0.005 | 0.002 | up-reg. | 5.581 | 5.237 | 5.080 | 2.961 | 3.086 | 3.579 |
| 339524 | LINC01140 | 0.982 | 0.014 | 0.033 | up-reg. | 6.609 | 6.420 | 6.600 | 5.725 | 5.757 | 5.202 |
| 339665 | SLC35E4 | 1.809 | 0.013 | 0.003 | up-reg. | 7.049 | 6.410 | 7.046 | 4.890 | 4.660 | 5.526 |
| 339807 | LINC02579 | 1.300 | 0.013 | 0.012 | up-reg. | 7.571 | 7.013 | 7.478 | 5.842 | 5.921 | 6.397 |
| 340094 | 0 | 1.325 | 0.023 | 0.011 | up-reg. | 8.221 | 7.830 | 8.330 | 6.248 | 6.941 | 7.217 |
| 341359 | SYT10 | 1.679 | 0.026 | 0.004 | up-reg. | 6.604 | 6.237 | 6.125 | 4.618 | 3.929 | 5.381 |
| 343521 | TCTEX1D4 | 0.809 | 0.044 | 0.065 | up-reg. | 4.209 | 4.662 | 3.940 | 3.601 | 3.131 | 3.651 |
| 345611 | IRGM | 1.078 | 0.018 | 0.024 | up-reg. | 5.480 | 5.649 | 5.744 | 4.223 | 4.452 | 4.964 |
| 345895 | RSPH4A | 0.871 | 0.027 | 0.051 | up-reg. | 5.676 | 5.183 | 5.967 | 4.814 | 4.678 | 4.721 |
| 347735 | SERINC2 | 0.826 | 0.003 | 0.060 | up-reg. | 14.469 | 14.618 | 14.504 | 13.619 | 13.711 | 13.782 |
| 349152 | DPY19L2P2 | 1.149 | 0.014 | 0.018 | up-reg. | 4.463 | 4.230 | 4.584 | 2.915 | 3.317 | 3.598 |
| 349408 | 0 | 0.907 | 0.020 | 0.044 | up-reg. | 5.217 | 5.266 | 5.027 | 3.963 | 4.635 | 4.190 |
| 353139 | LCE2A | 5.275 | 0.000 | 0.000 | up-reg. | 10.544 | 10.430 | 10.600 | 4.972 | 5.233 | 5.542 |
| 353189 | SLCO4C1 | 1.807 | 0.011 | 0.003 | up-reg. | 5.457 | 4.687 | 5.023 | 3.673 | 3.037 | 3.037 |
| 360030 | NANOGNB | 1.517 | 0.020 | 0.006 | up-reg. | 7.617 | 7.222 | 7.660 | 5.353 | 6.355 | 6.241 |
| 360200 | TMPRSS9 | 1.334 | 0.029 | 0.011 | up-reg. | 10.051 | 9.168 | 10.040 | 8.084 | 8.838 | 8.334 |
| 373509 | USP50 | 1.732 | 0.008 | 0.004 | up-reg. | 8.242 | 7.667 | 7.734 | 5.805 | 6.334 | 6.309 |
| 374395 | TMEM179B | 1.278 | 0.003 | 0.012 | up-reg. | 10.529 | 10.650 | 10.364 | 9.330 | 9.168 | 9.212 |
| 375057 | STUM | 1.587 | 0.009 | 0.005 | up-reg. | 6.561 | 6.140 | 6.345 | 4.491 | 4.608 | 5.186 |
| 375295 | 0 | 1.756 | 0.005 | 0.004 | up-reg. | 7.445 | 7.302 | 7.318 | 5.646 | 5.254 | 5.896 |
| 375612 | LHFPL3 | 2.269 | 0.017 | 0.001 | up-reg. | 6.407 | 5.613 | 5.738 | 3.255 | 4.467 | 3.228 |
| 375790 | AGRN | 1.507 | 0.001 | 0.007 | up-reg. | 12.453 | 12.580 | 12.378 | 11.050 | 10.918 | 10.921 |
| 375791 | CYSRT1 | 1.233 | 0.002 | 0.014 | up-reg. | 8.625 | 8.394 | 8.424 | 7.232 | 7.301 | 7.213 |
| 378805 | LINC-PINT | 0.726 | 0.021 | 0.089 | up-reg. | 5.825 | 5.916 | 6.347 | 5.246 | 5.406 | 5.258 |
| 387273 | KRTAP5-10 | 1.279 | 0.022 | 0.012 | up-reg. | 6.349 | 6.083 | 6.201 | 4.666 | 4.596 | 5.534 |
| 387521 | PEDS1 | 1.015 | 0.019 | 0.029 | up-reg. | 8.573 | 8.217 | 8.719 | 7.255 | 7.827 | 7.384 |
| 387640 | SKIDA1 | 0.825 | 0.030 | 0.061 | up-reg. | 4.733 | 5.194 | 4.891 | 3.911 | 3.942 | 4.489 |
| 387694 | SH2D4B | 1.151 | 0.012 | 0.018 | up-reg. | 7.775 | 7.249 | 7.698 | 6.198 | 6.479 | 6.592 |
| 387775 | SLC22A10 | 1.680 | 0.028 | 0.004 | up-reg. | 9.028 | 8.830 | 9.200 | 6.480 | 7.952 | 7.587 |
| 387882 | C12orf75 | 1.241 | 0.016 | 0.014 | up-reg. | 5.649 | 5.399 | 5.283 | 4.631 | 3.820 | 4.158 |
| 387895 | 0 | 1.157 | 0.013 | 0.018 | up-reg. | 6.033 | 5.766 | 5.792 | 4.321 | 4.808 | 4.990 |
| 387990 | TOMM20L | 0.754 | 0.015 | 0.079 | up-reg. | 15.233 | 15.043 | 15.465 | 14.337 | 14.636 | 14.504 |
| 388555 | IGFL3 | 1.378 | 0.017 | 0.010 | up-reg. | 6.794 | 6.548 | 6.902 | 5.877 | 4.925 | 5.307 |
| 388722 | C1orf53 | 1.332 | 0.009 | 0.011 | up-reg. | 5.919 | 5.710 | 5.645 | 4.221 | 4.263 | 4.794 |
| 388796 | SNHG17 | 0.985 | 0.026 | 0.033 | up-reg. | 5.855 | 5.838 | 6.289 | 5.428 | 4.853 | 4.746 |
| 388965 | 0 | 0.899 | 0.012 | 0.046 | up-reg. | 5.858 | 5.913 | 6.234 | 4.901 | 5.268 | 5.140 |
| 389084 | SNORC | 1.205 | 0.031 | 0.015 | up-reg. | 6.356 | 5.627 | 6.054 | 4.894 | 4.307 | 5.222 |
| 389124 | IQCF5 | 1.055 | 0.006 | 0.025 | up-reg. | 5.286 | 5.141 | 5.238 | 4.055 | 4.041 | 4.405 |
| 389816 | LRRC26 | 0.960 | 0.029 | 0.036 | up-reg. | 10.079 | 9.517 | 10.198 | 8.671 | 9.212 | 9.031 |
| 390003 | 0 | 0.832 | 0.025 | 0.059 | up-reg. | 5.487 | 5.298 | 5.326 | 4.134 | 4.699 | 4.782 |
| 390058 | OR51B6 | 1.191 | 0.005 | 0.016 | up-reg. | 5.665 | 5.758 | 5.831 | 4.812 | 4.483 | 4.385 |
| 391712 | TRIM61 | 1.136 | 0.020 | 0.019 | up-reg. | 6.564 | 6.589 | 6.371 | 5.121 | 5.118 | 5.877 |
| 392459 | CXXC1P1 | 0.952 | 0.012 | 0.037 | up-reg. | 6.707 | 6.414 | 6.597 | 5.398 | 5.560 | 5.903 |
| 392509 | ARL13A | 1.375 | 0.010 | 0.010 | up-reg. | 6.375 | 6.238 | 6.362 | 4.538 | 5.295 | 5.017 |
| 400046 | 0 | 0.719 | 0.039 | 0.091 | up-reg. | 3.879 | 4.032 | 4.357 | 3.707 | 3.291 | 3.113 |
| 400618 | SOX9-AS1 | 1.550 | 0.006 | 0.006 | up-reg. | 10.147 | 9.939 | 10.447 | 8.390 | 8.744 | 8.750 |
| 400627 | AC127496.1 | 1.066 | 0.009 | 0.024 | up-reg. | 5.229 | 5.110 | 4.835 | 3.776 | 4.127 | 4.072 |
| 400866 | LINC00114 | 0.790 | 0.023 | 0.070 | up-reg. | 4.848 | 4.825 | 5.206 | 4.088 | 3.955 | 4.466 |
| 400935 | IL17REL | 1.067 | 0.013 | 0.024 | up-reg. | 12.227 | 12.046 | 12.527 | 10.921 | 11.305 | 11.373 |
| 400946 | LINC00954 | 1.177 | 0.012 | 0.017 | up-reg. | 6.768 | 6.278 | 6.712 | 5.222 | 5.307 | 5.698 |
| 401021 | 0 | 1.121 | 0.004 | 0.020 | up-reg. | 6.553 | 6.349 | 6.568 | 5.228 | 5.336 | 5.543 |
| 401089 | FOXL2NB | 0.915 | 0.021 | 0.043 | up-reg. | 8.117 | 7.997 | 7.909 | 6.679 | 7.309 | 7.289 |
| 401207 | C5orf63 | 1.448 | 0.026 | 0.008 | up-reg. | 5.774 | 4.928 | 5.172 | 3.599 | 3.516 | 4.414 |
| 401288 | LINC00242 | 2.299 | 0.030 | 0.001 | up-reg. | 8.596 | 8.029 | 8.625 | 4.903 | 6.784 | 6.666 |
| 401474 | SAMD12 | 1.346 | 0.006 | 0.010 | up-reg. | 8.365 | 8.492 | 8.279 | 7.034 | 6.755 | 7.308 |
| 401494 | HACD4 | 1.113 | 0.007 | 0.021 | up-reg. | 6.058 | 5.934 | 5.651 | 4.700 | 4.930 | 4.675 |
| 401551 | WDR38 | 0.700 | 0.029 | 0.099 | up-reg. | 8.246 | 8.145 | 8.233 | 7.222 | 7.872 | 7.431 |
| 439950 | 0 | 0.932 | 0.042 | 0.040 | up-reg. | 5.059 | 5.182 | 5.619 | 3.859 | 4.595 | 4.611 |
| 440498 | HSBP1L1 | 1.595 | 0.007 | 0.005 | up-reg. | 7.238 | 7.009 | 6.810 | 5.310 | 5.184 | 5.778 |
| 440956 | IQCF6 | 1.867 | 0.012 | 0.003 | up-reg. | 7.883 | 7.280 | 7.882 | 5.285 | 6.055 | 6.106 |
| 440957 | SMIM4 | 0.910 | 0.014 | 0.044 | up-reg. | 10.207 | 10.570 | 10.065 | 9.495 | 9.422 | 9.195 |
| 441177 | LINC00602 | 1.377 | 0.034 | 0.010 | up-reg. | 5.995 | 5.366 | 5.672 | 4.192 | 3.732 | 4.977 |
| 441432 | 0 | 1.414 | 0.013 | 0.009 | up-reg. | 8.756 | 8.284 | 8.864 | 6.848 | 7.463 | 7.352 |
| 448835 | LCE6A | 1.686 | 0.011 | 0.004 | up-reg. | 9.472 | 9.342 | 9.618 | 7.227 | 8.077 | 8.070 |
| 493911 | PHOSPHO2 | 0.997 | 0.006 | 0.031 | up-reg. | 7.833 | 7.911 | 7.613 | 6.839 | 6.638 | 6.887 |
| 497258 | BDNF-AS | 0.804 | 0.009 | 0.066 | up-reg. | 6.905 | 6.821 | 6.724 | 5.800 | 6.080 | 6.157 |
| 550643 | NBDY | 1.513 | 0.014 | 0.007 | up-reg. | 5.650 | 5.218 | 5.243 | 3.923 | 3.386 | 4.263 |
| 613209 | DEFB135 | 0.755 | 0.040 | 0.079 | up-reg. | 4.510 | 4.909 | 4.759 | 4.352 | 3.634 | 3.928 |
| 619351 | LINC00589 | 0.839 | 0.026 | 0.057 | up-reg. | 4.779 | 4.600 | 4.212 | 3.810 | 3.407 | 3.858 |
| 641364 | SLC7A11-AS1 | 1.087 | 0.013 | 0.023 | up-reg. | 5.356 | 5.066 | 5.317 | 3.927 | 4.033 | 4.520 |
| 643338 | C15orf62 | 1.232 | 0.019 | 0.014 | up-reg. | 4.377 | 3.723 | 4.286 | 3.246 | 2.824 | 2.620 |
| 643650 | LINC00842 | 1.894 | 0.026 | 0.003 | up-reg. | 7.973 | 7.418 | 7.954 | 5.001 | 6.119 | 6.543 |
| 644150 | WIPF3 | 0.919 | 0.012 | 0.042 | up-reg. | 5.808 | 5.898 | 5.723 | 5.027 | 4.581 | 5.064 |
| 644215 | 0 | 1.471 | 0.027 | 0.007 | up-reg. | 6.970 | 6.447 | 6.991 | 4.651 | 5.543 | 5.803 |
| 644844 | PHGR1 | 1.392 | 0.013 | 0.009 | up-reg. | 5.625 | 5.110 | 5.087 | 3.651 | 3.750 | 4.245 |
| 645528 | FAM238A | 0.827 | 0.015 | 0.060 | up-reg. | 8.603 | 8.655 | 8.554 | 7.602 | 8.100 | 7.627 |
| 645638 | 0 | 1.762 | 0.003 | 0.004 | up-reg. | 8.651 | 8.484 | 8.440 | 6.502 | 6.810 | 6.977 |
| 645676 | ASH1L-AS1 | 0.881 | 0.012 | 0.049 | up-reg. | 7.289 | 6.878 | 7.332 | 6.372 | 6.169 | 6.315 |
| 646982 | LINC00598 | 1.323 | 0.010 | 0.011 | up-reg. | 11.350 | 11.142 | 11.631 | 9.771 | 10.069 | 10.314 |
| 650655 | ABCA17P | 0.908 | 0.042 | 0.044 | up-reg. | 5.859 | 5.671 | 5.975 | 4.454 | 4.902 | 5.427 |
| 652276 | AC141586.1 | 0.763 | 0.042 | 0.077 | up-reg. | 5.126 | 5.009 | 5.532 | 4.196 | 4.828 | 4.354 |
| 652968 | CASTOR1 | 1.534 | 0.023 | 0.006 | up-reg. | 6.927 | 6.150 | 6.908 | 4.627 | 5.179 | 5.577 |
| 653149 | NBPF6 | 0.890 | 0.011 | 0.047 | up-reg. | 6.273 | 5.777 | 6.170 | 5.168 | 5.145 | 5.237 |
| 654816 | NCF1B | 0.803 | 0.043 | 0.066 | up-reg. | 6.268 | 6.172 | 6.660 | 5.140 | 5.715 | 5.836 |
| 677681 | SCARNA20 | 0.974 | 0.047 | 0.034 | up-reg. | 5.025 | 4.793 | 4.369 | 4.291 | 3.419 | 3.556 |
| 677769 | SNHG22 | 2.034 | 0.004 | 0.002 | up-reg. | 6.985 | 6.886 | 6.598 | 5.000 | 4.525 | 4.841 |
| 677780 | SCARNA11 | 2.135 | 0.009 | 0.002 | up-reg. | 5.981 | 6.170 | 5.984 | 4.470 | 3.901 | 3.359 |
| 728012 | MCHR2-AS1 | 0.756 | 0.048 | 0.079 | up-reg. | 7.221 | 7.220 | 7.392 | 6.017 | 6.729 | 6.819 |
| 728175 | 0 | 1.710 | 0.006 | 0.004 | up-reg. | 5.985 | 5.649 | 5.833 | 3.878 | 3.977 | 4.482 |
| 728655 | 0 | 1.077 | 0.008 | 0.024 | up-reg. | 5.424 | 4.932 | 5.207 | 4.133 | 3.985 | 4.213 |
| 729082 | OIP5-AS1 | 0.805 | 0.005 | 0.066 | up-reg. | 9.487 | 9.503 | 9.311 | 8.690 | 8.494 | 8.703 |
| 729224 | 0 | 1.355 | 0.036 | 0.010 | up-reg. | 5.572 | 5.366 | 5.246 | 3.267 | 4.250 | 4.602 |
| 729291 | 0 | 1.649 | 0.004 | 0.005 | up-reg. | 9.714 | 9.445 | 9.871 | 7.809 | 8.186 | 8.089 |
| 729475 | RAD51AP2 | 0.943 | 0.023 | 0.038 | up-reg. | 5.681 | 5.272 | 5.129 | 4.294 | 4.220 | 4.738 |
| 729991 | BORCS8 | 1.107 | 0.016 | 0.021 | up-reg. | 10.552 | 9.946 | 10.639 | 9.269 | 9.382 | 9.164 |
| 730091 | LINC00886 | 3.102 | 0.003 | 0.000 | up-reg. | 6.676 | 6.135 | 5.977 | 3.357 | 2.944 | 3.182 |
| 100124533 | 0 | 0.856 | 0.043 | 0.054 | up-reg. | 3.312 | 3.889 | 3.877 | 2.616 | 3.247 | 2.646 |
| 100128164 | AC008040.1 | 1.304 | 0.017 | 0.011 | up-reg. | 7.850 | 7.512 | 7.031 | 5.879 | 6.248 | 6.353 |
| 100128252 | 0 | 1.140 | 0.025 | 0.019 | up-reg. | 7.194 | 6.937 | 7.074 | 5.364 | 6.192 | 6.230 |
| 100128653 | 0 | 1.085 | 0.030 | 0.023 | up-reg. | 4.968 | 5.518 | 5.077 | 4.019 | 3.714 | 4.575 |
| 100130274 | CCDC166 | 1.109 | 0.020 | 0.021 | up-reg. | 7.337 | 6.850 | 7.109 | 6.016 | 5.612 | 6.340 |
| 100130557 | 0 | 0.992 | 0.042 | 0.032 | up-reg. | 8.486 | 9.298 | 8.394 | 7.474 | 7.772 | 7.957 |
| 100130581 | 0 | 0.979 | 0.029 | 0.033 | up-reg. | 13.243 | 13.172 | 13.505 | 11.871 | 12.721 | 12.391 |
| 100130717 | HDHD5-AS1 | 0.988 | 0.010 | 0.032 | up-reg. | 7.557 | 7.396 | 7.516 | 6.473 | 6.261 | 6.772 |
| 100131017 | ZNF316 | 0.854 | 0.016 | 0.054 | up-reg. | 5.076 | 4.745 | 5.264 | 4.310 | 3.996 | 4.217 |
| 100131378 | C11orf91 | 1.508 | 0.001 | 0.007 | up-reg. | 10.077 | 9.997 | 10.167 | 8.444 | 8.660 | 8.612 |
| 100132501 | 0 | 0.774 | 0.030 | 0.074 | up-reg. | 3.884 | 3.512 | 3.846 | 3.099 | 2.614 | 3.208 |
| 100132815 | 0 | 0.774 | 0.030 | 0.074 | up-reg. | 9.445 | 9.485 | 9.463 | 8.750 | 9.030 | 8.289 |
| 100132987 | LINC00595 | 1.835 | 0.014 | 0.003 | up-reg. | 8.950 | 8.227 | 8.887 | 6.335 | 6.967 | 7.256 |
| 100134713 | NDUFB2-AS1 | 0.965 | 0.037 | 0.035 | up-reg. | 5.795 | 5.704 | 5.200 | 4.922 | 4.152 | 4.728 |
| 100134869 | UBE2Q2P2 | 0.865 | 0.019 | 0.052 | up-reg. | 5.363 | 5.086 | 5.239 | 4.086 | 4.310 | 4.697 |
| 100169851 | PATE3 | 1.563 | 0.009 | 0.006 | up-reg. | 7.893 | 7.377 | 7.689 | 5.729 | 6.327 | 6.213 |
| 100170841 | EPOP | 1.427 | 0.015 | 0.008 | up-reg. | 7.522 | 7.524 | 7.138 | 5.901 | 5.564 | 6.441 |
| 100240735 | AC023794.3 | 1.553 | 0.042 | 0.006 | up-reg. | 5.836 | 6.973 | 5.607 | 4.151 | 4.524 | 5.082 |
| 100287049 | AL139011.1 | 1.879 | 0.010 | 0.003 | up-reg. | 5.630 | 5.958 | 5.554 | 4.194 | 3.275 | 4.036 |
| 100288842 | 0 | 1.365 | 0.021 | 0.010 | up-reg. | 5.415 | 4.710 | 4.919 | 3.617 | 3.243 | 4.088 |
| 100288911 | 0 | 1.138 | 0.004 | 0.019 | up-reg. | 9.072 | 9.031 | 8.927 | 8.060 | 7.687 | 7.868 |
| 100289017 | 0 | 0.896 | 0.044 | 0.046 | up-reg. | 4.681 | 4.680 | 4.347 | 3.174 | 3.733 | 4.114 |
| 100289187 | TMEM225B | 1.513 | 0.005 | 0.007 | up-reg. | 8.679 | 8.585 | 8.477 | 7.060 | 6.814 | 7.328 |
| 100289410 | 0 | 0.939 | 0.044 | 0.039 | up-reg. | 10.516 | 10.392 | 10.780 | 9.058 | 10.015 | 9.798 |
| 100302692 | FTX | 1.906 | 0.006 | 0.003 | up-reg. | 9.302 | 9.020 | 9.589 | 7.083 | 7.604 | 7.508 |
| 100379345 | 0 | 2.025 | 0.008 | 0.002 | up-reg. | 6.761 | 6.324 | 6.052 | 4.679 | 3.975 | 4.409 |
| 100463486 | MTRNR2L8 | 1.253 | 0.014 | 0.013 | up-reg. | 7.393 | 7.253 | 6.842 | 5.877 | 5.634 | 6.218 |
| 100505573 | INAFM2 | 1.127 | 0.005 | 0.020 | up-reg. | 7.929 | 7.896 | 7.816 | 6.799 | 6.527 | 6.934 |
| 100505994 | 0 | 1.615 | 0.017 | 0.005 | up-reg. | 6.380 | 5.570 | 5.926 | 4.228 | 3.974 | 4.830 |
| 100506108 | SIX3-AS1 | 1.366 | 0.017 | 0.010 | up-reg. | 6.420 | 5.936 | 5.664 | 4.363 | 4.559 | 4.999 |
| 100506211 | 0 | 0.898 | 0.013 | 0.046 | up-reg. | 5.811 | 5.664 | 5.382 | 4.796 | 4.511 | 4.858 |
| 100506311 | 0 | 1.722 | 0.002 | 0.004 | up-reg. | 8.392 | 8.337 | 8.550 | 6.565 | 6.661 | 6.889 |
| 100506409 | ELOVL2-AS1 | 1.204 | 0.019 | 0.015 | up-reg. | 5.341 | 5.485 | 5.805 | 4.155 | 4.061 | 4.802 |
| 100506696 | PCAT6 | 0.732 | 0.005 | 0.087 | up-reg. | 9.280 | 9.045 | 9.115 | 8.373 | 8.380 | 8.490 |
| 100506783 | 0 | 0.850 | 0.005 | 0.055 | up-reg. | 7.175 | 7.056 | 7.136 | 6.285 | 6.112 | 6.420 |
| 100506834 | GAS1RR | 1.421 | 0.008 | 0.009 | up-reg. | 11.255 | 10.967 | 11.347 | 9.527 | 10.094 | 9.685 |
| 100506885 | LNC-LBCS | 1.642 | 0.015 | 0.005 | up-reg. | 4.243 | 4.414 | 4.529 | 2.521 | 2.367 | 3.371 |
| 100507050 | TPBGL | 1.279 | 0.037 | 0.012 | up-reg. | 6.712 | 6.204 | 6.476 | 4.709 | 4.943 | 5.902 |
| 100507058 | LINC00845 | 1.113 | 0.026 | 0.021 | up-reg. | 5.193 | 4.195 | 4.722 | 3.636 | 3.533 | 3.603 |
| 100507308 | TARID | 0.862 | 0.005 | 0.053 | up-reg. | 6.681 | 6.459 | 6.738 | 5.664 | 5.835 | 5.794 |
| 100507351 | 0 | 1.525 | 0.004 | 0.006 | up-reg. | 10.729 | 10.386 | 10.797 | 8.982 | 9.073 | 9.280 |
| 100507419 | 0 | 1.353 | 0.040 | 0.010 | up-reg. | 11.997 | 11.607 | 12.338 | 9.920 | 11.201 | 10.761 |
| 100507466 | 0 | 1.019 | 0.022 | 0.029 | up-reg. | 4.167 | 4.042 | 4.241 | 3.366 | 2.660 | 3.366 |
| 100507477 | AL357146.1 | 1.355 | 0.004 | 0.010 | up-reg. | 6.491 | 6.222 | 6.178 | 4.824 | 4.885 | 5.116 |
| 100507530 | 0 | 1.061 | 0.009 | 0.025 | up-reg. | 4.828 | 4.428 | 4.688 | 3.563 | 3.413 | 3.786 |
| 100507629 | LINC00658 | 0.705 | 0.044 | 0.096 | up-reg. | 6.341 | 6.098 | 6.407 | 5.150 | 5.800 | 5.779 |
| 100507657 | LINC02554 | 1.662 | 0.003 | 0.005 | up-reg. | 7.127 | 6.722 | 6.809 | 5.281 | 5.250 | 5.142 |
| 100861518 | P4HA2-AS1 | 1.103 | 0.003 | 0.021 | up-reg. | 5.978 | 5.879 | 5.786 | 4.843 | 4.652 | 4.840 |
| 100873944 | PRKX-AS1 | 0.897 | 0.015 | 0.046 | up-reg. | 8.760 | 8.527 | 8.830 | 7.508 | 7.971 | 7.947 |
| 100873947 | LMLN-AS1 | 1.118 | 0.004 | 0.020 | up-reg. | 7.201 | 6.899 | 7.195 | 5.879 | 6.027 | 6.037 |
| 100873949 | IPO9-AS1 | 0.703 | 0.028 | 0.097 | up-reg. | 3.872 | 3.929 | 4.052 | 3.582 | 3.213 | 2.949 |
| 100873963 | 0 | 0.718 | 0.031 | 0.092 | up-reg. | 5.340 | 5.277 | 5.415 | 4.755 | 4.888 | 4.236 |
| 100873982 | ABCC5-AS1 | 1.334 | 0.009 | 0.011 | up-reg. | 11.816 | 11.673 | 11.973 | 10.195 | 10.810 | 10.456 |
| 100874012 | NAV2-AS5 | 1.934 | 0.009 | 0.002 | up-reg. | 8.569 | 8.162 | 8.692 | 6.067 | 6.759 | 6.794 |
| 100874029 | CFAP44-AS1 | 1.440 | 0.042 | 0.008 | up-reg. | 10.995 | 10.708 | 11.289 | 8.818 | 10.329 | 9.527 |
| 100874055 | LINC00570 | 1.399 | 0.026 | 0.009 | up-reg. | 9.298 | 8.753 | 9.760 | 7.750 | 8.302 | 7.563 |
| 100874063 | CLYBL-AS2 | 1.316 | 0.009 | 0.011 | up-reg. | 8.090 | 7.690 | 8.167 | 6.528 | 6.917 | 6.553 |
| 100874097 | UBE2Q1-AS1 | 1.145 | 0.021 | 0.018 | up-reg. | 11.038 | 10.590 | 10.890 | 9.658 | 10.113 | 9.314 |
| 100874130 | 0 | 1.215 | 0.003 | 0.015 | up-reg. | 12.559 | 12.406 | 12.476 | 11.128 | 11.434 | 11.235 |
| 100874159 | 0 | 1.952 | 0.007 | 0.002 | up-reg. | 6.063 | 5.865 | 6.202 | 3.735 | 3.984 | 4.556 |
| 100874238 | ARHGEF7-AS2 | 0.754 | 0.030 | 0.079 | up-reg. | 12.721 | 12.621 | 12.881 | 11.628 | 12.305 | 12.027 |
| 100996425 | ARLNC1 | 2.448 | 0.001 | 0.001 | up-reg. | 6.804 | 6.908 | 6.581 | 4.236 | 4.389 | 4.323 |
| 101060691 | NUTM2B-AS1 | 1.076 | 0.022 | 0.024 | up-reg. | 6.799 | 7.006 | 6.617 | 5.853 | 5.278 | 6.063 |
| 101101772 | LINC00601 | 1.704 | 0.048 | 0.004 | up-reg. | 3.994 | 4.115 | 5.656 | 2.960 | 3.253 | 2.441 |
| 101410540 | FANK1-AS1 | 0.984 | 0.046 | 0.033 | up-reg. | 6.795 | 6.705 | 7.164 | 5.469 | 5.762 | 6.482 |
| 101926996 | 0 | 1.187 | 0.011 | 0.016 | up-reg. | 8.477 | 8.003 | 8.356 | 7.022 | 6.882 | 7.370 |
| 101927038 | LINC02400 | 1.194 | 0.049 | 0.016 | up-reg. | 7.282 | 7.233 | 7.409 | 5.293 | 6.489 | 6.560 |
| 101927049 | PLCE1-AS2 | 1.492 | 0.016 | 0.007 | up-reg. | 7.365 | 6.939 | 7.282 | 5.157 | 5.853 | 6.100 |
| 101927152 | SAMMSON | 1.072 | 0.027 | 0.024 | up-reg. | 5.804 | 5.660 | 5.501 | 4.271 | 4.353 | 5.124 |
| 101927186 | 0 | 1.792 | 0.008 | 0.003 | up-reg. | 6.073 | 5.945 | 5.452 | 3.703 | 4.164 | 4.228 |
| 101927273 | LINC01416 | 0.838 | 0.018 | 0.058 | up-reg. | 4.387 | 3.911 | 3.799 | 3.166 | 3.186 | 3.232 |
| 101927331 | 0 | 1.061 | 0.007 | 0.025 | up-reg. | 6.199 | 5.918 | 5.963 | 4.742 | 5.113 | 5.044 |
| 101927350 | LINC01254 | 1.016 | 0.045 | 0.029 | up-reg. | 4.874 | 4.114 | 3.957 | 3.610 | 2.957 | 3.332 |
| 101927502 | AL591368.1 | 1.619 | 0.011 | 0.005 | up-reg. | 11.212 | 10.933 | 11.422 | 9.100 | 9.771 | 9.838 |
| 101927762 | AL359878.2 | 0.959 | 0.041 | 0.036 | up-reg. | 5.357 | 5.389 | 5.319 | 4.323 | 3.905 | 4.960 |
| 101927766 | LINC01847 | 0.708 | 0.050 | 0.095 | up-reg. | 3.627 | 4.354 | 3.743 | 3.091 | 3.106 | 3.403 |
| 101927861 | 0 | 1.595 | 0.016 | 0.005 | up-reg. | 9.385 | 8.606 | 9.621 | 7.399 | 7.728 | 7.699 |
| 101928000 | ZNF232-AS1 | 0.812 | 0.012 | 0.064 | up-reg. | 8.965 | 9.116 | 8.636 | 8.094 | 8.065 | 8.122 |
| 101928165 | CASC17 | 1.106 | 0.045 | 0.021 | up-reg. | 5.344 | 4.363 | 4.852 | 3.292 | 3.855 | 4.093 |
| 101928168 | AC005232.1 | 1.459 | 0.028 | 0.008 | up-reg. | 8.301 | 8.030 | 8.375 | 6.014 | 7.222 | 7.093 |
| 101928251 | AC124804.1 | 1.621 | 0.006 | 0.005 | up-reg. | 6.694 | 6.191 | 6.300 | 4.515 | 4.882 | 4.924 |
| 101928370 | 0 | 0.777 | 0.013 | 0.073 | up-reg. | 5.543 | 5.297 | 5.150 | 4.662 | 4.378 | 4.619 |
| 101928476 | LINC01676 | 1.660 | 0.018 | 0.005 | up-reg. | 5.548 | 5.107 | 4.824 | 3.115 | 3.315 | 4.069 |
| 101928765 | LINC01807 | 1.622 | 0.006 | 0.005 | up-reg. | 9.874 | 9.437 | 9.697 | 7.759 | 8.102 | 8.281 |
| 101928809 | SDAD1-AS1 | 1.256 | 0.016 | 0.013 | up-reg. | 9.020 | 8.762 | 9.123 | 7.253 | 7.999 | 7.884 |
| 101929010 | SIRPG-AS1 | 0.816 | 0.028 | 0.063 | up-reg. | 6.522 | 6.274 | 6.183 | 5.129 | 5.606 | 5.796 |
| 101929023 | LINC01762 | 1.656 | 0.012 | 0.005 | up-reg. | 9.548 | 9.191 | 9.562 | 7.279 | 8.191 | 7.863 |
| 101929076 | AC009292.1 | 1.736 | 0.004 | 0.004 | up-reg. | 5.697 | 5.433 | 5.659 | 3.813 | 3.615 | 4.153 |
| 101929084 | LINC02401 | 1.393 | 0.019 | 0.009 | up-reg. | 10.481 | 10.239 | 10.456 | 8.474 | 8.991 | 9.532 |
| 101929123 | LINC02493 | 1.745 | 0.025 | 0.004 | up-reg. | 10.331 | 9.742 | 10.505 | 7.690 | 8.950 | 8.704 |
| 101929172 | AC021355.1 | 1.378 | 0.008 | 0.010 | up-reg. | 9.100 | 9.115 | 9.363 | 7.459 | 7.943 | 8.042 |
| 101929202 | 0 | 1.779 | 0.003 | 0.004 | up-reg. | 7.844 | 7.982 | 7.683 | 5.849 | 6.218 | 6.104 |
| 101929237 | AC037441.1 | 1.354 | 0.047 | 0.010 | up-reg. | 5.442 | 4.245 | 5.048 | 3.025 | 3.606 | 4.042 |
| 101929244 | CASC20 | 1.617 | 0.012 | 0.005 | up-reg. | 7.962 | 7.229 | 7.809 | 5.695 | 6.255 | 6.198 |
| 101929261 | LINC02123 | 1.074 | 0.027 | 0.024 | up-reg. | 6.487 | 6.131 | 6.363 | 4.792 | 5.242 | 5.724 |
| 101929340 | AP000866.2 | 1.492 | 0.013 | 0.007 | up-reg. | 7.471 | 6.996 | 7.191 | 5.424 | 5.569 | 6.188 |
| 101929387 | LMF1-AS1 | 1.207 | 0.001 | 0.015 | up-reg. | 8.132 | 8.168 | 8.013 | 6.879 | 6.862 | 6.949 |
| 101929445 | LINC02623 | 0.795 | 0.010 | 0.068 | up-reg. | 5.848 | 5.628 | 5.895 | 4.952 | 5.181 | 4.853 |
| 101929512 | 0 | 0.792 | 0.048 | 0.069 | up-reg. | 5.421 | 5.339 | 5.680 | 4.188 | 4.913 | 4.964 |
| 101929590 | 0 | 1.472 | 0.042 | 0.007 | up-reg. | 10.385 | 10.218 | 10.686 | 8.159 | 9.746 | 8.969 |
| 101929693 | RARA-AS1 | 1.029 | 0.008 | 0.028 | up-reg. | 5.330 | 5.308 | 5.164 | 4.387 | 3.975 | 4.354 |
| 101929719 | AC011379.2 | 0.939 | 0.023 | 0.039 | up-reg. | 8.285 | 7.785 | 8.251 | 7.117 | 7.473 | 6.913 |
| 102659288 | 0 | 1.648 | 0.005 | 0.005 | up-reg. | 8.658 | 8.086 | 8.350 | 6.558 | 6.832 | 6.759 |
| 102723335 | AC027020.1 | 1.037 | 0.033 | 0.027 | up-reg. | 6.166 | 5.609 | 5.660 | 4.328 | 5.175 | 4.821 |
| 102724774 | 0 | 0.973 | 0.038 | 0.034 | up-reg. | 5.332 | 5.233 | 5.303 | 4.015 | 4.015 | 4.919 |
| 102800314 | 0 | 1.848 | 0.010 | 0.003 | up-reg. | 6.506 | 5.634 | 6.087 | 4.052 | 4.068 | 4.564 |
| 103021296 | 0 | 1.602 | 0.020 | 0.005 | up-reg. | 7.570 | 6.633 | 7.331 | 5.222 | 5.474 | 6.034 |
| 103344929 | BGLT3 | 1.472 | 0.010 | 0.007 | up-reg. | 5.273 | 4.933 | 4.821 | 3.140 | 3.706 | 3.765 |
| 103752584 | CCND2-AS1 | 0.857 | 0.042 | 0.054 | up-reg. | 4.878 | 4.255 | 4.706 | 3.383 | 3.791 | 4.095 |
| 104266956 | LINC01044 | 1.017 | 0.006 | 0.029 | up-reg. | 7.291 | 7.305 | 7.012 | 6.150 | 6.351 | 6.055 |
| 104266961 | LINC01221 | 1.005 | 0.032 | 0.030 | up-reg. | 4.902 | 4.086 | 4.538 | 3.639 | 3.171 | 3.702 |
| 104326052 | 0 | 1.054 | 0.019 | 0.026 | up-reg. | 3.800 | 3.945 | 4.015 | 2.432 | 2.978 | 3.190 |
| 104326058 | 0 | 0.979 | 0.020 | 0.033 | up-reg. | 6.399 | 6.290 | 6.151 | 4.963 | 5.239 | 5.701 |
| 105371458 | AL139010.1 | 0.862 | 0.039 | 0.053 | up-reg. | 7.933 | 7.599 | 8.202 | 6.665 | 7.370 | 7.114 |
| 105371809 | 0 | 1.055 | 0.023 | 0.025 | up-reg. | 7.536 | 7.062 | 7.492 | 5.928 | 6.330 | 6.665 |
| 105372516 | 0 | 1.145 | 0.024 | 0.018 | up-reg. | 12.410 | 12.248 | 12.568 | 10.748 | 11.683 | 11.360 |
| 105372950 | LINC02257 | 1.470 | 0.005 | 0.007 | up-reg. | 5.211 | 5.539 | 5.451 | 3.851 | 3.755 | 4.185 |
| 105373869 | 0 | 1.943 | 0.010 | 0.002 | up-reg. | 5.616 | 5.191 | 5.468 | 2.932 | 3.580 | 3.933 |
| 105376736 | EXOSC10-AS1 | 0.720 | 0.012 | 0.091 | up-reg. | 6.831 | 6.700 | 6.806 | 5.920 | 5.956 | 6.303 |
| 105377434 | 0 | 1.597 | 0.039 | 0.005 | up-reg. | 10.023 | 9.305 | 9.598 | 7.138 | 8.548 | 8.450 |
| 105377763 | 0 | 0.801 | 0.014 | 0.067 | up-reg. | 5.712 | 5.826 | 5.348 | 4.782 | 4.777 | 4.925 |
| 105378732 | LINC01771 | 1.519 | 0.007 | 0.006 | up-reg. | 8.564 | 8.242 | 8.897 | 6.980 | 7.155 | 7.010 |
| 105379807 | 0 | 1.325 | 0.005 | 0.011 | up-reg. | 11.622 | 11.332 | 11.607 | 10.051 | 10.414 | 10.120 |
| 105416157 | NKILA | 0.926 | 0.033 | 0.041 | up-reg. | 5.216 | 5.666 | 5.676 | 4.829 | 4.147 | 4.805 |
| 105682749 | 0 | 1.870 | 0.016 | 0.003 | up-reg. | 11.332 | 10.845 | 11.146 | 8.909 | 8.841 | 9.965 |
| 106182249 | LUADT1 | 1.290 | 0.019 | 0.012 | up-reg. | 10.933 | 10.456 | 11.321 | 9.409 | 9.895 | 9.536 |
| 109729161 | LNCSRLR | 0.747 | 0.022 | 0.082 | up-reg. | 4.413 | 3.895 | 4.250 | 3.343 | 3.339 | 3.633 |
| 109729169 | LINC02154 | 3.471 | 0.000 | 0.000 | up-reg. | 7.447 | 7.496 | 7.253 | 3.914 | 3.921 | 3.947 |
| 110091777 | 0 | 1.036 | 0.033 | 0.027 | up-reg. | 7.871 | 7.549 | 7.796 | 6.138 | 7.060 | 6.911 |
| 110806276 | 0 | 1.571 | 0.005 | 0.006 | up-reg. | 6.952 | 6.337 | 6.626 | 5.082 | 5.004 | 5.118 |
| 111216282 | LINC02574 | 1.261 | 0.034 | 0.013 | up-reg. | 6.267 | 6.186 | 6.489 | 4.638 | 5.767 | 4.754 |
| 112637020 | 0 | 1.426 | 0.014 | 0.008 | up-reg. | 8.680 | 8.608 | 8.934 | 6.802 | 7.617 | 7.525 |
| 112840934 | NGF-AS1 | 0.753 | 0.045 | 0.080 | up-reg. | 7.490 | 6.965 | 7.176 | 6.644 | 6.669 | 6.058 |
| 29 | ABR | -0.723 | 0.039 | 0.090 | down-reg. | 5.799 | 5.868 | 5.436 | 6.790 | 6.239 | 6.244 |
| 30 | ACAA1 | -0.776 | 0.040 | 0.074 | down-reg. | 4.560 | 4.700 | 4.828 | 5.848 | 5.520 | 5.048 |
| 113 | ADCY7 | -1.856 | 0.002 | 0.003 | down-reg. | 5.219 | 5.539 | 5.387 | 7.379 | 7.065 | 7.270 |
| 116 | ADCYAP1 | -0.789 | 0.031 | 0.070 | down-reg. | 5.994 | 6.050 | 5.612 | 6.343 | 6.934 | 6.747 |
| 136 | ADORA2B | -1.075 | 0.030 | 0.024 | down-reg. | 5.170 | 5.640 | 4.995 | 5.879 | 6.559 | 6.592 |
| 146 | ADRA1D | -1.106 | 0.004 | 0.022 | down-reg. | 5.949 | 5.976 | 5.818 | 6.979 | 6.864 | 7.218 |
| 147 | ADRA1B | -1.046 | 0.023 | 0.027 | down-reg. | 4.496 | 4.692 | 3.892 | 5.379 | 5.572 | 5.267 |
| 158 | ADSL | -1.427 | 0.000 | 0.009 | down-reg. | 7.856 | 7.721 | 7.749 | 9.250 | 9.171 | 9.185 |
| 199 | AIF1 | -0.805 | 0.014 | 0.066 | down-reg. | 7.656 | 7.580 | 7.785 | 8.202 | 8.561 | 8.673 |
| 292 | SLC25A5 | -0.803 | 0.016 | 0.067 | down-reg. | 5.777 | 5.412 | 5.336 | 6.246 | 6.194 | 6.495 |
| 293 | SLC25A6 | -0.756 | 0.002 | 0.079 | down-reg. | 11.248 | 11.354 | 11.332 | 11.985 | 12.084 | 12.134 |
| 347 | APOD | -1.516 | 0.002 | 0.007 | down-reg. | 11.693 | 11.997 | 11.791 | 13.370 | 13.338 | 13.322 |
| 360 | AQP3 | -1.104 | 0.021 | 0.022 | down-reg. | 4.171 | 4.206 | 4.323 | 5.234 | 4.950 | 5.828 |
| 381 | ARF5 | -0.735 | 0.006 | 0.086 | down-reg. | 12.787 | 12.903 | 12.613 | 13.561 | 13.535 | 13.412 |
| 391 | RHOG | -0.761 | 0.003 | 0.078 | down-reg. | 9.756 | 9.854 | 9.737 | 10.586 | 10.424 | 10.621 |
| 393 | ARHGAP4 | -1.218 | 0.020 | 0.015 | down-reg. | 6.796 | 7.016 | 7.446 | 8.642 | 8.330 | 7.941 |
| 398 | ARHGDIG | -0.847 | 0.027 | 0.056 | down-reg. | 3.966 | 3.390 | 3.252 | 4.349 | 4.489 | 4.310 |
| 432 | ASGR1 | -0.874 | 0.024 | 0.051 | down-reg. | 3.991 | 3.296 | 3.753 | 4.658 | 4.393 | 4.612 |
| 509 | ATP5F1C | -0.730 | 0.005 | 0.088 | down-reg. | 7.643 | 7.542 | 7.533 | 8.422 | 8.154 | 8.332 |
| 513 | ATP5F1D | -0.732 | 0.003 | 0.087 | down-reg. | 9.437 | 9.507 | 9.507 | 10.283 | 10.103 | 10.260 |
| 528 | ATP6V1C1 | -0.830 | 0.006 | 0.060 | down-reg. | 10.236 | 9.953 | 9.934 | 10.965 | 10.817 | 10.829 |
| 547 | KIF1A | -1.031 | 0.039 | 0.028 | down-reg. | 4.349 | 4.353 | 5.002 | 5.293 | 6.071 | 5.432 |
| 573 | BAG1 | -1.538 | 0.042 | 0.006 | down-reg. | 3.672 | 2.620 | 3.790 | 4.832 | 5.498 | 4.366 |
| 578 | BAK1 | -0.787 | 0.047 | 0.071 | down-reg. | 4.726 | 5.348 | 4.965 | 5.989 | 6.002 | 5.408 |
| 586 | BCAT1 | -0.882 | 0.002 | 0.049 | down-reg. | 9.418 | 9.509 | 9.567 | 10.293 | 10.401 | 10.445 |
| 602 | BCL3 | -1.644 | 0.005 | 0.005 | down-reg. | 9.139 | 9.443 | 8.873 | 10.987 | 10.686 | 10.715 |
| 622 | BDH1 | -0.743 | 0.014 | 0.084 | down-reg. | 10.568 | 10.959 | 10.959 | 11.666 | 11.463 | 11.587 |
| 645 | BLVRB | -1.339 | 0.002 | 0.011 | down-reg. | 11.539 | 11.753 | 11.447 | 12.929 | 12.964 | 12.864 |
| 668 | FOXL2 | -1.617 | 0.002 | 0.005 | down-reg. | 5.497 | 5.351 | 5.160 | 6.968 | 6.872 | 7.019 |
| 669 | BPGM | -1.240 | 0.000 | 0.014 | down-reg. | 9.150 | 9.053 | 9.155 | 10.341 | 10.369 | 10.369 |
| 678 | ZFP36L2 | -2.589 | 0.000 | 0.001 | down-reg. | 10.463 | 10.560 | 10.338 | 13.030 | 13.066 | 13.030 |
| 726 | CAPN5 | -1.083 | 0.003 | 0.024 | down-reg. | 9.966 | 10.070 | 9.739 | 11.001 | 10.974 | 11.049 |
| 821 | CANX | -0.837 | 0.049 | 0.058 | down-reg. | 5.960 | 6.253 | 5.707 | 7.239 | 6.793 | 6.398 |
| 822 | CAPG | -1.726 | 0.002 | 0.004 | down-reg. | 6.586 | 6.512 | 6.299 | 8.217 | 8.038 | 8.319 |
| 823 | CAPN1 | -0.946 | 0.034 | 0.038 | down-reg. | 7.402 | 7.441 | 7.213 | 8.382 | 7.795 | 8.719 |
| 871 | SERPINH1 | -1.932 | 0.000 | 0.003 | down-reg. | 12.750 | 12.762 | 12.662 | 14.752 | 14.601 | 14.618 |
| 874 | CBR3 | -2.141 | 0.001 | 0.002 | down-reg. | 10.081 | 10.251 | 9.923 | 12.322 | 12.151 | 12.205 |
| 910 | CD1B | -1.420 | 0.003 | 0.009 | down-reg. | 7.801 | 7.486 | 7.824 | 9.065 | 9.113 | 9.191 |
| 960 | CD44 | -0.726 | 0.002 | 0.090 | down-reg. | 7.896 | 7.858 | 7.811 | 8.626 | 8.488 | 8.626 |
| 991 | CDC20 | -3.078 | 0.000 | 0.000 | down-reg. | 10.785 | 10.693 | 10.761 | 13.752 | 13.875 | 13.846 |
| 998 | CDC42 | -0.796 | 0.004 | 0.069 | down-reg. | 7.713 | 7.737 | 7.554 | 8.554 | 8.361 | 8.477 |
| 1120 | CHKB | -1.527 | 0.000 | 0.006 | down-reg. | 10.453 | 10.526 | 10.427 | 12.014 | 11.942 | 12.031 |
| 1152 | CKB | -1.354 | 0.000 | 0.010 | down-reg. | 10.475 | 10.550 | 10.498 | 11.925 | 11.826 | 11.834 |
| 1207 | CLNS1A | -0.866 | 0.006 | 0.053 | down-reg. | 3.037 | 3.242 | 2.965 | 3.868 | 3.890 | 4.083 |
| 1278 | COL1A2 | -1.250 | 0.000 | 0.014 | down-reg. | 8.801 | 8.721 | 8.707 | 10.044 | 9.941 | 9.993 |
| 1293 | COL6A3 | -0.869 | 0.012 | 0.052 | down-reg. | 6.391 | 6.615 | 6.084 | 7.198 | 7.234 | 7.266 |
| 1296 | COL8A2 | -1.904 | 0.003 | 0.003 | down-reg. | 6.932 | 6.835 | 6.448 | 8.707 | 8.474 | 8.746 |
| 1386 | ATF2 | -0.802 | 0.010 | 0.067 | down-reg. | 5.745 | 5.887 | 5.836 | 6.371 | 6.747 | 6.755 |
| 1396 | CRIP1 | -0.991 | 0.025 | 0.032 | down-reg. | 4.229 | 4.654 | 4.405 | 5.765 | 4.997 | 5.499 |
| 1397 | CRIP2 | -3.287 | 0.001 | 0.000 | down-reg. | 6.846 | 7.087 | 6.691 | 10.213 | 10.026 | 10.244 |
| 1431 | CS | -1.091 | 0.004 | 0.023 | down-reg. | 10.706 | 10.823 | 10.550 | 11.886 | 11.607 | 11.859 |
| 1479 | CSTF3 | -0.796 | 0.004 | 0.069 | down-reg. | 5.868 | 5.915 | 5.894 | 6.793 | 6.544 | 6.728 |
| 1501 | CTNND2 | -1.340 | 0.011 | 0.011 | down-reg. | 3.301 | 3.321 | 3.583 | 4.430 | 5.156 | 4.639 |
| 1518 | 0 | -1.401 | 0.002 | 0.009 | down-reg. | 6.175 | 6.153 | 6.140 | 7.502 | 7.441 | 7.728 |
| 1545 | CYP1B1 | -0.795 | 0.039 | 0.069 | down-reg. | 5.773 | 5.370 | 5.111 | 6.277 | 5.916 | 6.446 |
| 1670 | DEFA5 | -1.596 | 0.015 | 0.005 | down-reg. | 4.400 | 4.443 | 4.359 | 5.352 | 6.391 | 6.246 |
| 1675 | CFD | -2.635 | 0.005 | 0.001 | down-reg. | 4.752 | 4.073 | 4.928 | 7.259 | 6.981 | 7.417 |
| 1718 | DHCR24 | -2.398 | 0.000 | 0.001 | down-reg. | 8.168 | 7.943 | 7.989 | 10.481 | 10.427 | 10.386 |
| 1798 | DPAGT1 | -0.790 | 0.023 | 0.070 | down-reg. | 5.873 | 5.290 | 5.375 | 6.272 | 6.197 | 6.442 |
| 1815 | DRD4 | -1.033 | 0.007 | 0.028 | down-reg. | 5.741 | 6.067 | 5.859 | 6.721 | 7.059 | 6.987 |
| 1822 | ATN1 | -0.765 | 0.007 | 0.077 | down-reg. | 6.227 | 6.095 | 6.248 | 7.131 | 6.822 | 6.912 |
| 1870 | E2F2 | -1.652 | 0.002 | 0.005 | down-reg. | 6.482 | 6.673 | 6.288 | 8.120 | 8.043 | 8.236 |
| 1947 | EFNB1 | -0.935 | 0.021 | 0.040 | down-reg. | 7.117 | 7.148 | 6.766 | 8.184 | 7.583 | 8.070 |
| 1949 | EFNB3 | -1.760 | 0.002 | 0.004 | down-reg. | 5.419 | 5.532 | 5.194 | 7.126 | 7.018 | 7.281 |
| 1999 | ELF3 | -0.923 | 0.013 | 0.042 | down-reg. | 6.132 | 5.804 | 5.683 | 7.009 | 6.686 | 6.694 |
| 2005 | ELK4 | -0.867 | 0.004 | 0.052 | down-reg. | 7.812 | 7.774 | 7.646 | 8.614 | 8.487 | 8.733 |
| 2010 | EMD | -0.892 | 0.004 | 0.048 | down-reg. | 12.510 | 12.575 | 12.322 | 13.400 | 13.284 | 13.400 |
| 2023 | ENO1 | -1.444 | 0.001 | 0.008 | down-reg. | 11.683 | 11.631 | 11.597 | 13.104 | 12.978 | 13.162 |
| 2048 | EPHB2 | -1.750 | 0.001 | 0.004 | down-reg. | 6.930 | 7.041 | 6.844 | 8.753 | 8.624 | 8.689 |
| 2050 | EPHB4 | -0.862 | 0.035 | 0.053 | down-reg. | 3.438 | 3.382 | 3.385 | 4.595 | 4.437 | 3.759 |
| 2067 | ERCC1 | -0.851 | 0.002 | 0.055 | down-reg. | 6.702 | 6.555 | 6.644 | 7.402 | 7.539 | 7.513 |
| 2091 | FBL | -1.274 | 0.001 | 0.013 | down-reg. | 12.449 | 12.418 | 12.427 | 13.650 | 13.802 | 13.665 |
| 2184 | FAH | -0.797 | 0.002 | 0.068 | down-reg. | 10.091 | 10.092 | 10.009 | 10.897 | 10.774 | 10.913 |
| 2194 | FASN | -1.457 | 0.001 | 0.008 | down-reg. | 12.116 | 12.037 | 12.046 | 13.440 | 13.593 | 13.535 |
| 2275 | FHL3 | -1.593 | 0.004 | 0.005 | down-reg. | 6.733 | 6.263 | 6.386 | 8.001 | 7.914 | 8.246 |
| 2323 | FLT3LG | -1.666 | 0.000 | 0.005 | down-reg. | 9.948 | 9.965 | 9.977 | 11.618 | 11.561 | 11.709 |
| 2358 | FPR2 | -1.051 | 0.007 | 0.026 | down-reg. | 3.686 | 3.532 | 3.960 | 4.672 | 4.733 | 4.924 |
| 2650 | GCNT1 | -2.482 | 0.023 | 0.001 | down-reg. | 3.927 | 3.191 | 2.527 | 6.083 | 6.245 | 4.763 |
| 2681 | GGTA1 | -1.555 | 0.035 | 0.006 | down-reg. | 4.946 | 5.447 | 5.413 | 5.955 | 7.209 | 7.305 |
| 2710 | GK | -0.948 | 0.013 | 0.038 | down-reg. | 4.971 | 5.059 | 5.260 | 5.873 | 5.906 | 6.356 |
| 2717 | GLA | -0.775 | 0.034 | 0.074 | down-reg. | 3.529 | 4.096 | 3.738 | 4.880 | 4.423 | 4.385 |
| 2733 | GLE1 | -0.706 | 0.040 | 0.097 | down-reg. | 7.731 | 7.058 | 7.208 | 7.872 | 8.050 | 8.195 |
| 2738 | GLI4 | -1.076 | 0.001 | 0.024 | down-reg. | 10.418 | 10.341 | 10.397 | 11.504 | 11.385 | 11.495 |
| 2773 | GNAI3 | -1.510 | 0.002 | 0.007 | down-reg. | 9.347 | 9.377 | 9.191 | 10.933 | 10.675 | 10.836 |
| 2787 | GNG5 | -0.799 | 0.036 | 0.068 | down-reg. | 5.576 | 5.302 | 4.861 | 5.957 | 5.896 | 6.283 |
| 2788 | GNG7 | -0.914 | 0.002 | 0.044 | down-reg. | 5.140 | 5.190 | 5.191 | 6.036 | 6.005 | 6.220 |
| 2817 | GPC1 | -0.726 | 0.006 | 0.089 | down-reg. | 12.903 | 13.098 | 12.890 | 13.761 | 13.561 | 13.747 |
| 2906 | GRIN2D | -0.738 | 0.011 | 0.085 | down-reg. | 7.967 | 7.990 | 7.750 | 8.727 | 8.431 | 8.763 |
| 2914 | GRM4 | -0.784 | 0.043 | 0.072 | down-reg. | 4.239 | 4.314 | 3.998 | 4.498 | 5.244 | 5.162 |
| 2949 | GSTM5 | -2.734 | 0.001 | 0.001 | down-reg. | 5.845 | 5.922 | 5.528 | 8.564 | 8.342 | 8.589 |
| 2982 | GUCY1A1 | -0.951 | 0.021 | 0.038 | down-reg. | 6.135 | 6.197 | 6.287 | 7.583 | 6.838 | 7.051 |
| 2990 | GUSB | -0.910 | 0.038 | 0.044 | down-reg. | 3.549 | 4.406 | 4.310 | 4.968 | 5.133 | 4.893 |
| 3014 | H2AX | -1.102 | 0.001 | 0.022 | down-reg. | 11.189 | 11.220 | 11.136 | 12.382 | 12.199 | 12.270 |
| 3043 | HBB | -3.275 | 0.001 | 0.000 | down-reg. | 4.016 | 3.577 | 3.952 | 6.883 | 7.249 | 7.238 |
| 3082 | HGF | -2.014 | 0.002 | 0.002 | down-reg. | 3.878 | 4.349 | 3.967 | 5.988 | 6.136 | 6.112 |
| 3146 | HMGB1 | -0.867 | 0.017 | 0.052 | down-reg. | 5.437 | 5.222 | 4.904 | 5.863 | 6.078 | 6.222 |
| 3149 | HMGB3 | -1.425 | 0.002 | 0.009 | down-reg. | 5.063 | 5.261 | 5.116 | 6.711 | 6.401 | 6.605 |
| 3151 | HMGN2 | -1.249 | 0.008 | 0.014 | down-reg. | 5.313 | 5.656 | 5.451 | 6.447 | 6.996 | 6.724 |
| 3162 | HMOX1 | -1.693 | 0.002 | 0.004 | down-reg. | 11.791 | 12.031 | 11.727 | 13.505 | 13.650 | 13.471 |
| 3166 | HMX1 | -0.716 | 0.023 | 0.093 | down-reg. | 5.167 | 5.367 | 5.080 | 6.192 | 5.920 | 5.649 |
| 3191 | HNRNPL | -1.213 | 0.008 | 0.015 | down-reg. | 6.388 | 6.266 | 6.391 | 7.899 | 7.439 | 7.345 |
| 3290 | HSD11B1 | -1.187 | 0.024 | 0.016 | down-reg. | 5.205 | 5.893 | 5.508 | 6.276 | 6.892 | 7.000 |
| 3306 | HSPA2 | -0.959 | 0.011 | 0.037 | down-reg. | 6.310 | 6.415 | 6.121 | 7.441 | 6.962 | 7.321 |
| 3315 | HSPB1 | -1.045 | 0.018 | 0.027 | down-reg. | 7.451 | 7.769 | 7.804 | 9.082 | 8.679 | 8.396 |
| 3422 | IDI1 | -1.004 | 0.029 | 0.031 | down-reg. | 5.872 | 5.855 | 6.083 | 6.423 | 7.327 | 7.072 |
| 3485 | IGFBP2 | -1.660 | 0.001 | 0.005 | down-reg. | 9.295 | 9.431 | 9.283 | 11.108 | 10.928 | 10.952 |
| 3572 | IL6ST | -0.866 | 0.007 | 0.053 | down-reg. | 8.159 | 8.461 | 8.362 | 9.051 | 9.300 | 9.228 |
| 3590 | IL11RA | -1.137 | 0.026 | 0.019 | down-reg. | 6.181 | 6.217 | 5.396 | 7.212 | 6.795 | 7.198 |
| 3609 | ILF3 | -0.965 | 0.003 | 0.036 | down-reg. | 11.662 | 11.861 | 11.666 | 12.770 | 12.594 | 12.721 |
| 3613 | IMPA2 | -0.778 | 0.004 | 0.073 | down-reg. | 7.619 | 7.689 | 7.428 | 8.367 | 8.327 | 8.375 |
| 3615 | IMPDH2 | -2.191 | 0.002 | 0.002 | down-reg. | 6.650 | 6.280 | 6.368 | 8.710 | 8.459 | 8.701 |
| 3630 | INS | -1.271 | 0.004 | 0.013 | down-reg. | 8.062 | 7.957 | 7.676 | 9.130 | 9.064 | 9.313 |
| 3669 | ISG20 | -0.726 | 0.008 | 0.090 | down-reg. | 6.950 | 6.814 | 6.825 | 7.731 | 7.404 | 7.630 |
| 3707 | ITPKB | -1.230 | 0.002 | 0.014 | down-reg. | 10.688 | 10.706 | 10.511 | 11.904 | 11.791 | 11.900 |
| 3732 | CD82 | -0.788 | 0.036 | 0.071 | down-reg. | 4.844 | 4.799 | 4.958 | 6.087 | 5.587 | 5.291 |
| 3735 | KARS1 | -0.734 | 0.006 | 0.087 | down-reg. | 7.195 | 7.039 | 7.128 | 7.695 | 7.961 | 7.911 |
| 3788 | KCNS2 | -1.171 | 0.044 | 0.017 | down-reg. | 5.524 | 6.020 | 6.333 | 6.603 | 7.662 | 7.125 |
| 3835 | KIF22 | -1.694 | 0.001 | 0.004 | down-reg. | 9.728 | 9.806 | 9.765 | 11.557 | 11.347 | 11.479 |
| 3880 | KRT19 | -1.277 | 0.001 | 0.013 | down-reg. | 13.235 | 13.338 | 13.155 | 14.557 | 14.418 | 14.584 |
| 3910 | LAMA4 | -2.158 | 0.001 | 0.002 | down-reg. | 7.831 | 7.888 | 7.676 | 10.077 | 9.826 | 9.966 |
| 3911 | LAMA5 | -2.610 | 0.003 | 0.001 | down-reg. | 6.676 | 6.330 | 5.937 | 9.023 | 8.763 | 8.987 |
| 3920 | LAMP2 | -0.925 | 0.019 | 0.042 | down-reg. | 8.113 | 8.307 | 7.862 | 9.201 | 8.690 | 9.167 |
| 3931 | LCAT | -1.664 | 0.001 | 0.005 | down-reg. | 7.160 | 7.208 | 7.147 | 8.967 | 8.651 | 8.889 |
| 3956 | LGALS1 | -1.015 | 0.005 | 0.030 | down-reg. | 15.675 | 15.675 | 15.675 | 16.520 | 16.628 | 16.922 |
| 3958 | LGALS3 | -1.053 | 0.007 | 0.026 | down-reg. | 6.446 | 6.808 | 6.487 | 7.687 | 7.758 | 7.455 |
| 3991 | LIPE | -1.130 | 0.004 | 0.020 | down-reg. | 8.806 | 9.015 | 8.614 | 9.970 | 9.877 | 9.979 |
| 4000 | LMNA | -1.432 | 0.001 | 0.008 | down-reg. | 11.326 | 11.150 | 11.364 | 12.774 | 12.616 | 12.745 |
| 4123 | MAN2C1 | -0.725 | 0.009 | 0.090 | down-reg. | 7.502 | 7.606 | 7.321 | 8.289 | 8.051 | 8.265 |
| 4130 | MAP1A | -1.286 | 0.001 | 0.012 | down-reg. | 13.711 | 13.752 | 13.665 | 14.969 | 14.941 | 15.076 |
| 4144 | MAT2A | -1.004 | 0.002 | 0.031 | down-reg. | 11.949 | 12.080 | 11.891 | 13.044 | 12.938 | 12.951 |
| 4170 | MCL1 | -0.985 | 0.046 | 0.033 | down-reg. | 3.761 | 3.559 | 3.878 | 5.262 | 4.735 | 4.155 |
| 4171 | MCM2 | -1.636 | 0.009 | 0.005 | down-reg. | 4.464 | 4.757 | 4.377 | 6.113 | 5.805 | 6.589 |
| 4239 | MFAP4 | -1.522 | 0.002 | 0.007 | down-reg. | 10.887 | 11.106 | 10.748 | 12.463 | 12.391 | 12.453 |
| 4298 | MLLT1 | -0.911 | 0.008 | 0.044 | down-reg. | 9.307 | 9.576 | 9.224 | 10.445 | 10.247 | 10.147 |
| 4301 | AFDN | -0.741 | 0.004 | 0.085 | down-reg. | 7.859 | 7.745 | 7.884 | 8.581 | 8.454 | 8.675 |
| 4320 | MMP11 | -5.464 | 0.003 | 0.000 | down-reg. | 5.729 | 4.226 | 4.509 | 10.418 | 10.255 | 10.182 |
| 4435 | CITED1 | -0.934 | 0.024 | 0.040 | down-reg. | 3.972 | 3.747 | 3.332 | 4.499 | 4.449 | 4.904 |
| 4598 | MVK | -1.928 | 0.001 | 0.003 | down-reg. | 9.393 | 9.367 | 9.151 | 11.354 | 11.189 | 11.154 |
| 4599 | MX1 | -0.942 | 0.015 | 0.039 | down-reg. | 5.613 | 5.084 | 5.623 | 6.477 | 6.279 | 6.389 |
| 4600 | MX2 | -0.966 | 0.039 | 0.036 | down-reg. | 4.700 | 4.943 | 4.293 | 5.210 | 5.596 | 6.030 |
| 4649 | MYO9A | -0.917 | 0.004 | 0.043 | down-reg. | 7.902 | 7.850 | 7.983 | 8.785 | 8.708 | 8.994 |
| 4673 | NAP1L1 | -0.739 | 0.008 | 0.085 | down-reg. | 7.627 | 7.608 | 7.397 | 8.414 | 8.298 | 8.137 |
| 4682 | NUBP1 | -1.399 | 0.046 | 0.009 | down-reg. | 2.546 | 3.663 | 3.885 | 5.110 | 4.842 | 4.340 |
| 4700 | NDUFA6 | -0.912 | 0.005 | 0.044 | down-reg. | 7.315 | 7.213 | 7.323 | 8.330 | 8.001 | 8.257 |
| 4736 | RPL10A | -1.383 | 0.003 | 0.010 | down-reg. | 11.100 | 11.328 | 11.139 | 12.396 | 12.662 | 12.659 |
| 4783 | NFIL3 | -1.358 | 0.001 | 0.010 | down-reg. | 7.546 | 7.463 | 7.486 | 8.934 | 8.727 | 8.907 |
| 4799 | NFX1 | -0.822 | 0.014 | 0.062 | down-reg. | 4.213 | 4.424 | 4.018 | 5.023 | 4.869 | 5.230 |
| 4804 | NGFR | -1.573 | 0.020 | 0.006 | down-reg. | 4.260 | 4.541 | 4.836 | 6.009 | 5.625 | 6.722 |
| 4807 | NHLH1 | -1.131 | 0.033 | 0.020 | down-reg. | 5.767 | 6.047 | 5.604 | 6.345 | 7.339 | 7.126 |
| 4809 | SNU13 | -0.812 | 0.008 | 0.065 | down-reg. | 9.285 | 9.158 | 9.450 | 10.089 | 9.977 | 10.263 |
| 4837 | NNMT | -1.432 | 0.001 | 0.008 | down-reg. | 10.312 | 10.195 | 10.169 | 11.727 | 11.568 | 11.678 |
| 4854 | NOTCH3 | -1.798 | 0.007 | 0.003 | down-reg. | 6.739 | 6.935 | 6.354 | 8.445 | 8.174 | 8.806 |
| 4855 | NOTCH4 | -0.787 | 0.049 | 0.071 | down-reg. | 5.449 | 4.824 | 5.641 | 6.125 | 5.873 | 6.275 |
| 4856 | CCN3 | -1.917 | 0.009 | 0.003 | down-reg. | 6.108 | 6.094 | 5.249 | 7.821 | 7.577 | 7.803 |
| 4869 | NPM1 | -2.002 | 0.000 | 0.002 | down-reg. | 11.180 | 11.189 | 11.131 | 13.155 | 13.210 | 13.142 |
| 4885 | NPTX2 | -1.761 | 0.008 | 0.004 | down-reg. | 5.543 | 5.767 | 4.996 | 7.406 | 6.950 | 7.233 |
| 4893 | NRAS | -1.248 | 0.027 | 0.014 | down-reg. | 4.773 | 4.911 | 3.992 | 5.477 | 6.084 | 5.860 |
| 4900 | NRGN | -1.520 | 0.001 | 0.007 | down-reg. | 8.009 | 8.042 | 7.828 | 9.539 | 9.486 | 9.415 |
| 4919 | ROR1 | -2.621 | 0.001 | 0.001 | down-reg. | 5.907 | 5.875 | 5.624 | 8.606 | 8.244 | 8.418 |
| 5106 | PCK2 | -1.000 | 0.002 | 0.031 | down-reg. | 10.823 | 10.913 | 10.785 | 11.849 | 11.753 | 11.918 |
| 5155 | PDGFB | -0.861 | 0.036 | 0.054 | down-reg. | 4.872 | 4.206 | 4.089 | 5.427 | 5.166 | 5.157 |
| 5187 | PER1 | -0.723 | 0.014 | 0.091 | down-reg. | 7.977 | 7.873 | 7.791 | 8.788 | 8.359 | 8.663 |
| 5201 | PFDN1 | -0.739 | 0.020 | 0.085 | down-reg. | 6.616 | 6.612 | 6.628 | 7.217 | 7.163 | 7.693 |
| 5318 | PKP2 | -2.250 | 0.020 | 0.001 | down-reg. | 3.265 | 3.728 | 3.883 | 4.909 | 6.349 | 6.369 |
| 5369 | 0 | -0.902 | 0.047 | 0.046 | down-reg. | 5.282 | 5.408 | 5.797 | 5.882 | 6.758 | 6.554 |
| 5424 | POLD1 | -1.183 | 0.000 | 0.017 | down-reg. | 9.497 | 9.536 | 9.472 | 10.756 | 10.654 | 10.646 |
| 5478 | PPIA | -0.722 | 0.004 | 0.091 | down-reg. | 10.861 | 10.804 | 10.648 | 11.517 | 11.404 | 11.557 |
| 5479 | PPIB | -0.753 | 0.015 | 0.081 | down-reg. | 4.053 | 4.254 | 3.738 | 4.726 | 4.823 | 4.753 |
| 5701 | PSMC2 | -0.723 | 0.002 | 0.090 | down-reg. | 9.482 | 9.634 | 9.515 | 10.314 | 10.213 | 10.273 |
| 5723 | PSPH | -0.707 | 0.006 | 0.096 | down-reg. | 6.719 | 6.962 | 6.723 | 7.566 | 7.535 | 7.426 |
| 5728 | PTEN | -1.923 | 0.001 | 0.003 | down-reg. | 8.313 | 8.263 | 8.153 | 10.214 | 10.051 | 10.232 |
| 5730 | PTGDS | -1.369 | 0.026 | 0.010 | down-reg. | 4.092 | 3.277 | 4.318 | 5.321 | 4.942 | 5.531 |
| 5814 | PURB | -0.930 | 0.003 | 0.041 | down-reg. | 9.681 | 9.777 | 9.594 | 10.711 | 10.516 | 10.613 |
| 5816 | PVALB | -0.935 | 0.005 | 0.040 | down-reg. | 4.191 | 4.402 | 4.295 | 5.320 | 5.330 | 5.045 |
| 5827 | PXMP2 | -0.932 | 0.038 | 0.041 | down-reg. | 5.737 | 5.945 | 5.935 | 6.334 | 7.299 | 6.780 |
| 5866 | RAB3IL1 | -2.117 | 0.001 | 0.002 | down-reg. | 10.511 | 10.566 | 10.361 | 12.653 | 12.510 | 12.625 |
| 5885 | RAD21 | -1.135 | 0.028 | 0.019 | down-reg. | 8.181 | 9.030 | 8.128 | 9.714 | 9.403 | 9.627 |
| 5902 | RANBP1 | -0.837 | 0.001 | 0.058 | down-reg. | 6.413 | 6.347 | 6.251 | 7.158 | 7.187 | 7.176 |
| 5933 | RBL1 | -1.005 | 0.035 | 0.031 | down-reg. | 5.104 | 4.192 | 4.890 | 5.501 | 5.859 | 5.840 |
| 5937 | RBMS1 | -0.820 | 0.003 | 0.063 | down-reg. | 8.781 | 8.814 | 8.632 | 9.624 | 9.504 | 9.559 |
| 6004 | RGS16 | -1.827 | 0.006 | 0.003 | down-reg. | 4.554 | 4.736 | 4.333 | 6.220 | 6.128 | 6.757 |
| 6015 | RING1 | -0.721 | 0.011 | 0.091 | down-reg. | 7.138 | 7.168 | 6.808 | 7.755 | 7.681 | 7.840 |
| 6094 | ROM1 | -1.326 | 0.003 | 0.011 | down-reg. | 8.307 | 8.370 | 8.018 | 9.642 | 9.567 | 9.463 |
| 6124 | RPL4 | -0.874 | 0.002 | 0.051 | down-reg. | 13.172 | 13.284 | 13.113 | 14.109 | 14.020 | 14.060 |
| 6125 | RPL5 | -0.810 | 0.003 | 0.065 | down-reg. | 11.611 | 11.568 | 11.412 | 12.333 | 12.375 | 12.314 |
| 6129 | RPL7 | -0.960 | 0.007 | 0.036 | down-reg. | 8.057 | 7.993 | 7.815 | 9.041 | 8.707 | 8.998 |
| 6132 | RPL8 | -0.839 | 0.005 | 0.058 | down-reg. | 11.399 | 11.495 | 11.377 | 12.375 | 12.088 | 12.325 |
| 6135 | RPL11 | -0.942 | 0.001 | 0.039 | down-reg. | 11.657 | 11.638 | 11.618 | 12.559 | 12.645 | 12.536 |
| 6157 | RPL27A | -1.101 | 0.001 | 0.022 | down-reg. | 7.809 | 7.681 | 7.826 | 8.906 | 8.855 | 8.859 |
| 6158 | RPL28 | -0.774 | 0.010 | 0.074 | down-reg. | 9.236 | 9.234 | 9.203 | 10.158 | 10.091 | 9.746 |
| 6160 | RPL31 | -0.980 | 0.011 | 0.034 | down-reg. | 6.057 | 6.088 | 5.605 | 6.892 | 6.778 | 7.019 |
| 6161 | RPL32 | -0.882 | 0.008 | 0.049 | down-reg. | 8.899 | 8.707 | 8.714 | 9.789 | 9.443 | 9.733 |
| 6188 | RPS3 | -0.873 | 0.001 | 0.051 | down-reg. | 10.179 | 10.110 | 10.214 | 11.078 | 11.078 | 10.967 |
| 6193 | RPS5 | -0.878 | 0.003 | 0.050 | down-reg. | 14.020 | 14.060 | 14.038 | 14.909 | 14.801 | 15.043 |
| 6209 | RPS15 | -0.890 | 0.003 | 0.048 | down-reg. | 10.488 | 10.404 | 10.451 | 11.377 | 11.198 | 11.438 |
| 6217 | RPS16 | -1.080 | 0.003 | 0.024 | down-reg. | 11.868 | 11.738 | 11.805 | 12.745 | 12.871 | 13.035 |
| 6218 | RPS17 | -0.845 | 0.001 | 0.057 | down-reg. | 9.803 | 9.817 | 9.789 | 10.642 | 10.706 | 10.596 |
| 6230 | RPS25 | -0.783 | 0.002 | 0.072 | down-reg. | 11.395 | 11.242 | 11.263 | 12.097 | 12.063 | 12.088 |
| 6234 | RPS28 | -0.875 | 0.005 | 0.051 | down-reg. | 9.222 | 9.411 | 9.225 | 10.311 | 10.092 | 10.079 |
| 6274 | S100A3 | -1.146 | 0.000 | 0.019 | down-reg. | 10.271 | 10.214 | 10.255 | 11.419 | 11.342 | 11.416 |
| 6286 | S100P | -0.833 | 0.036 | 0.059 | down-reg. | 3.594 | 3.147 | 3.578 | 4.440 | 3.863 | 4.515 |
| 6297 | SALL2 | -1.760 | 0.003 | 0.004 | down-reg. | 7.112 | 7.205 | 6.835 | 8.945 | 8.596 | 8.891 |
| 6386 | SDCBP | -0.716 | 0.004 | 0.093 | down-reg. | 12.060 | 11.868 | 11.933 | 12.731 | 12.580 | 12.697 |
| 6387 | CXCL12 | -1.172 | 0.005 | 0.017 | down-reg. | 4.991 | 5.200 | 5.353 | 6.426 | 6.467 | 6.166 |
| 6421 | SFPQ | -2.265 | 0.000 | 0.001 | down-reg. | 8.788 | 8.587 | 8.647 | 10.990 | 10.924 | 10.902 |
| 6431 | SRSF6 | -1.097 | 0.004 | 0.022 | down-reg. | 9.426 | 9.548 | 9.212 | 10.468 | 10.433 | 10.578 |
| 6497 | SKI | -1.182 | 0.002 | 0.017 | down-reg. | 8.849 | 9.010 | 8.803 | 10.009 | 10.084 | 10.117 |
| 6502 | SKP2 | -1.288 | 0.002 | 0.012 | down-reg. | 8.126 | 8.178 | 8.010 | 9.460 | 9.270 | 9.450 |
| 6509 | SLC1A4 | -1.029 | 0.035 | 0.028 | down-reg. | 4.566 | 4.656 | 3.789 | 5.504 | 5.490 | 5.102 |
| 6541 | SLC7A1 | -0.790 | 0.004 | 0.070 | down-reg. | 4.192 | 4.336 | 4.198 | 4.953 | 5.172 | 4.971 |
| 6578 | SLCO2A1 | -1.849 | 0.002 | 0.003 | down-reg. | 6.689 | 6.446 | 6.365 | 8.164 | 8.372 | 8.511 |
| 6603 | SMARCD2 | -0.986 | 0.004 | 0.033 | down-reg. | 11.305 | 11.481 | 11.385 | 12.520 | 12.248 | 12.362 |
| 6611 | SMS | -1.071 | 0.001 | 0.025 | down-reg. | 10.341 | 10.286 | 10.257 | 11.460 | 11.277 | 11.358 |
| 6614 | SIGLEC1 | -1.491 | 0.022 | 0.007 | down-reg. | 4.822 | 5.788 | 5.780 | 6.698 | 7.279 | 6.885 |
| 6615 | SNAI1 | -1.516 | 0.001 | 0.007 | down-reg. | 5.732 | 5.870 | 5.658 | 7.282 | 7.291 | 7.234 |
| 6624 | FSCN1 | -2.394 | 0.001 | 0.001 | down-reg. | 12.532 | 12.721 | 12.388 | 15.007 | 14.909 | 14.909 |
| 6626 | SNRPA | -1.381 | 0.001 | 0.010 | down-reg. | 11.973 | 12.163 | 12.006 | 13.481 | 13.412 | 13.393 |
| 6627 | SNRPA1 | -0.835 | 0.006 | 0.059 | down-reg. | 7.969 | 8.073 | 7.767 | 8.882 | 8.716 | 8.714 |
| 6628 | SNRPB | -0.779 | 0.006 | 0.073 | down-reg. | 9.554 | 9.352 | 9.548 | 10.218 | 10.161 | 10.411 |
| 6636 | SNRPF | -0.962 | 0.005 | 0.036 | down-reg. | 8.822 | 8.950 | 8.615 | 9.879 | 9.683 | 9.711 |
| 6650 | CAPN15 | -1.111 | 0.002 | 0.021 | down-reg. | 10.928 | 10.965 | 10.809 | 12.046 | 11.909 | 12.080 |
| 6659 | SOX4 | -0.988 | 0.007 | 0.033 | down-reg. | 7.218 | 7.098 | 6.890 | 8.191 | 7.867 | 8.113 |
| 6726 | SRP9 | -0.792 | 0.005 | 0.070 | down-reg. | 11.980 | 12.151 | 11.904 | 12.890 | 12.725 | 12.796 |
| 6794 | STK11 | -0.858 | 0.010 | 0.054 | down-reg. | 7.028 | 7.209 | 6.952 | 7.709 | 7.924 | 8.130 |
| 6872 | TAF1 | -0.731 | 0.013 | 0.088 | down-reg. | 8.667 | 8.690 | 8.372 | 9.486 | 9.200 | 9.236 |
| 6909 | TBX2 | -1.105 | 0.006 | 0.022 | down-reg. | 8.529 | 8.117 | 8.488 | 9.549 | 9.418 | 9.482 |
| 6944 | VPS72 | -0.846 | 0.043 | 0.057 | down-reg. | 4.470 | 5.078 | 5.191 | 5.488 | 6.046 | 5.743 |
| 7076 | TIMP1 | -0.910 | 0.030 | 0.044 | down-reg. | 5.531 | 5.123 | 5.025 | 6.037 | 5.845 | 6.527 |
| 7083 | TK1 | -2.571 | 0.000 | 0.001 | down-reg. | 10.890 | 11.049 | 10.838 | 13.528 | 13.457 | 13.505 |
| 7090 | TLE3 | -1.285 | 0.002 | 0.012 | down-reg. | 6.876 | 6.699 | 6.557 | 7.999 | 8.014 | 7.973 |
| 7113 | TMPRSS2 | -0.969 | 0.006 | 0.035 | down-reg. | 8.616 | 8.824 | 8.950 | 9.923 | 9.704 | 9.669 |
| 7283 | TUBG1 | -0.733 | 0.017 | 0.087 | down-reg. | 7.519 | 7.868 | 7.741 | 8.534 | 8.583 | 8.209 |
| 7291 | TWIST1 | -2.102 | 0.001 | 0.002 | down-reg. | 8.492 | 8.486 | 8.342 | 10.706 | 10.453 | 10.468 |
| 7295 | TXN | -0.852 | 0.001 | 0.055 | down-reg. | 10.300 | 10.314 | 10.263 | 11.204 | 11.158 | 11.071 |
| 7301 | TYRO3 | -0.845 | 0.003 | 0.057 | down-reg. | 9.872 | 9.979 | 9.734 | 10.765 | 10.660 | 10.696 |
| 7468 | NSD2 | -0.809 | 0.011 | 0.065 | down-reg. | 5.541 | 5.416 | 5.843 | 6.358 | 6.329 | 6.540 |
| 7481 | WNT11 | -2.198 | 0.004 | 0.002 | down-reg. | 4.990 | 5.501 | 4.731 | 7.188 | 7.264 | 7.365 |
| 7482 | WNT2B | -0.837 | 0.005 | 0.058 | down-reg. | 9.697 | 9.598 | 9.856 | 10.516 | 10.488 | 10.657 |
| 7551 | ZNF3 | -0.740 | 0.008 | 0.085 | down-reg. | 7.299 | 7.412 | 7.177 | 8.168 | 7.882 | 8.057 |
| 7570 | ZNF22 | -1.122 | 0.015 | 0.020 | down-reg. | 6.686 | 6.651 | 6.104 | 7.628 | 7.369 | 7.811 |
| 7639 | ZNF85 | -1.419 | 0.009 | 0.009 | down-reg. | 4.046 | 4.634 | 3.989 | 5.662 | 5.508 | 5.757 |
| 7846 | TUBA1A | -1.272 | 0.001 | 0.013 | down-reg. | 13.640 | 13.665 | 13.619 | 15.043 | 14.874 | 14.825 |
| 7923 | HSD17B8 | -1.490 | 0.003 | 0.007 | down-reg. | 5.830 | 5.877 | 5.508 | 7.184 | 7.127 | 7.375 |
| 8128 | ST8SIA2 | -0.752 | 0.018 | 0.081 | down-reg. | 5.841 | 5.665 | 5.506 | 6.158 | 6.549 | 6.562 |
| 8225 | GTPBP6 | -1.460 | 0.001 | 0.008 | down-reg. | 10.924 | 10.780 | 10.933 | 12.427 | 12.211 | 12.378 |
| 8303 | SNN | -1.589 | 0.003 | 0.005 | down-reg. | 7.041 | 7.018 | 6.612 | 8.482 | 8.396 | 8.560 |
| 8420 | SNHG3 | -1.133 | 0.008 | 0.020 | down-reg. | 5.504 | 5.139 | 5.556 | 6.546 | 6.354 | 6.697 |
| 8492 | PRSS12 | -2.354 | 0.001 | 0.001 | down-reg. | 7.590 | 7.730 | 7.340 | 9.965 | 9.789 | 9.970 |
| 8501 | SLC43A1 | -1.221 | 0.015 | 0.015 | down-reg. | 5.372 | 5.344 | 5.131 | 6.676 | 6.040 | 6.795 |
| 8508 | NIPSNAP1 | -0.840 | 0.004 | 0.058 | down-reg. | 10.859 | 10.999 | 10.729 | 11.747 | 11.657 | 11.704 |
| 8525 | DGKZ | -0.872 | 0.034 | 0.051 | down-reg. | 4.962 | 4.896 | 4.306 | 5.825 | 5.640 | 5.313 |
| 8537 | BCAS1 | -0.825 | 0.039 | 0.062 | down-reg. | 4.655 | 5.013 | 4.146 | 5.433 | 5.529 | 5.326 |
| 8558 | CDK10 | -1.365 | 0.012 | 0.010 | down-reg. | 4.547 | 4.054 | 4.097 | 5.977 | 5.422 | 5.394 |
| 8560 | DEGS1 | -1.361 | 0.002 | 0.010 | down-reg. | 8.085 | 7.999 | 7.883 | 9.263 | 9.276 | 9.513 |
| 8620 | NPFF | -1.973 | 0.002 | 0.002 | down-reg. | 7.683 | 7.321 | 7.319 | 9.492 | 9.266 | 9.486 |
| 8660 | IRS2 | -1.505 | 0.002 | 0.007 | down-reg. | 11.709 | 11.816 | 11.517 | 13.201 | 13.201 | 13.155 |
| 8664 | EIF3D | -0.734 | 0.004 | 0.087 | down-reg. | 11.381 | 11.511 | 11.360 | 12.276 | 12.060 | 12.116 |
| 8665 | EIF3F | -0.942 | 0.002 | 0.039 | down-reg. | 9.609 | 9.495 | 9.652 | 10.608 | 10.445 | 10.529 |
| 8702 | B4GALT4 | -1.369 | 0.048 | 0.010 | down-reg. | 3.679 | 4.531 | 4.443 | 4.827 | 6.023 | 5.910 |
| 8748 | ADAM20 | -1.985 | 0.026 | 0.002 | down-reg. | 4.002 | 4.963 | 3.534 | 5.550 | 6.536 | 6.368 |
| 8787 | RGS9 | -0.875 | 0.006 | 0.051 | down-reg. | 7.207 | 7.047 | 6.855 | 7.873 | 7.870 | 7.990 |
| 8788 | DLK1 | -1.471 | 0.036 | 0.007 | down-reg. | 4.039 | 3.255 | 4.654 | 5.250 | 5.282 | 5.830 |
| 8801 | SUCLG2 | -1.433 | 0.006 | 0.008 | down-reg. | 6.951 | 6.860 | 6.639 | 8.051 | 8.137 | 8.561 |
| 8825 | LIN7A | -1.584 | 0.008 | 0.006 | down-reg. | 5.546 | 5.692 | 5.202 | 6.700 | 7.331 | 7.161 |
| 8833 | GMPS | -1.059 | 0.006 | 0.026 | down-reg. | 9.153 | 9.219 | 8.847 | 10.251 | 10.073 | 10.072 |
| 8839 | CCN5 | -2.231 | 0.001 | 0.001 | down-reg. | 5.387 | 5.280 | 5.525 | 7.707 | 7.482 | 7.695 |
| 8844 | KSR1 | -0.849 | 0.013 | 0.056 | down-reg. | 6.745 | 6.849 | 7.198 | 7.610 | 7.850 | 7.878 |
| 8880 | FUBP1 | -2.270 | 0.005 | 0.001 | down-reg. | 5.728 | 5.760 | 5.017 | 7.888 | 7.710 | 7.718 |
| 8905 | AP1S2 | -1.180 | 0.003 | 0.017 | down-reg. | 7.837 | 7.969 | 7.747 | 9.018 | 8.920 | 9.155 |
| 8988 | HSPB3 | -2.738 | 0.000 | 0.001 | down-reg. | 7.233 | 7.163 | 7.259 | 10.107 | 9.840 | 9.920 |
| 8991 | SELENBP1 | -1.768 | 0.001 | 0.004 | down-reg. | 9.840 | 9.957 | 9.872 | 11.757 | 11.517 | 11.698 |
| 9021 | SOCS3 | -1.127 | 0.001 | 0.020 | down-reg. | 10.048 | 10.069 | 10.171 | 11.242 | 11.131 | 11.296 |
| 9037 | SEMA5A | -1.205 | 0.001 | 0.016 | down-reg. | 8.428 | 8.365 | 8.301 | 9.634 | 9.527 | 9.549 |
| 9064 | MAP3K6 | -1.679 | 0.000 | 0.004 | down-reg. | 10.902 | 10.850 | 10.900 | 12.580 | 12.508 | 12.601 |
| 9097 | USP14 | -2.106 | 0.049 | 0.002 | down-reg. | 4.459 | 4.877 | 3.127 | 5.289 | 6.991 | 6.500 |
| 9123 | SLC16A3 | -0.963 | 0.033 | 0.036 | down-reg. | 6.574 | 6.621 | 6.858 | 8.180 | 7.414 | 7.349 |
| 9140 | ATG12 | -0.798 | 0.024 | 0.068 | down-reg. | 5.782 | 5.949 | 6.399 | 6.747 | 7.006 | 6.771 |
| 9143 | SYNGR3 | -2.996 | 0.000 | 0.000 | down-reg. | 7.559 | 7.422 | 7.240 | 10.338 | 10.406 | 10.465 |
| 9168 | TMSB10 | -0.991 | 0.001 | 0.032 | down-reg. | 11.552 | 11.572 | 11.662 | 12.616 | 12.625 | 12.520 |
| 9170 | LPAR2 | -0.701 | 0.004 | 0.098 | down-reg. | 7.828 | 7.674 | 7.612 | 8.449 | 8.350 | 8.419 |
| 9182 | RASSF9 | -1.563 | 0.017 | 0.006 | down-reg. | 2.713 | 3.784 | 3.282 | 4.753 | 4.636 | 5.079 |
| 9211 | LGI1 | -0.745 | 0.018 | 0.083 | down-reg. | 3.402 | 3.684 | 3.250 | 4.082 | 4.097 | 4.390 |
| 9244 | CRLF1 | -5.497 | 0.000 | 0.000 | down-reg. | 4.528 | 4.369 | 4.353 | 9.869 | 9.848 | 10.024 |
| 9253 | NUMBL | -2.264 | 0.002 | 0.001 | down-reg. | 6.343 | 6.328 | 5.976 | 8.583 | 8.260 | 8.596 |
| 9270 | ITGB1BP1 | -0.754 | 0.037 | 0.080 | down-reg. | 3.371 | 3.402 | 2.809 | 3.856 | 3.795 | 4.194 |
| 9329 | GTF3C4 | -0.907 | 0.020 | 0.045 | down-reg. | 7.932 | 8.109 | 7.580 | 9.001 | 8.523 | 8.817 |
| 9330 | GTF3C3 | -0.931 | 0.043 | 0.041 | down-reg. | 4.476 | 4.571 | 4.562 | 5.584 | 4.895 | 5.922 |
| 9361 | LONP1 | -0.865 | 0.027 | 0.053 | down-reg. | 4.924 | 4.221 | 4.753 | 5.326 | 5.646 | 5.522 |
| 9363 | RAB33A | -2.276 | 0.004 | 0.001 | down-reg. | 6.206 | 6.107 | 5.605 | 8.409 | 8.005 | 8.331 |
| 9368 | SLC9A3R1 | -0.816 | 0.002 | 0.064 | down-reg. | 11.308 | 11.358 | 11.252 | 12.142 | 12.039 | 12.185 |
| 9498 | SLC4A8 | -1.329 | 0.007 | 0.011 | down-reg. | 6.531 | 6.278 | 5.991 | 7.625 | 7.442 | 7.719 |
| 9509 | ADAMTS2 | -2.429 | 0.001 | 0.001 | down-reg. | 8.342 | 8.180 | 8.361 | 10.708 | 10.603 | 10.859 |
| 9517 | SPTLC2 | -1.060 | 0.005 | 0.026 | down-reg. | 8.644 | 8.760 | 8.391 | 9.737 | 9.573 | 9.663 |
| 9668 | ZNF432 | -0.718 | 0.040 | 0.092 | down-reg. | 3.781 | 3.187 | 3.910 | 4.441 | 4.308 | 4.283 |
| 9672 | SDC3 | -1.549 | 0.003 | 0.006 | down-reg. | 8.785 | 8.975 | 8.773 | 10.210 | 10.578 | 10.392 |
| 9696 | CROCC | -1.674 | 0.002 | 0.004 | down-reg. | 7.144 | 7.123 | 6.834 | 8.763 | 8.640 | 8.721 |
| 9768 | PCLAF | -1.156 | 0.030 | 0.018 | down-reg. | 3.484 | 4.268 | 3.533 | 5.261 | 4.576 | 4.916 |
| 9784 | SNX17 | -0.862 | 0.013 | 0.053 | down-reg. | 6.568 | 6.594 | 6.401 | 7.422 | 7.108 | 7.621 |
| 9811 | CTIF | -0.991 | 0.008 | 0.032 | down-reg. | 7.668 | 7.784 | 7.959 | 9.028 | 8.692 | 8.665 |
| 9824 | ARHGAP11A | -2.076 | 0.014 | 0.002 | down-reg. | 5.409 | 5.413 | 4.200 | 7.162 | 6.970 | 7.117 |
| 9839 | ZEB2 | -1.563 | 0.010 | 0.006 | down-reg. | 4.429 | 3.949 | 4.231 | 5.782 | 5.373 | 6.145 |
| 9904 | RBM19 | -1.190 | 0.007 | 0.016 | down-reg. | 6.334 | 6.135 | 5.866 | 7.177 | 7.265 | 7.465 |
| 9987 | HNRNPDL | -1.055 | 0.005 | 0.026 | down-reg. | 6.006 | 6.088 | 6.046 | 7.322 | 7.046 | 6.937 |
| 10000 | AKT3 | -0.724 | 0.002 | 0.090 | down-reg. | 8.602 | 8.616 | 8.500 | 9.308 | 9.231 | 9.352 |
| 10004 | NAALADL1 | -1.317 | 0.000 | 0.011 | down-reg. | 8.589 | 8.628 | 8.646 | 9.946 | 9.920 | 9.948 |
| 10123 | ARL4C | -2.330 | 0.001 | 0.001 | down-reg. | 7.103 | 6.816 | 6.756 | 9.183 | 9.105 | 9.377 |
| 10163 | WASF2 | -1.546 | 0.001 | 0.006 | down-reg. | 6.708 | 6.659 | 6.490 | 8.244 | 8.049 | 8.205 |
| 10179 | RBM7 | -0.866 | 0.040 | 0.053 | down-reg. | 5.031 | 4.161 | 4.491 | 5.545 | 5.226 | 5.510 |
| 10188 | TNK2 | -1.202 | 0.028 | 0.016 | down-reg. | 6.095 | 5.787 | 5.761 | 7.596 | 7.122 | 6.530 |
| 10212 | DDX39A | -1.762 | 0.001 | 0.004 | down-reg. | 6.296 | 6.456 | 6.320 | 8.214 | 8.036 | 8.109 |
| 10267 | RAMP1 | -1.044 | 0.005 | 0.027 | down-reg. | 7.162 | 7.268 | 6.975 | 8.310 | 8.008 | 8.219 |
| 10288 | LILRB2 | -1.810 | 0.035 | 0.003 | down-reg. | 3.239 | 4.755 | 4.140 | 5.228 | 6.221 | 6.114 |
| 10290 | SPEG | -0.765 | 0.003 | 0.077 | down-reg. | 9.957 | 9.929 | 9.869 | 10.780 | 10.586 | 10.686 |
| 10316 | NMUR1 | -1.664 | 0.034 | 0.005 | down-reg. | 3.454 | 4.038 | 4.488 | 4.882 | 5.950 | 6.140 |
| 10376 | TUBA1B | -1.833 | 0.000 | 0.003 | down-reg. | 13.729 | 13.782 | 13.861 | 15.675 | 15.598 | 15.598 |
| 10381 | TUBB3 | -3.525 | 0.001 | 0.000 | down-reg. | 8.885 | 8.965 | 8.655 | 12.514 | 12.167 | 12.399 |
| 10382 | TUBB4A | -1.195 | 0.002 | 0.016 | down-reg. | 8.649 | 8.653 | 8.703 | 9.844 | 9.728 | 10.018 |
| 10399 | RACK1 | -0.835 | 0.004 | 0.059 | down-reg. | 15.194 | 15.465 | 15.296 | 16.192 | 16.135 | 16.135 |
| 10409 | BASP1 | -0.751 | 0.011 | 0.081 | down-reg. | 9.131 | 9.006 | 9.041 | 10.056 | 9.685 | 9.688 |
| 10439 | OLFM1 | -1.400 | 0.002 | 0.009 | down-reg. | 5.404 | 5.564 | 5.342 | 6.860 | 6.704 | 6.948 |
| 10465 | PPIH | -1.504 | 0.003 | 0.007 | down-reg. | 9.259 | 9.487 | 9.081 | 10.853 | 10.690 | 10.797 |
| 10468 | FST | -3.092 | 0.000 | 0.000 | down-reg. | 7.763 | 7.619 | 7.537 | 10.739 | 10.680 | 10.777 |
| 10469 | TIMM44 | -0.895 | 0.010 | 0.047 | down-reg. | 8.880 | 8.903 | 8.536 | 9.808 | 9.489 | 9.706 |
| 10493 | VAT1 | -0.717 | 0.001 | 0.093 | down-reg. | 14.584 | 14.504 | 14.532 | 15.268 | 15.233 | 15.268 |
| 10536 | P3H3 | -0.839 | 0.021 | 0.058 | down-reg. | 4.072 | 4.416 | 4.062 | 5.212 | 5.155 | 4.700 |
| 10541 | ANP32B | -1.302 | 0.001 | 0.012 | down-reg. | 8.014 | 7.917 | 7.902 | 9.314 | 9.156 | 9.269 |
| 10595 | ERN2 | -2.349 | 0.001 | 0.001 | down-reg. | 6.569 | 6.491 | 6.164 | 8.841 | 8.665 | 8.764 |
| 10602 | CDC42EP3 | -1.662 | 0.001 | 0.005 | down-reg. | 7.971 | 8.141 | 7.990 | 9.754 | 9.605 | 9.728 |
| 10620 | ARID3B | -1.254 | 0.009 | 0.013 | down-reg. | 5.913 | 5.989 | 5.379 | 7.024 | 6.932 | 7.085 |
| 10682 | EBP | -0.905 | 0.004 | 0.045 | down-reg. | 10.955 | 11.166 | 10.864 | 11.938 | 11.891 | 11.871 |
| 10766 | TOB2 | -0.731 | 0.006 | 0.088 | down-reg. | 9.489 | 9.569 | 9.403 | 10.368 | 10.147 | 10.140 |
| 10811 | NOXA1 | -0.830 | 0.003 | 0.060 | down-reg. | 7.011 | 7.009 | 6.850 | 7.856 | 7.698 | 7.805 |
| 10841 | FTCD | -1.233 | 0.006 | 0.014 | down-reg. | 6.651 | 6.622 | 6.237 | 7.571 | 7.840 | 7.798 |
| 10859 | LILRB1 | -0.785 | 0.020 | 0.071 | down-reg. | 7.905 | 7.986 | 8.465 | 8.965 | 8.947 | 8.801 |
| 10882 | C1QL1 | -0.810 | 0.014 | 0.065 | down-reg. | 9.534 | 9.627 | 9.815 | 10.200 | 10.624 | 10.580 |
| 10905 | MAN1A2 | -1.160 | 0.003 | 0.018 | down-reg. | 9.450 | 9.624 | 9.301 | 10.621 | 10.583 | 10.650 |
| 10923 | SUB1 | -0.924 | 0.012 | 0.042 | down-reg. | 6.847 | 7.042 | 6.649 | 7.858 | 7.529 | 7.924 |
| 10926 | DBF4 | -0.847 | 0.010 | 0.056 | down-reg. | 5.746 | 5.535 | 5.616 | 6.296 | 6.425 | 6.716 |
| 10946 | SF3A3 | -0.797 | 0.006 | 0.068 | down-reg. | 8.042 | 8.081 | 8.113 | 8.822 | 9.062 | 8.742 |
| 10988 | METAP2 | -1.072 | 0.007 | 0.025 | down-reg. | 7.152 | 7.049 | 7.112 | 8.020 | 8.460 | 8.050 |
| 11000 | SLC27A3 | -2.005 | 0.001 | 0.002 | down-reg. | 6.935 | 7.105 | 6.805 | 9.008 | 8.871 | 8.980 |
| 11007 | CCDC85B | -0.789 | 0.001 | 0.070 | down-reg. | 12.858 | 12.881 | 12.822 | 13.684 | 13.684 | 13.561 |
| 11023 | VAX1 | -1.540 | 0.030 | 0.006 | down-reg. | 6.016 | 7.082 | 6.754 | 7.604 | 8.604 | 8.263 |
| 11083 | DIDO1 | -0.942 | 0.022 | 0.039 | down-reg. | 6.072 | 6.350 | 6.191 | 7.553 | 7.050 | 6.834 |
| 11117 | EMILIN1 | -0.954 | 0.001 | 0.037 | down-reg. | 13.914 | 13.914 | 13.950 | 14.874 | 14.825 | 14.941 |
| 11133 | KPTN | -1.054 | 0.003 | 0.026 | down-reg. | 9.203 | 9.179 | 8.997 | 10.316 | 10.117 | 10.107 |
| 11138 | TBC1D8 | -1.848 | 0.003 | 0.003 | down-reg. | 7.065 | 6.847 | 6.564 | 8.730 | 8.508 | 8.782 |
| 11165 | NUDT3 | -1.204 | 0.008 | 0.016 | down-reg. | 8.917 | 9.211 | 8.631 | 10.227 | 10.070 | 10.073 |
| 11180 | WDR6 | -0.914 | 0.001 | 0.043 | down-reg. | 10.624 | 10.613 | 10.538 | 11.499 | 11.442 | 11.577 |
| 11285 | B4GALT7 | -1.021 | 0.011 | 0.029 | down-reg. | 6.756 | 6.646 | 6.304 | 7.366 | 7.749 | 7.654 |
| 22850 | ADNP2 | -0.900 | 0.036 | 0.046 | down-reg. | 5.307 | 4.625 | 4.697 | 5.573 | 6.106 | 5.650 |
| 22859 | ADGRL1 | -0.715 | 0.039 | 0.094 | down-reg. | 5.533 | 5.426 | 4.950 | 5.814 | 5.968 | 6.272 |
| 22902 | RUFY3 | -1.587 | 0.003 | 0.006 | down-reg. | 8.100 | 8.231 | 7.800 | 9.693 | 9.576 | 9.623 |
| 22936 | ELL2 | -0.833 | 0.024 | 0.059 | down-reg. | 6.762 | 7.258 | 6.993 | 7.535 | 7.978 | 7.999 |
| 22973 | 0 | -0.826 | 0.019 | 0.061 | down-reg. | 4.546 | 5.010 | 4.888 | 5.413 | 5.675 | 5.835 |
| 22980 | TCF25 | -0.722 | 0.002 | 0.091 | down-reg. | 12.520 | 12.432 | 12.436 | 13.243 | 13.122 | 13.188 |
| 23052 | ENDOD1 | -0.802 | 0.008 | 0.067 | down-reg. | 8.962 | 9.228 | 8.859 | 9.871 | 9.760 | 9.822 |
| 23086 | EXPH5 | -1.932 | 0.014 | 0.003 | down-reg. | 4.933 | 4.614 | 3.776 | 6.371 | 6.121 | 6.629 |
| 23089 | PEG10 | -0.943 | 0.002 | 0.039 | down-reg. | 7.984 | 7.914 | 7.971 | 8.797 | 8.938 | 8.963 |
| 23095 | KIF1B | -0.729 | 0.018 | 0.088 | down-reg. | 5.993 | 6.086 | 5.949 | 7.045 | 6.603 | 6.566 |
| 23107 | MRPS27 | -0.964 | 0.000 | 0.036 | down-reg. | 10.765 | 10.771 | 10.765 | 11.771 | 11.704 | 11.716 |
| 23119 | HIC2 | -0.970 | 0.002 | 0.035 | down-reg. | 6.514 | 6.343 | 6.356 | 7.384 | 7.303 | 7.435 |
| 23151 | GRAMD4 | -0.832 | 0.042 | 0.060 | down-reg. | 5.950 | 5.593 | 5.209 | 6.274 | 6.231 | 6.744 |
| 23197 | FAF2 | -0.871 | 0.029 | 0.051 | down-reg. | 3.196 | 3.593 | 3.979 | 4.581 | 4.504 | 4.296 |
| 23254 | KAZN | -1.200 | 0.033 | 0.016 | down-reg. | 4.367 | 4.182 | 3.553 | 5.410 | 4.742 | 5.550 |
| 23277 | CLUH | -0.774 | 0.002 | 0.074 | down-reg. | 10.936 | 10.864 | 10.810 | 11.721 | 11.581 | 11.631 |
| 23316 | CUX2 | -1.124 | 0.008 | 0.020 | down-reg. | 5.357 | 5.407 | 5.649 | 6.568 | 6.844 | 6.371 |
| 23373 | CRTC1 | -2.545 | 0.007 | 0.001 | down-reg. | 5.232 | 5.554 | 4.503 | 7.769 | 7.355 | 7.800 |
| 23518 | R3HDM1 | -0.749 | 0.002 | 0.082 | down-reg. | 9.541 | 9.663 | 9.513 | 10.342 | 10.309 | 10.312 |
| 23527 | ACAP2 | -0.725 | 0.050 | 0.090 | down-reg. | 3.471 | 4.296 | 4.075 | 4.558 | 4.746 | 4.716 |
| 23531 | MMD | -0.716 | 0.005 | 0.093 | down-reg. | 7.028 | 6.787 | 6.834 | 7.650 | 7.607 | 7.539 |
| 23641 | LDOC1 | -0.996 | 0.002 | 0.032 | down-reg. | 10.234 | 10.401 | 10.409 | 11.412 | 11.242 | 11.377 |
| 23786 | BCL2L13 | -0.842 | 0.024 | 0.057 | down-reg. | 5.369 | 5.466 | 5.084 | 6.273 | 5.799 | 6.375 |
| 24137 | KIF4A | -1.799 | 0.006 | 0.003 | down-reg. | 5.632 | 5.692 | 4.958 | 7.170 | 7.238 | 7.272 |
| 25766 | PRPF40B | -0.804 | 0.021 | 0.066 | down-reg. | 6.973 | 7.453 | 7.350 | 7.825 | 8.183 | 8.180 |
| 25897 | RNF19A | -0.709 | 0.003 | 0.096 | down-reg. | 9.815 | 9.808 | 9.948 | 10.510 | 10.642 | 10.546 |
| 25920 | NELFB | -0.871 | 0.002 | 0.052 | down-reg. | 12.185 | 12.191 | 12.088 | 13.113 | 12.944 | 13.018 |
| 26030 | PLEKHG3 | -0.842 | 0.024 | 0.057 | down-reg. | 4.498 | 4.937 | 4.487 | 5.721 | 5.522 | 5.206 |
| 26145 | IRF2BP1 | -0.724 | 0.008 | 0.090 | down-reg. | 8.325 | 8.298 | 8.098 | 9.014 | 8.814 | 9.064 |
| 26147 | PHF19 | -1.114 | 0.002 | 0.021 | down-reg. | 10.508 | 10.502 | 10.341 | 11.676 | 11.470 | 11.548 |
| 26153 | KIF26A | -2.324 | 0.023 | 0.001 | down-reg. | 4.885 | 4.577 | 3.155 | 6.766 | 6.083 | 6.742 |
| 26160 | IFT172 | -1.041 | 0.021 | 0.027 | down-reg. | 3.983 | 3.967 | 3.669 | 5.022 | 4.482 | 5.238 |
| 26278 | SACS | -0.942 | 0.013 | 0.039 | down-reg. | 5.682 | 5.578 | 5.274 | 6.485 | 6.226 | 6.649 |
| 26575 | RGS17 | -1.389 | 0.032 | 0.009 | down-reg. | 2.936 | 3.932 | 3.004 | 5.055 | 4.259 | 4.726 |
| 26873 | OPLAH | -1.369 | 0.002 | 0.010 | down-reg. | 7.710 | 7.864 | 7.606 | 9.131 | 9.036 | 9.120 |
| 27099 | SND1-IT1 | -1.641 | 0.001 | 0.005 | down-reg. | 7.101 | 6.994 | 6.879 | 8.589 | 8.612 | 8.696 |
| 27303 | RBMS3 | -0.935 | 0.013 | 0.040 | down-reg. | 5.654 | 5.967 | 5.641 | 6.882 | 6.760 | 6.426 |
| 28234 | SLCO1B3 | -0.861 | 0.043 | 0.053 | down-reg. | 3.755 | 4.143 | 4.325 | 4.488 | 5.208 | 5.110 |
| 28959 | TMEM176B | -0.776 | 0.029 | 0.074 | down-reg. | 6.753 | 6.883 | 6.769 | 7.926 | 7.605 | 7.201 |
| 29121 | CLEC2D | -0.894 | 0.017 | 0.047 | down-reg. | 4.158 | 4.095 | 4.049 | 4.623 | 5.127 | 5.233 |
| 29775 | CARD10 | -2.457 | 0.004 | 0.001 | down-reg. | 4.578 | 3.902 | 4.030 | 6.350 | 6.636 | 6.895 |
| 29781 | NCAPH2 | -1.426 | 0.001 | 0.009 | down-reg. | 7.234 | 7.283 | 7.056 | 8.619 | 8.616 | 8.616 |
| 29890 | RBM15B | -0.764 | 0.002 | 0.077 | down-reg. | 9.099 | 9.162 | 9.064 | 9.936 | 9.811 | 9.869 |
| 29899 | GPSM2 | -2.103 | 0.002 | 0.002 | down-reg. | 4.332 | 4.371 | 4.114 | 6.346 | 6.180 | 6.601 |
| 29901 | SAC3D1 | -0.928 | 0.003 | 0.041 | down-reg. | 10.648 | 10.711 | 10.490 | 11.641 | 11.460 | 11.532 |
| 29935 | RPA4 | -1.176 | 0.023 | 0.017 | down-reg. | 4.540 | 3.804 | 3.956 | 5.490 | 4.906 | 5.433 |
| 29941 | PKN3 | -1.747 | 0.003 | 0.004 | down-reg. | 7.588 | 7.709 | 7.320 | 9.399 | 9.100 | 9.359 |
| 29969 | MDFIC | -0.811 | 0.015 | 0.065 | down-reg. | 6.549 | 6.431 | 6.080 | 7.305 | 7.016 | 7.171 |
| 29985 | SLC39A3 | -0.737 | 0.013 | 0.085 | down-reg. | 7.145 | 7.117 | 6.919 | 7.562 | 7.912 | 7.919 |
| 30833 | NT5C | -0.858 | 0.003 | 0.054 | down-reg. | 11.859 | 11.846 | 11.678 | 12.756 | 12.621 | 12.580 |
| 50509 | COL5A3 | -1.994 | 0.001 | 0.002 | down-reg. | 8.394 | 8.471 | 8.246 | 10.490 | 10.218 | 10.385 |
| 50619 | DEF6 | -1.948 | 0.000 | 0.002 | down-reg. | 5.840 | 6.011 | 5.840 | 7.807 | 7.858 | 7.872 |
| 50810 | HDGFL3 | -0.810 | 0.009 | 0.065 | down-reg. | 4.896 | 5.254 | 4.999 | 5.739 | 5.989 | 5.850 |
| 50861 | STMN3 | -1.458 | 0.014 | 0.008 | down-reg. | 5.290 | 5.378 | 5.138 | 7.019 | 6.175 | 6.986 |
| 51000 | SLC35B3 | -0.736 | 0.009 | 0.086 | down-reg. | 7.532 | 7.645 | 7.692 | 8.386 | 8.521 | 8.172 |
| 51005 | AMDHD2 | -0.870 | 0.003 | 0.052 | down-reg. | 6.666 | 6.731 | 6.610 | 7.683 | 7.456 | 7.479 |
| 51065 | RPS27L | -0.926 | 0.019 | 0.042 | down-reg. | 6.400 | 6.921 | 7.018 | 7.572 | 7.862 | 7.683 |
| 51087 | YBX2 | -1.038 | 0.017 | 0.027 | down-reg. | 4.801 | 5.171 | 5.057 | 5.671 | 6.273 | 6.200 |
| 51111 | KMT5B | -0.873 | 0.021 | 0.051 | down-reg. | 6.530 | 6.852 | 6.275 | 7.467 | 7.200 | 7.610 |
| 51155 | JPT1 | -0.837 | 0.015 | 0.058 | down-reg. | 5.229 | 5.217 | 5.158 | 6.366 | 5.846 | 5.903 |
| 51181 | DCXR | -0.733 | 0.009 | 0.087 | down-reg. | 7.950 | 7.786 | 7.775 | 8.719 | 8.375 | 8.618 |
| 51196 | PLCE1 | -0.927 | 0.049 | 0.041 | down-reg. | 3.840 | 3.985 | 3.599 | 4.578 | 5.314 | 4.314 |
| 51199 | NIN | -1.626 | 0.002 | 0.005 | down-reg. | 6.832 | 6.698 | 6.474 | 8.307 | 8.227 | 8.347 |
| 51213 | LUZP4 | -0.985 | 0.025 | 0.033 | down-reg. | 3.345 | 2.817 | 3.637 | 4.343 | 4.074 | 4.336 |
| 51236 | HGH1 | -0.798 | 0.014 | 0.068 | down-reg. | 7.274 | 7.153 | 7.111 | 7.943 | 7.742 | 8.245 |
| 51279 | C1RL | -1.023 | 0.008 | 0.029 | down-reg. | 6.859 | 6.842 | 6.457 | 7.883 | 7.693 | 7.650 |
| 51285 | RASL12 | -2.142 | 0.003 | 0.002 | down-reg. | 8.141 | 7.516 | 8.057 | 10.018 | 9.991 | 10.132 |
| 51309 | ARMCX1 | -0.829 | 0.029 | 0.060 | down-reg. | 6.183 | 5.722 | 5.437 | 6.530 | 6.551 | 6.747 |
| 51337 | THEM6 | -0.816 | 0.009 | 0.064 | down-reg. | 9.503 | 9.749 | 9.323 | 10.394 | 10.321 | 10.306 |
| 51386 | EIF3L | -0.804 | 0.015 | 0.067 | down-reg. | 6.083 | 6.192 | 6.033 | 7.172 | 6.644 | 6.903 |
| 51393 | TRPV2 | -2.227 | 0.000 | 0.001 | down-reg. | 10.826 | 10.902 | 10.771 | 13.087 | 13.025 | 13.066 |
| 51397 | COMMD10 | -0.749 | 0.021 | 0.082 | down-reg. | 2.552 | 3.017 | 3.061 | 3.634 | 3.735 | 3.509 |
| 51435 | SCARA3 | -5.914 | 0.000 | 0.000 | down-reg. | 6.679 | 6.561 | 6.129 | 12.284 | 12.302 | 12.525 |
| 51439 | FAM8A1 | -0.827 | 0.004 | 0.061 | down-reg. | 9.033 | 8.789 | 8.810 | 9.654 | 9.720 | 9.739 |
| 51450 | PRRX2 | -1.962 | 0.001 | 0.002 | down-reg. | 10.526 | 10.688 | 10.358 | 12.584 | 12.388 | 12.486 |
| 51545 | ZNF581 | -0.787 | 0.018 | 0.071 | down-reg. | 6.626 | 6.868 | 6.562 | 7.705 | 7.201 | 7.510 |
| 51601 | LIPT1 | -1.100 | 0.020 | 0.022 | down-reg. | 8.153 | 8.330 | 8.036 | 9.700 | 9.241 | 8.877 |
| 51621 | KLF13 | -1.229 | 0.006 | 0.015 | down-reg. | 6.601 | 6.735 | 6.322 | 7.949 | 7.615 | 7.780 |
| 51634 | RBMX2 | -0.875 | 0.022 | 0.051 | down-reg. | 6.821 | 7.262 | 7.027 | 7.621 | 8.208 | 7.905 |
| 51642 | MRPL48 | -1.093 | 0.006 | 0.023 | down-reg. | 8.847 | 9.076 | 8.727 | 10.108 | 9.795 | 10.026 |
| 51655 | RASD1 | -1.382 | 0.002 | 0.010 | down-reg. | 8.036 | 8.183 | 8.070 | 9.618 | 9.393 | 9.422 |
| 51659 | GINS2 | -1.690 | 0.000 | 0.004 | down-reg. | 9.174 | 9.241 | 9.185 | 10.978 | 10.807 | 10.885 |
| 51669 | SARAF | -1.345 | 0.026 | 0.010 | down-reg. | 3.397 | 3.686 | 3.265 | 5.441 | 4.585 | 4.357 |
| 51693 | TRAPPC2L | -0.728 | 0.044 | 0.089 | down-reg. | 7.678 | 8.167 | 7.831 | 8.754 | 8.864 | 8.240 |
| 51752 | ERAP1 | -0.766 | 0.008 | 0.077 | down-reg. | 8.864 | 9.033 | 8.762 | 9.663 | 9.505 | 9.789 |
| 51776 | MAP3K20 | -1.362 | 0.003 | 0.010 | down-reg. | 8.836 | 8.788 | 8.564 | 9.984 | 10.061 | 10.229 |
| 53822 | FXYD7 | -0.890 | 0.029 | 0.048 | down-reg. | 6.248 | 5.702 | 5.482 | 6.626 | 6.600 | 6.877 |
| 53947 | A4GALT | -1.117 | 0.002 | 0.021 | down-reg. | 9.789 | 9.988 | 9.758 | 11.014 | 10.868 | 11.006 |
| 54069 | MIS18A | -1.029 | 0.004 | 0.028 | down-reg. | 6.221 | 6.536 | 6.252 | 7.296 | 7.385 | 7.414 |
| 54328 | GPR173 | -2.841 | 0.001 | 0.001 | down-reg. | 4.744 | 4.572 | 4.795 | 7.743 | 7.335 | 7.554 |
| 54331 | GNG2 | -3.607 | 0.001 | 0.000 | down-reg. | 7.046 | 7.238 | 6.795 | 10.791 | 10.455 | 10.654 |
| 54438 | GFOD1 | -0.987 | 0.004 | 0.033 | down-reg. | 7.665 | 7.683 | 7.550 | 8.766 | 8.463 | 8.631 |
| 54460 | MRPS21 | -0.819 | 0.006 | 0.063 | down-reg. | 8.068 | 7.929 | 7.977 | 8.927 | 8.632 | 8.871 |
| 54478 | PIMREG | -2.989 | 0.000 | 0.000 | down-reg. | 8.365 | 8.229 | 8.205 | 11.276 | 11.296 | 11.194 |
| 54492 | NEURL1B | -2.261 | 0.002 | 0.001 | down-reg. | 6.137 | 6.073 | 5.765 | 8.294 | 8.117 | 8.348 |
| 54541 | DDIT4 | -0.838 | 0.005 | 0.058 | down-reg. | 10.015 | 10.210 | 10.105 | 11.100 | 10.825 | 10.920 |
| 54621 | VSIG10 | -0.749 | 0.013 | 0.082 | down-reg. | 4.695 | 4.619 | 4.861 | 5.405 | 5.312 | 5.706 |
| 54788 | DNAJB12 | -0.729 | 0.004 | 0.088 | down-reg. | 7.673 | 7.585 | 7.771 | 8.458 | 8.315 | 8.442 |
| 54832 | VPS13C | -1.635 | 0.025 | 0.005 | down-reg. | 3.701 | 3.874 | 4.602 | 5.058 | 5.966 | 6.057 |
| 54838 | WBP1L | -0.724 | 0.004 | 0.090 | down-reg. | 8.282 | 8.338 | 8.460 | 8.994 | 9.193 | 9.065 |
| 54855 | TENT5C | -4.706 | 0.001 | 0.000 | down-reg. | 6.448 | 6.583 | 5.771 | 10.944 | 10.962 | 11.014 |
| 54876 | DCAF16 | -1.010 | 0.003 | 0.030 | down-reg. | 9.047 | 9.174 | 8.899 | 10.110 | 9.986 | 10.056 |
| 54881 | TEX10 | -0.981 | 0.003 | 0.034 | down-reg. | 9.654 | 9.832 | 9.649 | 10.650 | 10.608 | 10.821 |
| 54919 | DNAAF5 | -0.918 | 0.001 | 0.043 | down-reg. | 9.111 | 9.059 | 8.976 | 9.991 | 9.911 | 9.997 |
| 54974 | THG1L | -1.321 | 0.004 | 0.011 | down-reg. | 6.759 | 7.011 | 6.585 | 8.114 | 7.989 | 8.215 |
| 54976 | C20orf27 | -0.817 | 0.004 | 0.063 | down-reg. | 11.846 | 11.918 | 11.853 | 12.832 | 12.653 | 12.584 |
| 54978 | SLC35F6 | -0.805 | 0.009 | 0.066 | down-reg. | 10.897 | 10.895 | 10.739 | 11.861 | 11.481 | 11.604 |
| 55018 | ANKRD40CL | -0.780 | 0.009 | 0.072 | down-reg. | 4.250 | 4.252 | 4.394 | 4.852 | 5.187 | 5.197 |
| 55095 | SAMD4B | -1.694 | 0.030 | 0.004 | down-reg. | 3.893 | 4.507 | 3.217 | 5.550 | 5.070 | 6.080 |
| 55096 | EBLN2 | -1.268 | 0.002 | 0.013 | down-reg. | 7.496 | 7.301 | 7.252 | 8.701 | 8.585 | 8.567 |
| 55190 | NUDT11 | -1.052 | 0.016 | 0.026 | down-reg. | 4.960 | 4.471 | 4.675 | 5.621 | 5.559 | 6.080 |
| 55211 | DPPA4 | -1.170 | 0.013 | 0.017 | down-reg. | 4.048 | 4.166 | 4.291 | 4.926 | 5.511 | 5.579 |
| 55268 | ECHDC2 | -1.056 | 0.003 | 0.026 | down-reg. | 9.824 | 9.941 | 9.659 | 10.952 | 10.850 | 10.791 |
| 55339 | WDR33 | -1.284 | 0.016 | 0.012 | down-reg. | 5.500 | 5.689 | 4.855 | 6.549 | 6.533 | 6.814 |
| 55421 | NCBP3 | -0.747 | 0.002 | 0.082 | down-reg. | 7.155 | 7.111 | 7.063 | 7.868 | 7.778 | 7.925 |
| 55623 | THUMPD1 | -0.741 | 0.017 | 0.084 | down-reg. | 7.443 | 7.449 | 7.207 | 8.338 | 7.873 | 8.112 |
| 55646 | LYAR | -1.329 | 0.033 | 0.011 | down-reg. | 3.853 | 3.918 | 4.886 | 5.918 | 5.491 | 5.235 |
| 55700 | MAP7D1 | -0.813 | 0.012 | 0.064 | down-reg. | 4.004 | 3.994 | 3.614 | 4.771 | 4.543 | 4.736 |
| 55702 | YJU2 | -0.907 | 0.005 | 0.045 | down-reg. | 9.206 | 9.280 | 9.158 | 10.273 | 9.934 | 10.158 |
| 55707 | NECAP2 | -0.802 | 0.036 | 0.067 | down-reg. | 5.143 | 5.373 | 4.725 | 6.184 | 5.735 | 5.729 |
| 55771 | PRR11 | -1.239 | 0.012 | 0.014 | down-reg. | 3.615 | 3.128 | 3.552 | 4.796 | 4.337 | 4.880 |
| 55839 | CENPN | -1.203 | 0.004 | 0.016 | down-reg. | 8.227 | 8.378 | 7.999 | 9.446 | 9.331 | 9.434 |
| 55850 | USE1 | -0.818 | 0.003 | 0.063 | down-reg. | 8.094 | 8.035 | 8.126 | 8.944 | 8.774 | 8.991 |
| 55893 | ZNF395 | -1.840 | 0.000 | 0.003 | down-reg. | 8.025 | 7.897 | 7.877 | 9.734 | 9.766 | 9.818 |
| 56256 | SERTAD4 | -1.325 | 0.004 | 0.011 | down-reg. | 4.662 | 4.520 | 4.603 | 5.644 | 6.065 | 6.053 |
| 56658 | TRIM39 | -0.895 | 0.042 | 0.047 | down-reg. | 3.629 | 3.377 | 3.706 | 4.330 | 4.077 | 4.991 |
| 56681 | SAR1A | -0.832 | 0.042 | 0.060 | down-reg. | 5.620 | 5.697 | 5.439 | 5.929 | 6.808 | 6.513 |
| 56731 | SLC2A4RG | -0.943 | 0.003 | 0.039 | down-reg. | 7.300 | 7.445 | 7.412 | 8.384 | 8.199 | 8.403 |
| 56901 | NDUFA4L2 | -0.808 | 0.003 | 0.065 | down-reg. | 6.938 | 6.987 | 6.838 | 7.673 | 7.673 | 7.842 |
| 56904 | SH3GLB2 | -0.870 | 0.002 | 0.052 | down-reg. | 10.978 | 10.924 | 10.872 | 11.874 | 11.698 | 11.810 |
| 56927 | GPR108 | -0.755 | 0.012 | 0.080 | down-reg. | 3.904 | 4.072 | 3.923 | 4.973 | 4.613 | 4.577 |
| 56929 | FEM1C | -1.736 | 0.008 | 0.004 | down-reg. | 5.687 | 5.619 | 5.041 | 6.917 | 7.213 | 7.424 |
| 56944 | OLFML3 | -2.486 | 0.000 | 0.001 | down-reg. | 11.499 | 11.657 | 11.461 | 14.060 | 13.978 | 14.038 |
| 57121 | LPAR5 | -0.968 | 0.012 | 0.035 | down-reg. | 4.951 | 4.962 | 5.091 | 6.282 | 5.911 | 5.714 |
| 57124 | CD248 | -0.985 | 0.001 | 0.033 | down-reg. | 14.375 | 14.318 | 14.418 | 15.296 | 15.385 | 15.385 |
| 57162 | PELI1 | -2.150 | 0.005 | 0.002 | down-reg. | 4.903 | 4.416 | 4.139 | 6.814 | 6.431 | 6.664 |
| 57178 | ZMIZ1 | -0.885 | 0.038 | 0.049 | down-reg. | 4.492 | 4.971 | 4.873 | 5.209 | 5.974 | 5.806 |
| 57522 | SRGAP1 | -1.094 | 0.028 | 0.023 | down-reg. | 3.007 | 3.295 | 3.512 | 4.570 | 4.672 | 3.853 |
| 57537 | SORCS2 | -1.038 | 0.034 | 0.027 | down-reg. | 4.297 | 4.842 | 4.880 | 5.792 | 6.079 | 5.260 |
| 57555 | NLGN2 | -1.131 | 0.001 | 0.020 | down-reg. | 10.321 | 10.396 | 10.445 | 11.543 | 11.438 | 11.572 |
| 57593 | EBF4 | -0.761 | 0.037 | 0.078 | down-reg. | 5.654 | 5.819 | 5.099 | 6.302 | 6.131 | 6.421 |
| 57597 | BAHCC1 | -1.210 | 0.019 | 0.015 | down-reg. | 5.025 | 4.789 | 4.281 | 5.921 | 5.629 | 6.177 |
| 57616 | TSHZ3 | -1.738 | 0.000 | 0.004 | down-reg. | 9.352 | 9.274 | 9.229 | 11.098 | 10.955 | 11.017 |
| 57648 | KIAA1522 | -1.155 | 0.006 | 0.018 | down-reg. | 9.283 | 9.440 | 8.941 | 10.358 | 10.358 | 10.414 |
| 57690 | TNRC6C | -2.260 | 0.004 | 0.001 | down-reg. | 4.465 | 4.938 | 4.239 | 6.959 | 6.742 | 6.722 |
| 57700 | FAM160B1 | -0.823 | 0.002 | 0.062 | down-reg. | 10.810 | 10.817 | 10.654 | 11.646 | 11.535 | 11.568 |
| 57761 | TRIB3 | -1.009 | 0.006 | 0.030 | down-reg. | 9.856 | 10.115 | 9.822 | 11.102 | 10.845 | 10.872 |
| 57819 | LSM2 | -1.142 | 0.006 | 0.019 | down-reg. | 9.971 | 10.091 | 9.663 | 11.180 | 10.920 | 11.050 |
| 58504 | ARHGAP22 | -1.449 | 0.032 | 0.008 | down-reg. | 5.224 | 5.861 | 5.523 | 6.264 | 7.509 | 7.182 |
| 59353 | TMEM35A | -3.297 | 0.003 | 0.000 | down-reg. | 5.734 | 5.550 | 4.859 | 8.846 | 8.602 | 8.587 |
| 60494 | CCDC81 | -1.190 | 0.028 | 0.016 | down-reg. | 3.019 | 3.423 | 3.549 | 5.067 | 4.368 | 4.125 |
| 63827 | BCAN | -1.001 | 0.015 | 0.031 | down-reg. | 4.572 | 3.994 | 4.528 | 5.396 | 5.207 | 5.496 |
| 63922 | CHTF18 | -1.562 | 0.001 | 0.006 | down-reg. | 8.407 | 8.525 | 8.367 | 10.120 | 9.872 | 9.991 |
| 63925 | ZNF335 | -0.745 | 0.005 | 0.083 | down-reg. | 8.367 | 8.487 | 8.365 | 9.295 | 9.049 | 9.111 |
| 63967 | CLSPN | -1.953 | 0.011 | 0.002 | down-reg. | 5.371 | 5.077 | 4.292 | 6.880 | 6.660 | 7.058 |
| 63977 | PRDM15 | -0.772 | 0.010 | 0.075 | down-reg. | 7.469 | 7.800 | 7.529 | 8.516 | 8.236 | 8.362 |
| 64170 | CARD9 | -0.715 | 0.008 | 0.093 | down-reg. | 7.041 | 7.128 | 6.985 | 7.939 | 7.606 | 7.755 |
| 64518 | TEKT3 | -1.278 | 0.044 | 0.012 | down-reg. | 2.796 | 3.640 | 3.740 | 4.992 | 4.944 | 4.073 |
| 64755 | RUSF1 | -0.764 | 0.013 | 0.077 | down-reg. | 7.667 | 7.557 | 7.515 | 8.391 | 8.090 | 8.550 |
| 64773 | PCED1A | -0.977 | 0.002 | 0.034 | down-reg. | 10.396 | 10.439 | 10.250 | 11.399 | 11.289 | 11.328 |
| 64857 | PLEKHG2 | -0.879 | 0.049 | 0.050 | down-reg. | 5.510 | 5.229 | 4.695 | 5.780 | 5.900 | 6.390 |
| 65055 | REEP1 | -1.150 | 0.047 | 0.018 | down-reg. | 3.901 | 3.124 | 3.472 | 5.253 | 4.165 | 4.530 |
| 65057 | ACD | -0.704 | 0.006 | 0.098 | down-reg. | 5.946 | 5.907 | 5.739 | 6.680 | 6.513 | 6.510 |
| 65083 | NOL6 | -0.792 | 0.017 | 0.070 | down-reg. | 7.889 | 8.041 | 7.987 | 9.049 | 8.764 | 8.479 |
| 65108 | MARCKSL1 | -2.731 | 0.001 | 0.001 | down-reg. | 8.254 | 8.149 | 7.961 | 10.826 | 10.761 | 10.971 |
| 78991 | PCYOX1L | -0.805 | 0.016 | 0.066 | down-reg. | 7.180 | 7.160 | 6.749 | 7.961 | 7.659 | 7.885 |
| 78999 | LRFN4 | -1.019 | 0.004 | 0.029 | down-reg. | 9.852 | 10.001 | 9.685 | 10.885 | 10.902 | 10.809 |
| 79026 | AHNAK | -1.458 | 0.002 | 0.008 | down-reg. | 10.635 | 10.829 | 10.552 | 12.193 | 12.076 | 12.121 |
| 79140 | CCDC28B | -1.068 | 0.008 | 0.025 | down-reg. | 6.443 | 6.681 | 6.415 | 7.795 | 7.360 | 7.588 |
| 79144 | PPDPF | -1.016 | 0.002 | 0.029 | down-reg. | 15.097 | 15.296 | 15.076 | 16.135 | 16.192 | 16.192 |
| 79153 | GDPD3 | -3.148 | 0.001 | 0.000 | down-reg. | 5.141 | 4.882 | 5.250 | 8.334 | 8.149 | 8.233 |
| 79156 | PLEKHF1 | -1.520 | 0.001 | 0.007 | down-reg. | 9.403 | 9.482 | 9.693 | 11.045 | 11.062 | 11.032 |
| 79159 | NOL12 | -1.554 | 0.004 | 0.006 | down-reg. | 7.111 | 6.745 | 6.735 | 8.375 | 8.294 | 8.585 |
| 79171 | RBM42 | -0.748 | 0.021 | 0.082 | down-reg. | 6.679 | 6.566 | 6.358 | 7.527 | 7.009 | 7.312 |
| 79191 | IRX3 | -1.492 | 0.003 | 0.007 | down-reg. | 8.137 | 8.153 | 7.844 | 9.685 | 9.369 | 9.554 |
| 79646 | PANK3 | -1.455 | 0.007 | 0.008 | down-reg. | 6.467 | 6.364 | 5.968 | 7.588 | 7.618 | 7.959 |
| 79657 | RPAP3 | -1.380 | 0.044 | 0.010 | down-reg. | 4.188 | 4.641 | 4.149 | 4.857 | 6.230 | 6.031 |
| 79660 | PPP1R3B | -1.633 | 0.004 | 0.005 | down-reg. | 7.705 | 7.804 | 7.379 | 9.482 | 9.076 | 9.231 |
| 79675 | FASTKD1 | -1.353 | 0.024 | 0.010 | down-reg. | 3.328 | 3.722 | 2.699 | 4.800 | 4.727 | 4.281 |
| 79680 | RTL10 | -0.970 | 0.024 | 0.035 | down-reg. | 5.532 | 5.163 | 5.306 | 6.259 | 5.943 | 6.708 |
| 79690 | GAL3ST4 | -2.190 | 0.001 | 0.002 | down-reg. | 8.058 | 8.023 | 7.840 | 10.236 | 10.038 | 10.218 |
| 79723 | SUV39H2 | -0.922 | 0.005 | 0.042 | down-reg. | 3.825 | 3.866 | 3.951 | 5.005 | 4.695 | 4.708 |
| 79762 | C1orf115 | -1.309 | 0.001 | 0.011 | down-reg. | 8.257 | 8.332 | 8.137 | 9.573 | 9.476 | 9.605 |
| 79802 | HHIPL2 | -1.171 | 0.041 | 0.017 | down-reg. | 5.516 | 6.214 | 5.967 | 6.457 | 7.478 | 7.277 |
| 79842 | ZBTB3 | -0.939 | 0.010 | 0.039 | down-reg. | 7.200 | 7.342 | 6.862 | 8.117 | 7.954 | 8.151 |
| 80014 | WWC2 | -1.095 | 0.011 | 0.023 | down-reg. | 3.248 | 3.495 | 2.903 | 4.151 | 4.453 | 4.327 |
| 80115 | BAIAP2L2 | -1.913 | 0.002 | 0.003 | down-reg. | 4.338 | 4.515 | 4.253 | 6.305 | 6.067 | 6.474 |
| 80119 | PIF1 | -1.825 | 0.003 | 0.003 | down-reg. | 7.449 | 7.520 | 7.134 | 9.347 | 9.150 | 9.082 |
| 80128 | TRIM46 | -1.218 | 0.003 | 0.015 | down-reg. | 8.075 | 8.070 | 7.764 | 9.195 | 9.146 | 9.222 |
| 80161 | 0 | -0.951 | 0.005 | 0.038 | down-reg. | 6.410 | 6.423 | 6.298 | 7.345 | 7.138 | 7.501 |
| 80178 | TEDC2 | -1.379 | 0.002 | 0.010 | down-reg. | 7.650 | 7.665 | 7.547 | 9.113 | 8.859 | 9.028 |
| 80279 | CDK5RAP3 | -1.054 | 0.004 | 0.026 | down-reg. | 10.657 | 10.639 | 10.481 | 11.826 | 11.495 | 11.618 |
| 80336 | PABPC1L | -1.782 | 0.003 | 0.003 | down-reg. | 6.887 | 6.952 | 6.477 | 8.600 | 8.446 | 8.615 |
| 80740 | LY6G6C | -0.958 | 0.015 | 0.037 | down-reg. | 8.590 | 8.733 | 8.969 | 9.411 | 9.818 | 9.936 |
| 80863 | PRRT1 | -1.005 | 0.006 | 0.031 | down-reg. | 9.561 | 9.773 | 9.379 | 10.682 | 10.526 | 10.519 |
| 81575 | APOLD1 | -1.563 | 0.015 | 0.006 | down-reg. | 4.866 | 4.292 | 4.659 | 6.542 | 5.651 | 6.315 |
| 81698 | 0 | -2.538 | 0.003 | 0.001 | down-reg. | 6.403 | 6.653 | 5.922 | 9.036 | 8.712 | 8.844 |
| 81844 | TRIM56 | -0.892 | 0.004 | 0.048 | down-reg. | 9.602 | 9.637 | 9.494 | 10.600 | 10.353 | 10.455 |
| 81855 | SFXN3 | -1.526 | 0.000 | 0.006 | down-reg. | 11.481 | 11.488 | 11.434 | 13.035 | 12.936 | 13.011 |
| 81857 | MED25 | -0.699 | 0.001 | 0.100 | down-reg. | 11.826 | 11.849 | 11.796 | 12.544 | 12.453 | 12.571 |
| 83259 | PCDH11Y | -1.395 | 0.021 | 0.009 | down-reg. | 6.080 | 6.495 | 6.455 | 7.255 | 8.279 | 7.681 |
| 83463 | MXD3 | -2.536 | 0.000 | 0.001 | down-reg. | 8.574 | 8.681 | 8.454 | 11.150 | 11.049 | 11.118 |
| 83543 | AIF1L | -0.932 | 0.048 | 0.041 | down-reg. | 3.723 | 4.410 | 3.334 | 4.734 | 4.861 | 4.668 |
| 83752 | LONP2 | -1.155 | 0.003 | 0.018 | down-reg. | 6.908 | 6.841 | 6.703 | 8.103 | 7.803 | 8.010 |
| 83786 | FRMD8 | -0.714 | 0.021 | 0.094 | down-reg. | 7.874 | 7.994 | 7.565 | 8.488 | 8.356 | 8.730 |
| 83849 | SYT15 | -0.857 | 0.016 | 0.054 | down-reg. | 6.129 | 6.033 | 5.622 | 6.731 | 6.679 | 6.945 |
| 83937 | RASSF4 | -1.375 | 0.007 | 0.010 | down-reg. | 7.718 | 7.874 | 7.247 | 9.072 | 8.934 | 8.958 |
| 83987 | CCDC8 | -2.420 | 0.003 | 0.001 | down-reg. | 3.614 | 3.878 | 3.984 | 6.378 | 5.895 | 6.464 |
| 84033 | OBSCN | -1.215 | 0.008 | 0.015 | down-reg. | 5.311 | 5.482 | 5.119 | 6.434 | 6.338 | 6.787 |
| 84062 | DTNBP1 | -0.934 | 0.018 | 0.040 | down-reg. | 6.885 | 6.964 | 6.353 | 7.801 | 7.546 | 7.659 |
| 84080 | ENKD1 | -0.862 | 0.003 | 0.053 | down-reg. | 9.974 | 10.072 | 10.023 | 10.974 | 10.748 | 10.933 |
| 84154 | RPF2 | -1.024 | 0.004 | 0.029 | down-reg. | 8.348 | 8.239 | 8.114 | 9.415 | 9.191 | 9.168 |
| 84163 | GTF2IRD2 | -0.923 | 0.007 | 0.042 | down-reg. | 8.046 | 7.933 | 7.685 | 8.857 | 8.681 | 8.895 |
| 84254 | CAMKK1 | -0.979 | 0.009 | 0.034 | down-reg. | 6.283 | 6.549 | 6.472 | 7.667 | 7.313 | 7.261 |
| 84261 | FBXW9 | -0.906 | 0.014 | 0.045 | down-reg. | 7.484 | 7.687 | 7.137 | 8.341 | 8.221 | 8.465 |
| 84270 | CARD19 | -0.739 | 0.008 | 0.085 | down-reg. | 9.377 | 9.623 | 9.380 | 10.161 | 10.105 | 10.330 |
| 84445 | LZTS2 | -0.961 | 0.003 | 0.036 | down-reg. | 9.918 | 9.800 | 9.766 | 10.895 | 10.648 | 10.825 |
| 84458 | LCOR | -0.699 | 0.035 | 0.100 | down-reg. | 6.345 | 6.586 | 5.901 | 7.079 | 6.974 | 6.876 |
| 84532 | ACSS1 | -2.360 | 0.001 | 0.001 | down-reg. | 6.965 | 7.172 | 6.909 | 9.369 | 9.398 | 9.362 |
| 84542 | KIAA1841 | -1.338 | 0.023 | 0.011 | down-reg. | 5.508 | 4.940 | 4.470 | 6.381 | 6.040 | 6.512 |
| 84553 | FAXC | -0.705 | 0.036 | 0.097 | down-reg. | 5.189 | 5.170 | 5.406 | 5.564 | 6.139 | 6.176 |
| 84623 | KIRREL3 | -1.070 | 0.036 | 0.025 | down-reg. | 5.389 | 5.934 | 5.677 | 6.179 | 7.094 | 6.939 |
| 84627 | ZNF469 | -1.063 | 0.008 | 0.025 | down-reg. | 8.162 | 8.214 | 7.917 | 9.284 | 8.923 | 9.274 |
| 84662 | GLIS2 | -1.491 | 0.002 | 0.007 | down-reg. | 7.296 | 7.217 | 7.032 | 8.810 | 8.530 | 8.677 |
| 84687 | PPP1R9B | -1.027 | 0.001 | 0.028 | down-reg. | 12.142 | 12.314 | 12.151 | 13.235 | 13.243 | 13.210 |
| 84795 | PYROXD2 | -1.003 | 0.001 | 0.031 | down-reg. | 7.791 | 7.940 | 7.761 | 8.842 | 8.795 | 8.862 |
| 84823 | LMNB2 | -1.779 | 0.002 | 0.004 | down-reg. | 8.945 | 8.994 | 8.733 | 10.787 | 10.550 | 10.675 |
| 84858 | ZNF503 | -0.788 | 0.013 | 0.071 | down-reg. | 7.058 | 7.190 | 6.760 | 7.917 | 7.665 | 7.789 |
| 85364 | ZCCHC3 | -0.879 | 0.011 | 0.050 | down-reg. | 7.296 | 7.179 | 6.937 | 7.972 | 7.856 | 8.221 |
| 85369 | STRIP1 | -1.304 | 0.001 | 0.012 | down-reg. | 8.180 | 7.963 | 8.164 | 9.403 | 9.371 | 9.446 |
| 85453 | TSPYL5 | -1.189 | 0.004 | 0.016 | down-reg. | 7.417 | 7.110 | 7.087 | 8.490 | 8.261 | 8.428 |
| 89795 | NAV3 | -1.286 | 0.031 | 0.012 | down-reg. | 4.259 | 5.126 | 3.913 | 5.673 | 5.776 | 5.706 |
| 89845 | ABCC10 | -1.139 | 0.030 | 0.019 | down-reg. | 3.779 | 3.863 | 3.232 | 4.903 | 4.278 | 5.112 |
| 89848 | FCHSD1 | -0.878 | 0.001 | 0.050 | down-reg. | 8.008 | 7.973 | 7.872 | 8.830 | 8.810 | 8.849 |
| 89941 | RHOT2 | -0.727 | 0.003 | 0.089 | down-reg. | 10.519 | 10.583 | 10.396 | 11.272 | 11.196 | 11.211 |
| 90007 | MIDN | -0.815 | 0.005 | 0.064 | down-reg. | 10.120 | 10.271 | 10.097 | 11.139 | 10.890 | 10.905 |
| 90324 | CCDC97 | -0.916 | 0.005 | 0.043 | down-reg. | 9.623 | 9.669 | 9.409 | 10.603 | 10.422 | 10.424 |
| 91151 | TIGD7 | -1.962 | 0.030 | 0.002 | down-reg. | 4.736 | 3.663 | 3.056 | 6.002 | 5.302 | 6.036 |
| 91574 | C12orf65 | -1.100 | 0.028 | 0.022 | down-reg. | 2.878 | 3.234 | 2.831 | 4.544 | 4.095 | 3.606 |
| 91603 | ZNF830 | -0.799 | 0.016 | 0.068 | down-reg. | 6.647 | 6.900 | 6.363 | 7.557 | 7.350 | 7.400 |
| 91608 | RASL10B | -0.830 | 0.002 | 0.060 | down-reg. | 8.933 | 9.089 | 8.987 | 9.800 | 9.817 | 9.881 |
| 91653 | BOC | -0.956 | 0.048 | 0.037 | down-reg. | 3.350 | 3.603 | 4.277 | 4.349 | 4.874 | 4.875 |
| 92170 | MTG1 | -0.802 | 0.003 | 0.067 | down-reg. | 7.631 | 7.692 | 7.520 | 8.440 | 8.319 | 8.493 |
| 92344 | GORAB | -1.101 | 0.028 | 0.022 | down-reg. | 4.404 | 4.452 | 4.553 | 5.016 | 5.673 | 6.023 |
| 92799 | SHKBP1 | -0.724 | 0.002 | 0.090 | down-reg. | 11.557 | 11.522 | 11.529 | 12.305 | 12.172 | 12.302 |
| 93058 | COQ10A | -0.807 | 0.005 | 0.066 | down-reg. | 10.920 | 10.920 | 11.150 | 11.709 | 11.853 | 11.849 |
| 93129 | ORAI3 | -0.915 | 0.002 | 0.043 | down-reg. | 11.568 | 11.662 | 11.565 | 12.590 | 12.406 | 12.544 |
| 93436 | ARMC6 | -0.861 | 0.004 | 0.054 | down-reg. | 7.995 | 7.856 | 7.833 | 8.712 | 8.655 | 8.899 |
| 93654 | ST7-AS2 | -1.490 | 0.019 | 0.007 | down-reg. | 3.085 | 3.248 | 3.136 | 5.220 | 4.645 | 4.074 |
| 94097 | SFXN5 | -1.196 | 0.005 | 0.016 | down-reg. | 7.217 | 7.244 | 6.979 | 8.461 | 8.114 | 8.454 |
| 94234 | FOXQ1 | -1.058 | 0.002 | 0.026 | down-reg. | 7.840 | 7.775 | 7.737 | 8.952 | 8.753 | 8.820 |
| 112464 | CAVIN3 | -1.379 | 0.001 | 0.010 | down-reg. | 13.381 | 13.518 | 13.457 | 14.941 | 14.752 | 14.801 |
| 112476 | PRRT2 | -2.858 | 0.000 | 0.001 | down-reg. | 5.311 | 5.105 | 5.085 | 8.086 | 7.969 | 8.020 |
| 112479 | ERI2 | -0.844 | 0.014 | 0.057 | down-reg. | 6.722 | 6.582 | 6.207 | 7.405 | 7.253 | 7.385 |
| 112885 | PHF21B | -0.741 | 0.019 | 0.084 | down-reg. | 8.038 | 8.216 | 8.523 | 9.179 | 8.882 | 8.938 |
| 113026 | PLCD3 | -0.986 | 0.008 | 0.033 | down-reg. | 11.385 | 11.666 | 11.198 | 12.504 | 12.352 | 12.352 |
| 113146 | AHNAK2 | -1.479 | 0.005 | 0.007 | down-reg. | 4.380 | 4.187 | 4.093 | 5.777 | 5.431 | 5.889 |
| 113452 | TMEM54 | -0.979 | 0.004 | 0.034 | down-reg. | 9.374 | 9.527 | 9.284 | 10.463 | 10.239 | 10.418 |
| 113746 | ODF3 | -0.888 | 0.032 | 0.048 | down-reg. | 3.438 | 4.136 | 3.671 | 4.846 | 4.339 | 4.724 |
| 114783 | LMTK3 | -0.950 | 0.037 | 0.038 | down-reg. | 5.247 | 4.792 | 4.406 | 5.453 | 5.848 | 5.994 |
| 114785 | MBD6 | -0.798 | 0.006 | 0.068 | down-reg. | 10.227 | 10.442 | 10.204 | 11.212 | 10.992 | 11.061 |
| 114787 | GPRIN1 | -1.047 | 0.003 | 0.027 | down-reg. | 8.227 | 8.294 | 8.050 | 9.318 | 9.111 | 9.283 |
| 114794 | ELFN2 | -2.901 | 0.001 | 0.001 | down-reg. | 6.433 | 6.457 | 6.228 | 9.440 | 9.185 | 9.196 |
| 114823 | LENG8 | -1.984 | 0.001 | 0.002 | down-reg. | 10.276 | 10.470 | 10.077 | 12.270 | 12.227 | 12.279 |
| 114881 | OSBPL7 | -1.831 | 0.001 | 0.003 | down-reg. | 7.397 | 7.435 | 7.194 | 9.283 | 9.031 | 9.206 |
| 115110 | 0 | -0.951 | 0.038 | 0.038 | down-reg. | 4.493 | 5.488 | 5.114 | 6.083 | 5.947 | 5.918 |
| 115207 | KCTD12 | -2.618 | 0.001 | 0.001 | down-reg. | 8.527 | 8.375 | 8.217 | 11.061 | 10.967 | 10.947 |
| 115572 | TENT5B | -1.405 | 0.007 | 0.009 | down-reg. | 6.355 | 6.385 | 5.940 | 7.681 | 7.391 | 7.824 |
| 116068 | LYSMD3 | -0.991 | 0.013 | 0.032 | down-reg. | 2.886 | 3.199 | 2.978 | 4.304 | 3.764 | 3.968 |
| 116535 | MRGPRF | -0.713 | 0.012 | 0.094 | down-reg. | 11.136 | 11.402 | 11.067 | 12.042 | 11.777 | 11.925 |
| 118472 | ZNF511 | -0.855 | 0.003 | 0.055 | down-reg. | 10.132 | 10.263 | 10.018 | 11.035 | 10.947 | 10.995 |
| 118881 | COMTD1 | -0.726 | 0.004 | 0.089 | down-reg. | 8.373 | 8.470 | 8.282 | 9.168 | 9.018 | 9.116 |
| 118980 | SFXN2 | -1.920 | 0.007 | 0.003 | down-reg. | 4.838 | 5.587 | 5.666 | 7.321 | 7.176 | 7.355 |
| 119692 | OR51S1 | -0.845 | 0.047 | 0.057 | down-reg. | 4.684 | 4.120 | 3.831 | 4.896 | 5.332 | 4.942 |
| 121793 | TEX29 | -3.097 | 0.001 | 0.000 | down-reg. | 5.781 | 5.396 | 5.457 | 8.748 | 8.569 | 8.608 |
| 122748 | OR11H6 | -1.231 | 0.047 | 0.014 | down-reg. | 3.665 | 4.381 | 4.884 | 5.106 | 5.852 | 5.665 |
| 124222 | PAQR4 | -1.035 | 0.007 | 0.028 | down-reg. | 8.089 | 8.324 | 7.870 | 9.239 | 9.091 | 9.058 |
| 125965 | COX6B2 | -0.858 | 0.013 | 0.054 | down-reg. | 4.780 | 4.652 | 4.751 | 5.583 | 5.860 | 5.315 |
| 127435 | PODN | -0.900 | 0.003 | 0.046 | down-reg. | 9.219 | 9.222 | 9.098 | 10.192 | 10.064 | 9.982 |
| 128653 | C20orf141 | -1.293 | 0.002 | 0.012 | down-reg. | 7.649 | 7.420 | 7.590 | 8.917 | 8.834 | 8.787 |
| 128817 | CSTL1 | -0.847 | 0.016 | 0.056 | down-reg. | 3.520 | 3.514 | 3.305 | 4.513 | 4.383 | 3.983 |
| 130814 | SLC66A3 | -0.705 | 0.021 | 0.097 | down-reg. | 7.293 | 7.519 | 6.964 | 7.995 | 7.891 | 8.006 |
| 131669 | UROC1 | -0.955 | 0.025 | 0.037 | down-reg. | 5.882 | 6.405 | 5.858 | 6.762 | 7.330 | 6.918 |
| 133396 | IL31RA | -1.163 | 0.037 | 0.018 | down-reg. | 4.734 | 5.244 | 4.350 | 6.391 | 5.860 | 5.567 |
| 133619 | PRRC1 | -0.861 | 0.044 | 0.053 | down-reg. | 3.563 | 4.189 | 3.592 | 4.831 | 4.250 | 4.847 |
| 134218 | DNAJC21 | -1.179 | 0.042 | 0.017 | down-reg. | 3.847 | 4.399 | 4.460 | 4.760 | 5.679 | 5.803 |
| 134391 | GPR151 | -0.813 | 0.040 | 0.064 | down-reg. | 4.258 | 4.798 | 4.009 | 4.962 | 5.204 | 5.338 |
| 136259 | KLF14 | -2.138 | 0.003 | 0.002 | down-reg. | 5.506 | 5.139 | 5.028 | 7.519 | 7.114 | 7.453 |
| 137075 | CLDN23 | -1.796 | 0.017 | 0.003 | down-reg. | 5.260 | 5.451 | 4.325 | 6.816 | 6.532 | 7.074 |
| 139285 | AMER1 | -0.850 | 0.014 | 0.056 | down-reg. | 6.458 | 6.530 | 6.215 | 7.391 | 6.988 | 7.377 |
| 139886 | SPIN4 | -1.169 | 0.010 | 0.017 | down-reg. | 4.674 | 4.763 | 4.586 | 5.955 | 5.470 | 6.105 |
| 140576 | S100A16 | -0.698 | 0.004 | 0.100 | down-reg. | 13.846 | 13.861 | 13.738 | 14.584 | 14.396 | 14.557 |
| 140738 | TMEM37 | -1.410 | 0.011 | 0.009 | down-reg. | 3.903 | 3.366 | 3.582 | 4.799 | 4.887 | 5.396 |
| 140766 | ADAMTS14 | -2.843 | 0.003 | 0.001 | down-reg. | 5.116 | 4.871 | 4.246 | 7.629 | 7.474 | 7.658 |
| 142913 | CFL1P1 | -0.860 | 0.019 | 0.054 | down-reg. | 7.941 | 8.046 | 8.162 | 8.646 | 8.818 | 9.266 |
| 143187 | VTI1A | -1.087 | 0.033 | 0.023 | down-reg. | 4.703 | 4.118 | 4.040 | 5.039 | 5.245 | 5.838 |
| 144453 | BEST3 | -1.010 | 0.045 | 0.030 | down-reg. | 3.313 | 3.924 | 3.135 | 4.928 | 4.128 | 4.347 |
| 145200 | LINC00239 | -1.305 | 0.005 | 0.012 | down-reg. | 3.379 | 3.488 | 3.584 | 4.989 | 4.846 | 4.530 |
| 145864 | HAPLN3 | -1.396 | 0.003 | 0.009 | down-reg. | 5.969 | 5.737 | 5.991 | 7.170 | 7.293 | 7.422 |
| 146540 | ZNF785 | -1.307 | 0.009 | 0.012 | down-reg. | 5.094 | 5.621 | 5.194 | 6.711 | 6.737 | 6.382 |
| 146760 | RTN4RL1 | -1.716 | 0.011 | 0.004 | down-reg. | 5.888 | 5.449 | 4.920 | 7.025 | 7.077 | 7.305 |
| 147372 | CCBE1 | -0.757 | 0.005 | 0.079 | down-reg. | 9.867 | 10.015 | 9.800 | 10.753 | 10.552 | 10.648 |
| 148252 | DIRAS1 | -2.971 | 0.000 | 0.000 | down-reg. | 8.572 | 8.714 | 8.452 | 11.657 | 11.511 | 11.481 |
| 148418 | SAMD13 | -0.810 | 0.042 | 0.065 | down-reg. | 3.235 | 2.843 | 3.325 | 4.367 | 3.647 | 3.821 |
| 148479 | PHF13 | -1.652 | 0.025 | 0.005 | down-reg. | 2.727 | 3.712 | 3.903 | 4.663 | 5.210 | 5.425 |
| 149076 | ZNF362 | -1.016 | 0.004 | 0.030 | down-reg. | 8.372 | 8.604 | 8.442 | 9.544 | 9.338 | 9.583 |
| 150290 | DUSP18 | -0.840 | 0.012 | 0.058 | down-reg. | 7.308 | 7.241 | 7.124 | 8.209 | 7.784 | 8.199 |
| 150368 | PHETA2 | -1.328 | 0.003 | 0.011 | down-reg. | 8.360 | 8.496 | 8.201 | 9.704 | 9.518 | 9.820 |
| 150763 | GPAT2 | -2.636 | 0.003 | 0.001 | down-reg. | 4.164 | 3.879 | 3.774 | 6.396 | 6.325 | 7.004 |
| 153571 | C5orf38 | -1.417 | 0.001 | 0.009 | down-reg. | 10.001 | 10.162 | 9.984 | 11.568 | 11.370 | 11.460 |
| 153572 | IRX2 | -2.246 | 0.002 | 0.001 | down-reg. | 6.086 | 6.163 | 5.918 | 8.576 | 8.103 | 8.225 |
| 155368 | METTL27 | -0.860 | 0.002 | 0.054 | down-reg. | 9.887 | 10.064 | 10.053 | 10.834 | 10.905 | 10.845 |
| 158056 | MAMDC4 | -0.811 | 0.002 | 0.065 | down-reg. | 6.962 | 6.979 | 6.882 | 7.732 | 7.690 | 7.833 |
| 158219 | TTC39B | -0.907 | 0.038 | 0.045 | down-reg. | 4.254 | 4.234 | 4.814 | 4.947 | 5.425 | 5.652 |
| 158866 | ZDHHC15 | -1.207 | 0.041 | 0.015 | down-reg. | 3.542 | 4.228 | 3.339 | 4.367 | 5.102 | 5.262 |
| 160622 | TAMALIN | -2.497 | 0.001 | 0.001 | down-reg. | 8.356 | 8.331 | 8.023 | 10.703 | 10.739 | 10.760 |
| 160851 | DGKH | -1.453 | 0.010 | 0.008 | down-reg. | 6.495 | 6.967 | 6.563 | 8.492 | 7.950 | 7.943 |
| 161176 | SYNE3 | -2.314 | 0.049 | 0.001 | down-reg. | 7.499 | 7.513 | 7.227 | 10.660 | 10.368 | 8.153 |
| 162417 | NAGS | -1.446 | 0.004 | 0.008 | down-reg. | 6.599 | 6.527 | 6.210 | 7.952 | 7.753 | 7.971 |
| 163175 | LGI4 | -1.027 | 0.020 | 0.028 | down-reg. | 4.540 | 4.371 | 4.950 | 5.958 | 5.542 | 5.440 |
| 163227 | ZNF100 | -1.515 | 0.016 | 0.007 | down-reg. | 4.658 | 4.669 | 3.975 | 5.530 | 6.061 | 6.255 |
| 167691 | LCA5 | -0.726 | 0.025 | 0.089 | down-reg. | 4.137 | 3.847 | 4.239 | 4.511 | 4.994 | 4.896 |
| 169611 | OLFML2A | -2.629 | 0.002 | 0.001 | down-reg. | 5.645 | 5.721 | 5.195 | 8.208 | 7.987 | 8.254 |
| 170680 | PSORS1C2 | -2.061 | 0.041 | 0.002 | down-reg. | 2.651 | 3.965 | 4.038 | 4.657 | 6.039 | 6.142 |
| 196549 | 0 | -0.750 | 0.004 | 0.081 | down-reg. | 10.439 | 10.460 | 10.353 | 11.293 | 11.057 | 11.152 |
| 197187 | SNAI3-AS1 | -0.716 | 0.009 | 0.093 | down-reg. | 8.240 | 8.279 | 8.075 | 9.064 | 8.748 | 8.930 |
| 197259 | MLKL | -1.189 | 0.007 | 0.016 | down-reg. | 3.360 | 3.454 | 3.182 | 4.672 | 4.650 | 4.242 |
| 197342 | EME2 | -1.417 | 0.002 | 0.009 | down-reg. | 7.909 | 7.596 | 7.838 | 9.247 | 9.164 | 9.183 |
| 197407 | ZNF48 | -0.850 | 0.017 | 0.056 | down-reg. | 3.871 | 4.118 | 4.222 | 5.014 | 5.116 | 4.630 |
| 199720 | GGN | -0.727 | 0.012 | 0.089 | down-reg. | 6.782 | 6.567 | 6.601 | 7.409 | 7.162 | 7.559 |
| 199745 | THAP8 | -0.728 | 0.020 | 0.089 | down-reg. | 5.561 | 5.671 | 5.349 | 6.385 | 5.974 | 6.406 |
| 200407 | CREG2 | -0.959 | 0.029 | 0.037 | down-reg. | 2.670 | 3.117 | 2.556 | 3.762 | 4.078 | 3.382 |
| 200634 | KRTCAP3 | -1.282 | 0.040 | 0.012 | down-reg. | 3.833 | 3.351 | 4.469 | 4.726 | 5.212 | 5.560 |
| 200916 | RPL22L1 | -1.551 | 0.004 | 0.006 | down-reg. | 6.358 | 6.406 | 5.960 | 7.828 | 7.843 | 7.705 |
| 201965 | RWDD4 | -1.206 | 0.048 | 0.015 | down-reg. | 5.699 | 5.843 | 6.057 | 6.300 | 7.643 | 7.274 |
| 205327 | C2orf69 | -1.049 | 0.004 | 0.027 | down-reg. | 8.064 | 8.281 | 7.967 | 9.225 | 9.174 | 9.059 |
| 219539 | YPEL4 | -0.857 | 0.043 | 0.054 | down-reg. | 4.780 | 4.543 | 4.072 | 5.271 | 5.026 | 5.670 |
| 219654 | ZCCHC24 | -1.452 | 0.000 | 0.008 | down-reg. | 12.504 | 12.590 | 12.449 | 13.966 | 13.966 | 13.966 |
| 220323 | OAF | -0.756 | 0.011 | 0.080 | down-reg. | 11.977 | 12.297 | 11.874 | 12.881 | 12.756 | 12.779 |
| 221079 | ARL5B | -0.742 | 0.009 | 0.084 | down-reg. | 7.639 | 7.547 | 7.403 | 8.400 | 8.084 | 8.328 |
| 221756 | 0 | -1.132 | 0.026 | 0.020 | down-reg. | 4.534 | 4.956 | 4.003 | 5.833 | 5.467 | 5.588 |
| 221908 | PPP1R35 | -1.026 | 0.007 | 0.029 | down-reg. | 8.795 | 8.970 | 8.549 | 9.865 | 9.659 | 9.867 |
| 221937 | FOXK1 | -1.521 | 0.001 | 0.007 | down-reg. | 7.722 | 7.692 | 7.556 | 9.108 | 9.183 | 9.241 |
| 222166 | MTURN | -1.433 | 0.001 | 0.008 | down-reg. | 8.762 | 8.801 | 8.642 | 10.247 | 10.032 | 10.223 |
| 245939 | DEFB128 | -1.027 | 0.016 | 0.028 | down-reg. | 4.799 | 4.805 | 5.307 | 5.766 | 6.012 | 6.215 |
| 253260 | RICTOR | -1.228 | 0.022 | 0.015 | down-reg. | 5.303 | 4.929 | 5.000 | 5.783 | 6.718 | 6.415 |
| 253264 | ZNF503-AS1 | -0.979 | 0.023 | 0.034 | down-reg. | 5.622 | 5.707 | 5.172 | 6.153 | 6.514 | 6.772 |
| 254122 | SNX32 | -1.489 | 0.004 | 0.007 | down-reg. | 7.052 | 6.895 | 6.623 | 8.454 | 8.224 | 8.358 |
| 254552 | NUDT8 | -1.911 | 0.001 | 0.003 | down-reg. | 8.333 | 8.380 | 8.130 | 10.171 | 10.158 | 10.247 |
| 254863 | TMEM256 | -0.807 | 0.009 | 0.066 | down-reg. | 7.161 | 7.272 | 6.895 | 8.033 | 7.830 | 7.889 |
| 255193 | CSNK1G2-AS1 | -0.826 | 0.032 | 0.061 | down-reg. | 4.136 | 4.732 | 4.499 | 5.606 | 5.092 | 5.146 |
| 255783 | INAFM1 | -0.967 | 0.004 | 0.036 | down-reg. | 9.595 | 9.829 | 9.611 | 10.696 | 10.538 | 10.702 |
| 256148 | OR4S1 | -0.993 | 0.026 | 0.032 | down-reg. | 4.338 | 4.016 | 4.135 | 4.836 | 5.636 | 4.995 |
| 256435 | ST6GALNAC3 | -2.403 | 0.004 | 0.001 | down-reg. | 2.892 | 3.205 | 3.184 | 5.084 | 5.591 | 5.815 |
| 256892 | OR51F1 | -1.504 | 0.033 | 0.007 | down-reg. | 3.961 | 4.933 | 4.420 | 5.273 | 6.234 | 6.320 |
| 259217 | HSPA12A | -0.915 | 0.020 | 0.043 | down-reg. | 5.111 | 5.641 | 5.132 | 6.117 | 6.446 | 6.067 |
| 261726 | TIPRL | -1.522 | 0.001 | 0.007 | down-reg. | 10.921 | 10.926 | 10.804 | 12.370 | 12.338 | 12.510 |
| 266727 | MDGA1 | -0.706 | 0.027 | 0.097 | down-reg. | 6.865 | 7.212 | 6.796 | 7.676 | 7.422 | 7.892 |
| 282969 | FUOM | -1.360 | 0.019 | 0.010 | down-reg. | 6.255 | 6.780 | 6.657 | 8.419 | 7.819 | 7.535 |
| 283131 | NEAT1 | -0.840 | 0.007 | 0.058 | down-reg. | 6.527 | 6.635 | 6.506 | 7.450 | 7.549 | 7.190 |
| 283149 | BCL9L | -1.424 | 0.007 | 0.009 | down-reg. | 6.974 | 7.098 | 6.860 | 8.744 | 8.163 | 8.298 |
| 283487 | PRECSIT | -0.798 | 0.031 | 0.068 | down-reg. | 6.119 | 5.712 | 5.541 | 6.355 | 6.555 | 6.856 |
| 283592 | FAM181A-AS1 | -0.834 | 0.037 | 0.059 | down-reg. | 4.608 | 4.299 | 4.027 | 4.763 | 5.311 | 5.363 |
| 283651 | 0 | -0.959 | 0.023 | 0.037 | down-reg. | 5.076 | 5.082 | 4.978 | 6.472 | 5.807 | 5.732 |
| 283869 | NPW | -0.761 | 0.003 | 0.078 | down-reg. | 9.272 | 9.247 | 9.342 | 9.939 | 10.123 | 10.081 |
| 284454 | 0 | -1.086 | 0.005 | 0.023 | down-reg. | 5.750 | 5.741 | 6.004 | 7.111 | 6.824 | 6.820 |
| 284618 | RUSC1-AS1 | -1.338 | 0.033 | 0.011 | down-reg. | 5.318 | 4.696 | 4.096 | 6.197 | 5.711 | 6.216 |
| 284723 | SLC25A34 | -1.680 | 0.000 | 0.004 | down-reg. | 6.921 | 7.032 | 6.849 | 8.596 | 8.592 | 8.655 |
| 284865 | AC007663.2 | -0.927 | 0.033 | 0.041 | down-reg. | 3.297 | 3.637 | 3.222 | 4.547 | 4.553 | 3.837 |
| 285768 | LINC01622 | -0.906 | 0.025 | 0.045 | down-reg. | 4.074 | 4.754 | 4.426 | 5.492 | 5.408 | 5.072 |
| 285908 | LINC00174 | -1.802 | 0.014 | 0.003 | down-reg. | 6.202 | 5.308 | 5.163 | 7.111 | 7.377 | 7.592 |
| 286059 | AC107959.2 | -1.069 | 0.031 | 0.025 | down-reg. | 4.303 | 4.387 | 4.465 | 4.883 | 5.581 | 5.899 |
| 286257 | PAXX | -0.706 | 0.003 | 0.097 | down-reg. | 11.318 | 11.231 | 11.212 | 12.048 | 11.859 | 11.973 |
| 286527 | TMSB15B | -1.002 | 0.011 | 0.031 | down-reg. | 6.923 | 6.486 | 6.591 | 7.615 | 7.508 | 7.883 |
| 338707 | B4GALNT4 | -2.038 | 0.009 | 0.002 | down-reg. | 3.635 | 2.941 | 3.120 | 5.282 | 4.837 | 5.691 |
| 339105 | PRSS53 | -0.911 | 0.009 | 0.044 | down-reg. | 6.082 | 5.888 | 6.207 | 6.873 | 6.855 | 7.181 |
| 339231 | ARL16 | -1.163 | 0.017 | 0.018 | down-reg. | 2.548 | 2.762 | 3.200 | 4.298 | 3.751 | 3.951 |
| 339456 | TMEM52 | -1.842 | 0.002 | 0.003 | down-reg. | 5.475 | 5.472 | 5.171 | 7.234 | 7.109 | 7.301 |
| 339768 | ESPNL | -1.118 | 0.007 | 0.021 | down-reg. | 6.758 | 6.838 | 6.431 | 7.948 | 7.649 | 7.783 |
| 340419 | RSPO2 | -0.831 | 0.042 | 0.060 | down-reg. | 4.590 | 5.289 | 4.591 | 5.407 | 5.863 | 5.694 |
| 341116 | MS4A10 | -0.794 | 0.029 | 0.069 | down-reg. | 4.465 | 4.579 | 3.950 | 5.230 | 5.228 | 4.917 |
| 347862 | GATD1 | -0.883 | 0.003 | 0.049 | down-reg. | 12.006 | 12.148 | 11.997 | 13.004 | 12.832 | 12.964 |
| 353376 | TICAM2 | -1.132 | 0.048 | 0.020 | down-reg. | 4.209 | 5.171 | 4.403 | 6.235 | 5.497 | 5.448 |
| 373863 | DND1 | -1.547 | 0.008 | 0.006 | down-reg. | 4.930 | 5.184 | 4.604 | 6.453 | 6.192 | 6.715 |
| 374354 | NHLRC2 | -1.266 | 0.004 | 0.013 | down-reg. | 6.703 | 6.867 | 6.729 | 8.270 | 7.955 | 7.870 |
| 374864 | CCDC178 | -1.143 | 0.013 | 0.019 | down-reg. | 5.237 | 5.327 | 4.930 | 6.026 | 6.642 | 6.257 |
| 374907 | B3GNT8 | -1.060 | 0.003 | 0.025 | down-reg. | 7.575 | 7.370 | 7.301 | 8.549 | 8.418 | 8.461 |
| 376497 | SLC27A1 | -1.924 | 0.003 | 0.003 | down-reg. | 8.650 | 8.806 | 8.334 | 10.632 | 10.475 | 10.453 |
| 379025 | PSMA3-AS1 | -1.560 | 0.006 | 0.006 | down-reg. | 6.872 | 6.833 | 6.281 | 8.292 | 8.087 | 8.287 |
| 387103 | CENPW | -1.767 | 0.004 | 0.004 | down-reg. | 6.272 | 6.166 | 6.181 | 8.263 | 7.622 | 8.035 |
| 387509 | GPR153 | -2.026 | 0.000 | 0.002 | down-reg. | 9.742 | 9.679 | 9.771 | 11.834 | 11.678 | 11.757 |
| 388323 | GLTPD2 | -1.303 | 0.000 | 0.012 | down-reg. | 7.231 | 7.180 | 7.318 | 8.558 | 8.558 | 8.523 |
| 388610 | TRNP1 | -1.161 | 0.003 | 0.018 | down-reg. | 8.538 | 8.449 | 8.450 | 9.765 | 9.475 | 9.681 |
| 389119 | INKA1 | -1.454 | 0.000 | 0.008 | down-reg. | 12.267 | 12.270 | 12.217 | 13.640 | 13.738 | 13.738 |
| 389289 | ANXA2R | -1.268 | 0.014 | 0.013 | down-reg. | 3.557 | 3.797 | 3.752 | 4.554 | 5.002 | 5.354 |
| 389741 | GLIDR | -0.756 | 0.002 | 0.080 | down-reg. | 6.416 | 6.307 | 6.297 | 7.179 | 7.032 | 7.077 |
| 389792 | IER5L | -2.290 | 0.048 | 0.001 | down-reg. | 3.499 | 4.197 | 6.013 | 7.022 | 6.400 | 7.158 |
| 390595 | UBAP1L | -1.718 | 0.006 | 0.004 | down-reg. | 5.636 | 5.544 | 4.991 | 7.127 | 6.927 | 7.272 |
| 400236 | FOXN3-AS1 | -1.258 | 0.017 | 0.013 | down-reg. | 5.422 | 6.158 | 6.044 | 6.860 | 7.342 | 7.194 |
| 401022 | HAGLR | -1.337 | 0.025 | 0.011 | down-reg. | 5.829 | 5.890 | 6.169 | 7.941 | 7.123 | 6.835 |
| 401261 | 0 | -1.557 | 0.024 | 0.006 | down-reg. | 4.508 | 4.305 | 3.417 | 5.649 | 5.283 | 5.969 |
| 401588 | 0 | -1.118 | 0.021 | 0.021 | down-reg. | 4.230 | 4.016 | 4.004 | 5.301 | 4.726 | 5.577 |
| 402778 | IFITM10 | -0.990 | 0.001 | 0.033 | down-reg. | 8.562 | 8.680 | 8.624 | 9.627 | 9.551 | 9.658 |
| 404550 | C16orf74 | -0.763 | 0.016 | 0.078 | down-reg. | 6.562 | 6.574 | 6.185 | 7.244 | 7.030 | 7.337 |
| 441150 | C6orf226 | -0.762 | 0.006 | 0.078 | down-reg. | 8.122 | 8.099 | 7.868 | 8.874 | 8.680 | 8.822 |
| 442425 | FOXB2 | -1.020 | 0.026 | 0.029 | down-reg. | 2.831 | 3.054 | 3.636 | 4.363 | 4.247 | 3.970 |
| 494514 | TYMSOS | -1.829 | 0.003 | 0.003 | down-reg. | 6.716 | 6.639 | 6.318 | 8.544 | 8.307 | 8.310 |
| 606495 | CYB5RL | -0.871 | 0.044 | 0.052 | down-reg. | 5.403 | 5.316 | 4.789 | 5.841 | 5.813 | 6.467 |
| 619190 | 0 | -0.775 | 0.004 | 0.074 | down-reg. | 10.311 | 10.463 | 10.460 | 11.136 | 11.321 | 11.102 |
| 643749 | 0 | -1.019 | 0.031 | 0.029 | down-reg. | 4.719 | 4.031 | 3.927 | 5.209 | 5.516 | 5.009 |
| 644068 | 0 | -2.480 | 0.001 | 0.001 | down-reg. | 7.206 | 7.001 | 7.009 | 9.673 | 9.487 | 9.495 |
| 644656 | AC132192.2 | -1.685 | 0.005 | 0.004 | down-reg. | 3.473 | 4.105 | 3.917 | 5.453 | 5.500 | 5.596 |
| 645958 | 0 | -0.755 | 0.049 | 0.080 | down-reg. | 3.205 | 3.401 | 2.586 | 3.698 | 3.759 | 3.998 |
| 646962 | HRCT1 | -1.743 | 0.006 | 0.004 | down-reg. | 5.211 | 5.391 | 4.977 | 6.583 | 7.203 | 7.022 |
| 653145 | ANXA8 | -1.435 | 0.049 | 0.008 | down-reg. | 5.300 | 5.273 | 5.680 | 7.812 | 6.438 | 6.307 |
| 653319 | KIAA0895L | -1.641 | 0.001 | 0.005 | down-reg. | 8.215 | 8.373 | 8.187 | 9.854 | 9.857 | 9.986 |
| 677767 | SCARNA7 | -0.724 | 0.032 | 0.090 | down-reg. | 3.246 | 3.624 | 3.099 | 3.791 | 4.246 | 4.104 |
| 727910 | TLCD2 | -1.038 | 0.002 | 0.027 | down-reg. | 8.461 | 8.328 | 8.325 | 9.504 | 9.316 | 9.408 |
| 728264 | 0 | -2.838 | 0.004 | 0.001 | down-reg. | 5.449 | 5.484 | 5.515 | 8.862 | 8.310 | 7.791 |
| 728294 | D2HGDH | -1.212 | 0.003 | 0.015 | down-reg. | 9.269 | 9.391 | 9.086 | 10.583 | 10.394 | 10.406 |
| 728586 | CCDC192 | -1.587 | 0.046 | 0.005 | down-reg. | 4.123 | 4.805 | 5.527 | 5.708 | 6.784 | 6.723 |
| 728621 | CCDC30 | -1.540 | 0.005 | 0.006 | down-reg. | 7.971 | 8.315 | 7.986 | 9.463 | 9.891 | 9.539 |
| 729438 | CASTOR2 | -0.815 | 0.003 | 0.064 | down-reg. | 8.820 | 8.895 | 8.818 | 9.760 | 9.528 | 9.691 |
| 731424 | 0 | -1.189 | 0.012 | 0.016 | down-reg. | 3.795 | 3.642 | 3.245 | 4.539 | 4.692 | 5.019 |
| 100128071 | FAM229A | -1.554 | 0.003 | 0.006 | down-reg. | 7.422 | 7.247 | 6.995 | 8.866 | 8.689 | 8.771 |
| 100129034 | 0 | -1.250 | 0.001 | 0.014 | down-reg. | 8.991 | 8.827 | 8.844 | 10.132 | 10.120 | 10.161 |
| 100129550 | LINC02035 | -0.800 | 0.020 | 0.067 | down-reg. | 7.248 | 7.381 | 6.928 | 8.173 | 7.751 | 8.033 |
| 100130148 | MAPT-IT1 | -2.126 | 0.015 | 0.002 | down-reg. | 5.034 | 5.676 | 5.830 | 6.950 | 8.141 | 7.826 |
| 100130417 | LINC02593 | -1.025 | 0.027 | 0.029 | down-reg. | 4.438 | 4.610 | 4.613 | 6.114 | 5.330 | 5.292 |
| 100130691 | AC019080.1 | -0.776 | 0.033 | 0.074 | down-reg. | 5.038 | 5.303 | 4.703 | 5.806 | 5.533 | 6.033 |
| 100130705 | ATP6V1FNB | -0.941 | 0.046 | 0.039 | down-reg. | 3.704 | 3.710 | 3.437 | 5.114 | 4.066 | 4.493 |
| 100132288 | TEKT4P2 | -0.903 | 0.022 | 0.046 | down-reg. | 4.794 | 4.645 | 4.539 | 5.968 | 5.299 | 5.419 |
| 100132677 | BSN-DT | -1.264 | 0.026 | 0.013 | down-reg. | 3.494 | 2.481 | 3.166 | 4.609 | 4.126 | 4.198 |
| 100216546 | 0 | -3.107 | 0.003 | 0.000 | down-reg. | 4.936 | 5.232 | 4.490 | 8.175 | 7.755 | 8.049 |
| 100240734 | 0 | -1.305 | 0.049 | 0.012 | down-reg. | 5.275 | 6.276 | 5.435 | 6.320 | 7.365 | 7.217 |
| 100272217 | 0 | -1.705 | 0.011 | 0.004 | down-reg. | 4.184 | 3.844 | 3.938 | 5.640 | 5.248 | 6.192 |
| 100287569 | LINC00173 | -1.174 | 0.007 | 0.017 | down-reg. | 4.975 | 5.114 | 5.302 | 6.113 | 6.250 | 6.551 |
| 100288152 | 0 | -2.230 | 0.001 | 0.001 | down-reg. | 6.999 | 6.826 | 6.679 | 9.148 | 9.086 | 8.962 |
| 100316904 | SAP25 | -3.435 | 0.001 | 0.000 | down-reg. | 4.341 | 4.383 | 4.761 | 8.099 | 7.864 | 7.827 |
| 100507218 | 0 | -1.102 | 0.007 | 0.022 | down-reg. | 7.916 | 8.064 | 7.871 | 9.116 | 8.785 | 9.255 |
| 100507398 | 0 | -1.316 | 0.010 | 0.011 | down-reg. | 4.332 | 4.014 | 3.714 | 5.097 | 5.534 | 5.377 |
| 101180901 | 0 | -1.317 | 0.006 | 0.011 | down-reg. | 4.538 | 4.149 | 4.607 | 5.810 | 5.848 | 5.587 |
| 101410542 | UCHL1-AS1 | -0.885 | 0.011 | 0.049 | down-reg. | 3.275 | 3.198 | 3.413 | 4.350 | 4.286 | 3.908 |
| 101448202 | 0 | -0.964 | 0.049 | 0.036 | down-reg. | 3.756 | 4.308 | 4.538 | 4.693 | 5.346 | 5.455 |
| 101926917 | 0 | -0.882 | 0.035 | 0.049 | down-reg. | 5.112 | 5.654 | 5.298 | 5.895 | 6.622 | 6.192 |
| 101927380 | 0 | -1.230 | 0.028 | 0.014 | down-reg. | 4.634 | 4.986 | 4.166 | 5.359 | 6.068 | 6.049 |
| 101927683 | LINC01744 | -0.802 | 0.036 | 0.067 | down-reg. | 5.925 | 5.684 | 5.911 | 6.186 | 6.904 | 6.837 |
| 101927832 | PAQR9-AS1 | -0.795 | 0.021 | 0.069 | down-reg. | 4.710 | 4.537 | 4.195 | 5.374 | 5.405 | 5.048 |
| 101927934 | LINC01843 | -0.835 | 0.042 | 0.059 | down-reg. | 5.408 | 5.561 | 4.973 | 6.358 | 5.744 | 6.345 |
| 101928304 | 0 | -1.563 | 0.003 | 0.006 | down-reg. | 7.078 | 7.081 | 6.925 | 8.680 | 8.367 | 8.727 |
| 101928402 | AL121601.1 | -1.170 | 0.016 | 0.017 | down-reg. | 3.789 | 3.904 | 4.439 | 5.201 | 4.980 | 5.460 |
| 101929019 | LINC02270 | -1.039 | 0.013 | 0.027 | down-reg. | 3.618 | 3.361 | 3.257 | 4.658 | 4.561 | 4.134 |
| 101929074 | PIK3CD-AS2 | -0.988 | 0.013 | 0.033 | down-reg. | 6.886 | 6.947 | 6.785 | 7.668 | 7.700 | 8.216 |
| 101929478 | LINGO1-AS2 | -0.796 | 0.003 | 0.069 | down-reg. | 8.370 | 8.463 | 8.467 | 9.128 | 9.234 | 9.326 |
| 101929524 | AC024588.1 | -1.931 | 0.033 | 0.003 | down-reg. | 3.697 | 4.830 | 4.713 | 5.499 | 6.903 | 6.632 |
| 101929736 | STAG3L4 | -0.867 | 0.020 | 0.052 | down-reg. | 5.345 | 4.953 | 5.601 | 6.061 | 6.165 | 6.273 |
| 102724450 | RNF207-AS1 | -0.959 | 0.021 | 0.037 | down-reg. | 5.302 | 5.483 | 4.751 | 6.023 | 6.166 | 6.224 |
| 102724679 | LINC02556 | -1.430 | 0.045 | 0.008 | down-reg. | 4.904 | 5.707 | 4.961 | 5.844 | 7.164 | 6.855 |
| 103344928 | KIAA1614-AS1 | -1.117 | 0.037 | 0.021 | down-reg. | 5.451 | 5.926 | 5.264 | 6.114 | 7.027 | 6.852 |
| 104355141 | LINC01143 | -1.094 | 0.021 | 0.023 | down-reg. | 3.449 | 3.812 | 3.209 | 4.296 | 4.511 | 4.946 |
| 105375013 | HCG20 | -1.068 | 0.030 | 0.025 | down-reg. | 3.772 | 4.596 | 4.153 | 4.905 | 5.301 | 5.519 |
| 105375606 | MNX1-AS2 | -1.228 | 0.049 | 0.015 | down-reg. | 3.657 | 4.490 | 3.190 | 5.049 | 5.313 | 4.657 |
